# Self-reproduction and doubling time limits of different cellular subsystems

**DOI:** 10.1038/s41540-023-00306-4

**Published:** 2023-09-20

**Authors:** Kristo Abner, Peter Šverns, Janar Arold, Indrek Morell, Taivo Lints, Sander Medri, Andrus Seiman, Kaarel Adamberg, Raivo Vilu

**Affiliations:** 1 Center of Food and Fermentation Technologies, Mäealuse 2/4, 12618 Tallinn, Estonia; 2 Department of Chemistry and Biotechnology, School of Science, Tallinn University of Technology, Akadeemia tee 15, 12618 Tallinn, Estonia

**Keywords:** Computational biology and bioinformatics, Systems biology, Computer modelling, Systems analysis

## Abstract

Ribosomes which can self-replicate themselves practically autonomously in beneficial physicochemical conditions have been recognized as the central organelles of cellular self-reproduction processes. The challenge of cell design is to understand and describe the rates and mechanisms of self-reproduction processes of cells as of coordinated functioning of ribosomes and the enzymatic networks of different functional complexity that support those ribosomes. We show that doubling times of proto-cells (ranging from simplest replicators up to those reaching the size of *E. coli*) increase rather with the number of different cell component species than with the total numbers of cell components. However, certain differences were observed between cell components in increasing the doubling times depending on the types of relationships between those cell components and ribosomes. Theoretical limits of doubling times of the self-reproducing proto-cells determined by the molecular parameters of cell components and cell processes were in the range between 6–40 min.

## Introduction

Theoretical studies with attempts to understand the detailed mechanisms of the self-reproduction processes of cellular systems have been published. A uniquely important role of ribosomes in copying themselves and other protein components of cells (polymerases, enzymes) has been noted^1–5^. Ribosomes (more precisely ribosomal proteins) have been recognized as the smallest molecular subsystems of bacterial cells that are capable of carrying out self-reproduction^2,6,7^. The molecular properties of ribosomes determine the absolute theoretical minimal cellular self-reproduction time to be less than 10 min for *E*. *coli*^1,2,6,8–11^. Recently, the analysis of self-reproduction of ribosomal proteins has been extended to systems containing also some other components (e.g., enzymes and RNA) and processes (transcription)^2,5,9,12–14^. However, compared to the simple systems composed of only ribosomal proteins, there has been much less attention and research efforts devoted to the analysis of more complex, heterogeneous systems, and for this reason mechanisms of functioning of these systems more close to real cells have not been elaborated.

In this paper, we show that it is possible to theoretically identify and analyze practically all types of the subsystems of the cells (replicators) that are capable of copying themselves. These subsystems have been defined as the self-reproduction systems (SRS, abbreviations are explained in Supplementary Discussion 1) of the cells. Ribosomes are central units of SRS functioning in the processes of copying mother cells (building daughter cells). The functioning of ribosomes is supported by the enzymes of the metabolic networks (responsible for the synthesis of metabolites and monomers of macromolecules) and by the polymerases (responsible for the synthesis of macromolecules). Different combinations of SRS components (step by step from ribosomal protein complex (RPC) to DNA) were analyzed in the current work using simplified stoichiometric models describing the self-reproduction of single cells (named proto-cells). Self-reproduction is considered in these models explicitly as a doubling process of SRS as the result of patterns of coordinated metabolic reactions, through which the existing proto-cell (mother proto-cell) builds a copy of itself (a daughter proto-cell) during the doubling time of the SRS of the proto-cell (*t*_*d_srs*_, symbols are explained in Supplementary Discussion 2). The values of parameters of proto-cells were calculated assuming that all components of proto-cells are precisely duplicated during *t*_*d_srs*_. The calculations showed that the cell components and processes of the SRS that support ribosomes can be divided into two groups based on their relations to ribosomes and translation. In case of fixed stoichiometric relationships (majority of cell components and processes), the value of *t*_*d_srs*_ increases with the total number of species of all relevant cell components and respective cell processes belonging to the SRS (named as complexity of SRS) but the value of *t*_*d_srs*_ is constant for specific SRS because it does not depend on the number of cell component molecules/complexes in the cell (*N*_*cell_comp*_). In these cases there are also no limits on the sizes of the SRSs. In case of non-stoichiometric relationships (DNA, cell membrane), non-linear dependences between the number of ribosomes in the cell (*N*_*rs*_) or *N*_*cell_comp*_ and *t*_*d_srs*_ were observed at the low values of *N*_*rs*_ due to replication of single genome and due to changes of surface to volume ratio. In that case there are clear limits on the maximal sizes of the SRSs at minimal *t*_*d_srs*_ values but the values of *t*_*d_srs*_ increase with the decrease of sizes of SRSs. In all cases the values of main characteristic parameters were calculated from the models and analyzed in the paper.

## Results and discussion

**Fig. 1:**
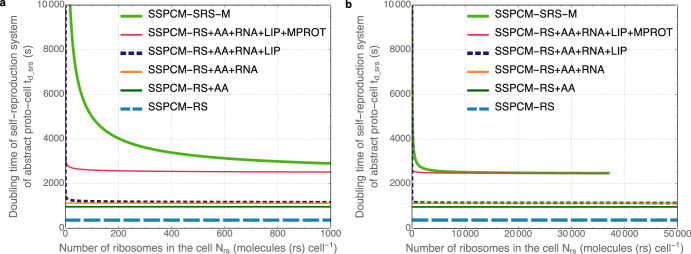
Doubling time of proto-cells depending on their number of ribosomes. *N*_*rs*_ is the number of ribosomes (equal to ribosomal protein complexes) in the cell (molecules (rs) cell^−1^) and *t*_*d_srs*_ is the doubling time of the self-reproduction system (SRS) of the abstract proto-cell (s). Relationships of *N*_*rs*_ and *t*_*d_srs*_ are visualized for different proto-cells (SSPCM-RS, SSPCM-RS + AA, SSPCM-RS + AA + RNA, SSPCM-RS + AA + RNA + LIP, SSPCM-RS + AA + RNA + LIP + MPROT, SSPCM-SRS-M) growing on various media and consisting of different parts of the SRS of SSPCM-SRS-M. The models can be divided into different groups by the type of visualized relationships and doubling time limits (Table 1). The same relationships are shown across two ranges of *N*_*rs*_ values: **a** On this plot the range of *N*_*rs*_ values is limited to low nonphysiological values because this gives the clearest visualization of changes in *t*_*d_srs*_ values. The largest possible values of *N*_*rs*_ for the different models are reported in Table 1. **b** Here the range of *N*_*rs*_ values covers also physiological values of a bacterial cell (*E. coli*). The lines of SSPCM-RS + AA + RNA + LIP + MPROT and SSPCM-SRS-M do not cover all of the range because they reach growth limits there, as described in more detail in section “Ribosomes, RNA, proteins, cell membrane, corresponding monomer synthesis”.

**Fig. 2:**
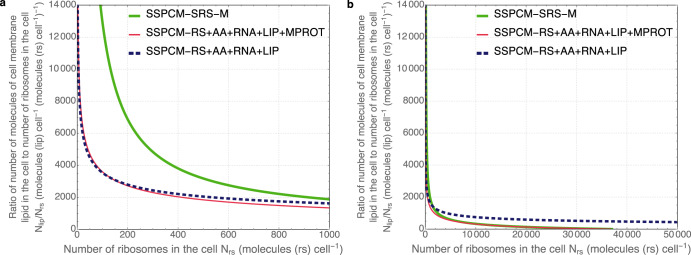
Ratio of membrane lipids to ribosomes in proto-cells. *N*_*lip*_ is the number of cell membrane lipid molecules in the cell (molecules (lip) cell^−1^) and *N*_*rs*_ is the number of ribosomes (equal to ribosomal protein complexes) in the cell (molecules (rs) cell^−1^). Relationships of the ratio of *N*_*lip*_ to *N*_*rs*_ (*N*_*lip*_/*N*_*rs*_, molecules (lip) cell^−1^ (molecules (rs) cell^−1^)^−1^) and *N*_*rs*_ are visualized for different proto-cells (SSPCM-RS + AA + RNA + LIP, SSPCM-RS + AA + RNA + LIP + MPROT, SSPCM-SRS-M) growing on various media and consisting of different parts of the self-reproduction system of SSPCM-SRS-M. **a** On this plot the range of *N*_*rs*_ values is limited to low nonphysiological values to present most clearly the details of the consequences in the changes of the *N*_*lip*_/*N*_*rs*_ values. The largest possible values of *N*_*rs*_ are reported in Table 1. **b** Here the range of *N*_*rs*_ values covers also physiological values of a bacterial cell (*E. coli*). The growth limits (the values of *N*_*lip*_/*N*_*rs*_ touching *x*-axis at maximal values of *N*_*rs*_) of SSPCM-RS + AA + RNA + LIP + MPROT and SSPCM-SRS-M can be seen. The growth limits are reached because the cell membrane surface area is exhausted—it is covered only by membrane proteins. Further faster growth would mean that the requirement of membrane proteins is higher than the cell membrane can accommodate.

The analysis of the theoretical minimal *t*_*d_srs*_ limits that are constrained and affected by the properties of the bacterial cell starts with the identification of cell components necessary in addition to ribosomes or RPC for the self-reproduction of the bacterial cell. These components are briefly described below after the list of used models. Cellular properties that affect theoretical *t*_*d_srs*_ limits are actually combinations of properties of different cell components and processes (combinations of peculiarities of SRS components). Simplified single-cell models of proto-cells (SSPCMs) of increasing complexity (the list is presented below) based on different combinations of SRS components are the main result in this paper together with an analysis of some key characteristics of these models. Our motivation to create such a set of models is based on a goal to develop the foundations of bottom-up ab initio cell design, i.e., to understand how to construct functioning self-replicating cells from basic cell components. Given that ribosomes/RPC are the fundamental biological self-replicators, we take them as the starting point of our analysis, and then build up the complexity by gradually adding various main cell components and processes that support their functioning. Note that not all mathematically possible combinations of SRS components were analyzed, but the selection made was biologically relevant (naturally, RPC or ribosome was present in all the models). The ten models presented in this paper are obviously just a first step of this effort, dealing only with basic proto-cells, but they already allow us to get some important insights into the main functional design principles of cells. In the analysis we have currently focused on the character of the dependence of *t*_*d_srs*_ on the size and complexity of SRS, and the key quantitative results of this analysis are presented on Figs. 1 and 2, and in Table 1. Qualitatively, the key results are that for certain simpler types of basic proto-cell architectures the value of *t*_*d_srs*_ is constant and there is no limitation on the size of the proto-cells, whereas in more complex proto-cells these relationships are hockey-stick-shaped and the sizes of the proto-cells become limited to finite ranges.

**Table 1:** Characteristics of doubling time limits of different proto-cells.

Model	Minimal *t*_*d_srs*_ (s)	Maximal *N*_*rs*_ (molecules (rs) cell^−1^)	Doubling time limit type	Slower growth
SSPCM-RS	362.10	∞	Molecular properties of RPC	–
SSPCM-RS + AA	962.10	∞	Molecular properties of RPC and PW_2_ enzymes	–
SSPCM-RS + AA + PROT	962.10	∞	Molecular properties of RPC	Synthesis of unspecified protein
SSPCM-RS + AA + RNA	1127.15	∞	Molecular properties of ribosomes, RNA polymerase (RP) complex, PW_2_ and PW_4_ enzymes and RNAs	–
SSPCM-RS + AA + RNA + LIP	1127.15	6.19·10^14^	Molecular properties of ribosomes, RP complex, PW_2_ and PW_4_ enzymes and RNAs	Cell geometry
SSPCM-RS + LIP	362.10	1.16·10^28^	Molecular properties of RPC	Cell geometry
SSPCM-RS + AA + RNA + LIP + MPROT	2462.68	36,699	Membrane surface area	Cell geometry
SSPCM-SRS-M	2474.27	36,992	Membrane surface area	Cell geometry and DNA replication
SSPCM-RS + DNA	362.10	7.82·10^16^	Molecular properties of RPC	DNA replication
SSPCM-SRS-R	936.40	24,681	Membrane surface area	Cell geometry and DNA replication

*N*_*rs*_ is the number of ribosomes (equal to ribosomal protein complexes (RPC)) in the cell, *t*_*d_srs*_ is the doubling time of the self-reproduction system (SRS) of the abstract proto-cell. Calculated minimal values of *t*_*d_srs*_, calculated maximal values of *N*_*rs*_ corresponding to minimal *t*_*d_srs*_ values, doubling time limit types and slower growth enabling factors of different proto-cells containing different combinations of SRS components.

The set of models developed for the current work in order to demonstrate different principles of cells is the following:

1. SSPCM-RS describes the growth of proto-cells consisting of only the RPC. The model illustrates the theoretical minimal *t*_*d_srs*_ of self-reproducing proto-cells.
2. SSPCM-RS + AA describes the growth of proto-cells consisting of the RPC and the amino acid biosynthesis pathway PW_2_.
3. SSPCM-RS + AA + PROT describes the growth of proto-cells consisting of the RPC, the amino acid biosynthesis pathway PW_2_ and amino acids of unspecified protein.
4. SSPCM-RS + AA + RNA describes the growth of proto-cells consisting of the RPC and RNA polymerase (RP) complexes, all RNA types, and amino acid and ribonucleotide synthesis pathways (PW_2_, PW_4_).
5. SSPCM-RS + AA + RNA + LIP describes the growth of proto-cells consisting of the RPC, RP complexes, the membrane lipid synthesis enzyme (LPE), all RNA types, membrane lipids, monomer (amino acid, ribonucleotide and lipid precursor) synthesis pathways (PW_2_, PW_4_, PW_5_) and the cell membrane. The model illustrates the effect of the cell membrane on *t*_*d_srs*_.
6. SSPCM-RS + LIP describes the growth of proto-cells consisting of the RPC, the LPE, membrane lipids and the cell membrane. The model was used to outline the special position of membrane lipids among other components of the SRS.
7. SSPCM-RS + AA + RNA + LIP + MPROT describes the growth of proto-cells consisting of the RPC, RP complexes, the LPE, membrane proteins, all RNA types, membrane lipids, monomer (amino acid, ribonucleotide and lipid precursor) synthesis pathways (PW_2_, PW_4_, PW_5_), the central metabolic pathway PW_1_ and the cell membrane.
8. SSPCM-SRS-M describes the growth of proto-cells on a minimal medium and consists of the RPC, RP complexes, the LPE, membrane proteins, the replisome complex (RC), all RNA types, membrane lipids, DNA, monomer (amino acid, deoxyribonucleotide, ribonucleotide and lipid precursor) synthesis pathways (PW_2-5_), the central metabolic pathway PW_1_ and the cell membrane.
9. SSPCM-RS + DNA describes the growth of proto-cells consisting of the RPC, the RC, DNA and the deoxyribonucleotide synthesis pathway PW_3_. The model was used to outline the special position of the genome among other components of the SRS.
10. SSPCM-SRS-R describes the growth of proto-cells on a rich medium and consists of the RPC, RP complexes, the LPE, membrane proteins, the RC, all RNA types, membrane lipids, DNA and the cell membrane.

The logic behind choosing these particular sets of SRS components and constructing these particular models from them is based on a simplified analysis of the current knowledge of known bacterial cells and on identifying the main universal (across the large diversity of bacterial species) modules necessary for self-reproduction. First, polymerization of proteins and nucleic acids (translation, transcription, DNA replication) should be included. As mentioned already several times above, ribosomes or RPC are needed for the synthesis of all relevant proteins, and they are very special components considering their ability to also reproduce their own protein part. Therefore, they form the core of all the listed models. Additionally, all RNA types (rRNA, tRNA, mRNA) synthesized by RP complex and the genome synthesized by RC should be considered. Also, obviously, all monomers of biopolymers must be available together with necessary synthesis pathways. Two different extremes of the reaction networks in very simplified forms making monomers available for the self-reproduction are analyzed: (1) biosynthesis of all monomers from a limited number of external substrates (mostly sugars) by enzymes (SSPCM-SRS-M) or (2) all the monomers needed are assumed to be available from the growth environment (such as yeast hydrolysate) via transporter proteins (SSPCM-SRS-R). Between these two extremes there are also various intermediate possibilities where some monomers or other intermediate metabolites are available from the environment and others are biosynthesized. The bacterial cell also needs energy for the various synthesis and transport processes, thus a simplified scheme of ATP synthesis on the cell membrane representing a simplified respiration process has been included. For cellular containment, a simple bilayer lipid membrane is selected, together with necessary membrane proteins. So, overall, the listed models represent a variety of SRSs of different complexity made of various combinations of important cell components (polymerases^13^, enzymes of metabolic pathways^5,10,15–17^, transporter, electron transport chain (ETC), RNA^6,12,14^, DNA, membrane lipids) and processes (polymerization, biosynthesis) that are inevitably necessary for self-reproduction of the cell. Living cells also contain cell components that are not directly used for self-reproduction of the cell, but these have not been included in the models presented in this paper and, therefore, the cells (models) here are termed proto-cells. Another important feature of cells that is not included in the models presented here is the mechanisms of cell cycle. Both of these omissions are a deliberate choice that follows from the logic of starting with the simplest components and gradually moving up in complexity—these presented models have been developed with extendability in mind and it is possible to later add and analyze these important additional features as well.

The following sections of the paper present the primary analysis and main results of the work in concise form to improve the readability and understanding. However, detailed derivations of equations, explanations of model calculations and of dependencies between model parameters together with comparison of literature data is available in Supplementary Discussions 5.1–5.10 which should corroborate the details of the analysis.

### Ribosomal proteins—minimal core components of the SRS

As already mentioned (see “Introduction”), recognizing the central role of ribosomes in the self-reproduction of the cell allows us to approach the question about the absolute minimal SRS complexity and calculate the absolute theoretical minimal *t*_*d_srs*_ limit for the (proto-)cells. The absolute minimal *t*_*d_srs*_ (the fastest growth) is determined by the time the protein part of ribosomes can self-reproduce in a simplified approach^2,6,9,15,18^. We have calculated the doubling time of a single RPC (*t*_*d_rs*_) from SSPCM-RS and the result (*t*_*d_rs*_ = 362.1 s, Table 1) is comparable to previous estimations in the literature. *t*_*d_rs*_ is not dependent on *N*_*rs*_ and the value of *t*_*d_rs*_ is determined by the ratio of the number of amino acid molecules in RPC (*n*_*rpc*_) to the apparent working rate of ribosome (*k*_*rs*_) (*t*_*d_srs*_ = *n*_*rpc*_/*k*_*rs*_, Fig. 1). The constancy of *t*_*d_rs*_ on *N*_*rs*_ from 0 to ∞ shows that if sets of independent ribosomes could be considered simple proto-cells there is no limitation on the size of the proto-cells. This peculiarity is a very important general feature of the SRS of certain types—with stoichiometric relationships between the ribosomes and the reaction networks that support them.

### Ribosomal proteins and amino acid synthesis

Compared to the previous SSPCM-RS, the next proto-cell (SSPCM-RS + AA) contains, in addition to the RPC, also a single amino acid synthesis pathway PW_2_ consisting of a linear chain of reactions (synthesis of amino acids from available building blocks) catalyzed by enzymes. The doubling time of SSPCM-RS + AA (*t*_*d_rs+PW2*_) is described by Eq. (1):

1 $${t}_{d\_rs+PW2}={t}_{d\_rs}+\frac{{n}_{enz}\cdot {l}_{PW2}}{{k}_{enz}}$$

where *n*_*enz*_ is the number of amino acid molecules in a molecule of the enzyme catalyzing the reactions of amino acid biosynthesis pathway PW_2_, *k*_*enz*_ is the apparent working rate of the same enzyme and *l*_*PW2*_ is the number of reactions in amino acid synthesis pathway PW_2_.

According to Eq. (1), *t*_*d_rs+PW2*_ does not depend on *N*_*rs*_ but is determined by the properties of the macromolecules and by *l*_*PW2*_, and it is the same for the SRS containing one or 10^4^ molecules (rs) cell^−1^ or even more (Fig. 1). Based on Eq. (1) and the values of the corresponding input parameters, it is possible to calculate the value of *t*_*d_rs+PW2*_ (962.1 s) (Table 1). It appears that the addition of the amino acid synthesis pathway PW_2_ increased the value of *t*_*d_srs*_ by 600 s.

The calculated value of *t*_*d_rs+PW2*_ is very low compared to the cell cycle length (*t*_*d*_) values of living cells—*t*_*d*_ of “average” bacterial cells (*E. coli*) would typically be ~3600 s (specific growth rate of the cell culture is 0.7 h^−1^)^19^. If we assume that *t*_*d_rs+PW2*_ = 3600 s, the value of *l*_*PW2*_ needed to reach *t*_*d_rs+PW2*_ = 3600 s in the simple two-component SRS of SSPCM-RS + AA should be 1079.3 reactions PW_2_^−1^, according to Eq. (1). In case cells would contain *N*_*rs*_ = 10^4^ molecules (rs) cell^−1^ and *l*_*PW2*_ = 1079.3 reactions PW_2_^−1^, the number of molecules of the enzyme of amino acid biosynthesis pathway PW_2_ in the cell (*N*_*enz_PW2*_) would be 2.2·10^6^ molecules (enz PW_2_) cell^−1^ (Supplementary Table 29), which is, in fact, a number similar to that of living cells (Table 2 of ref. ^20^). However, it should be emphasized that assuming the existence of very long metabolic pathways supporting RPC functioning is most likely not compatible with the metabolic networks of known bacteria (the total number of reactions of amino acid biosynthesis pathways combined is below 200 for cells of *E*. *coli*). As we shall see, a more reasonable assumption would be that the oversized PW_2_ must be interpreted as a sum of other missing cell components of SRS (e.g., enzymes of different synthesis pathways, polymerases).

Let us assume that a new proto-cell SSPCM-RS + AA + PROT has similar SRS components as in the case of SSPCM-RS + AA, but contains also additional unspecified (their functions are not described) proteins to ensure that *l*_*PW2*_ has a *generic* (terms characterizing conformance between parameter values of used models and living *E*. *coli* cells are explained in Supplementary Discussion 3 and written in italics) value at doubling time of SSPCM-RS + AA + PROT (*t*_*d_rs+PW2+prot*_) = 3600 s according to the Eq. (2):

2 $${t}_{d\_rs+PW2+prot}=\frac{{N}_{aa\_prot}}{{N}_{rs}\cdot {k}_{rs}}+{t}_{d\_rs+PW2}$$

where *N*_*aa_prot*_ is the number of molecules of polymerized amino acid of unspecified protein in the cell. It appears that *t*_*d_rs+PW2+prot*_ depends on the values of *N*_*aa_prot*_ and *N*_*rs*_, and *N*_*aa_prot*_ > 5·10^4^ molecules (aa prot) cell^−1^ for every RPC at *t*_*d_rs+PW2+prot*_ = 3600 s. It is easy to verify that in the case of *N*_*aa_prot*_ = 0, Eq. (2) transforms into Eq. (1). Therefore, the addition of an unspecified protein fraction to the SRS of SSPCM-RS + AA does not increase the value of minimal *t*_*d_rs+PW2+prot*_ (962.1 s) compared to the value of *t*_*d_rs+PW2*_ (Table 1). The dependence between the ratio of *N*_*aa_prot*_ to *N*_*rs*_ and between *t*_*d_rs+PW2+prot*_ is always linear and the value of the ratio increases when *t*_*d_rs+PW2+prot*_ increases.

### Ribosomes, RNA, amino acid and ribonucleotide synthesis

The next SRS case is described by SSPCM-RS + AA + RNA which enables us to analyze the effect of RNA synthesis in addition to protein synthesis to *t*_*d_srs*_. Compared to the previous model (SSPCM-RS + AA + PROT), the new proto-cell contains again the amino acid synthesis pathway PW_2_, but RPC is replaced by functional ribosomes by introducing rRNA. Ribonucleotide synthesis pathway PW_4_, RP complexes and the remaining fractions of RNA (tRNA, mRNA) are included instead of the unspecified protein fraction.

Firstly, the calculations show that the value of the doubling time of SSPCM-RS + AA + RNA (*t*_*d_rs+PW2+rna*_) increases to 1127.15 s compared to *t*_*d_rs+PW2*_ = 962.10 s (Table 1). Secondly, the value of *t*_*d_rs+PW2+rna*_ does not depend on *N*_*cell_comp*_ (Fig. 1) because the numbers of added cell components are fixed to *N*_*rs*_ in relevant equations. However, mathematical relationships between the cell component parameters of the SSPCM-RS + AA + RNA are different from those considered earlier due to the increased number of parameters (also including the number of amino acids in RP complex and apparent working rate of RP complex) and interactions.

### Ribosomes, RNA, proteins, membrane lipids and corresponding monomer synthesis

Cells should also have a membrane around their cytoplasm to avoid the dispersion of cell structures. Lipid synthesis pathway PW_5_, LPE and membrane lipids are included in the next proto-cell (SSPCM-RS + AA + RNA + LIP) in comparison to the previous case (SSPCM-RS + AA + RNA). Enclosing the proto-cell into lipid membrane of defined geometry changes remarkably the mathematical properties of SSPCM-RS + AA + RNA + LIP compared to models in previous sections.

Firstly, the value of the corresponding doubling time (*t*_*d_rs+PW2+rna+lip*_) depends now on *N*_*cell_comp*_ in addition to properties of cell components. In the case of very small and decreasing numbers, the value of *t*_*d_rs+PW2+rna+lip*_ increases exponentially to infinity leading to the hockey-stick-shaped dependence of *t*_*d_rs+PW2+rna+lip*_ on *N*_*rs*_ (Fig. 1). For example, *N*_*rs*_ = 8.17 molecules (rs) cell^−1^ corresponds to *t*_*d_rs+PW2+rna+lip*_ = 1300 s. Such dependence appears because the number of cell membrane lipid molecules in the cell (*N*_*lip*_) is not directly fixed to *N*_*rs*_ in equations but the value is determined by the cell surface area. A considerable increase in the *t*_*d_rs+PW2+rna+lip*_ value is due to the remarkable change in the ratio of *N*_*lip*_ to *N*_*cell_comp*_ of cytosolic cell components (Fig. 2) caused by the change in the surface to volume ratio.

Secondly, in the case of large and increasing *N*_*cell_comp*_ values, the value of *t*_*d_rs+PW2+rna+lip*_ decreases. However, the value it asymptotically approaches is not zero but some specific positive value. It appears that the calculated value of that minimal *t*_*d_rs+PW2+rna+lip*_ is equal to the value of *t*_*d_rs+PW2+rna*_ (Table 1). In such large proto-cells (corresponding to the minimal *t*_*d_rs+PW2+rna+lip*_), the composition is dominated by cytosolic components whereas contributions from membrane lipids and associated cell components (LPE, PW_5_ enzymes) are negligible.

To illustrate the changes in mathematical properties and factors determining the minimal *t*_*d_srs*_ after the inclusion of cell membrane, let us consider a simpler proto-cell (SSPCM-RS + LIP) consisting of RPC (that synthesize themselves and LPE), LPE (that synthesize membrane lipids), and membrane lipids that form the cell membrane. The analysis shows that the corresponding doubling time (*t*_*d_rs+lip*_) depends again on *N*_*cell_comp*_ and the dependence is qualitatively similar to the previous example. The minimal *t*_*d_rs+lip*_ is equal to *t*_*d_rs*_ at very large cell size (Table 1). It means that the removal of all monomer synthesis pathways and RNA decreases considerably the value of *t*_*d_rs+lip*_ compared to previous *t*_*d_srs*_ values but still, minimal *t*_*d_rs+lip*_ is determined only by the properties of ribosomes (Table 1). Unchanged minimal *t*_*d_rs+lip*_ compared to *t*_*d_rs*_ is explained by considerable changes in the ratio of surface to volume of the proto-cell, due to which the membrane lipids form only 10^−7^ % of the macromolecular composition of such large proto-cells. Evidently, such a small fraction cannot have practically any effect on minimal *t*_*d_rs+lip*_.

### Ribosomes, RNA, proteins, cell membrane, corresponding monomer synthesis

The further addition of SRS components (membrane proteins (ETC, substrate transport protein), central metabolic pathway PW_1_) changes mathematical properties in the corresponding proto-cell (SSPCM-RS + AA + RNA + LIP + MPROT) compared to previous cases. Firstly, it increases the calculated minimal corresponding doubling time (*t*_*d_rs+PW2+rna+lip+mprot*_) value to 2462.68 s. Such an increase is considerable (more than 2 times) compared to the value of *t*_*d_rs+PW2+rna*_ (Table 1). Secondly, the inclusion of membrane proteins changes the growth limitation factor as minimal *t*_*d_rs+PW2+rna+lip+mprot*_ is determined by the cell surface. The number of membrane proteins on the cell membrane increases due to the increased demand for energy and substrates. At the same time, the surface area covered by membrane lipids gradually decreases due to changes in the surface area to volume ratio with the increase of the proto-cell size. Eventually, there is not enough area for the lipids on the surface, which makes further growth even theoretically impossible (Fig. 2). Certainly, there is some kind of minimal value (higher than 0) of lipid-covered membrane surface area in living cells that is needed to maintain the integrity of the cell membrane and therefore the minimal physiologically acceptable value of *t*_*d_rs+PW2+rna+lip+mprot*_ should be higher. Note that the growth-limiting factor is different in the two previous steps as the membrane is there covered only by lipids. Finally, *t*_*d_rs+PW2+rna+lip+mprot*_ depends also hyperbolically on *N*_*cell_comp*_ as in the previous three cases (SSPCM-RS + AA + PROT, SSPCM-RS + AA + RNA + LIP and SSPCM-RS + LIP) and the dependence manifests remarkably only in cases of small numbers (Fig. 1). For example, *N*_*rs*_ = 2.22 molecules (rs) cell^-1^ corresponds to *t*_*d_rs+PW2+rna+lip+mprot*_ = 3·10^3^ s. As most of the energy and substrate are consumed by translation, it is reasonable to assume that the numbers of membrane proteins and amino acid synthesis enzymes are almost stoichiometrically fixed to *N*_*rs*_ values. Therefore, the inclusion of these cell components is not responsible for the non-linear dependence between *t*_*d_rs+PW2+rna+lip+mprot*_ and *N*_*rs*_. This dependence is imposed by cell geometry and is preserved also in SSPCM-RS + AA + RNA + LIP + MPROT.

### Complete SRS for minimal medium (SSPCM-SRS-M)

Finally, a single copy of the genome and other cell components associated with the synthesis of DNA (RC, deoxyribonucleotide synthesis pathway PW_3_) are included in the previous proto-cell SSPCM-RS + AA + RNA + LIP + MPROT. Therefore, the complete minimal SRS of a bacterial cell growing on a minimal medium is now described (SSPCM-SRS-M). The calculations show that there is a similar hockey-stick-shaped dependence of the corresponding doubling time (*t*_*d_srs-m*_) on the *N*_*rs*_ (Fig. 1). The minimal value of *t*_*d_srs-m*_ (determined again by the surface area of membrane lipids (Fig. 2)) is ~2474.27 s (lower than most of the experimentally determined values of *E. coli* cells growing on mineral media) meaning that the increase of minimal *t*_*d_srs-m*_ is not considerable compared to the previous step (SSPCM-RS + AA + RNA + LIP + MPROT) (Table 1). At minimal *t*_*d_srs-m*_ the total content of DNA-associated cell components is relatively small (1%) and independent of growth because this model has in all growth conditions only a single genome and a pair of RC. However, during slower growth the total content of DNA-associated cell components is relatively high (75%) and therefore also relatively high mass of other cell components is needed. For example, *N*_*rs*_ = 17.45 molecules (rs) cell^−1^ which corresponds to *t*_*d_srs-m*_ = 10^4^ s is 5 orders of magnitude higher than for the previous proto-cell (SSPCM-RS + AA + RNA + LIP + MPROT).

It must be stressed that DNA has a similar effect on *t*_*d_srs*_ as do membrane lipids and proto-cell geometry although the mathematical properties and corresponding equations are different. To illustrate more the uniqueness of DNA among other cell components, let us examine a simpler proto-cell (SSPCM-RS + DNA) consisting only of RPC (that synthesize themselves and enzymes of PW_3_), pathway PW_3_ with corresponding enzymes (synthesis of deoxyribonucleotides) and DNA. The minimal corresponding doubling time (*t*_*d_rs+dna*_) is exactly equal to the mathematical symbolic solution of *t*_*d_rs*_ and thus determined by the properties of ribosomes and not by the properties of DNA-associated cell components (Table 1). *N*_*rs*_ increases exponentially if *t*_*d_rs+dna*_ decreases. The amount of DNA (and associated synthesis equipment) is negligible compared to the value of *N*_*rs*_ at minimal *t*_*d_rs+dna*_ (Supplementary Table 37). On the other hand, DNA comprises almost 100% of the dry mass of cell at *t*_*d_rs+dna*_ = 10^4^ s where *N*_*rs*_ = 3.4 molecules (rs) cell^−1^ and the cell mass (cell components and water in the cell) (*M*_*tot*_) is 1.63·10^−14^ g cell^−1^.

### Complete SRS for rich medium (SSPCM-SRS-R)

The previous results of proto-cell SRS calculations showed that the increase of lengths of enzyme pathways or adding new cell components to the proto-cell will increase the value of minimal *t*_*d_srs*_. However, if cells are growing on a medium supplemented with all necessary monomers of macromolecules, then biosynthesis pathways of monomers are not needed to feed the ribosomes. There are now less cell components in the proto-cell whose numbers are stoichiometrically fixed to *N*_*rs*_ values compared to SSPCM-SRS-M. Less ribosomes and RP complexes are needed to carry out synthesis of proteins and RNA during the self-reproduction of SSPCM-SRS-R. Calculations show that the minimal value of corresponding doubling time (*t*_*d_srs-r*_) decreases to 936.4 s which is comparable to the value of *t*_*d_rs+PW2*_ and more than 2 times lower than minimal *t*_*d_srs-m*_ (Table 1). The results of the calculations are in agreement with the very well-known fact that growth on rich media is faster than on minimal media. The dependence between *t*_*d_srs-r*_ and *N*_*rs*_ has an already familiar weaker dependence in the case of larger proto-cells and sharp changes in the case of smaller cells. The value of minimal *t*_*d_srs-r*_ is again determined by the cell membrane surface area.

### Summary of results

Results of comprehensive analysis of the functioning of different combinations of SRS components from RPC to minimal functional SRSs based on respective SSPCMs are presented above and summarized in Fig. 1 and Table 1. The cellular components of the SRS that support ribosomes in self-replication can be divided into two groups. The common characteristic of both groups is that all the components are not capable to self-reproduce. The majority of *N*_*cell_comp*_ (proteins, RNA) are stoichiometrically linked to *N*_*rs*_—for example, the fluxes of amino acid synthesis pathway PW_2_ and translation must be balanced. The inclusion of these SRS components (their synthesis reactions) in proto-cells increases the values of *t*_*d_srs*_, but the values of *t*_*d_srs*_ do not depend on the values of *N*_*rs*_ (*N*_*cell_comp*_). The SRSs of this type have no constraints on their sizes—see Fig. 1 and Table 1.

Inclusion of the remaining components (e.g., membrane lipids, DNA, unspecified protein) whose *N*_*cell_comp*_ are not directly stoichiometrically linked to *N*_*rs*_ leads to the hockey-stick-shaped dependence of *t*_*d_srs*_ on *N*_*rs*_ (and *N*_*cell_comp*_) if the values of *N*_*rs*_ are small. It also leads to the growth boundary of the proto-cell (restricted value of minimal *t*_*d_srs*_) at higher values of *N*_*cell_comp*_ (at membrane overload). It must be stressed that *t*_*d_srs*_ depends here on *N*_*cell_comp*_ of all cell components (large proto-cell at minimal *t*_*d_srs*_ and small at slower growth), but the inclusion has little effect on minimal *t*_*d_srs*_ values, as the overall content of cell components belonging to the second group at minimal *t*_*d_srs*_ is very small.

The analysis of the peculiarities of the functioning of the SRSs of different compositions and structures allowed us to understand the basic mechanisms determining the values of *t*_*d_srs*_, and relationships between *M*_*tot*_, *N*_*rs*_ and *t*_*d_srs*_. Smaller values of *t*_*d_srs*_ (faster growth) can be achieved in the case of lower SRS complexity (smaller number of species of nonribosomal components in SRS). For example, the value of the minimal *t*_*d_srs-r*_ (936.4 s, Table 1) was considerably lower than that of the minimal value of *t*_*d_srs-m*_ (2474.27 s, Table 1). Calculated values of *t*_*d_srs*_ comparable to the regular values of *t*_*d*_ of living cells corresponded to very (extremely) small proto-cells (*N*_*cell_comp*_ < 1 molecule (cell comp) cell^−1^), indicating that the doubling of SRS is clearly not sufficient to explain the mechanisms of slow growth of large proto-cells. Cell parameter values that would be physiologically meaningful for a full cell of *E. coli* (based on experimentally observed *M*_*tot*_ range of 10^−13^–10^−12^ g cell^−1^ of *E. coli*) were found only in a very narrow *t*_*d_srs*_ range immediately near minimal *t*_*d_srs*_ and the range width depended on the complexity of SRS. For example, the range of *t*_*d_srs-m*_ was 2474.27–2730 s (~250 s). Note that other cell parameter values of SSPCM-SRS-M (*M*_*tot*_ range of 10^−13^–1.55·10^−12^ g cell^−1^, *N*_*rs*_ range of 1790–36,990 molecules (rs) cell^−1^) varied considerably more than *t*_*d_srs-m*_, which is also illustrated in Fig. 1. In the case of *t*_*d_srs-r*_, the corresponding range was 936.4–960 s (less than 25 s) for values that would be physiologically reasonable for a full *E. coli* cell (*M*_*tot*_ range of 10^−13^–5.5·10^−13^ g cell^−1^, *N*_*rs*_ range of 3920–24,680 molecules (rs) cell^−1^). The differences between the *t*_*d_srs-m*_ and *t*_*d_srs-r*_ ranges can again be explained by different SRS compositions, as the total dry weight content of the ribosomal molecular fraction in the cell was much higher in SSPCM-SRS-R (48–58%) than in SSPCM-SRS-M (23–31%). The comparison of calculated (*t*_*d_srs-r*_, *t*_*d_srs-m*_) and experimentally determined minimal *t*_*d*_ values of *E. coli* showed that the calculated values were mostly considerably lower, although in some cases the differences were not that large and unreachable.

### Discussion

A single ribosome/RPC is the absolute minimal system capable of the highest self-reproduction rate among bacterial cell components in favorable physicochemical conditions where all the necessary substances and energy are available. The absolute minimal *t*_*d_srs*_ can be obtained for cells consisting only of self-reproducing components (such as ribosomes/RPC^6^). The addition of non-self-reproducing components will usually prolong *t*_*d_srs*_. The lowest *t*_*d_srs*_ can be observed in the case of cells that have the lowest SRS complexity and high ribosomal molecular fraction content in the cell. Faster-growing cells might have also faster enzymes, and the nonribosomal part of the SRS might be even smaller compared to slower-growing cells, as shown in the comparison of SSPCM-SRS-M and SSPCM-SRS-R. The complexity of SRS can also be interpreted as an indicator of the growth medium type. Higher complexity of SRS is needed in case of less complex growth media (minimal media). This conclusion is compatible with the generally known fact that growth on rich medium is considerably faster than growth on mineral medium. The values of *t*_*d_srs*_ of different proto-cells (SSPCMs) depended on the molecular properties of cell components (e.g., the number of monomer molecules in a macromolecular cell component molecule/complex (*n*_*cell_comp*_), the apparent working rate of a catalyzing cell component (*k*_*cell_comp*_)), but were relatively independent of the size of SRS (*N*_*cell_comp*_ values) (Fig. 1). However, there are slight differences between cell components in this respect. In case of most cell components of the SRS, *N*_*cell_comp*_ are stoichiometrically fixed with *N*_*rs*_ (such as enzymes of the metabolic network, RNA, and polymerases) in our models that enables the cell to grow with constant *t*_*d_srs*_ independently of its size (Fig. 1). Other cell components of the SRS for which *N*_*cell_comp*_ are not directly determined by *N*_*rs*_ (such as cell membrane lipids and DNA) did not practically change the minimal value of the corresponding *t*_*d_srs*_, but they caused a gradual increase in *t*_*d_srs*_ with decreasing *M*_*tot*_. It should be stressed that the increase in *t*_*d_srs*_ took place at relatively small *M*_*tot*_ values, which in the current calculations were below the usual experimentally determined *M*_*tot*_ values of a full *E. coli* cell. As said, the minimal values of *t*_*d_srs*_ were mostly determined by certain combinations of molecular parameters of SRS components (represented in equations by the doubling time of the cell component molecule/complex or by the time coefficient of the cell component) so that it is possible to talk about “stoichiometric” doubling time limits (especially in the case of proto-cell models with lower complexities) (Table 1). However, the introduction of the cell membrane accompanied by all relevant SRS components (membrane lipids and proteins) led to doubling time limits determined by the geometrical properties of the proto-cell (surface area), which has also been found in experimental physiology studies of cells^21^. Based on that, one possible conclusion would be that cells that can avoid overcrowding the surfaces of the cell membranes would have faster growth. However, it is not possible to link that behavior with certain metabolic types without a detailed analysis of the corresponding SRS.

Several critical points should be considered when interpreting the results of the current work. First, it should be noted that calculated values of theoretical doubling time limits are definitely dependent on the molecular characteristics of cell components and processes and on the values of environmental parameters (e.g., pH, T, concentrations of chemical substances). Environmental parameters can change the kinetics and stoichiometry of cell processes and inhibit growth to such an extent that growth boundaries based on cellular/molecular properties are unreachable. These effects were not considered in the work. Second, only one metabolic type (very simplified heterotrophic growth) was analyzed in the current work. Although some of the conclusions (for example, the results from models with less complex SRS: SSPCM-RS, SSPCM-RS + AA, and SSPCM-RS + AA + RNA) can be expanded to other metabolic types as well (polymerization processes are fairly uniform between bacteria), most of them are not directly applicable considering differences in metabolism and cell architecture, not to mention cellular parameter values. Third, the very simplified model calculations reflected the ideal physiological state of proto-cells, which might not directly represent living cells. Several simplifications and assumptions were applied that were crucial for the obtained results. If some of them would be changed (such as precise balancing of different cell processes or growth depending on *k*_*cell_comp*_ values), then some of the results would also change (such as the ratio of *N*_*cell_comp*_ to *N*_*rs*_ in the case of enzymes and membrane proteins, which might lead to changing *t*_*d_srs*_ values). Finally, the proposed results were obtained from theoretical simplified models of proto-cells (including only necessary parts for self-reproduction). Therefore, they cannot be directly applied to living cells/strains (as already argued before) with complicated metabolic networks and with large number of cell components and growth conditions, which make the precise detection of growth limitation factors very difficult^1^.

However, the modeling framework developed can be extended for further studies to include environmental parameters, different bacterial metabolic types and kinetics needed to address the abovementioned concerns. As always, the current work also raised some new puzzles and questions. The calculations of fully functional SRSs of proto-cells (SSPCM-SRS-M, SSPCM-SRS-R) indicated that quite realistic and physiologically relevant *M*_*tot*_ and *N*_*cell_comp*_ (including *N*_*rs*_) values could be obtained from the models, but they were exclusively limited to the fastest growth region. Although it is possible to increase values of the number of reactions in linear metabolic pathway (i.e., the length of the pathway) PW_i_ in SSPCM-SRS-M to the point where cellular parameters reach values comparable to those of a full *E. coli* cell, additional assumptions are needed in the case of SSPCM-SRS-R. What are the factors that slow down the SRS in this case? How is slower growth (considerably slower than minimal *t*_*d_srs*_) realized in cells? To answer these questions, additional ideas are needed. Moreover, only the SRS of cells was analyzed, but it would be interesting to expand such analysis to complete bacterial cells that also contain other cell components that are not directly necessary for self-reproduction but carry out secondary functions or are useful in biotechnological applications.

## Methods

### General description of models

**Fig. 3:**
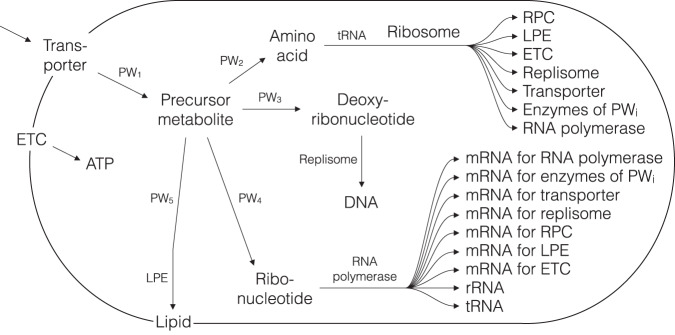
Scheme of the bacterial proto-cell with the simplified metabolic network growing on minimal medium. SSPCM-SRS-M is a simplified single-cell model of the abstract proto-cell growing on minimal medium. SSPCM-SRS-M includes all the main cell components required for self-reproduction—macromolecules (DNA, RNA, proteins, membrane lipids) and their monomers (nucleotides, amino acids, lipids). Localization of the components either in the cytoplasm or in the bilayer cell membrane is considered. An unspecified substrate is transported through the membrane by the membrane transporter and is converted to unspecified metabolic intermediates via a series of reactions of the central metabolic pathway PW_1_. Energy is synthesized by the electron transport chain (ETC) complex on the membrane. The unspecified metabolic intermediates are used to synthesize monomers—amino acids, nucleotides, and lipids—through four different biosynthetic pathways, PW_2_–PW_5_, consisting of sequentially arranged linear chains of reactions. Monomers and energy (ATP) are further utilized by polymerization processes (replication catalyzed by replisome complex, transcription catalyzed by RNA polymerase complex, translation catalyzed by ribosomes (consisting of rRNA and ribosomal protein complex (RPC)), and membrane lipid synthesis catalyzed by membrane lipid synthesis enzyme (LPE)). The stoichiometries of the energizing polymerization reactions are known.

Developed SSPCMs describe the growth of different proto-cells (Supplementary Discussions 5.11.2–5.11.11, Fig. 3 and Supplementary Figs. 1 and 9). A proto-cell is defined in the current work shortly as a cell composed of an (incomplete) set of main cell components needed strictly for self-reproduction. A united set of all used cell components (DNA, RNA, proteins, membrane lipids) and their monomers (nucleotides, amino acids) corresponds to SSPCM-SRS-M (Fig. 3) which is the most complex proto-cell analyzed in this paper.

As can be seen in Fig. 3, SSPCM-SRS-M is structured explicitly into cytoplasmic space and bilayer cell membrane of defined geometry. Most of the cell components are localized in the cytoplasmic space except membrane lipids, substrate transporter proteins and ETC complexes localized on the bilayer cell membrane. The information about the localization of cell components is necessary for including cell geometry and size calculations in the models. The list of cell components of SSPCM-SRS-M includes DNA, RNA, membrane lipids, proteins and monomers of macromolecules (amino acids, nucleotides). The genome of the cell is a bihelix circular chromosome. The RNA fraction consists of diverse RNA types (assembled rRNA sub-units based on 5S, 16S and 23S sub-units with 1:1:1 stoichiometry, tRNA (only one universal tRNA is introduced as different amino acids are not specified) and mRNAs for all proteins (mRNAs contain only coding regions)). Different proteins are considered based on their functions (enzymes, polymerases, membrane proteins). Identical enzymes with *generic* amino acid sequence length catalyze all reactions in central metabolic and biosynthesis pathways PW_1_–PW_5_. Ribosomes, RP complex, RC, LPE carry out polymerization processes. Energy is produced on the membrane by ETC and the substrate is transported to the cell through transport protein. A simplified proto-cell contains only one type of membrane lipid that forms a bilayer cell membrane. The main simplifications of cell components are as follows:

1. All biopolymers are composed of *average* monomers in terms of their mass. For example, the mass of a polymerized amino acid (monomer of proteins) molecule is the arithmetic mean of all 20 amino acids.
2. Biopolymer complexes (assembled rRNA complex, RC, RP, RPC, ETC, transport protein) are single molecules without sub-units (monomeric sequences) in the models for simplification purposes but the values of their monomer sequence lengths correspond to *specific*, *precise* or *approximate* lengths of actual sub-units with appropriate stoichiometries in *E. coli* K12 MG1655.
3. Part of the cell components (mRNA, LPE, enzyme of metabolic pathways PW_1_–PW_5_) have *generic* parameter values. The substrate and the intermediates of the metabolic network are unspecified molecules without specified structure and molecular characteristics (parameters).

There are many kinds of simplifications introduced in the cellular processes and stoichiometric constraints of cell components but the main types of simplifications are as follows:

1. Polymerization processes (DNA replication, transcription, translation) were considered one stage processes starting from the synthesis of primary structures and ending with the formation of mature 3D structures.
2. Metabolic network is composed of 5 linear chains (PW_1-5_) of isomerization reactions with identical unspecific stoichiometries. It means that the numbers of enzyme molecules for each reaction of the same pathway PW_i_ are precisely equal assuming that *k*_*enz*_ is constant. Also, the flux values for each reaction of the same pathway PW_i_ are precisely equal. All reactions are strictly unidirectional which means that there are only net fluxes and exchange fluxes of enzymes operating close to the thermodynamic equilibrium were excluded.
3. Cell has an ideal cylindrical shape with spherical caps which resembles the idealized shape of an *E. coli* cell. It is assumed that the surface areas of both lipid layers are equal.

The self-reproduction processes were assumed to take place according to the following base assumptions. It is assumed that *M*_*tot*_ and *N*_*cell_comp*_ are exactly doubled during *t*_*d_srs*_ by relevant cellular processes carried out in parallel and continuously. The numbers of active cell components (ribosomes, polymerases, enzymes, etc.) are the same during *t*_*d_srs*_. A stationary state condition is assumed and cell metabolism is optimized and organized so that the values of all cell parameters (numbers of cell components, fluxes of reactions/pathways/processes, etc.) are precisely balanced. Due to these base assumptions, the developed modeling approach is stoichiometric, and therefore, all defined synthesis processes can be described by simple algebraic balances explained below. The form of the balance equation (essentially similar to constraint (8) of ref. ^3^) for a general metabolic intermediate on a linear reaction chain (as in pathways PW_1_–PW_5_) is described by Eq. (3):

3 $${N}_{cell\_comp\_cat}\cdot {k}_{cell\_comp}={N}_{cell\_comp\_cat}\cdot {k}_{cell\_comp}$$

where synthesis and degradation reactions are denoted by the number of catalyzing cell component molecules/complexes in the cell (*N*_*cell_comp_cat*_) and their *k*_*cell_comp*_. Multiplications in Eq. (3) are equal to the synthesis and degradation fluxes of the general metabolic intermediate.

The description of polymerization (synthesis of proteins, RNA, DNA) is based on published descriptions of ribosome and RP complex translation growth laws^8,12,22^. The polymerization rate depends on the apparent working rate of polymerase (*k*_*pol*_) and *t*_*d_srs*_. Considering the base assumptions, material balances for biopolymers can be written as a simple non-linear equation (Eq. (4)):

4 $${t}_{d\_srs}=\frac{\sum {N}_{cell\_comp}\cdot {n}_{cell\_comp}}{{N}_{pol}\cdot {k}_{pol}}$$

where *N*_*pol*_ denotes the number of molecules of the corresponding polymerase (ribosome, RP complex and RC) in the cell. All biopolymers must be doubled during *t*_*d_srs*_, and there must be enough catalysts. It must be stressed again that the total number of biopolymers (designated by *N*_*cell_comp*_) polymerized during *t*_*d_srs*_ is precisely equal to the number of biopolymers at the beginning of proto-cell doubling. Therefore, Eq. (4) describes the simplified doubling of biopolymers in the proto-cell.

For example, the symbol *N*_*cell_comp*_ encompasses RPC (*N*_*rs*_) and enzymes from amino acid synthesis pathway PW_2_ (*N*_*enz_PW2*_) in the case of protein synthesis of SSPCM-RS + AA (Eq. (5)). The symbol *n*_*cell_comp*_ takes into account the corresponding amino acid contents of RPC (*n*_*rpc*_) and enzymes (*n*_*enz*_). Correspondingly, *N*_*pol*_ = *N*_*rs*_ and *k*_*pol*_ = *k*_*rs*_:

5 $${t}_{d\_rs+PW2}=\frac{{N}_{rs}\cdot {n}_{rpc}+{N}_{enz\_PW2}\cdot {n}_{enz}}{{N}_{rs}\cdot {k}_{rs}}$$

In the case of SSPCM-RS + AA, Eq. (5) describes the simplified overall synthesis and polymerization of proteins (RPC, enzymes) in the proto-cell. The synthesis of the required number of proteins (described by the numerator of Eq. (5)) for the doubling of the proto-cell is carried out by RPC from amino acids (the denominator of Eq. (5) describes the overall flux of translation as molecules (aa) s^−1^ cell^−1^). Note that the required time for the synthesis of proteins is exactly equal to *t*_*d_rs+PW2*_ because *N*_*rs*_·*k*_*rs*_·*t*_*d_rs+PW2*_ is equal to the required number of polymerized monomers of these proteins. The overall flux of translation is equal to the flux of amino acid synthesis according to Eq. (3), which means that amino acid synthesis takes also place during the whole *t*_*d_rs+PW2*_. Similarly, the polymerization of different biopolymers and synthesis of their monomers are carried out during the whole *t*_*d_srs*_ and in parallel in case of more complicated models. Additional balances are related to the geometry and size of proto-cells. ETC on the cell membrane must produce the exact amount of ATP that is consumed by different monomer biosynthesis pathways (PW_2_–PW_5_), polymerization processes and substrate transport (Fig. 3) to meet the requirements of the precise doubling condition. The surface area of the cell (membrane) is the sum of surface areas of different membrane components (ETC, substrate transporter, membrane lipid) and it depends on the volume of cytoplasmic space of the cell. Note that whereas ETC and transport proteins are needed to carry out cellular processes on the membrane, the number of membrane lipids is solely determined by the remaining surface area. *M*_*tot*_ can be divided roughly into two parts by cell structures and they are the sums of masses of all cell components including water.

The parameters of equations are divided into input (predetermined) and output (calculated using the system of equations) parameters. The number of independent model cell parameters is ~1/3 larger than the number of independent equations of the SSPCM-SRS-M, which means that a smaller number of the parameters’ values must be fixed (input model parameters) to get a determined system with a unique solution. Input parameters for solving the models were chosen mostly among those cell parameters (dimensions and compositions of molecules, stoichiometries of reactions and cellular processes, *k*_*pol*_, etc.) that have constant or relatively unchanging values for a given strain in a given environment. The values of the input parameters of the used models are assumed in most cases to be equal to the characteristics of the respective molecules of *E. coli*. For example, the biopolymers (labeled with terms *average*, *approximate*, *specific*, *precise*) are of the length of corresponding *E. coli* polymers, if possible (otherwise labeled with term *generic*) and they consist of *average* (in terms of mass) monomers. Output parameters of the models were chosen among those cell parameters that lack respective databases and have nonstatic nature (cellular parameters that depend on growth): *N*_*cell_comp*_, fluxes, cell compositions, geometric dimensions and sizes of cells. Sets of calculated values of selected output parameters of all used models are presented in Supplementary Tables 27–30 and 33–38.

Very simplified models were analyzed in this study, as they were considered the most reasonable for presentation and explanation. For example, the metabolic network was composed of linear synthesis pathways without any product synthesis. Additionally, it was expected that enzyme kinetics are ultimately simple (*k*_*cell_comp*_ values are constant). The cell structure was composed of only two compartments, and the number of different cell components was heavily reduced compared to living cells. These simplifications (a more detailed list is available in Supplementary Discussion 5.11.2) were introduced to demonstrate basic capabilities without the complications that would otherwise be caused by the high number of different cell components and the elaborate interactions between them. Such coarse-graining of models has also been successfully applied by others for similar purposes^2,5,10–17^.

### Computations

The modeling work was carried out on a PC, and the used models were coded in the Mathematica 8.0 software environment (Wolfram Research). Wolfram Workbench 2.0 (Wolfram Research) was used for model building and editing. A home-built software tool written in Java was used to carry out semiautomated comparisons of different models in Mathematica m-file format by creating an xls-format report of differences between models. These reports outlined the differences in input variables and equations while ignoring irrelevant aspects such as the order of the variables.

The Mathematica Solve algorithm was used to carry out calculations of models that consisted of determined (equal number of equations and output parameters) systems of non-linear equations. Calculation results were parsed from Mathematica output files, and data arrays were generated in MS Excel using a home-built converter written in Java. The converter also carried out some basic calculations on the data for correlation analysis.

Conventional software tools were used to create schemes of proto-cell models (Fig. 3 and Supplementary Figs. 1–9). Combined Figs. 1 and 2 were created with Mathematica 10 using several functions for the visualization of 2D graphs (Wolfram Research).

### Reporting summary

Further information on research design is available in the Nature Research Reporting Summary linked to this article.

### Supplementary information

Supplementary Information

Reporting Summary

Supplemental Code

## Data Availability

All data analyzed in this article are included in the article and its SI file.

## Code availability

The code of used models in Mathematica m-file format is provided in the Supplementary File.
