# Supplementary material for: Self-reproduction and doubling time limits of different cellular subsystems
Source: NPJ Syst Biol Appl. 2023 Sep 20;9:44. doi: 10.1038/s41540-023-00306-4 (PMC10511633; doi:10.1038/s41540-023-00306-4)
Supplement: Supplementary file 1 — Supplementary Information [file 41540_2023_306_MOESM1_ESM.pdf]

SUPPLEMENTARY DISCUSSION 1: LIST OF ABBREVIATIONS 4
SUPPLEMENTARY DISCUSSION 2: LIST OF SYMBOLS 4
SUPPLEMENTARY DISCUSSION 3: LIST OF TERMS 15
SUPPLEMENTARY DISCUSSION 4: LIST OF SHORT DEFINITIONS 15
SUPPLEMENTARY DISCUSSION 5 16
SUPPLEMENTARY DISCUSSION 5.1: DERIVATIONS, ANALYSIS AND CALCULATIONS BASED ON SSPCM-RS 16
SUPPLEMENTARY DISCUSSION 5.2: DERIVATIONS, ANALYSIS AND CALCULATIONS BASED ON SSPCM-RS+AA 17
SUPPLEMENTARY DISCUSSION 5.3: DERIVATIONS, ANALYSIS AND CALCULATIONS BASED ON SSPCM-RS+AA+PROT 20
SUPPLEMENTARY DISCUSSION 5.4: DERIVATIONS, ANALYSIS AND CALCULATIONS BASED ON SSPCM-RS+AA+RNA 22
SUPPLEMENTARY DISCUSSION 5.5: DERIVATIONS, ANALYSIS AND CALCULATIONS BASED ON SSPCM-RS+AA+RNA+LIP 28
SUPPLEMENTARY DISCUSSION 5.6: DERIVATIONS, ANALYSIS AND CALCULATIONS BASED ON SSPCM-RS+LIP 29
SUPPLEMENTARY DISCUSSION 5.7: DERIVATIONS, ANALYSIS AND CALCULATIONS BASED ON SSPCM-RS+AA+RNA+LIP+MPROT 32
SUPPLEMENTARY DISCUSSION 5.8: DERIVATIONS, ANALYSIS AND CALCULATIONS BASED ON SSPCM-SRS-M 33
SUPPLEMENTARY DISCUSSION 5.9: DERIVATIONS, ANALYSIS AND CALCULATIONS BASED ON SSPCM-RS+DNA 34
SUPPLEMENTARY DISCUSSION 5.10: DERIVATIONS, ANALYSIS AND CALCULATIONS BASED ON SSPCM-SRS-R 35
SUPPLEMENTARY DISCUSSION 5.11: DESCRIPTIONS OF USED MODELS 36
SUPPLEMENTARY DISCUSSION 5.11.1: BASE ASSUMPTIONS OF USED MODELS 37
SUPPLEMENTARY DISCUSSION 5.11.2: DESCRIPTION OF SSPCM-SRS-M 38
Supplementary Discussion 5.11.2.1: Model components and interactions 50
Supplementary Discussion 5.11.2.1.1: Metabolic network 50
Supplementary Discussion 5.11.2.1.2: Protein synthesis 50
Supplementary Discussion 5.11.2.1.3: RNA synthesis 52
Supplementary Discussion 5.11.2.1.4: Lipid synthesis 54
Supplementary Discussion 5.11.2.1.5: Energy balance 54
Supplementary Discussion 5.11.2.1.6: Cell geometry 54
Supplementary Discussion 5.11.2.1.7: Mass balance 55
Supplementary Discussion 5.11.2.1.8: DNA synthesis 55
Supplementary Discussion 5.11.2.2: Model parameters 55
Supplementary Discussion 5.11.2.2.1: Input parameters 56
Supplementary Discussion 5.11.2.2.2: Output parameters 76
Supplementary Discussion 5.11.2.3: Calculation scheme 79
SUPPLEMENTARY DISCUSSION 5.11.3: DESCRIPTION OF SSPCM-RS 84
Supplementary Discussion 5.11.3.1: Model components and interactions 84
Supplementary Discussion 5.11.3.1.1: Protein synthesis 84
Supplementary Discussion 5.11.3.1.2: Mass balance 84
Supplementary Discussion 5.11.3.2: Model parameters 85
Supplementary Discussion 5.11.3.3: Calculation scheme 85
SUPPLEMENTARY DISCUSSION 5.11.4: DESCRIPTION OF SSPCM-RS+AA 85

Supplementary Discussion 5.11.4.1: Model components and interactions 85
Supplementary Discussion 5.11.4.1.1: Metabolic network 86
Supplementary Discussion 5.11.4.1.2: Protein synthesis.. 86
Supplementary Discussion 5.11.4.1.3: Mass balance... 87
Supplementary Discussion 5.11.4.2: Model parameters 87
Supplementary Discussion 5.11.4.3: Calculation scheme...................................... 87
SUPPLEMENTARY DISCUSSION 5.11.5: DESCRIPTION OF SSPCM-RS+AA+PROT 89
Supplementary Discussion 5.11.5.1: Model components and interactions 89
Supplementary Discussion 5.11.5.1.1: Metabolic network 89
Supplementary Discussion 5.11.5.1.2: Protein synthesis.. 89
Supplementary Discussion 5.11.5.1.3: Mass balance... 90
Supplementary Discussion 5.11.5.2: Model parameters 90
Supplementary Discussion 5.11.5.3: Calculation scheme...................................... 90
SUPPLEMENTARY DISCUSSION 5.11.6: DESCRIPTION OF SSPCM-RS+AA+RNA 92
Supplementary Discussion 5.11.6.1: Model components and interactions 92
Supplementary Discussion 5.11.6.1.1: Metabolic network 92
Supplementary Discussion 5.11.6.1.2: Protein synthesis.. 93
Supplementary Discussion 5.11.6.1.3: RNA synthesis 93
Supplementary Discussion 5.11.6.1.4: Mass balance... 94
Supplementary Discussion 5.11.6.2: Model parameters 94
Supplementary Discussion 5.11.6.3: Calculation scheme...................................... 95
SUPPLEMENTARY DISCUSSION 5.11.7: DESCRIPTION OF SSPCM-RS+AA+RNA+LIP 97
Supplementary Discussion 5.11.7.1: Model components and interactions 97
Supplementary Discussion 5.11.7.1.1: Metabolic network 98
Supplementary Discussion 5.11.7.1.2: Protein synthesis.. 98
Supplementary Discussion 5.11.7.1.3: RNA synthesis 98
Supplementary Discussion 5.11.7.1.4: Lipid synthesis... 99
Supplementary Discussion 5.11.7.1.5: Cell geometry... 99
Supplementary Discussion 5.11.7.1.6: Mass balance... 99
Supplementary Discussion 5.11.7.2: Model parameters 100
Supplementary Discussion 5.11.7.3: Calculation scheme.................................... 100
SUPPLEMENTARY DISCUSSION 5.11.8: DESCRIPTION OF SSPCM-RS+LIP...103
Supplementary Discussion 5.11.8.1: Model components and interactions 103
Supplementary Discussion 5.11.8.1.1: Protein synthesis.. 103
Supplementary Discussion 5.11.8.1.2: Lipid synthesis... 104
Supplementary Discussion 5.11.8.1.3: Cell geometry... 104
Supplementary Discussion 5.11.8.1.4: Mass balance... 104

Supplementary Discussion 5.11.8.2: Model parameters 104
Supplementary Discussion 5.11.8.3: Calculation scheme 104
SUPPLEMENTARY DISCUSSION 5.11.9: DESCRIPTION OF SSPCM-RS+RNA+LIP+MPROT 106
Supplementary Discussion 5.11.9.1: Model components and interactions 106
Supplementary Discussion 5.11.9.1.1: Metabolic network 106
Supplementary Discussion 5.11.9.1.2: Protein synthesis... 107
Supplementary Discussion 5.11.9.1.3: RNA synthesis 107
Supplementary Discussion 5.11.9.1.4: Lipid synthesis.. 108
Supplementary Discussion 5.11.9.1.5: Energy balance 108
Supplementary Discussion 5.11.9.1.6: Cell geometry... 108
Supplementary Discussion 5.11.9.1.7: Mass balance... 108
Supplementary Discussion 5.11.9.2: Model parameters 109
Supplementary Discussion 5.11.9.3: Calculation scheme 109
SUPPLEMENTARY DISCUSSION 5.11.10: DESCRIPTION OF SSPCM-RS+DNA 112
Supplementary Discussion 5.11.10.1: Model components and interactions 112
Supplementary Discussion 5.11.10.1.1: Metabolic network 112
Supplementary Discussion 5.11.10.1.2: Protein synthesis....................................... 112
Supplementary Discussion 5.11.10.1.3: Mass balance... 113
Supplementary Discussion 5.11.10.1.4: DNA synthesis 113
Supplementary Discussion 5.11.10.2: Model parameters 113
Supplementary Discussion 5.11.10.3: Calculation scheme 113
SUPPLEMENTARY DISCUSSION 5.11.11: DESCRIPTION OF SSPCM-SRS+R .116
Supplementary Discussion 5.11.11.1: Model components and interactions 116
Supplementary Discussion 5.11.11.1.1: Metabolic network 116
Supplementary Discussion 5.11.11.1.2: Protein synthesis....................................... 117
Supplementary Discussion 5.11.11.1.3: RNA synthesis 117
Supplementary Discussion 5.11.11.1.4: Lipid synthesis.. 118
Supplementary Discussion 5.11.11.1.5: Energy balance 118
Supplementary Discussion 5.11.11.1.6: Cell geometry... 118
Supplementary Discussion 5.11.11.1.7: Mass balance... 118
Supplementary Discussion 5.11.11.1.8: DNA synthesis 118
Supplementary Discussion 5.11.11.2: Model parameters 119
Supplementary Discussion 5.11.11.3: Calculation scheme 119
SUPPLEMENTARY REFERENCES.. 122

Supplementary Discussion 1: List of abbreviations

ETC – electron transport chain.
KEGG – Kyoto Encyclopedia of Genes and Genomes.
LPE – membrane lipid synthesis enzyme.
RC – replisome complex.
RP – RNA polymerase.
RPC – ribosomal protein complex.
SRS – self-reproduction system of the cell (Supplementary Discussion 4).
SSPCM – simplified single-cell model of proto-cell.
SSPCM-RS – simplified single-cell model of the abstract proto-cell (SRS includes only RPC) growing on amino acid medium (Supplementary Discussion 5.11.3).
SSPCM-RS+AA – simplified single-cell model of the abstract proto-cell (SRS includes only RPC and amino acid synthesis) (Supplementary Discussion 5.11.4).
SSPCM-RS+AA+PROT – simplified single-cell model of the abstract proto-cell (SRS includes only RPC, amino acid synthesis and unspecified protein) (Supplementary Discussion 5.11.5).
SSPCM-RS+AA+RNA – simplified single-cell model of the abstract proto-cell (SRS includes only ribosomes, amino acid synthesis, all RNA types and ribonucleotide synthesis) (Supplementary Discussion 5.11.6).
SSPCM-RS+AA+RNA+LIP – simplified single-cell model of the abstract proto-cell (SRS includes all main cell components of SSPCM-SRS-M except DNA replication, deoxyribonucleotide synthesis, membrane proteins and central metabolic pathway) (Supplementary Discussion 5.11.7).
SSPCM-RS+AA+RNA+LIP+MPROT – simplified single-cell model of the abstract proto-cell (SRS includes all main cell components of SSPCM-SRS-M except DNA replication and deoxyribonucleotide synthesis) (Supplementary Discussion 5.11.9).
SSPCM-RS+DNA – simplified single-cell model of the abstract proto-cell (SRS includes only RPC, DNA replication and deoxyribonucleotide synthesis) (Supplementary Discussion 5.11.10).
SSPCM-RS+LIP – simplified single-cell model of the abstract proto-cell (SRS includes only RPC and membrane lipids) (Supplementary Discussion 5.11.8).
SSPCM-SRS-M – simplified single-cell model of the abstract proto-cell (SRS includes all main cell components required for self-reproduction) growing on minimal medium (Supplementary Discussion 5.11.2).
SSPCM-SRS-R – simplified single-cell model of the abstract proto-cell (SRS includes all main cell components required for self-reproduction) growing on rich medium (Supplementary Discussion 5.11.11).

Supplementary Discussion 2: List of symbols

The abbreviations of names of cell components for descriptions of units and indexes of symbols have been written with small letters except for pathway names (PW_1-PW_5). If the description of a symbol does not include terms characterizing values of the corresponding

parameter, these parameters either belong to growth dependent parameter class (usually calculated output parameters or independent variables like most of the t_{d_srs}) by default or they are general designations involving various terms or they are not part of the model. The descriptions of units of parameters (separated by square brackets) have some nontrivial features. Besides usual unit symbols (for example g, cm, cm^2, cm^3, mol, s, h), abbreviations (for example aa, dw) and signs (%), other symbols and abbreviations (defined above and below) have been used that are specific for SSPCMs. Superscript $^{-1}$ with (or without) rounded parentheses designate division operation whereas rounded parentheses without superscript express the relationship or belonging between different terms. For example, the unit of *DWC* [g (dw cell) (g (cell))$^{-1}$] must be interpreted as: grams dry weight of cell per gram of cell. The unit of $cell\_comp\%_{mmc}$ [% (g (tot cell comp) (g (dw cell))$^{-1}$)] must be interpreted as: the per cent of macromolecular fraction based on grams of total amounts of all different cell components of the fraction per gram dry weight of the cell. The term "molecules" must be interpreted as "number of molecules".

$\bar{K}$ – average number of amino acids polymerized per second per ribosome (not part of SSPCM framework, original description from[1]) [amino acids/sec].

f_{ribo}^{active} – the fraction of ribosomes actively engaged in translation (not part of SSPCM framework, original description from[2]) [%].

f_{RNAP}^{active} – the fraction of RNAP actively engaged in transcription (not part of SSPCM framework, original description from[2]) [%].

$N_{RNAP}^{a.a.}$ – the number of amino acids per RNAP (not part of SSPCM framework, original description from[2]) [unit not specified].

N_{ribo}^{nucl} – the number of nucleotides required per ribosome (not part of SSPCM framework, original description from[2]) [unit not specified].

Φ_{RNAP}^{rRNA} – the time fraction active RNAPs dedicated to rRNA synthesis (not part of SSPCM framework, original description from[2]) [%].

Φ_{ribo}^{RNAP} – the time fraction an active ribosome spends synthesizing r-proteins (or, equivalently, the fraction of active ribosomes synthesizing r-proteins) (not part of SSPCM framework, original description from[2]) [%].

aa – amino acid (monomer of proteins).

c – number of amino acids in the protein of a ribosome (not part of SSPCM framework, original description from[1]) [amino acids].

cell_comp – cell component.

$cell\_comp\%_{mmc}$ – dry weight content of macromolecular fraction in the cell [% (g (tot cell comp) (g (dw cell))$^{-1}$)]. $cell\_comp\%_{mmc}$ is the general designation for any of the specific fractions of macromolecular composition including $DNA\%_{mmc}$, $LIP\%_{mmc}$.

cell_comp_cat – general designation for following different catalysing cell components: enzymes of the metabolic network, tRNA, polymerases (RC, RP, RPC/ribosome, LPE) and membrane proteins (ETC, substrate transport protein).

cyt – cytoplasm.

$DNA\%_{mmc}$ – total dry weight content of DNA fraction in the cell [% (g (tot dna) (g (dw cell))$^{-1}$)].

dnt – deoxyribonucleotide (monomer of DNA).

dp – DNA polymerase.

dw – dry weight.

DWC – dry weight content of the cell (*approximate*, see (Supplementary Discussion 3)) [g (dw cell) (g (cell))$^{-1}$].
enz – enzyme of central and biosynthesis pathways.
etc – electron transport chain.
F_{cell_comp} – metabolic flux of reaction/pathway/process [molecules (metabolite) s^{-1} cell^{-1}]. F_{cell_comp} is the general designation for any of the specific metabolic fluxes of different reactions/pathways/processes including $F_{enz\_PWi\_r}$, $F_{enz\_PW2\_r}$, F_{rs}.
$F_{enz\_PW2\_r}$ – flux of reaction r of amino acid synthesis pathway PW_2 [molecules (metabolite) s^{-1} cell^{-1} reaction^{-1}].
$F_{enz\_PWi\_r}$ – flux of reaction r of pathway PW_i [molecules (metabolite) s^{-1} cell^{-1} reaction^{-1}].
F_{rs} – translation flux [molecules (aa) s^{-1} cell^{-1}].
H_{cyl} – length of the cylindrical part of the cell [cm (cell)].
h_{etc} – height of the electron transport chain complex (*generic*, see (Supplementary Discussion 3)) [cm etc^{-1}].
h_{mprot} – the height of the membrane protein (transporter, electron transport chain) complex assuming an ideal cylindrical shape [cm mprot^{-1}]. h_{mprot} is the general designation for any of the following specific heights of different protein complexes: h_{etc}, h_{stp}.
HR – the ratio of the length of the cylindrical part of the cell to cell radius (*approximate*) [cm (cylinder) (cm (radius))$^{-1}$]. The parameter was previously designated as *α*[3] but the designation was changed in the current work for clarification purposes (*HR* refers to cell height and cell radius).
h_{stp} – the height of the transport protein (*generic*) complex [cm stp^{-1}].
k_{cell_comp} – the apparent working rate of catalysing cell component [molecules (metabolite) s^{-1} (cell comp)$^{-1}$]. k_{cell_comp} is the general designation for any of the following specific apparent working rates of different catalysing cell components: k_{trna}, k_{pol}, k_{enz}, k_{etc}, k_{stp}.
k_{dp} – the apparent working rate of DNA polymerase (*approximate* except for faster growth regions of SSPCM-RS+DNA and SSPCM-SRS-R if $t_{d\_srs} < t_C$ = 2319.84 s (Supplementary Table 24)) [molecules (dnt) s^{-1} dp^{-1}].
k_{enz} – the apparent working rate of the enzyme catalysing intracellular reactions of central and biosynthesis pathways PW_1 – PW_5 (*generic*) [molecules (metabolite) s^{-1} enz^{-1}].
k_{etc} – the apparent working rate of electron transport chain complex (*generic*) [molecules (atp) s^{-1} etc^{-1}].
k_{lpe} – the apparent working rate of membrane lipid synthesis enzyme (*generic*) [molecules (lip) s^{-1} lpe^{-1}].
k_{pol} – the apparent working rate of polymerase [molecules (mon) s^{-1} pol^{-1}]. k_{pol} is the general designation for any of the following specific apparent working rates of different polymerases: k_{dp}, k_{lpe}, k_{rp}, k_{rs}.
k_{ribo} – average translation rate (peptide chain elongation rate) (not part of SSPCM framework, original description from[2]) [a.a./sec].
k_{RNAP} – average transcription rate (not part of SSPCM framework, original description from[2]) [nts/sec].
k_{rp} – the apparent working rate of RNA polymerase complex (*approximate*) [molecules (nt) s^{-1} rp^{-1}].
k_{rs} – the apparent working rate of ribosome (translation) (*approximate*) [molecules (aa) s^{-1} rs^{-1}].
k_{stp} – the apparent working rate of transport protein (*generic*) [molecules (substrate) s^{-1} stp^{-1}].
k_T – translation efficiency of the ribosome (not part of SSPCM framework, original description from[4]) [unit not specified].
k_{trna} – combined (various processes) effective working rate of tRNA (*generic*) [molecules (aa) s^{-1} trna^{-1}].

lip – cell membrane lipid.
$LIP\%_{mmc}$ – total dry weight content of lipid macromolecular fraction in the cell [% (g (tot lip) (g (dw cell))$^{-1}$)].
lpe – membrane lipid synthesis enzyme.
l_{PW1} – number of reactions in central metabolic pathway (i.e. length of the pathway) PW_1 (*generic*) [reactions PW_1^{-1}].
l_{PW2} – number of reactions in amino acid synthesis pathway (i.e. length of the pathway) PW_2 (*generic* except for t_{d_rs+PW2} = 3600 s example in Supplementary Discussion 5.2) [reactions PW_2^{-1}].
l_{PW3} – number of reactions in deoxyribonucleotide synthesis pathway (i.e. length of the pathway) PW_3 (*generic*) [reactions PW_3^{-1}].
l_{PW4} – number of reactions in ribonucleotide synthesis pathway (i.e. length of the pathway) PW_4 (*generic*) [reactions PW_4^{-1}].
l_{PW5} – number of reactions in lipid synthesis pathway (i.e. length of the pathway) PW_5 (*generic*) [reactions PW_5^{-1}].
l_{PWi} – number of reactions in linear metabolic pathway (i.e. length of the pathway) PW_i [reactions PW_i^{-1}].
m_{aa} – mass of polymerized amino acid (monomer of proteins) molecule (*average*, see (Supplementary Discussion 3)) [g aa^{-1}].
mc – molecular composition.
m_{cell_comp} – mass of cell component molecule/complex [g (cell comp)$^{-1}$]. m_{cell_comp} is the general designation for any of the following specific masses of different cell component molecules/complexes: m_{rc}, m_{enz}, m_{mprot}, m_{lpe}, m_{rp}, m_{rpc}, m_{dna}, m_{rrna}, m_{trna}, $m_{mrna\_cell\_comp}$.
M_{cyt} – mass of cytoplasm (cell components and water in cytoplasm) of the cell [g cyt^{-1}].
m_{dna} – mass of genome (*approximate*) [g genome^{-1}].
m_{dnt} – mass of polymerized deoxyribonucleotide (monomer of DNA) molecule (*average*) [g dnt^{-1}].
mem – cell membrane.
m_{enz} – mass of a molecule of the enzyme of central and biosynthesis pathways (*generic*) [g enz^{-1}].
m_{etc} – mass of ETC complex (*approximate*) [g etc^{-1}].
m_{lip} – mass of membrane lipid molecule (*generic*) [g lip^{-1}].
m_{lpe} – mass of lipid synthesis enzyme molecule (*generic*) [g lpe^{-1}].
mmc – macromolecular composition.
M_{mem} – mass of cell membrane (cell components and water in cell membrane) [g mem^{-1}].
m_{mon} – mass of polymerized monomer molecule [g mon^{-1}]. m_{mon} is the general designation for any of the following specific masses of different polymerized monomer molecules: m_{nt}, m_{dnt}, m_{aa}.
m_{mprot} – mass of membrane protein complex [g (mprot)$^{-1}$]. m_{mprot} is the general designation for any of the following specific masses of different membrane protein complexes: m_{etc}, m_{stp}.
$m_{mrna\_cell\_comp}$ – mass of a molecule of mRNA of protein [g (mrna cell comp)$^{-1}$]. $m_{mrna\_cell\_comp}$ is the general designation for any of the following specific masses of mRNA molecules of different proteins: m_{mrna_rc}, m_{mrna_enz}, m_{mrna_etc}, m_{mrna_lpe}, m_{mrna_stp}, m_{mrna_rp}, m_{mrna_rpc}.
m_{mrna_enz} – mass of a molecule of mRNA of the enzyme of central and biosynthesis pathways (*generic*) [g (mrna enz)$^{-1}$].
m_{mrna_etc} – mass of a molecule of mRNA of ETC complex (*approximate*) [g (mrna etc)$^{-1}$].
m_{mrna_lpe} – mass of a molecule of mRNA of lipid synthesis enzyme (*generic*) [g (mrna lpe)$^{-1}$].
m_{mrna_rc} – mass of a molecule of mRNA of replisome complex (*approximate*) [g (mrna rc)$^{-1}$].
m_{mrna_rp} – mass of a molecule of mRNA of RNA polymerase complex (*approximate*) [g (mrna rp)$^{-1}$].

m_{mrna_rpc} – mass of a molecule of mRNA of ribosomal protein complex (*approximate*) [g $(\text{mrna rpc})^{-1}$].
m_{mrna_stp} – mass of a molecule of mRNA of transport protein (*approximate*) [g $(\text{mrna stp})^{-1}$].
m_{nt} – mass of polymerized ribonucleotide (monomer of RNA) molecule (*average*) [g nt^{-1}].
mon – monomer of biopolymer.
mprot – membrane protein (ETC, substrate transport protein).
m_{rc} – mass of replisome complex (*approximate*) [g rc^{-1}].
m_{rp} – mass of RNA polymerase complex (*approximate*) [g rp^{-1}].
m_{rpc} – mass of ribosomal protein complex (*approximate*) [g rpc^{-1}].
M_{rpc} – total mass of RPC in the cell [g (tot rpc) $cell^{-1}$].
m_{rrna} – mass of assembled rRNA complex (*approximate*) [g $rrna^{-1}$].
M_{rrna} – total mass of assembled rRNA complexes in the cell [g (tot rrna) $cell^{-1}$].
m_{stp} – mass of transport protein complex (*approximate*) [g stp^{-1}].
M_{tot} – cell mass (cell components and water in the cell) [g $cell^{-1}$].
m_{trna} – mass of tRNA molecule (*approximate*) [g $trna^{-1}$].
M_{trna} – total mass of tRNA molecules in the cell [g (tot trna) $cell^{-1}$].
Mw_{aa} – molar mass of polymerized amino acid (*average*) [g $(\text{mol (aa)})^{-1}$].
Mw_{dnt} – molar mass of polymerized deoxyribonucleotide (*average*) [g $(\text{mol (dnt)})^{-1}$].
Mw_{lip} – molar mass of membrane lipid (*generic*) [g $(\text{mol (lip)})^{-1}$].
Mw_{mon} – molar mass of polymerized monomer [g $(\text{mol (mon)})^{-1}$]. Mw_{mon} is the general designation for any of the following specific molar masses of different polymerized monomers: Mw_{aa}, Mw_{dnt}, Mw_{nt}.
Mw_{nt} – molar mass of polymerized ribonucleotide (*average*) [g $(\text{mol (nt)})^{-1}$].
N_A – Avogadro constant (*specific*, *precise*, see (Supplementary Discussion 3)) [molecules mol^{-1}].
$N_{aa\_enz\_PW2}$ – number of molecules of polymerized amino acid of the enzyme of amino acid biosynthesis pathway PW_2 in the cell [molecules (aa enz PW_2) $cell^{-1}$].
N_{aa_prot} – number of molecules of polymerized amino acid of unspecified protein in the cell [molecules (aa prot) $cell^{-1}$].
$N_{aa\_rpc+enz\_PW2}$ – the combined number of molecules of polymerized amino acid of RPC and enzyme of amino acid biosynthesis pathway PW_2 in the cell [molecules (aa enz PW_2 rpc) $cell^{-1}$].
$N_{aa\_rpc+enz\_PW2+prot}$ – total number of molecules of polymerized amino acid in the proto-cell of SSPCM-RS+AA+PROT [molecules (tot aa) $cell^{-1}$].
N_{cell_comp} – number of cell component molecules/complexes in the cell [molecules (cell comp) $cell^{-1}$]. N_{cell_comp} is the general designation for any of the specific numbers of different cell component molecules including N_{pol}, N_{rc}, $N_{enz\_PWi\_r}$, N_{enz_PW2}, N_{etc}, N_{stp}, N_{dna}, N_{rrna}, N_{trna}, $N_{mrna\_cell\_comp}$, N_{lip}, N_{aa_prot}, $N_{aa\_enz\_PW2}$, $N_{aa\_rpc+enz\_PW2}$, $N_{aa\_rpc+enz\_PW2+prot}$.
n_{cell_comp} – number of monomer molecules in (i.e. length of monomer sequence of) the macromolecular cell component molecule/complex [molecules (mon) $(\text{cell comp})^{-1}$]. n_{cell_comp} is the general designation for any of the following specific numbers of monomer molecules in different cell component molecules/complexes: n_{rc}, n_{enz}, n_{etc}, n_{lpe}, n_{stp}, n_{rp}, n_{rpc}, n_{dna}, n_{rrna}, n_{trna}, $n_{mrna\_cell\_comp}$.
$N_{cell\_comp\_cat}$ – number of catalysing cell component molecules/complexes in the cell [molecules (cell comp) $cell^{-1}$]. $N_{cell\_comp\_cat}$ is the general designation for any of the specific numbers of molecules of different catalysing cell components including $N_{enz\_PWi\_r}$, N_{rs}.
n_{dna} – number of deoxyribonucleotide molecules in (i.e. length of deoxyribonucleotide sequence of) the genome (*specific*, *precise*) [molecules (dnt) $genome^{-1}$] or [molecules (bp) $genome^{-1}$].

N_{dna} – number of genomes (genome copy number) in the cell (*approximate*) [molecules (genome) cell^{-1}]. The parameter was originally designated as G in previous works[5] but the designation was changed in the current work for clarification purposes (N is the usual designation of numbers of molecules in the literature).

n_{enz} – number of amino acid molecules in (i.e. length of amino acid sequence of) the molecule of the enzyme catalysing reactions of central pathway and biosynthesis pathways PW_1 – PW_5 (*generic*) [molecules (aa) enz^{-1}].

$N_{enz\_PW1\_r}$ – number of molecules of the enzyme catalysing a single reaction r of central metabolic pathway PW_1 [molecules (enz PW_1) cell^{-1} reaction^{-1}].

N_{enz_PW2} – number of molecules of the enzyme of amino acid biosynthesis pathway PW_2 in the cell [molecules (enz PW_2) cell^{-1}].

$N_{enz\_PW2\_r}$ – number of molecules of the enzyme catalysing a single reaction r of amino acid biosynthesis pathway PW_2 [molecules (enz PW_2) cell^{-1} reaction^{-1}].

$N_{enz\_PW3\_r}$ – number of molecules of the enzyme catalysing a single reaction r of deoxyribonucleotide biosynthesis pathway PW_3 [molecules (enz PW_3) cell^{-1} reaction^{-1}].

$N_{enz\_PW4\_r}$ – number of molecules of the enzyme catalysing a single reaction r of ribonucleotide biosynthesis pathway PW_4 [molecules (enz PW_4) cell^{-1} reaction^{-1}].

$N_{enz\_PW5\_r}$ – number of molecules of the enzyme catalysing a single reaction r of lipid biosynthesis pathway PW_5 [molecules (enz PW_5) cell^{-1} reaction^{-1}].

$N_{enz\_PWi\_r}$ – number of molecules of the enzyme catalysing a single reaction r of pathway PW_i [molecules (enz PW_i) cell^{-1} reaction^{-1}].

n_{etc} – number of amino acid molecules in (i.e. length of amino acid sequence of) the ETC complex (*approximate*) [molecules (aa) etc^{-1}].

N_{etc} – number of ETC complexes in the cell [molecules (etc) cell^{-1}].

N_{lip} – number of cell membrane lipid molecules in the cell [molecules (lip) cell^{-1}].

n_{lpe} – number of amino acid molecules in (i.e. length of amino acid sequence of) the lipid synthesis enzyme molecule (*generic*) [molecules (aa) lpe^{-1}].

N_{lpe} – number of lipid synthesis enzyme molecules in the cell [molecules (lpe) cell^{-1}].

n_{mrna} – number of molecules of ribonucleotides in (i.e. length of ribonucleotide sequence of) the unspecified mRNA molecule (*generic*) [molecules (nt) mrna^{-1}].

$N_{mrna\_cell\_comp}$ – number of molecules of mRNA of protein in the cell [molecules (mrna prot) cell^{-1}]. $N_{mrna\_cell\_comp}$ is the general designation for any of the following specific numbers of mRNA molecules of different proteins: N_{mrna_rc}, N_{mrna_enz}, N_{mrna_etc}, N_{mrna_lpe}, N_{mrna_stp}, N_{mrna_rp}, N_{mrna_rpc}.

$n_{mrna\_cell\_comp}$ – number of ribonucleotide molecules in (i.e. length of ribonucleotide sequence of) the molecule of mRNA of protein [molecules (nt) (mrna prot)$^{-1}$]. $n_{mrna\_cell\_comp}$ is the general designation for any of the following specific numbers of ribonucleotide molecules in mRNA molecule of different proteins: n_{mrna_rc}, n_{mrna_enz}, n_{mrna_etc}, n_{mrna_lpe}, n_{mrna_stp}, n_{mrna_rp}, n_{mrna_rpc}.

N_{mrna_enz} – number of molecules of mRNA of the enzyme of central and biosynthesis pathways in the cell [molecules (mrna enz) cell^{-1}].

n_{mrna_enz} – number of ribonucleotide molecules in (i.e. length of ribonucleotide sequence of) the molecule of mRNA of the enzyme of central and biosynthesis pathways (*generic*) [molecules (nt) (mrna enz)$^{-1}$].

N_{mrna_etc} – number of molecules of mRNA of ETC complex in the cell [molecules (mrna etc) cell^{-1}].

n_{mrna_etc} – number of ribonucleotide molecules in (i.e. length of ribonucleotide sequence of) the molecule of mRNA of ETC complex (*approximate*) [molecules (nt) (mrna etc)$^{-1}$].

N_{mrna_lpe} – number of molecules of mRNA of lipid synthesis enzyme in the cell [molecules (mrna lpe) cell^{-1}].

n_{mrna_lpe} – number of ribonucleotide molecules in (i.e. length of ribonucleotide sequence of) the molecule of mRNA of lipid synthesis enzyme (*generic*) [molecules (nt) (mrna lpe)$^{-1}$].
N_{mrna_rc} – number of molecules of mRNA of replisome complex in the cell [molecules (mrna rc) cell^{-1}].
n_{mrna_rc} – number of ribonucleotide molecules in (i.e. length of ribonucleotide sequence of) the molecule of mRNA of replisome complex (*approximate*) [molecules (nt) (mrna rc)$^{-1}$].
N_{mrna_rp} – number of molecules of mRNA of RNA polymerase complex in the cell [molecules (mrna rp) cell^{-1}].
n_{mrna_rp} – number of ribonucleotide molecules in (i.e. length of ribonucleotide sequence of) the molecule of mRNA of RNA polymerase complex (*approximate*) [molecules (nt) (mrna rp)$^{-1}$].
N_{mrna_rpc} – number of molecules of mRNA of ribosomal protein complex in the cell [molecules (mrna rpc) cell^{-1}].
n_{mrna_rpc} – number of ribonucleotide molecules in (i.e. length of ribonucleotide sequence of) the molecule of mRNA of ribosomal protein complex (*approximate*) [molecules (nt) (mrna rpc)$^{-1}$].
N_{mrna_stp} – number of molecules of mRNA of transport protein in the cell [molecules (mrna stp) cell^{-1}].
n_{mrna_stp} – number of ribonucleotide molecules in (i.e. length of ribonucleotide sequence of) the molecule of mRNA of transport protein (*approximate*) [molecules (nt) (mrna stp)$^{-1}$].
N_{pol} – number of molecules of polymerase in the cell [molecules (pol) cell^{-1}]. N_{pol} is the general designation for any of the following specific numbers of molecules of different polymerases: N_{rce}, N_{lpe}, N_{rp}, N_{rs}, $N_{rs\_cell\_comp}$.
N^R – the number of amino acids of a functional ribosome (not part of SSPCM framework, original description from[6]) [unit not specified].
n_{rc} – number of amino acid molecules in (i.e. length of amino acid sequence of) the replisome complex (*approximate*) [molecules (aa) rc^{-1}].
N_{rc} – number of replisome complexes (corresponds to the replisomes that are necessary for the periodical replication process during t_C) in the cell (*approximate*) [molecules (rc) cell^{-1}].
N_{rce} – number of effective replisome complexes (corresponds to the replisomes that are necessary for the replication process that is averaged over the whole t_{d_srs} (takes place continuously)) in the cell (*approximate* for faster growth regions of SSPCM-RS+DNA and SSPCM-SRS-R if $t_{d\_srs} < t_C$ = 2319.84 s (Supplementary Table 24)) [molecules (rce) cell^{-1}].
N_{ribo} – the total number of ribosomes in the cell (not part of SSPCM framework, original description from[2]) [unit not specified].
N_{RNAP} – the number of RNAPs in the cell (not part of SSPCM framework, original description from[2]) [unit not specified].
n_{rp} – number of amino acid molecules in (i.e. length of amino acid sequence of) the RNA polymerase complex (*approximate*) [molecules (aa) rp^{-1}].
N_{rp} – number of RNA polymerase complexes in the cell [molecules (rp) cell^{-1}].
n_{rpc} – number of amino acid molecules in (i.e. length of amino acid sequence of) the ribosomal protein complex (*approximate*) [molecules (aa) rpc^{-1}].
N_{rrna} – number of assembled rRNA complexes in the cell [molecules (rrna) cell^{-1}].
n_{rrna} – number of ribonucleotide molecules in (i.e. length of ribonucleotide sequence of) the assembled rRNA complex (*specific, precise*) [molecules (nt) rrna^{-1}].
N_{rs} – number of ribosomes (equal for ribosomal protein complexes) in the cell [molecules (rs) cell^{-1}].
$N_{rs\_cell\_comp}$ – number of ribosomes for the synthesis of protein in the cell [molecules (rs prot) cell^{-1}]. $N_{rs\_cell\_comp}$ is the general designation for any of the following specific numbers of ribosomes for the synthesis of different proteins: N_{rs_rc}, N_{rs_enz}, N_{rs_etc}, N_{rs_lpe}, N_{rs_stp}, N_{rs_rp}, N_{rs_rpc}.

N_{rs_enz} – number of ribosomes for the synthesis of enzymes in central and biosynthesis pathways [molecules (rs enz) cell^{-1}].
N_{rs_etc} – number of ribosomes for the synthesis of ETC complexes [molecules (rs etc) cell^{-1}].
N_{rs_lpe} – number of ribosomes for the synthesis of lipid synthesis enzymes [molecules (rs lpe) cell^{-1}].
N_{rs_rc} – number of ribosomes for the synthesis of replisome complexes [molecules (rs rc) cell^{-1}].
N_{rs_rp} – number of ribosomes for the synthesis of RNA polymerase complexes [molecules (rs rp) cell^{-1}].
N_{rs_rpc} – number of ribosomes for the synthesis of ribosomal proteins [molecules (rs rpc) cell^{-1}].
N_{rs_stp} – number of ribosomes for the synthesis of transport proteins [molecules (rs stp) cell^{-1}].
n_{stp} – number of amino acid molecules in (i.e. length of amino acid sequence of) the transport protein complex (*approximate*) [molecules (aa) stp^{-1}].
N_{stp} – number of transport protein complexes in the cell [molecules (stp) cell^{-1}].
nt – ribonucleotide (monomer of RNA).
n_{trna} – number of ribonucleotide molecules in (i.e. length of ribonucleotide sequence of) the tRNA molecule (*average*) [molecules (nt) trna^{-1}].
N_{trna} – number of tRNA molecules in the cell [molecules (trna) cell^{-1}].
P_{cell_comp} – polysome density of mRNA of protein [molecules (nt (covered by rs)) (molecules (nt (covered by tot rs)))$^{-1}$]. P_{cell_comp} is the general designation for any of the following specific polysome densities of mRNAs of different proteins: P_{rc}, P_{enz}, P_{etc}, P_{lpe}, P_{stp}, P_{rp}, P_{rpc}.
$P_{cell\_comp\_min}$ – minimal (single ribosome) polysome density of mRNA of protein [molecules (nt (covered by rs)) (molecules (nt (mrna prot)$^{-1}$))$^{-1}$]. $P_{cell\_comp\_min}$ is the general designation for any of the following specific minimal polysome densities of mRNAs of different proteins: P_{rc_min}, P_{enz_min}, P_{etc_min}, P_{lpe_min}, P_{stp_min}, P_{rp_min}, P_{rpc_min}.
P_{enz} – polysome density of mRNA of the enzyme of central and biosynthesis pathways (*generic*) [molecules (nt (covered by rs)) (molecules (nt (covered by tot rs)))$^{-1}$].
P_{enz_min} – minimum polysome density of mRNA of the enzyme of central and biosynthesis pathways [molecules (nt (covered by rs)) (molecules (nt (mrna enz)$^{-1}$))$^{-1}$].
P_{etc} – polysome density of mRNA of ETC complex (*generic*) [molecules (nt (covered by rs)) (molecules (nt (covered by tot rs)))$^{-1}$].
P_{etc_min} – minimum polysome density of mRNA of ETC complex [molecules (nt (covered by rs)) (molecules (nt (mrna etc)$^{-1}$))$^{-1}$].
P_{lpe} – polysome density of mRNA of lipid synthesis enzyme (*generic*) [molecules (nt (covered by rs)) (molecules (nt (covered by tot rs)))$^{-1}$].
P_{lpe_min} – minimum polysome density of mRNA of lipid synthesis enzyme [molecules (nt (covered by rs)) (molecules (nt (mrna lpe)$^{-1}$))$^{-1}$].
P_{mrna} – polysome density of unspecified mRNA (*generic*) [molecules (nt (covered by rs)) (molecules (nt (covered by tot rs)))$^{-1}$].
pol – polymerase complexes (RC, RP, ribosome, LPE).
P_{rc} – polysome density of mRNA of replisome complex (*generic*) [molecules (nt (covered by rs)) (molecules (nt (covered by tot rs)))$^{-1}$].
P_{rc_min} – minimum polysome density of mRNA of replisome complex [molecules (nt (covered by rs)) (molecules (nt (mrna rc)$^{-1}$))$^{-1}$].
prot – protein (replisome complex, enzyme of metabolic pathways, electron transport chain, lipid synthesis enzyme, transport protein, RNA polymerase complex, ribosome protein complex, unspecified).
P_{rp} – polysome density of mRNA of RNA polymerase complex (*generic*) [molecules (nt (covered by rs)) (molecules (nt (covered by tot rs)))$^{-1}$].

P_{rp_min} – minimum polysome density of mRNA of RNA polymerase complex [molecules (nt (covered by rs)) (molecules (nt (mrna rp)$^{-1}$))$^{-1}$].
P_{rpc} – polysome density of mRNA of ribosomal protein complex (*generic*) [molecules (nt (covered by rs)) (molecules (nt (covered by tot rs)))$^{-1}$].
P_{rpc_min} – minimum polysome density of mRNA of ribosomal protein complex [molecules (nt (covered by rs)) (molecules (nt (mrna rpc)$^{-1}$))$^{-1}$].
P_{stp} – polysome density of mRNA of transport protein (*generic*) [molecules (nt (covered by rs)) (molecules (nt (covered by tot rs)))$^{-1}$].
P_{stp_min} – minimum polysome density of mRNA of transport protein [molecules (nt (covered by rs)) (molecules (nt (mrna stp)$^{-1}$))$^{-1}$].
PW_1 – central metabolic pathway consisting of a linear chain of reactions (synthesis of building blocks from substrate).
PW_2 – amino acid synthesis pathway consisting of a linear chain of reactions (synthesis of amino acids from building blocks).
PW_3 – deoxyribonucleotide synthesis pathway consisting of a linear chain of reactions (synthesis of deoxyribonucleotides from building blocks).
PW_4 – ribonucleotide synthesis pathway consisting of a linear chain of reactions (synthesis of ribonucleotides from building blocks).
PW_5 – lipid synthesis pathway consisting of a linear chain of reactions (synthesis of lipids from building blocks).
PW_i – metabolic pathway i consisting of a linear chain of reactions.
rc – replisome complex. Corresponds to the replisomes that are necessary for the periodical replication process.
rce – effective replisome complex. Corresponds to the replisomes that are necessary for the replication process that is averaged over t_{d_srs} (takes place continuously).
rp – RNA polymerase complex.
rpc – ribosomal protein complex.
rs – ribosome.
$RS\%_{mc}$ – total dry weight content of ribosomal molecular fraction in the cell [% (g (tot rs) (g (dw cell))$^{-1}$)].
R_{tot} – cell radius [cm cell^{-1}].
s_{cell_comp} – the cell membrane surface area occupied by a single cell component molecule/complex [cm^2 (cell comp)$^{-1}$]. s_{cell_comp} is the general designation for any of the following specific cell surface areas occupied by different cell component molecules/complexes: s_{mprot}, s_{lip}.
S_{cell_comp} – the total cell membrane surface area occupied by cell component molecules/complexes [cm^2 (tot cell comp)$^{-1}$]. S_{cell_comp} is the general designation for any of the following specific total cell surface areas occupied by different cell component molecules/complexes: S_{etc}, S_{lip}, S_{stp}.
s_{etc} – the cell membrane surface area occupied by a single ETC complex (*approximate*) [cm^2 etc^{-1}].
S_{etc} – the total cell membrane surface area covered by ETC complexes [cm^2 (tot etc)$^{-1}$].
s_{lip} – the cell membrane surface area occupied by a single membrane lipid (*generic*) [cm^2 lip^{-1}].
S_{lip} – the total cell membrane surface area covered by membrane lipids [cm^2 (tot lip)$^{-1}$].
s_{mprot} – the cell membrane surface area occupied by a single membrane protein complex [cm^2 (mprot)$^{-1}$]. s_{mprot} is the general designation for any of the following specific cell surface areas occupied by different membrane protein complexes: s_{etc}, s_{stp}.
srs – self-replication system.

s_{stp} – the cell membrane surface area occupied by a single transport protein (*approximate*) [cm^2 stp^{-1}].
S_{stp} – the total cell membrane surface area covered by transport proteins [cm^2 (tot $stp)^{-1}$].
S_{tot} – the surface area of the cell (membrane) [cm^2 $cell^{-1}$].
stp – substrate transport protein.
t_C – genome replication time (the period between DNA replication initiation and termination of a single genome) (*approximate* except for faster growth regions of SSPCM-RS+DNA and SSPCM-SRS-R if t_{d_srs} < 2319.84 s) [s] or [s $genome^{-1}$]. The parameter was originally designated as *C* in previous works (for example[7]) but the designation was changed in the current work for clarification purposes (*t* is an established designation for time in the literature).
t_{cell_comp} – time coefficient of cell component [s]. t_{cell_comp} is the general designation for any of the following specific time coefficients of different cell components: t_{PW2}, t_{PW4}, t_{rp}, t_{trna}, t_{d_rs}, t_{lpe}.
t_d – cell cycle duration or length (doubling time), cell age at the end of the cell cycle [s]. The parameter was originally designated as τ in previous works (for example[7]) but the designation was changed in the current work for clarification purposes (*t* is an established designation for time in the literature).
$t_{d\_cell\_comp}$ – doubling time of cell component molecule/complex [s]. $t_{d\_cell\_comp}$ is the general designation for any of the following specific doubling times of different cell components: t_{d_PW4}, t_{d_rp}, t_{d_rrna}, t_{d_rs}, t_{d_trna}, t_{d_mrna}, $t_{d\_mrna\_enz}$, $t_{d\_mrna\_rp}$, $t_{d\_mrna\_rpc}$, $t_{d\_rs+rrna}$.
t_{d_min} – minimal cell cycle duration or length (cell cycle length on the growth boundary) [s].
t_{d_mrna} – relative doubling time of a single unspecified mRNA molecule [s].
$t_{d\_mrna\_enz}$ – relative doubling time of a single molecule of mRNA of the enzyme of biosynthesis pathways [s].
$t_{d\_mrna\_rp}$ – relative doubling time of a single molecule of mRNA of RP complex [s].
$t_{d\_mrna\_rpc}$ – relative doubling time of a single molecule of mRNA of ribosomal protein complex [s].
t_{d_PW4} – relative doubling time of ribonucleotide biosynthesis pathway PW_4 [s].
t_{d_rp} – doubling time of a single RP complex (*approximate*) [s] or [s rp^{-1}].
t_{d_rrna} – doubling time of a single assembled rRNA complex [s] or [s $rrna^{-1}$].
t_{d_rs} – doubling time of SSPCM-RS or doubling time of a single RPC (*approximate*) [s] or [s rpc^{-1}].
t_{d_rs+dna} – doubling time of SSPCM-RS+DNA [s].
t_{d_rs+lip} – doubling time of SSPCM-RS+LIP [s].
t_{d_rs+PW2} – doubling time of SSPCM-RS+AA [s].
$t_{d\_rs+PW2+prot}$ – doubling time of SSPCM-RS+AA+PROT [s].
$t_{d\_rs+PW2+rna}$ – doubling time of SSPCM-RS+AA+RNA [s].
$t_{d\_rs+PW2+rna+lip}$ – doubling time of SSPCM-RS+AA+RNA+LIP [s].
$t_{d\_rs+PW2+rna+lip+mprot}$ – doubling time of SSPCM-RS+AA+RNA+LIP+MPROT [s].
$t_{d\_rs+rrna}$ – doubling time of a single ribosome (RPC and assembled rRNA complex) [s].
t_{d_srs} – doubling time of the self-reproduction system of the abstract proto-cell [s]. t_{d_srs} is the general designation for any of the following specific doubling times of different self-reproduction systems: t_{d_srs-m}, t_{d_srs-r}, t_{d_rs}, t_{d_rs+PW2}, $t_{d\_rs+PW2+prot}$, $t_{d\_rs+PW2+rna}$, $t_{d\_rs+PW2+rna+lip}$, t_{d_rs+lip}, $t_{d\_rs+PW2+rna+lip+mprot}$, t_{d_rs+dna}.
t_{d_srs-m} – doubling time of SSPCM-SRS-M [s].
$t_{d\_srs-m\_min}$ – minimal doubling time or doubling time on the growth boundary of SSPCM-SRS-M [s].
t_{d_srs-r} – doubling time of SSPCM-SRS-R [s].

$t_{d\_srs\text{-}r\_min}$ – minimal doubling time or doubling time on the growth boundary of SSPCM-SRS-R [s].
t_{d_trna} – doubling time of tRNA [s] or [s $trna^{-1}$].
t_{lpe} – time coefficient of LPE (*generic*) [s] or [s molecules (aa) (molecules $(lip))^{-1}$].
tot – total. In the case of symbols, tot designates whole-cell parameters (for example M_{tot} as cell mass). In the case of unit descriptions, tot designates the total amount of all different (if available) necessary cell components that belong to the corresponding fraction (for example [g (tot rrna) $cell^{-1}$] as the mass of all rRNA complexes in the cell).
t_{PW2} – time coefficient of amino acid biosynthesis pathway PW_2 (*generic*) [s] or [s molecules (aa) (molecules (metabolite) $PW_2)^{-1}$].
t_{PW4} – time coefficient of ribonucleotide biosynthesis pathway PW_4 (*generic*) [s] or [s molecules (aa) (molecules (metabolite) $PW_4)^{-1}$].
t_{rp} – time coefficient of RP complex (*approximate*) [s] or [s molecules (aa) (molecules $(nt))^{-1}$].
t_{trna} – time coefficient of tRNA [s] or [s molecules (nt) (molecules $(aa))^{-1}$].
u_{rs} – length of mRNA sequence covered by the single ribosome during translation (*approximate*) [molecules (nt) rs^{-1}].
V_{cyt} – the volume of cytoplasmic space (cell components and water in cytoplasm) of the cell [cm^3 cyt^{-1}].
V_{tot} – cell volume (cell components and water in the cell) [cm^3 $cell^{-1}$].
X_{cell_comp} – energy cost of cell process per single cell component [molecules (atp) (cell $comp)^{-1}$]. X_{cell_comp} is the general designation for any of the following specific energy costs per single cell components: X_{dna}, X_{PWi}, X_{lip}, X_{prot}, X_{stp}, X_{rna}.
X_{dna} – energy cost of DNA replication (*approximate*) [molecules (atp) dnt^{-1}].
X_{lip} – energy cost of membrane lipid synthesis (*approximate*) [molecules (atp) lip^{-1}].
X_{prot} – energy cost of translation (*approximate*) [molecules (atp) aa^{-1}].
X_{PW2} – energy cost of amino acid synthesis (*approximate*) [molecules (atp) aa^{-1}].
X_{PW3} – energy cost of deoxyribonucleotide synthesis (*approximate*) [molecules (atp) dnt^{-1}].
X_{PW4} – energy cost of ribonucleotide synthesis (*approximate*) [molecules (atp) nt^{-1}].
X_{PW5} – energy cost of lipid synthesis (*generic*) [molecules (atp) $lipid^{-1}$].
X_{PWi} – energy cost of monomer synthesis in biosynthesis pathway PW_i [molecules (atp) mon^{-1}].
X_{rna} – energy cost of transcription (*approximate*) [molecules (atp) nt^{-1}].
X_{stp} – energy cost of substrate transport (*generic*) [molecules (atp) $substrate^{-1}$].
α – the rate of synthesis of ribosomal protein per rate of synthesis of total protein (not part of SSPCM framework, original description from[1]) [unit not specified].
γ – inverse time to translate a ribosome (not part of SSPCM framework, original description from[8]) [h^{-1}].
γ – translation rate (not part of SSPCM framework, original description from[6]) [unit not specified].
γ' – effective translation rate of a functional ribosome (not part of SSPCM framework, original description from[6]) [unit not specified].
η – protein degradation rate (not part of SSPCM framework, original description from[8]) [h^{-1}].
μ – specific growth rate of the cell culture [h^{-1}].
μ_2 – growth rate of the cell culture (not part of SSPCM framework, original description from[9]) [doubling h^{-1}].
μ_{max} – maximal specific growth rate of the cell culture (corresponds to t_{d_min} of a single cell) [h^{-1}].
ρ_{mprot} – density of membrane proteins (transporter complex, electron transport chain complex) (*generic*) [g (mprot) (cm^3 $(mprot))^{-1}$].
ρ_{tot} – cell density (*generic*) [g (cell) (cm^3 $(cell))^{-1}$].

τ_{rRNA} – the time an RNAP takes to synthesize a full set of rRNA (not part of SSPCM framework, original description from[2]) [min].

Supplementary Discussion 3: List of terms

The following list of terms is used to characterize cellular entities (cell components, cellular processes, cellular interactions) and their properties (values of parameters) in developed SSPCMs.

Approximate – corresponds to entities and properties that belong to the usual range of varying values of *E. coli* cells. The sources of variations in values include different *E. coli* K12 strains (growth independent physiological parameters, part of the input parameter values of used models), effects of cell growth and changes in growth environment (growth dependent physiological parameters, part of the input and output parameter values of used models). The range of values is only possible due to the lack of information about *E. coli* K12 MG1655, not well described (exact) effects of cell growth and changes in the growth environment, simplifications made in the models. The values with this term comprised part of the input parameter values of used models.
Average – corresponds to the arithmetic mean of entities and properties of *E. coli* K12 MG1655. Again, the term is involved for the same parameter types and used models as mentioned before. Average enables to reduce the complexity (number of components, relations and parameters) of the models but maintains principal relations between cell components.
Generic – corresponds to entities and properties that are not related to any certain strain of *E. coli* K12 but are still generally acceptable and correspond to the known biological range.
Specific, precise – corresponds to entities and properties of *E. coli* K12 MG1655. The term is usually utilized for growth independent physiological parameters and part of the input parameter values of used models.

Supplementary Discussion 4: List of short definitions

Complexity of SRS – the total number of species of all relevant cell components and respective cell processes belonging to the synthesis equipment or SRS. The complexity of SRS is independent of proto-cell growth in SSPCMs. Indirectly, this term characterizes the size of the metabolic network (number of reactions) and growth media type. Rich growth medium (includes monomers of macromolecules) corresponds to shorter SRS while minimal growth medium requires longer SRS.
Proto-cell – cell (model) that is consisting of only SRS (essential cell components necessary for the self-reproduction of the cell) and lacking all other cell components and processes which can be found in living cells. Note that the term proto-cell has been used relatively loosely in the literature to refer to primitive cells or the first cells[10].
Self-reproduction system (SRS) – set of essential cell components with functions (including polymerisation of macromolecules and synthesis of respective monomers/metabolites, energy synthesis, substrate transport, cell membrane formation) directly necessary for cell growth and self-reproduction at given growth conditions. Cell components and processes that are not directly necessary for self-reproduction (like regulation) do not belong to SRS including those

cell components that are necessary for self-reproduction but at different growth conditions (like metabolic pathways for different media). In the current work, only simplified (incomplete) SRSs consisting of different combinations of essential cell components were described by respective SSPCMs. It should be stressed that SRS does not correspond to homonymic computer science[11], vesicle formation[12], viral protein assemblies[13] and complexity theory[14] terms.

Size of SRS – the total number of molecules/complexes or the total mass of all relevant cell components belonging to the synthesis equipment or SRS. The size of SRS is dependent on proto-cell growth in SSPCMs. Size generally increases during faster growth because there is less time to finish all the required cellular processes and, therefore, the numbers of catalysts increase to compensate for the lack of time. Note that not all sizes of subparts of SRS increase monotonously during faster growth (lipid synthesis depends on the change of surface/volume change and available free space on the cell membrane, DNA replication is required only for a single genome).

Supplementary Discussion 5

Supplementary Discussion 5.1: Derivations, analysis and calculations based on SSPCM-RS

Numerous formulas have been derived previously describing ribosome self-replication. The corresponding formula for an exponentially growing cell population can be, for example, derived from Supplementary Eq. (79) [1] assuming that α = 1. More specifically, it has been formulated, for example, according to Supplementary Eq. (1) [6]:

$$\frac{\log(2)}{\gamma'} = \frac{\log(2) \cdot N^R}{\gamma} \qquad (1)$$

Also, the following constraint (Supplementary Eq. (2)) has been defined[4]:

$$\mu \le k_T \qquad (2)$$

Although k_T is not clearly defined, it can be assumed that it is identical to γ' in Supplementary Eq. (1). Growth boundary that is similar to Supplementary Eq. (2) (protein degradation included) has been termed ribosome catastrophe in metabolic scaling study of diverse bacteria based on various cellular parameters[15] and this term designates the condition of the cell completely filled by ribosomes (equation S24). The latter boundary, however, has been observed only in the case of large cells.

In the current work, the content of previous formulas was rewritten based on the model named SSPCM-RS (Supplementary Figure 1, Supplementary Discussion 5.11.3) and using our own original notations for clarity. The minimal theoretical limit (t_{d_rs}) is expressed by Supplementary Eq. (3):

$$t_{d\_rs} = \frac{N_{rs} \cdot n_{rpc}}{N_{rs} \cdot k_{rs}} = \frac{n_{rpc}}{k_{rs}} \qquad (3)$$

Note that the Supplementary Eq. (3) of SSPCM-RS (Supplementary Figure 1, Supplementary Discussion 5.11.3) is very similar to Supplementary Eq. (1). N^R is identical to the n_{rpc} in the current work. Although γ has not been clearly defined, we can phenomenologically assume that it is identical to k_{rs} in the current work. It appears that Supplementary Eq. (3) and Supplementary Eq. (1) differ only by the factor ln 2 as the exponential growth of cell population was assumed[6] and linear growth of a single cell in the current work.

If the *approximate* (terms characterizing parameter values are explained in Supplementary Discussion 3) n_{rpc} = 7242 molecules (aa) rpc^{-1} as in *E. coli* and the *approximate* k_{rs} = 20 molecules (aa) s^{-1} rs^{-1} (Supplementary Table 4, Supplementary Table 10), then the *approximate* t_{d_rs} = 362.1 s or s rpc^{-1}, or approximately 6 minutes (Table 1, Supplementary Table 28). The result is comparable to other estimations of 7-8 minutes[1,6,16-18]. This means that the (absolute) limit of the maximal specific growth rate of the cell culture is 7 h^{-1} if based on the characteristics of the ribosomes of *E. coli*. Certainly, current calculations were extremely simplified assuming ideal conditions (e.g. amino acids provided, assembly of rRNA and RPC not counted, diffusion limitations not counted). According to the literature, when considering also translational cofactors, the value of t_{d_rs} increases to approximately 9 minutes[19], and if promoter activity values for ribosomal proteins are experimentally determined for *E. coli* cells in various medium compositions[20] and these values then extrapolated to the extreme boundary (all promoter activity devoted to ribosomal proteins), then the value of μ = 2.9 h^{-1} or t_{d_rs} = 14 minutes[15].
It is also possible to conclude from Supplementary Eq. (3) that t_{d_rs} is not dependent on N_{rs} (Figure 1, Supplementary Table 28) – its value is determined by the ratio of n_{rpc} to k_{rs}. In other words, the doubling time of such a proto-cell is equal to the self-reproduction time of a single RPC[2]. The value of t_{d_rs} would change if we changed the values of the ribosome characteristics n_{rpc} and k_{rs}.

Supplementary Discussion 5.2: Derivations, analysis and calculations based on SSPCM-RS+AA

The next proto-cell is described by SSPCM-RS+AA (Supplementary Figure 2, Supplementary Discussion 5.11.4). Compared to the previous SSPCM-RS, the SRS here contains, in addition to the RPC, also a single amino acid synthesis pathway PW_2 consisting of a linear chain of reactions (synthesis of amino acids from building blocks) catalysed by enzymes. In this case, the RPC must also synthesize enzymes responsible for the synthesis of amino acids of a single type during t_{d_rs+PW2} according to Supplementary Eq. (4) (by combining Supplementary Eq. (80) and Supplementary Eq. (128)) describing the translation of the proto-cell:

$$t_{d\_rs+PW2} = \frac{N_{rs} \cdot n_{rpc} + N_{enz\_PW2\_r} \cdot n_{enz} \cdot l_{PW2}}{N_{rs} \cdot k_{rs}} \tag{4}$$

Note that a very similar formula describing the translation of ribosomal proteins and metabolic proteins has been derived earlier (S33 [15]). Flux through the amino acid synthesis pathway PW_2 equals the total translation flux according to Supplementary Eq. (73). $N_{enz\_PW2\_r}$ does not need to be equal to N_{rs}, and the ratio is determined by the values of k_{rs} and k_{enz}, whose values in the models are *approximate* 20 molecules (aa) s^{-1} rs^{-1} and *generic* 100 molecules (metabolite) s^{-1} enz^{-1}, respectively (Supplementary Table 4). The alternative form of t_{d_rs+PW2} is expressed by Supplementary Eq. (5) after making the necessary substitution of $N_{enz\_PW2\_r}$ from Supplementary Eq. (73):

$$t_{d\_rs+PW2} = \frac{N_{rs} \cdot n_{rpc} + N_{rs} \cdot \frac{k_{rs}}{k_{enz}} \cdot n_{enz} \cdot l_{PW2}}{N_{rs} \cdot k_{rs}} = \frac{n_{rpc}}{k_{rs}} + \frac{n_{enz} \cdot l_{PW2}}{k_{enz}} \tag{5}$$

This shows that N_{rs} can be removed altogether, which means that t_{d_rs+PW2} does not depend on N_{rs} and $N_{enz\_PW2\_r}$ in this case – it is determined by the properties of the macromolecules and by l_{PW2}, and it is the same for the SRS containing one or 10^4 molecules (rs) cell^{-1} or even more – see also Figure 1 and Supplementary Table 29. One important conclusion from the derivation of Supplementary Eq. (5) for all subsequent proto-cells is that the value of t_{d_srs} would be independent of N_{cell_comp} if those N_{cell_comp} of respective cell components are

stoichiometrically fixed to N_{rs}. In the case of SSPCM-RS+AA this is determined by the flux balance (Supplementary Eq. (73)).

It must be stressed that t_{d_rs+PW2} in the second part of Supplementary Eq. (5) is equal to the sum of two different expressions. The first part is again equal to t_{d_rs}, representing the self-reproduction of RPC. The second part corresponds to the time needed to synthesize enzymes of pathway PW_2. We can define this time (the time coefficient of amino acid biosynthesis pathway PW_2) as t_{PW2}:

$$t_{PW2} = \frac{n_{enz} \cdot l_{PW2}}{k_{enz}} \tag{6}$$

It must be stressed that t_{PW2} is not explicitly defined as the replication time (ratio of the size of the cell component to the apparent working rate of the polymerase) but the time coefficient (ratio of size to apparent working rate of the cell component) of pathway PW_2. Nevertheless, it is possible to deduce from the first part of Supplementary Eq. (5) that Supplementary Eq. (6) describes the doubling of enzymes of pathway PW_2, although k_{rs} from the denominator is cancelled out.

Considering Supplementary Eqs. (3) and (6), t_{d_rs+PW2} can be expressed as a sum:

$$t_{d\_rs+PW2} = t_{d\_rs} + t_{PW2} \tag{7}$$

If different (all 20) amino acids would be synthesized (instead of one general amino acid) by different parallel pathways, then the doubling time of the respective proto-cell would be the sum of all time coefficients of individual amino acid synthesis pathways and t_{d_rs}.

Note that the ratios of t_{d_rs+PW2}, t_{d_rs} and t_{PW2} are proportional to the ratios of different protein fractions (described by Supplementary Eqs. (8)-(10)) by combining Supplementary Eqs. (3)-(7) above and Supplementary Eq. (117):

$$\frac{t_{d\_rs+PW2}}{t_{d\_rs}} = 1 + \frac{t_{PW2}}{t_{d\_rs}} = \frac{N_{rs} \cdot n_{rpc} + N_{enz\_PW2\_r} \cdot n_{enz} \cdot l_{PW2}}{N_{rs} \cdot n_{rpc}} = 1 + \frac{N_{enz\_PW2\_r} \cdot n_{enz} \cdot l_{PW2}}{N_{rs} \cdot n_{rpc}} \tag{8}$$

$$\frac{t_{PW2}}{t_{d\_rs}} = \frac{t_{d\_rs+PW2}}{t_{d\_rs}} - 1 = \frac{n_{enz} \cdot l_{PW2} \cdot k_{rs}}{n_{rpc} \cdot k_{enz}} = \frac{N_{enz\_PW2\_r} \cdot n_{enz} \cdot l_{PW2}}{N_{rs} \cdot n_{rpc}} \tag{9}$$

$$\frac{t_{d\_rs+PW2}}{t_{PW2}} = 1 + \frac{t_{d\_rs}}{t_{PW2}} = \frac{t_{d\_rs+PW2}}{t_{d\_rs+PW2} - t_{d\_rs}} = \frac{t_{d\_rs}}{t_{PW2}} + \frac{F_{enz\_PW2\_r}}{F_{rs}} = \frac{k_{enz}}{k_{rs}} \cdot \left(\frac{n_{rpc}}{n_{enz} \cdot l_{PW2}} + \frac{N_{enz\_PW2\_r}}{N_{rs}} \right) = \frac{N_{rs} \cdot n_{rpc}}{N_{enz\_PW2\_r} \cdot n_{enz} \cdot l_{PW2}} + 1 \tag{10}$$

Based on Supplementary Eq. (5) and the values of the corresponding input parameters (Supplementary Tables 4-5, 10), it is possible to calculate the value of t_{d_rs+PW2} (962.1 s) (Table 1, Supplementary Table 29). It appears that the addition of the amino acid synthesis pathway PW_2 increased the value of t_{d_srs} by 600 s.

The calculated value of t_{d_rs+PW2} is very low compared to the t_d values of living cells – t_d of "average" bacterial cells (*E. coli*) would typically be approximately t_d = 3600 s (μ = 0.7 h^{-1})[21]. If we assume that t_{d_rs+PW2} = 3600 s, the value of l_{PW2} needed to reach t_{d_rs+PW2} = 3600 s in the simple two-component SRS of SSPCM-RS+AA should be l_{PW2} = 1079.3 reactions PW_2^{-1}, according to Supplementary Eq. (5). Indeed, if *approximate* k_{rs} = 20 molecules (aa) s^{-1} rs^{-1} (Supplementary Table 4), every RPC can create $7.2 \cdot 10^4$ polypeptide bonds (molecules (aa enz PW_2 rpc) $cell^{-1}$) during t_{d_rs+PW2} according to Supplementary Eq. (11):

$$N_{aa\_rpc+enz\_PW2} = N_{rs} \cdot n_{rpc} + N_{enz\_PW2\_r} \cdot n_{enz} \cdot l_{PW2} = N_{rs} \cdot k_{rs} \cdot t_{d\_rs+PW2} \tag{11}$$

This allows an RPC to duplicate itself and polymerize additional $N_{aa\_enz\_PW2}$ = 64758 molecules (aa enz PW_2) $cell^{-1}$, according to Supplementary Eq. (12):

$$N_{aa\_enz\_PW2} = N_{enz\_PW2\_r} \cdot n_{enz} \cdot l_{PW2} = N_{aa\_rpc+enz\_PW2} - N_{rs} \cdot n_{rpc} = N_{rs\_enz} \cdot k_{rs} \cdot t_{d\_rs+PW2} \quad (12)$$

The calculated value of $N_{aa\_enz\_PW2}$ can be presented alternatively as N_{enz_PW2} = 215.86 molecules (enz PW_2) cell^{-1} according to the Supplementary Eq. (116). The equivalence of l_{PW2} = 1079.3 reactions PW_2^{-1} and N_{enz_PW2} = 215.86 molecules (enz PW_2) cell^{-1} is explained also considering the difference of values of *generic* k_{enz} = 100 molecules (metabolite) s^{-1} enz^{-1} and *approximate* k_{rs} = 20 molecules (aa) s^{-1} rs^{-1} (Supplementary Table 4). Therefore, the value of $N_{enz\_PW2\_r}$ should be five times smaller than the value of N_{rs} according to Supplementary Eq. (73).

According to Supplementary Eq. (12), $N_{aa\_enz\_PW2} = 1.2 \cdot 10^4$ molecules (aa enz PW_2) cell^{-1} if N_{rs} = 1 molecules (rs) cell^{-1}. This means that effectively only the catalytic "power" of N_{enz_PW2} = 40 molecules (enz PW_2) cell^{-1} (calculated from Supplementary Eq. (116) or from Supplementary Eq. (12) by $N_{aa\_enz\_PW2}/n_{enz}$) is needed to feed one RPC. If $k_{enz} = k_{rs}$, then $N_{enz\_PW2\_r} = N_{rs}$. For example, if N_{rs} = 1 molecules (rs) cell^{-1} and *approximate* k_{rs} = 20 molecules (aa) s^{-1} rs^{-1}, then $N_{aa\_enz\_PW2} = 6 \cdot 10^4$ molecules (aa enz PW_2) cell^{-1} and N_{enz_PW2} = 200 molecules (enz PW_2) cell^{-1}. Alternatively, if $k_{enz} >> k_{rs}$, then $N_{enz\_PW2\_r} << N_{rs}$ and t_{PW2} approaches 0. Balancing the metabolic fluxes of amino acid synthesis and translation leads to the fractional parameter values of N_{cell_comp}.

These calculations indicate that the partial SRS of a typical bacterial cell (*E. coli*) could maintain remarkably long amino acid synthesis pathways during slower growth. In case cells would contain $N_{rs} = 10^4$ molecules (rs) cell^{-1} and l_{PW2} = 1079.3 reactions PW_2^{-1}, the total number of enzymes in the cells could be $N_{enz\_PW2} = 2.2 \cdot 10^6$ molecules (enz PW_2) cell^{-1} (Supplementary Table 29), which is, in fact, a number similar to that of living cells (Table 2 of[22]). However, it should be emphasized that assuming the existence of very long metabolic pathways supporting RPC functioning is most likely not compatible with the metabolic networks of known bacteria. As we shall see, a more reasonable assumption would be that the oversized PW_2 must be interpreted as a sum of other missing cell components of SRS (e.g. enzymes of different synthesis pathways, polymerases).

Distantly, SSPCM-RS+AA (model description in Supplementary Discussion 5.11.4) is similar to the dynamical PTR model[23] which is describing the self-replication of exponentially growing proto-cell population. The PTR model was used to analytically determine μ and various ratios of other cell parameters at steady-state. It was shown that the dependence of μ and the size of the fraction of ribosomes for self-reproduction was non monotonous with optimum (μ_{max}) after solving a complicated quadratic equation. Also, a trade-off was shown between metabolic and ribosomal protein production. Such results were not observed in the case of SSPCM-RS+AA because of differences in cell component interactions (amino acid synthesis pathway missing in PTR, protein degradation missing in SSPCM-RS+AA (Supplementary Figure 2)) and, most importantly, because of completely different base assumptions of models. The trade-off between enzymes and ribosomes was not possible in SSPCM-RS+AA due to strict stoichiometry according to Supplementary Eq. (73). The ratio of $N_{enz\_PW2\_r}$ to N_{rs} (Supplementary Eq. (13)) can be derived from Supplementary Eqs. (73) and (4):

$$\frac{N_{enz\_PW2\_r}}{N_{rs}} = \frac{k_{rs}}{k_{enz}} = \frac{t_{d\_rs+PW2} \cdot k_{rs} - n_{rpc}}{n_{enz} \cdot l_{PW2}} \quad (13)$$

The obtained results of another kinetic model[24] were similarly different compared to SSPCM-RS+AA (Supplementary Figure 2) due to significant differences already in cell components (ATP pool) and interactions (energy balance). However, the prediction of the latter model that μ_{max} is achieved if only ribosomes are produced is valid also for SSPCM-RS+AA assuming that $k_{enz} >> k_{rs}$.

Supplementary Discussion 5.3: Derivations, analysis and calculations based on SSPCM-RS+AA+PROT

Let us assume that a new proto-cell SSPCM-RS+AA+PROT (Supplementary Figure 3, Supplementary Discussion 5.11.5) has similar SRS components as in the case of SSPCM-RS+AA (RPC, amino acid synthesis pathway PW_2), but contains also additional unspecified (their functions are not described) proteins to ensure that l_{PW2} has a *generic* value at $t_{d\_rs+PW2+prot}$ = 3600 s. All proteins are synthesized again by RPCs during $t_{d\_rs+PW2+prot}$ according to Supplementary Eq. (14):

$$t_{d\_rs+PW2+prot} = \frac{N_{rs} \cdot n_{rpc} + N_{enz\_PW2\_r} \cdot n_{enz} \cdot l_{PW2} + N_{aa\_prot}}{N_{rs} \cdot k_{rs}} \quad (14)$$

The unspecified proteins might be undescribed enzymes of other synthesis pathways, other polymerases, or membrane proteins. It is possible to rewrite Supplementary Eq. (14) after the necessary substitution from Supplementary Eq. (73):

$$t_{d\_rs+PW2+prot} = \frac{n_{rpc}}{k_{rs}} + \frac{n_{enz} \cdot l_{PW2}}{k_{enz}} + \frac{N_{aa\_prot}}{N_{rs} \cdot k_{rs}} \quad (15)$$

After rearrangements, it is possible to express the ratio of N_{aa_prot} to N_{rs} by considering Supplementary Eq. (5):

$$k_{rs} \cdot \left(t_{d\_rs+PW2+prot} - t_{d\_rs+PW2}\right) = \frac{N_{aa\_prot}}{N_{rs}} \quad (16)$$

or in original form:

$$t_{d\_rs+PW2+prot} \cdot k_{rs} - n_{rpc} - \frac{k_{rs}}{k_{enz}} \cdot n_{enz} \cdot l_{PW2} = \frac{N_{aa\_prot}}{N_{rs}} \quad (17)$$

Supplementary Eqs. (16)-(17) show that $t_{d\_rs+PW2+prot}$ depends on the values of N_{aa_prot} and N_{rs} (Supplementary Table 30). Note that without the amino acid synthesis enzyme part, Supplementary Eq. (17) is quite similar to equation 4.3 of[25] (excluding the protein degradation part) in their notation describing the translation of ribosomes and nonribosomal proteins.
It is easy to verify that in the case of N_{aa_prot} = 0, Supplementary Eqs. (16)-(17) transform into Supplementary Eq. (5). Therefore, the addition of an unspecified protein fraction to the SRS of SSPCM-RS+AA did not increase the value of minimal $t_{d\_rs+PW2+prot}$ (962.1 s) compared to the value of t_{d_rs+PW2} (Figure 1, Table 1, Supplementary Table 30). The minimal $t_{d\_rs+PW2+prot}$ corresponded to the special case where N_{aa_prot} = 0 and the proto-cell was essentially identical to SSPCM-RS+AA – RPC with the specified amino acid synthesis chain PW_2. Additional derivations of the ratios of different proto-cell parameters have been explained in Supplementary Discussion 5.3.
Assuming that $t_{d\_rs+PW2+prot}$ = 3600 s and N_{rs} = 1 molecules (rs) cell^{-1}, the size of the SRS of the current proto-cell (enzymes of amino acid synthesis pathway PW_2 together with the RPC) is $N_{aa\_rpc+enz\_PW2}$ = 19242 molecules (aa enz PW_2 rpc) cell^{-1} (Supplementary Table 30) according to Supplementary Eq. (11). Again, one RPC can polymerize $N_{aa\_rpc+enz\_PW2+prot}$ = $7.2 \cdot 10^4$ molecules (tot aa) cell^{-1} at $t_{d\_rs+PW2+prot}$ = 3600 s according to Supplementary Eq. (18):

$$N_{aa\_rpc+enz\_PW2+prot} = N_{aa\_rpc+enz\_PW2} + N_{aa\_prot} = N_{rs} \cdot k_{rs} \cdot t_{d\_rs+PW2+prot} \quad (18)$$

This means that N_{aa_prot} = 52758 molecules (aa prot) cell^{-1} calculated from Supplementary Eq. (18) belong to the unspecified protein fraction. Whereas the enzymes of the amino acid synthesis pathway PW_2 are organized into the linear chain of sequentially functioning (linked) enzymes to provide amino acids for the RPC in SSPCM-RS+AA+PROT, the proteins of the unspecified protein fraction could have arbitrary catalytic or structural competence.

The dependence between the ratio of N_{aa_prot} to N_{rs} and between $t_{d\_rs+PW2+prot}$ is always linear according to Supplementary Eq. (17) and the value of the ratio increases with $t_{d\_rs+PW2+prot}$ increase. The dependence between the inverse of the ratio and $t_{d\_rs+PW2+prot}$ is at the same time nonlinear according to Supplementary Eq. (19):

$$\frac{1}{k_{rs} \cdot \left(t_{d\_rs+PW2+prot} - t_{d\_rs+PW2}\right)} = \frac{N_{rs}}{N_{aa\_prot}} \tag{19}$$

Similarly, it is possible to derive the dependence between the ratio of M_{tot} to N_{rs} and between $t_{d\_rs+PW2+prot}$ (Supplementary Eq. (20)):

$$\frac{M_{tot}}{N_{rs}} = \frac{m_{rpc} + \frac{k_{rs}}{k_{enz}} \cdot m_{enz} \cdot l_{PW2} + k_{rs} \cdot \left(t_{d\_rs+PW2+prot} - t_{d\_rs+PW2}\right) \cdot m_{aa}}{DWC} \tag{20}$$

Again, the dependence between the ratio of M_{tot} to N_{rs} and between $t_{d\_rs+PW2+prot}$ is always linear and the value of the ratio increases with $t_{d\_rs+PW2+prot}$ increase. This result can be explained again by the accumulation of amino acids of unspecified proteins in the cell (an increase of N_{aa_prot}) which increases also the M_{tot} value. The dependence between the inverse of the ratio (basically $RS\%_{mc}$) and $t_{d\_rs+PW2+prot}$ is at the same time nonlinear and decreases exponentially with $t_{d\_rs+PW2+prot}$ increase according to Supplementary Eq. (21):

$$\frac{N_{rs}}{M_{tot}} = \frac{DWC}{m_{rpc} + \frac{k_{rs}}{k_{enz}} \cdot m_{enz} \cdot l_{PW2} + k_{rs} \cdot \left(t_{d\_rs+PW2+prot} - t_{d\_rs+PW2}\right) \cdot m_{aa}} \tag{21}$$

It must be stressed that if $t_{d\_rs+PW2+prot}$ is replaced by μ, the dependence between N_{rs}/M_{tot} and μ is linear and N_{rs}/M_{tot} increases with μ increase. Therefore, it reproduces the basic ribosomal protein allocation growth law described and visualized in several publications where very similar proto-cell models were used[6,8,26]. The trends of dependences are qualitatively the same for molecular and mass fractions of RPC in the proteome. Because the SSPCM-RS+AA+PROT model (Supplementary Figure 3) takes into account only proteins in the mass balance, M_{tot} is actually the total mass of proteins in the proto-cell. Therefore, M_{rpc}/M_{tot} is the mass fraction of RPC in the proteome. The calculations showed that the dependence between M_{rpc}/M_{tot} and μ reproduced qualitatively the Fig 3B for the growth without degradation (line at $\gamma = 7.2$ h^{-1} and $\eta = 0$)[8].

The dependence between the ratio of N_{aa_prot} to M_{tot} (basically the content of additional amino acids in the cell) and between $t_{d\_rs+PW2+prot}$ is expressed by the following Supplementary Eq. (22):

$$\frac{N_{aa\_prot}}{M_{tot}} = \frac{DWC}{\frac{m_{rpc} + \frac{k_{rs}}{k_{enz}} \cdot m_{enz} \cdot l_{PW2}}{k_{rs} \cdot \left(t_{d\_rs+PW2+prot} - t_{d\_rs+PW2}\right)} + m_{aa}} \tag{22}$$

It appears from Supplementary Eq. (22) that the dependence between the ratio of N_{aa_prot} to M_{tot} and between $t_{d\_rs+PW2+prot}$ can be perfectly ($R^2 > 0.99$) explained by following second order inverse polynomial (Supplementary Eq. (23)) for the range of $t_{d\_rs+PW2+prot} = 962.1 - 10^4$ s by using known input parameter values from Supplementary Discussion 5.11.2.2.1:

$$\frac{N_{aa\_prot}}{M_{tot}} = 1.52 \cdot 10^{21} - 1.47 \cdot 10^{24} \cdot t_{d\_rs+PW2+prot}{}^{-1} + 2.72 \cdot 10^{24} \cdot t_{d\_rs+PW2+prot}{}^{-2} \tag{23}$$

It must be stressed that the ratio is independent of N_{rs} whereas N_{aa_prot} and M_{tot} separately, of course, depend on N_{rs}. According to Supplementary Eq. (23), the value of the ratio increases with $t_{d\_rs+PW2+prot}$. The dependence between the inverse of the ratio and $t_{d\_rs+PW2+prot}$ (Supplementary Eq. (24)) has a similar shape as for Supplementary Eq. (19):

$$\frac{M_{tot}}{N_{aa\_prot}} = \frac{\frac{m_{rpc} + \frac{k_{rs}}{k_{enz}} \cdot m_{enz} \cdot l_{PW2}}{k_{rs} \cdot \left(t_{d\_rs+PW2+prot} - t_{d\_rs+PW2}\right)} + m_{aa}}{DWC} \tag{24}$$

Different cell parameter ratios (similar to these ratios analysed above) could be and should be used in the design of SRS.

Supplementary Discussion 5.4: Derivations, analysis and calculations based on SSPCM-RS+AA+RNA

The next SRS case is described by SSPCM-RS+AA+RNA which enables to analyze the effect of RNA synthesis in addition to protein synthesis (Supplementary Figure 4, Supplementary Discussion 5.11.6). Compared to the previous model (SSPCM-RS+AA+PROT, Supplementary Discussion 5.11.5), the proto-cell consists of again amino acid synthesis pathway PW_2. RPC is replaced by functional ribosomes by introducing rRNA. Ribonucleotide synthesis pathway PW_4 (*generic* l_{PW4} = 200 reactions PW_4^{-1} (Supplementary Table 5)), RP complexes and reminding fractions of RNA (tRNA, mRNA) are included instead of the unspecified protein fraction.

Firstly, the calculations showed that the value of $t_{d\_rs+PW2+rna}$ increased to 1127.15 s compared to t_{d_rs+PW2} = 962.10 s (Table 1). The effect of the addition of transcription and ribonucleotide synthesis can be estimated only very roughly due to interrelations between cell components. If $l_{PW4} = 0$ in Supplementary Eq. (37), then it is possible to calculate the effect of transcription. According to the calculations, $t_{d\_rs+PW2+rna}$ = 997.33 s which means that transcription (or more precisely the synthesis of RP complexes) took only approximately 35 s. Therefore, the addition of ribonucleotide synthesis pathway PW_4 and additional transcription increased the value of $t_{d\_rs+PW2+rna}$ approximately by 130 s compared to t_{d_rs+PW2}. Note that the synthesis of RNA does not increase the value of $t_{d\_rs+PW2+rna}$ because it takes place in parallel to protein synthesis.

Secondly, the value of $t_{d\_rs+PW2+rna}$ did not depend on N_{rs} and N_{cell_comp} of other cellular components (Supplementary Eqs. (25)-(43), Figure 1, Supplementary Table 33) because N_{cell_comp} of added cell components was fixed to N_{rs} in relevant equations (like rRNA in Supplementary Eq. (92) and tRNA in Supplementary Eq. (93)). In some cases, the connection is not directly observable but it can be traced through multiple equations. For example, N_{rp}

and $N_{enz\_PW4\_r}$ are not directly linked to N_{rs} in any of the equations but the indirect link appears via equations including N_{rrna} and N_{trna}. However, mathematical relationships between the cell component parameters of the SSPCM-RS+AA+RNA are different from those considered earlier due to the increased number of parameters and interactions. For example, in the case of generic mRNA (used for all proteins), the corresponding $t_{d\_rs+PW2+rna}$ is a solution of the quadratic equation (Supplementary Eq. (25)):

$$t_{d\_rs+PW2+rna} = \frac{\frac{n_{rpc}}{k_{rs}} + \frac{n_{enz} \cdot l_{PW2}}{k_{enz}}}{2} + \frac{\left[\left(\frac{n_{rpc}}{k_{rs}} + \frac{n_{enz} \cdot l_{PW2}}{k_{enz}}\right)^2 + 4 \cdot \frac{\left(n_{rp} + \frac{k_{rp}}{k_{enz}} \cdot n_{enz} \cdot l_{PW4}\right) \cdot \left(n_{rrna} + \frac{k_{rs}}{k_{trna}} \cdot n_{trna} + P_{mrna} \cdot n_{mrna}\right)}{k_{rp} \cdot k_{rs}}\right]^{\frac{1}{2}}}{2} \quad (25)$$

Derivation of $t_{d\_rs+PW2+rna}$ using previous Supplementary Eq. (5) gives the following expression (Supplementary Eq. (26)):

$$t_{d\_rs+PW2+rna} = \frac{t_{d\_rs+PW2}}{2} + \frac{\left[t_{d\_rs+PW2}{}^2 + 4 \cdot \frac{\left(n_{rp} + \frac{k_{rp}}{k_{enz}} \cdot n_{enz} \cdot l_{PW4} \right) \cdot \left(n_{rrna} + \frac{k_{rs}}{k_{trna}} \cdot n_{trna} + P_{mrna} \cdot n_{mrna} \right)}{k_{rp} \cdot k_{rs}} \right]^{\frac{1}{2}}}{2} \tag{26}$$

Previous Supplementary Eq. (26) can be further simplified by introducing $t_{d\_cell\_comp}$ (Supplementary Table 31) to describe the doubling of individual cell components based on the following Supplementary Eqs. (27)-(31):

$$t_{d\_rp} = \frac{n_{rp}}{k_{rs}} \tag{27}$$

$$t_{d\_PW4} = \frac{n_{enz} \cdot l_{PW4}}{k_{rs}} \tag{28}$$

$$t_{d\_rrna} = \frac{n_{rrna}}{k_{rp}} \tag{29}$$

$$t_{d\_trna} = \frac{n_{trna}}{k_{rp}} \tag{30}$$

$$t_{d\_mrna} = \frac{n_{mrna} \cdot P_{mrna}}{k_{rp}} \tag{31}$$

Note that Supplementary Eq. (29) is analogical to the defined equation in[2]. The form of the further simplified expression of $t_{d\_rs+PW2+rna}$ (Supplementary Eq. (32)) is following:

$$t_{d\_rs+PW2+rna} = \frac{t_{d\_rs+PW2}}{2} + \frac{\left[t_{d\_rs+PW2}{}^2 + 4 \cdot \left(\frac{\left(t_{d\_rrna} + t_{d\_mrna} \right)}{k_{rs}} + \frac{t_{d\_trna}}{k_{trna}} \right) \cdot \left(\frac{t_{d\_rp}}{k_{rp}} + \frac{t_{d\_PW4}}{k_{enz}} \right) \right]^{\frac{1}{2}}}{2} \tag{32}$$

It must be stressed that Supplementary Eq. (32) can be also expressed by analogical time coefficients of catalytic cell components t_{cell_comp} (Supplementary Table 32) like *generic* t_{PW2} using Supplementary Eq. (6) and following Supplementary Eqs. (33)-(35):

$$t_{rp} = \frac{n_{rp}}{k_{rp}} \tag{33}$$

$$t_{PW4} = \frac{n_{enz} \cdot l_{PW4}}{k_{enz}} \tag{34}$$

$$t_{trna} = \frac{n_{trna}}{k_{trna}} \tag{35}$$

The alternative form of Supplementary Eq. (32) is thus after derivation described by Supplementary Eq. (36):

$$t_{d\_rs+PW2+rna} = \frac{t_{d\_rs} + t_{PW2}}{2} + \frac{\left[\left(t_{d\_rs} + t_{PW2} \right)^2 + 4 \cdot \left(\frac{\left(t_{d\_rrna} + t_{d\_mrna} \right)}{k_{rs}} + \frac{t_{trna}}{k_{rp}} \right) \cdot \left(\frac{t_{rp} + t_{PW4}}{k_{rs}} \right) \right]^{\frac{1}{2}}}{2} \tag{36}$$

However, if specific mRNA molecules (different mRNAs for different proteins) are used then the corresponding $t_{d\_rs+PW2+rna}$ is expressed already by complicated cubic Supplementary Eq. (37):

$$t_{d\_rs+PW2+rna}{}^3 - t_{d\_rs+PW2+rna}{}^2 \cdot \left(\frac{n_{rpc}}{k_{rs}} + \frac{n_{enz} \cdot l_{PW2}}{k_{enz}} \right) - \frac{t_{d\_rs+PW2+rna}}{k_{rs} \cdot k_{rp}} \cdot$$

$$\cdot \left[\left(\frac{k_{rp}}{k_{enz}} \cdot n_{enz} \cdot l_{PW4} \cdot P_{enz} \cdot n_{mrna\_enz} + n_{rp} \cdot P_{rp} \cdot n_{mrna\_rp} \right) + \left(n_{rp} + \frac{k_{rp}}{k_{enz}} \cdot n_{enz} \cdot l_{PW4} \right) \cdot \left(n_{rrna} + \frac{k_{rs}}{k_{trna}} \cdot n_{trna} \right) \right] =$$

$$= \frac{\left(n_{rpc} \cdot P_{rpc} \cdot n_{mrna\_rpc} + \frac{k_{rs}}{k_{enz}} \cdot n_{enz} \cdot l_{PW2} \cdot P_{enz} \cdot n_{mrna\_enz} \right) \cdot \left(n_{rp} + \frac{k_{rp}}{k_{enz}} \cdot n_{enz} \cdot l_{PW4} \right)}{k_{rs}{}^2 \cdot k_{rp}} -$$

$$- \frac{\left(\frac{k_{rp}}{k_{enz}} \cdot n_{enz} \cdot l_{PW4} \cdot P_{enz} \cdot n_{mrna\_enz} + n_{rp} \cdot P_{rp} \cdot n_{mrna\_rp} \right) \cdot \left(n_{rpc} + \frac{k_{rs}}{k_{enz}} \cdot n_{enz} \cdot l_{PW2} \right)}{k_{rs}{}^2 \cdot k_{rp}}$$

(37)

Previous Supplementary Eq. (37) can be further simplified by introducing $t_{d\_cell\_comp}$ (Supplementary Table 31) to describe the doubling of individual cell components based on Supplementary Eqs. (27)-(30). Note that Supplementary Eq. (31) is replaced by the following Supplementary Eqs. (38)-(40) due to the introduction of individual mRNAs:

$$t_{d\_mrna\_rp} = \frac{n_{mrna\_rp} \cdot P_{rp}}{k_{rp}} \tag{38}$$

$$t_{d\_mrna\_rpc} = \frac{n_{mrna\_rpc} \cdot P_{rpc}}{k_{rp}} \tag{39}$$

$$t_{d\_mrna\_enz} = \frac{n_{mrna\_enz} \cdot P_{enz}}{k_{rp}} \tag{40}$$

After substitutions it is possible to rewrite Supplementary Eq. (37) by following Supplementary Eq. (41):

$$t_{d\_rs+PW2+rna}{}^3 - t_{d\_rs+PW2+rna}{}^2 \cdot t_{d\_rs+PW2} -$$

$$-t_{d\_rs+PW2+rna} \cdot \left[\left(t_{d\_PW4} \cdot \frac{P_{enz} \cdot n_{mrna\_enz}}{k_{enz}} + t_{d\_rp} \cdot t_{d\_mrna\_rp} \right) + \left(\frac{t_{d\_rp}}{k_{rp}} + \frac{t_{d\_PW4}}{k_{enz}} \right) \cdot \left(\frac{t_{d\_rrna}}{k_{rs}} + \frac{t_{d\_trna}}{k_{trna}} \right) \right] =$$

$$= \left(\frac{t_{d\_rs} \cdot t_{d\_mrna\_rpc}}{k_{rs}} + \frac{t_{d\_PW2} \cdot t_{d\_mrna\_enz}}{k_{enz}} \right) \cdot \frac{\left(\frac{t_{d\_rp}}{k_{rp}} + \frac{t_{d\_PW4}}{k_{enz}} \right)}{k_{rs}} - \frac{\left(\frac{t_{d\_PW4}}{k_{enz}} \cdot P_{enz} \cdot n_{mrna\_enz} + t_{d\_rp} \cdot t_{d\_mrna\_rp} \right)}{k_{rs}} \cdot$$

$$\cdot \frac{\left(\frac{t_{d\_rs}}{k_{rs}} + \frac{t_{d\_PW2}}{k_{enz}} \right)}{k_{rp}}$$

(41)

Alternatively, Supplementary Eq. (37) can be expressed by t_{cell_comp} using again Supplementary Eq. (6) and Supplementary Eqs. (33)-(35):

$$t_{d\_rs+PW2+rna}{}^3 - t_{d\_rs+PW2+rna}{}^2 \cdot \left(t_{d\_rs} + t_{PW2}\right) -$$
$$-t_{d\_rs+PW2+rna} \cdot \left[\left(t_{PW4} \cdot \frac{P_{enz} \cdot n_{mrna\_enz}}{k_{rs}} + t_{rp} \cdot \frac{P_{rp} \cdot n_{mrna\_rp}}{k_{rs}}\right) + \left(\frac{t_{rp} + t_{PW4}}{k_{rs}}\right) \cdot \left(\frac{t_{d\_rrna}}{k_{rs}} + \frac{t_{trna}}{k_{rp}}\right)\right] =$$
$$= \left(\frac{t_{d\_rs} \cdot t_{d\_mrna\_rpc} + t_{PW2} \cdot t_{d\_mrna\_enz}}{k_{rs}}\right) \cdot \left(\frac{t_{rp} + t_{PW4}}{k_{rs}{}^2}\right) - \left(\frac{t_{PW4} \cdot P_{enz} \cdot n_{mrna\_enz} + t_{rp} \cdot P_{rp} \cdot n_{mrna\_rp}}{k_{rs}{}^2}\right) \cdot \left(\frac{t_{d\_rs} + t_{PW2}}{k_{rs} \cdot k_{rp}}\right)$$
(42)

After further derivation of Supplementary Eq. (42):

$$t_{d\_rs+PW2+rna}{}^3 - t_{d\_rs+PW2+rna}{}^2 \cdot t_{d\_rs+PW2} -$$
$$-\frac{t_{d\_rs+PW2+rna}}{k_{rs}} \cdot \left[t_{PW4} \cdot P_{enz} \cdot n_{mrna\_enz} + t_{rp} \cdot P_{rp} \cdot n_{mrna\_rp} + \left(t_{rp} + t_{PW4}\right) \cdot \left(\frac{t_{d\_rrna}}{k_{rs}} + \frac{t_{trna}}{k_{rp}}\right)\right] =$$
$$= \frac{\left(t_{d\_rs} \cdot t_{d\_mrna\_rpc} + t_{PW2} \cdot t_{d\_mrna\_enz}\right) \cdot \left(t_{rp} + t_{PW4}\right) - \left(t_{PW4} \cdot P_{enz} \cdot n_{mrna\_enz} + t_{rp} \cdot P_{rp} \cdot n_{mrna\_rp}\right) \cdot \frac{t_{d\_rs+PW2}}{k_{rp}}}{k_{rs}{}^3}$$
(43)

By the structure, SSPCM-RS+AA+RNA (Supplementary Discussion 5.11.6) is partially similar to the model[2] which describes the synthesis of ribosomes, rRNA and RP complex. The latter model was used to demonstrate the uniqueness of ribosomal composition which maximizes theoretically μ in cells of *E. coli*. It is further shown that certain properties of both models were similar despite of structural differences. For example, μ value did not depend on N_{cell_comp} and was determined by properties of macromolecules (Eqs. (2), (5) of[2]). The new observed growth law for the ratio of the number of RP polymerizing rRNA to N_{rs} is expressed by Supplementary Eq. (44) in their notation (Eq. (7) of[2]):

$$\frac{\text{number of RNAPs making rRNA}}{\text{number of ribosomes}} \simeq \frac{N_{ribo}^{nucl}}{k_{RNAP}} \cdot \mu \simeq \tau_{rRNA} \cdot \mu \qquad (44)$$

Supplementary Eq. (44) can be also deduced from Supplementary Eqs. (136)-(137) and Supplementary Eq. (92) using SSPCM-RS+AA+RNA by removing tRNA and mRNA terms based on assumption that only rRNA is synthesized by RP complex:

$$t_{d\_rs+rrna} = \frac{N_{rs} \cdot n_{rrna}}{N_{rp} \cdot k_{rp}} \qquad (45)$$

μ is identical to the notation of the current work, N_{ribo}^{nucl} is identical to n_{rrna} and k_{RNAP} is identical to k_{rp} in the current work. The cellular growth boundary for rRNA and RP complex synthesis is expressed by (Eq. (5) of[2]; equation [4] of[27]):

$$\mu \le \sqrt{\frac{k_{RNAP} \cdot \Phi_{RNAP}^{rRNA} \cdot f_{RNAP}^{active}}{N_{RNAP}^{a.a.}} \cdot \frac{k_{ribo} \cdot \Phi_{ribo}^{RNAP} \cdot f_{ribo}^{active}}{N_{ribo}^{nucl}}} \qquad (46)$$

Supplementary Eq. (46) can be also deduced from Supplementary Eqs. (134), (80), (45) of SSPCM-RS+AA+RNA by removing tRNA and mRNA terms based on assumption that only rRNA is synthesized by RP complex and all molecules are active. Supplementary Eqs. (134), (80) are combined assuming that $N_{rs} = N_{rs\_rpc} + N_{rs\_rp}$:

$$t_{d\_rs+rrna} = \frac{N_{rs} \cdot n_{rpc} + N_{rp} \cdot n_{rp}}{N_{rs} \cdot k_{rs}} \qquad (47)$$

N_{rp} is expressed from Supplementary Eq. (45) and substituted in Supplementary Eq. (47):

$$t_{d\_rs+rrna} = \frac{N_{rs} \cdot n_{rpc} + \frac{N_{rs} \cdot n_{rrna}}{t_{d\_rs+rrna} \cdot k_{rp}} \cdot n_{rp}}{N_{rs} \cdot k_{rs}} \quad (48)$$

We can immediately remove N_{rs} from Supplementary Eq. (48) and after simplifying:

$$t_{d\_rs+rrna} \cdot k_{rs} = \frac{n_{rpc} \cdot t_{d\_rs+rrna} \cdot k_{rp} + n_{rrna} \cdot n_{rp}}{t_{d\_rs+rrna} \cdot k_{rp}} \quad (49)$$

After rearrangements it is possible to rewrite Supplementary Eq. (49):

$$t_{d\_rs+rrna} \cdot k_{rp} \left(t_{d\_rs+rrna} \cdot k_{rs} - n_{rpc}\right) = n_{rrna} \cdot n_{rp} \quad (50)$$

The quadratic Supplementary Eq. (50) can be simplified further by dividing both sides by k_{rs} and k_{rp}:

$$t_{d\_rs+rrna} \cdot \left(t_{d\_rs+rrna} - \frac{n_{rpc}}{k_{rs}}\right) = \frac{n_{rrna} \cdot n_{rp}}{k_{rs} \cdot k_{rp}} \quad (51)$$

After substitutions from Supplementary Eq. (3) and already defined terms from Supplementary Eqs. (27) and (29), Supplementary Eq. (51) is modified:

$$t_{d\_rs+rrna} \cdot \left(t_{d\_rs+rrna} - t_{d\_rs}\right) = t_{d\_rrna} \cdot t_{d\_rp} \quad (52)$$

After solving quadratic Supplementary Eq. (52) it is possible to express $t_{d\_rs+rrna}$:

$$t_{d\_rs+rrna} = \frac{t_{d\_rs} \pm \left(t_{d\_rs}{}^{2} - 4 \cdot t_{d\_rrna} \cdot t_{d\_rp}\right)^{\frac{1}{2}}}{2} \quad (53)$$

Compared to[2], the results (Supplementary Eqs. (46), (53)) are still remarkably different. The first difference (besides the lack of ln2 coefficient) is that the ratios of N_{rs} and N_{rp} could be removed during the derivation of Supplementary Eq. (53). Secondly, Supplementary Eq. (46) does not include the linear term of the quadratic equation whereas it is present in Supplementary Eq. (52). Thirdly, the free term of Supplementary Eq. (46) does not incorporate t_{d_rs} at all. The explanation is that only one type of RP fraction (for the synthesis of rRNA) was assumed to be in the proto-cell in the case of Supplementary Eq. (53). In the case of Supplementary Eq. (46), the similar assumption requires Φ_{RNAP}^{rRNA} = 100 %. If Φ_{RNAP}^{rRNA} < 100 %, then also tRNA and/or mRNAs must be synthesized which is not described in[2]. If described, the result would be similar to Supplementary Eq. (37). The explanation for Φ_{ribo}^{RNAP} is that Supplementary Eq. (53) takes into account also ribosome self-replication whereas Supplementary Eq. (46) does not (described by Eq. (2) of[2]). This also explains the lack of t_{d_rs} and the different form of the quadratic equation. If ribosome self-replication is subtracted from the simplified translation balance of Supplementary Eq. (47):

$$t_{d\_rs+rrna} = \frac{N_{rp} \cdot n_{rp}}{N_{rs\_rp} \cdot k_{rs}} \quad (54)$$

N_{rp} is expressed from Supplementary Eq. (45) and after necessary substitution to Supplementary Eq. (54):

$$t_{d\_rs+rrna} = \frac{\frac{N_{rs} \cdot n_{rrna}}{t_{d\_rs+rrna} \cdot k_{rp}} \cdot n_{rp}}{N_{rs\_rp} \cdot k_{rs}} \quad (55)$$

After rearrangements of Supplementary Eq. (55):

$$t_{d\_rs+rrna}{}^2 = \frac{N_{rs} \cdot n_{rrna} \cdot n_{rp}}{N_{rs\_rp} \cdot k_{rs} \cdot k_{rp}} \quad (56)$$

The obtained Supplementary Eq. (56) is already very similar to Supplementary Eq. (46) assuming that $100 \cdot N_{rs\_rp}/N_{rs} = \Phi_{ribo}^{RNAP}$.

A more simplified coarse-grained model of translation involves also tRNA and rRNA (ternary complex) but without describing the transcription process[28]. The latter model was used to describe the μ dependent growth law of RNA composition (tRNA/rRNA) that coincided with theoretical corresponding gene dosage ratios at fast growth. It must be stressed that although the fractional RNA composition depends very slightly on growth in SSPCM-RS+AA+RNA, the ratio of tRNA to rRNA is invariant (usually expected in the literature[22,29]):

$$\frac{M_{trna}}{M_{rrna}} = \frac{k_{rs} \cdot m_{trna}}{k_{trna} \cdot m_{rrna}} \quad (57)$$

Supplementary Eq. (57) is derived from Supplementary Eqs. (92)-(93), (140)-(141). However, the physical meaning of k_{trna} is ambiguous because it is not the usual apparent working rate parameter but rather involves transport and diffusion. Therefore, k_{trna} might have a much more growth condition dependent nature compared to, for example, k_{pol}.

Supplementary Discussion 5.5: Derivations, analysis and calculations based on SSPCM-RS+AA+RNA+LIP

Cells should also have a membrane around their cytoplasm to avoid the dispersion of cell structures. Lipid synthesis pathway PW_5, LPE and membrane lipids were included in the next proto-cell (SSPCM-RS+AA+RNA+LIP (Supplementary Figure 5, Supplementary Discussion 5.11.7)) in comparison to the previous case (SSPCM-RS+AA+RNA). Membrane lipids formed a bilayer membrane (cylindrical shape) whereas other cell components were localized in the cytoplasm. Enclosing of lipids and more importantly geometry changed remarkably mathematical properties of SSPCM-RS+AA+RNA+LIP compared to previous cases (Supplementary Discussion 5.1-5.2, 5.4).

Firstly, the value of the corresponding $t_{d\_rs+PW2+rna+lip}$ depended now on N_{cell_comp} values (in addition to properties of macromolecules). In the case of very small and decreasing N_{cell_comp} values, the value of $t_{d\_rs+PW2+rna+lip}$ increased exponentially to infinity leading to the hockey-stick-type of dependence of $t_{d\_rs+PW2+rna+lip}$ on N_{rs} – see Figure 1 in the main text. For example, N_{rs} = 8.17 molecules (rs) cell^{-1} corresponded to $t_{d\_rs+PW2+rna+lip}$ = 1300 s (Supplementary Table 34). Such dependence appeared because N_{lip} was not directly fixed to N_{rs} in equations but the value was determined by the cell surface area (Supplementary Eq. (149)). A considerable increase in the $t_{d\_rs+PW2+rna+lip}$ value was due to the remarkable change in the ratio of lipids to cytosolic cell components (Figure 2) caused by the change in the surface to volume ratio. The inclusion of geometry added nonlinearities and parameters which made detailed derivations already too impractical for the reader and therefore derivations were left out here. However, the role of geometry and cell membrane will be explained in the next chapter (Supplementary Discussion 5.6) using a simpler proto-cell model SSPCM-RS+LIP (Supplementary Discussion 5.11.8).

Secondly, in the case of large and increasing N_{cell_comp} values, the value of $t_{d\_rs+PW2+rna+lip}$ decreased but not to infinity, instead reaching a minimal value. It appeared that the calculated value of minimal $t_{d\_rs+PW2+rna+lip}$ was equal to the value of $t_{d\_rs+PW2+rna}$ (Table 1, Supplementary Discussion 5.4). Indeed, surrounding proto-cell by lipid cell membrane gave the respective minimum of $t_{d\_rs+PW2+rna+lip}$ = 1127.15 s as in the case of previous proto-cell SSPCM-RS+AA+RNA. This value was calculated at maximal N_{cell_comp} values (N_{rs} =

$6.19 \cdot 10^{14}$ molecules (rs) cell^{-1}) (Supplementary Table 34) and it can be explained again by the change of surface to volume ratio. In the case of large proto-cells, the composition was dominated by cytosolic components whereas membrane lipids and associated cell components (LPE, PW_5 enzymes) were negligible as explained in the next chapter (Supplementary Discussion 5.6). Consequently, the inclusion of lipids, LPE and lipid synthesis pathway PW_5 did not change the value of minimal $t_{d\_rs+PW2+rna+lip}$ compared to $t_{d\_rs+PW2+rna}$ (at maximal N_{cell_comp} values) but the value of $t_{d\_rs+PW2+rna+lip}$ increased with the decrease of N_{cell_comp} values.

Distantly, SSPCM-RS+AA+RNA+LIP is similar to the dynamical model[27] which was used to derive already known (ribosome self-replication, rRNA synthesis) and new (RP, tRNA, aa-tRNA synthetase) closed loop growth laws of autocatalytic cycles in the exponentially growing population of proto-cells. These growth laws were applied to analyse μ dependence on temperature, drug effects on ribosome assembly and cell wall synthesis. However, the derived growth laws cannot be directly compared to the calculated results of SSPCM-RS+AA+RNA+LIP. Firstly, there were many differences between model structures (more complicated polymerization but lack of monomer synthesis and cell geometry in dynamical model[27]). Secondly, the analysis in[27] was carried out in separate modules of the cell whereas all processes were taken into account together in SSPCM-RS+AA+RNA+LIP.

Supplementary Discussion 5.6: Derivations, analysis and calculations based on SSPCM-RS+LIP

To illustrate changes in mathematical properties after the inclusion of cell membrane, let us consider a simpler proto-cell (SSPCM-RS+LIP) consisting only of RPC (that synthesize themselves and LPE), LPE (synthesis of membrane lipids) and membrane lipids that form cell membrane (Supplementary Figure 6, Supplementary Discussion 5.11.8). The following derivation shows that t_{d_rs+lip} depends again on N_{cell_comp} (represented by V_{cyt} currently) and the dependence is qualitatively similar to the previous example (Supplementary Discussion 5.5). By combining Supplementary Eqs. (80), (149), (153)-(156) after necessary substitutions, t_{d_rs+lip} is described by following complicated quadratic equation (Supplementary Eq. (58)):

$$t_{d\_rs+lip}^{2} - t_{d\_rs+lip} \cdot \left(\frac{n_{rpc}}{k_{rs}} + \frac{\left(\frac{V_{cyt}}{\frac{4}{3}+HR}\right)^{\frac{2}{3}} \cdot (4+2\cdot HR)\cdot \pi^{\frac{1}{3}} \cdot m_{lpe}}{V_{cyt}\cdot DWC \cdot s_{lip}\cdot k_{lpe}} \right) = -\frac{\left(\frac{V_{cyt}}{\frac{4}{3}+HR}\right)^{\frac{2}{3}} \cdot (4+2\cdot HR)\cdot \pi^{\frac{1}{3}}}{V_{cyt}\cdot DWC \cdot s_{lip}\cdot k_{lpe}\cdot k_{rs}} \cdot \left(m_{rpc}\cdot n_{lpe} - n_{rpc}\cdot m_{lpe}\right) \quad (58)$$

It is possible to express t_{d_rs+lip} after solving the previous Supplementary Eq. (58):

$$t_{d\_rs+lip} = \frac{\frac{n_{rpc}}{k_{rs}} + \frac{\left(\frac{V_{cyt}}{\frac{4}{3}+HR}\right)^{\frac{2}{3}} \cdot (4+2\cdot HR)\cdot \pi^{\frac{1}{3}} \cdot m_{lpe}}{V_{cyt}\cdot DWC \cdot s_{lip}\cdot k_{lpe}}}{2} \pm \frac{\left[\left(\frac{n_{rpc}}{k_{rs}} + \frac{\left(\frac{V_{cyt}}{\frac{4}{3}+HR}\right)^{\frac{2}{3}} \cdot (4+2\cdot HR)\cdot \pi^{\frac{1}{3}} \cdot m_{lpe}}{V_{cyt}\cdot DWC \cdot s_{lip}\cdot k_{lpe}}\right)^2 - 4\cdot \frac{\left(\frac{V_{cyt}}{\frac{4}{3}+HR}\right)^{\frac{2}{3}} \cdot (4+2\cdot HR)\cdot \pi^{\frac{1}{3}}}{V_{cyt}\cdot DWC \cdot s_{lip}\cdot k_{lpe}\cdot k_{rs}} \cdot (m_{rpc}\cdot n_{lpe} - n_{rpc}\cdot m_{lpe})\right]^{\frac{1}{2}}}{2} \quad (59)$$

The solution above (Supplementary Eq. (59)) can be further simplified by removing *approximate* m_{rpc} and *generic* m_{lpe} based on Supplementary Eq. (111):

$$t_{d\_rs+lip} = \frac{\frac{n_{rpc}}{k_{rs}} + \frac{\left(\frac{V_{cyt}}{\frac{4}{3}+HR}\right)^{\frac{2}{3}} \cdot (4+2\cdot HR)\cdot \pi^{\frac{1}{3}} \cdot n_{lpe}\cdot m_{aa}}{V_{cyt}\cdot DWC \cdot s_{lip}\cdot k_{lpe}}}{2} \pm \frac{\left[\left(\frac{n_{rpc}}{k_{rs}} + \frac{\left(\frac{V_{cyt}}{\frac{4}{3}+HR}\right)^{\frac{2}{3}} \cdot (4+2\cdot HR)\cdot \pi^{\frac{1}{3}} \cdot n_{lpe}\cdot m_{aa}}{V_{cyt}\cdot DWC \cdot s_{lip}\cdot k_{lpe}}\right)^2 - 4\cdot \frac{\left(\frac{V_{cyt}}{\frac{4}{3}+HR}\right)^{\frac{2}{3}} \cdot (4+2\cdot HR)\cdot \pi^{\frac{1}{3}}}{V_{cyt}\cdot DWC \cdot s_{lip}\cdot k_{lpe}\cdot k_{rs}} \cdot n_{rpc}\cdot n_{lpe}\cdot (m_{aa} - m_{aa})\right]^{\frac{1}{2}}}{2} \quad (60)$$

It appears that the free term of the quadratic Supplementary Eq. (58) can be removed from the solution (Supplementary Eq. (60)) because *approximate* m_{aa} – *approximate* m_{aa} = 0 and also:

$$-\frac{\left(\frac{V_{cyt}}{\frac{4}{3}+HR}\right)^{\frac{2}{3}}\cdot(4+2\cdot HR)\cdot\pi^{\frac{1}{3}}}{V_{cyt}\cdot DWC\cdot s_{lip}\cdot k_{lpe}\cdot k_{rs}}\cdot n_{rpc}\cdot n_{lpe}\cdot(m_{aa}-m_{aa})=0 \tag{61}$$

Therefore, the solution (Supplementary Eq. (60)) of the quadratic Supplementary Eq. (58) can be rewritten:

$$t_{d\_rs+lip}=\frac{\frac{n_{rpc}}{k_{rs}}+\frac{\left(\frac{V_{cyt}}{\frac{4}{3}+HR}\right)^{\frac{2}{3}}\cdot(4+2\cdot HR)\cdot\pi^{\frac{1}{3}}\cdot n_{lpe}\cdot m_{aa}}{V_{cyt}\cdot DWC\cdot s_{lip}\cdot k_{lpe}}}{2}\pm\frac{\left[\left(\frac{n_{rpc}}{k_{rs}}+\frac{\left(\frac{V_{cyt}}{\frac{4}{3}+HR}\right)^{\frac{2}{3}}\cdot(4+2\cdot HR)\cdot\pi^{\frac{1}{3}}\cdot n_{lpe}\cdot m_{aa}}{V_{cyt}\cdot DWC\cdot s_{lip}\cdot k_{lpe}}\right)^{2}\right]^{\frac{1}{2}}}{2} \tag{62}$$

It appears from Supplementary Eq. (62) that t_{d_rs+lip} is determined only by the linear coefficient of the quadratic Supplementary Eq. (58). As $t_{d\_rs+lip}=0$ is not feasible, there remains only one solution:

$$t_{d\_rs+lip}=\frac{n_{rpc}}{k_{rs}}+\frac{\left(\frac{V_{cyt}}{\frac{4}{3}+HR}\right)^{\frac{2}{3}}\cdot(4+2\cdot HR)\cdot\pi^{\frac{1}{3}}\cdot n_{lpe}\cdot m_{aa}}{V_{cyt}\cdot DWC\cdot s_{lip}\cdot k_{lpe}} \tag{63}$$

It is now possible to introduce *approximate* t_{d_rs} to Supplementary Eq. (63) based on previous Supplementary Eq. (3) and *generic* t_{lpe} which is defined by the following Supplementary Eq. (64):

$$t_{lpe}=\frac{n_{lpe}}{k_{lpe}} \tag{64}$$

Finally, t_{d_rs+lip} is expressed in Supplementary Eq. (65) after substitutions:

$$t_{d\_rs+lip}=t_{d\_rs}+\frac{\left(\frac{V_{cyt}}{\frac{4}{3}+HR}\right)^{\frac{2}{3}}\cdot(4+2\cdot HR)\cdot\pi^{\frac{1}{3}}\cdot t_{lpe}\cdot m_{aa}}{V_{cyt}\cdot DWC\cdot s_{lip}} \tag{65}$$

It is possible to conclude from Supplementary Eq. (65) that t_{d_rs+lip} depends on N_{cell_comp} (and V_{cyt}) and the shape of the dependence is determined by the ratio of $V_{cyt}^{2/3}/V_{cyt}$. The calculations showed that the second part of the sum Supplementary Eq. (65) was quite insignificant at bigger proto-cell sizes. If V_{cyt} = 10^{-13} – 10^{-12} cm^3 cyt^{-1} then t_{d_rs+lip} = 362.14 – 362.18 s (Supplementary Table 35). The minimal t_{d_rs+lip} = *approximate* t_{d_rs} according to the following case at very large cell size (Table 1) if:

$$\frac{\left(\frac{V_{cyt}}{\frac{4}{3}+HR}\right)^{\frac{2}{3}}\cdot\left(4+2\cdot HR\right)\cdot\pi^{\frac{1}{3}}\cdot t_{lpe}\cdot m_{aa}}{V_{cyt}\cdot DWC\cdot s_{lip}}=0 \qquad (66)$$

Only in the case of considerably smaller cells and $V_{cyt}^{2/3}/V_{cyt} > 10^{25}$ the second part of the sum Supplementary Eq. (65) started to increase together with t_{d_rs+lip}. For example, $V_{cyt} = 1.75\cdot10^{-27}$ cm^3 cyt^{-1} and $N_{rs} = 3.78\cdot10^{-11}$ molecules (rs) cell^{-1} corresponded to t_{d_rs+lip} = 3520 s (Supplementary Table 35).

It means that the removal of all monomer synthesis pathways and RNA decreased considerably the value of t_{d_rs+lip} compared to previous *approximate* t_{d_srs} values but minimal t_{d_rs+lip} was equal with *approximate* t_{d_rs} and was determined only by properties of ribosomes (Table 1). Unchanged minimal t_{d_rs+lip} compared to *approximate* t_{d_rs} is explained by considerable changes in the ratio of surface to volume of the proto-cell due to which the macromolecular composition had only $LIP\%_{mmc} = 10^{-7}$ % (g (tot lip) (g (dw cell))$^{-1}$) of lipids in dry weight mass of the proto-cell. Evidently, such a small fraction did not have almost any effect on minimal t_{d_rs+lip}.

Supplementary Discussion 5.7: Derivations, analysis and calculations based on SSPCM-RS+AA+RNA+LIP+MPROT

The further addition of SRS components (membrane proteins (ETC, substrate transport protein), central metabolic pathway PW_1) changed several mathematical properties in the corresponding proto-cell (SSPCM-RS+AA+RNA+LIP+MPROT) (Supplementary Figure 7, Supplementary Discussion 5.11.9) compared to previous cases (Supplementary Discussions 5.4-5.5). Firstly, it increased the corresponding calculated minimal $t_{d\_rs+PW2+rna+lip+mprot}$ value to 2462.68 s (Supplementary Table 36). Such an increase was considerable (more than 2 times) compared to the values of minimal $t_{d\_rs+PW2+rna+lip}$ and $t_{d\_rs+PW2+rna}$ (Table 1). Again, it was difficult to estimate the effects of individual cell components due to the interrelations between them. If $l_{PW1} = 0$ then minimal $t_{d\_rs+PW2+rna+lip+mprot}$ = 1865.4 s which means that the synthesis of membrane proteins increased the value of minimal $t_{d\_rs+PW2+rna+lip+mprot}$ by approximately 738 s compared to the value of $t_{d\_rs+PW2+rna}$. However, the additional calculated gap of approximately 597 s (2462.68 – 1865.4) cannot be taken entirely as the effect of the central metabolic pathway PW_1.

Secondly, the inclusion of membrane proteins changed the growth limitation factor as minimal $t_{d\_rs+PW2+rna+lip+mprot}$ was determined by the cell surface (Figure 2). Assuming that k_{cell_comp} of cell components have constant values, faster growth can be achieved only by increasing the number of catalysts. Therefore, the values of N_{cell_comp} of membrane proteins on the cell membrane increased due to the increased demand for energy and substrates (Supplementary Table 36). At the same time, the value of S_{lip} gradually decreased to 0 due to changes in the surface area to volume ratio. Certainly, there is some kind of minimal value of S_{lip}/S_{tot} in living cells that is needed to maintain the integrity of cell membrane and therefore minimal $t_{d\_rs+PW2+rna+lip+mprot}$ has probably higher value.

Finally, $t_{d\_rs+PW2+rna+lip+mprot}$ depended also hyperbolically on N_{cell_comp} as in the previous three cases (introduction of unspecified protein in SSPCM-RS+AA+PROT (Supplementary Discussion 5.3), the introduction of membrane lipids and cell geometry in SSPCM-RS+AA+RNA+LIP (Supplementary Discussion 5.5) and SSPCM-RS+LIP (Supplementary

Discussion 5.6)) and the dependence manifested remarkably only in cases of small values of N_{cell_comp} (Figure 1). For example, N_{rs} = 2.22 molecules (rs) cell^{-1} corresponded to $t_{d\_rs+PW2+rna+lip+mprot}$ = $3 \cdot 10^3$ s (Supplementary Table 36). In the case of higher values of N_{rs}, the $t_{d\_rs+PW2+rna+lip+mprot}$ was not noticeably depending on the N_{rs} approaching asymptotically to the minimal value. Due to changing ratio of volume to the surface area of the proto-cell and the increase of relative surface covered by membrane proteins, the N_{lip}/N_{rs} decreased during the M_{tot} increase (Figure 2). Therefore, less enzymes, energy and monomers were needed for lipid synthesis resulting in decreases of relative contents of cell components per ribosomes and decrease of doubling time. However, in the case of smaller cells the value of N_{rs} was rather low (< 100 molecules (rs) cell^{-1}) compared to other cell components and the cell required notably longer $t_{d\_rs+PW2+rna+lip+mprot}$ for reproduction. As most of the energy and substrate were consumed by translation, it is reasonable to assume that the values of N_{etc}, N_{stp} and $N_{enz\_PW2\_r}$ were mainly stoichiometrically fixed to N_{rs} values. Therefore, the inclusion of these cell components was not mainly responsible for the nonlinear dependence between $t_{d\_rs+PW2+rna+lip+mprot}$ and N_{rs}. Presumably, this dependence was again imposed by cell geometry (Supplementary Discussions 5.5-5.6) and was preserved also in SSPCM-RS+AA+RNA+LIP+MPROT. Lipids and related cell components (LPE, lipid synthesis pathway) were exceptional among other cell components because their N_{cell_comp} values dropped significantly around minimal $t_{d\_rs+PW2+rna+lip+mprot}$ values due to the increasing need for more membrane proteins (ETC, substrate transporter) (Supplementary Table 36). Eventually, there was not enough area for the lipids on the surface, which caused the membrane to lose its integrity and made further growth even theoretically impossible. Note that N_{lip} was not a growth limiting factor in the two previous steps as the membrane was covered only by lipids.

Supplementary Discussion 5.8: Derivations, analysis and calculations based on SSPCM-SRS-M

Finally, a single copy of the genome and other cell components associated with the synthesis of DNA (RC, deoxyribonucleotide synthesis pathway PW_3) were included in the previous proto-cell SSPCM-RS+AA+RNA+LIP+MPROT. Therefore, the complete minimal SRS of bacterial cell growing on a minimal medium was now described (SSPCM-SRS-M, (Supplementary Discussion 5.11.2, Figure 3, Supplementary Tables 1-3)). The calculations showed that the addition of DNA replication also led to the appearance of hockey-stick-type-dependence of the $t_{d\_srs\text{-}m}$ on the N_{rs} (Figure 1), although the mathematical properties and corresponding equations were different in these cases (Supplementary Discussion 5.9). In short, DNA and associated cell components also contributed (their N_{cell_comp} values are not stoichiometrically fixed to N_{rs} values) to the dependence beside cell geometry.
The calculations also showed that the value of $t_{d\_srs\text{-}m\_min}$ was approximately 2474.27 s (Supplementary Table 27) (determined again by N_{lip} (Figure 2)) meaning that the increase of $t_{d\_srs\text{-}m\_min}$ was not considerable compared to the previous step (SSPCM-RS+AA+RNA+LIP+MPROT) (Table 1). The total mass of DNA associated cell components was relatively small ($DNA\%_{mmc}$ = 1 % (g (tot dna) (g (dw cell))$^{-1}$)) and independent of growth at $t_{d\_srs\text{-}m\_min}$ because N_{dna} and N_{rc} values did not change (single genome and a pair of RC was expected to be in all growth conditions (Supplementary Table 24)). However, the latter property meant that the total mass of DNA associated cell components during slower growth was relatively high ($DNA\%_{mmc}$ = 75 % (g (tot dna) (g (dw cell))$^{-1}$)) and therefore also relatively high mass of other cell components was accompanied. For example, N_{rs} = 17.45 molecules (rs) cell^{-1} corresponded to the $t_{d\_srs\text{-}m}$ = 10^4 s (Supplementary Table 27) which was 5

orders of magnitudes higher than for the previous step (SSPCM-RS+AA+RNA+LIP+MPROT).

The calculated $t_{d\_srs\text{-}m\_min}$ = 2474.27 s for SSPCM-SRS-M (includes all main macromolecules of minimal functional SRS growing on minimal medium) is somewhat lower than experimentally determined values of *E. coli* cells growing on a minimal medium which remain usually below 1 doubling per hour[21,30]. This difference can be explained by various factors. Current calculations described only cell components that were necessary for reproduction at certain conditions. Living cells, however, include various polymers and molecules for secondary functions (regulatory, reserves etc). Besides, calculations excluded constraints imposed by cell cycle that are available in living cells, doubling time limit was defined at N_{lip} = 0 which is probably too extreme as already mentioned etc. These details were left aside because the aim was to estimate minimal t_{d_srs} (constrained by SRS, necessary for cell reproduction). However, there are also reports[31] that *E. coli* cells growing on minimal glucose medium can reach up to t_{d_min} = 2570 s (calculated from μ_2 = 1.4 doublings h^{-1} according to Supplementary Eq. (67)) which is comparable to the calculated $t_{d\_srs\text{-}m\_min}$. Therefore, current calculations are partly in the range of experimentally determined values. At the same time, it must be cautious with the conclusion that living cells sometimes only consist of SRSs as the results of theoretical models cannot be directly applied to living cells/strains.

$$t_d = \frac{3600}{\mu_2} \tag{67}$$

Supplementary Discussion 5.9: Derivations, analysis and calculations based on SSPCM-RS+DNA

It must be stressed that DNA has a similar effect on t_{d_srs} as did membrane lipids and proto-cell geometry although the mathematical properties and corresponding equations are different. To illustrate more uniqueness of DNA among other cell components, let us examine simpler proto-cell (SSPCM-RS+DNA) consisting only of RPC (that synthesize themselves and enzymes of PW_3), pathway PW_3 with corresponding enzymes (synthesis of deoxyribonucleotides) and DNA (Supplementary Figure 8, Supplementary Discussion 5.11.10). DNA replication rate is equal to the synthesis rate of deoxyribonucleotides and takes place during t_{d_rs+dna} according to the following Supplementary Eq. (68) (combined Supplementary Eqs. (67), (174)):

$$N_{enz\_PW3\_r} \cdot k_{enz} = \frac{N_{dna} \cdot n_{dna}}{t_{d\_rs+dna}} \tag{68}$$

At the same time, ribosomes produce all necessary proteins including RPC during t_{d_rs+dna} according to the following Supplementary Eq. (69) (combined Supplementary Eqs. (80), (170)-(172)):

$$t_{d\_rs+dna} = \frac{N_{rs} \cdot n_{rpc} + N_{rc} \cdot n_{rc} + N_{enz\_PW3\_r} \cdot n_{enz} \cdot l_{PW3}}{N_{rs} \cdot k_{rs}} \tag{69}$$

As N_{dna} and N_{rc} have determined *approximate* values (Supplementary Table 24), it is possible to calculate two unknown parameters from the system of equations (Supplementary Eqs. (68)-(69)). If N_{rs} is variable, it is possible to make the necessary substitution of $N_{enz\_PW3\_r}$ and find the symbolic solution of the resulting quadratic equation for t_{d_rs+dna} (Supplementary Eq. (70)):

$$t_{d\_rs+dna} = \frac{N_{rs} \cdot n_{rpc} + N_{rc} \cdot n_{rc} \pm \sqrt{\left(\left(-\left(N_{rc} \cdot n_{rc} + N_{rs} \cdot n_{rpc}\right)\right)^2 + 4 \cdot N_{dna} \cdot n_{dna} \cdot N_{rs} \cdot n_{enz} \cdot l_{PW3} \cdot k_{rs}\right)}}{2 \cdot N_{rs} \cdot k_{rs}} \tag{70}$$

It can be immediately seen from Supplementary Eq. (70) that t_{d_rs+dna} is indeed not constant, and the value depends on N_{cell_comp}. However, t_{d_srs} is worse than other unknown cell parameters in terms of solving efficiency and transparency of the solution. If t_{d_rs+dna} is a variable and N_{rs} is an unknown parameter, then the system of equations (Supplementary Eqs. (68)-(69)) can be solved more easily by making necessary substitutions and the symbolic solution of N_{rs} is expressed by following Supplementary Eq. (71):

$$N_{rs} = \frac{N_{rc} \cdot n_{rc} \cdot t_{d\_rs+dna} \cdot k_{enz} + N_{dna} \cdot n_{dna} \cdot n_{enz} \cdot l_{PW3}}{t_{d\_rs+dna} \cdot k_{enz} \cdot \left(t_{d\_rs+dna} \cdot k_{rs} - n_{rpc}\right)} \tag{71}$$

Firstly, it appears from Supplementary Eq. (71) that N_{rs} increases exponentially if t_{d_rs+dna} decreases (Supplementary Table 37). Secondly, the doubling time limit of the proto-cell or minimal t_{d_rs+dna} is determined by the following expression from the denominator of the previous Supplementary Eq. (71):

$$t_{d\_rs+dna} \cdot k_{rs} - n_{rpc} = 0 \tag{72}$$

N_{rs} drops sharply from maximal value to 0 value at minimal t_{d_rs+dna} according to the previous Supplementary Eqs. (71)-(72). Thirdly, minimal t_{d_rs+dna} is exactly equal to the symbolic solution of *approximate* t_{d_rs} and thus determined by the properties of ribosomes and not by the properties of DNA associated cell components (Table 1). As mentioned before, in terms of mathematical properties the uniqueness of DNA among other cell components in the proposed SSPCM-RS+DNA is related to the independence from growth and lack of fixed stoichiometric relations with ribosomes. The amount of DNA (and associated synthesis equipment) is negligible compared to the value of N_{rs} at minimal t_{d_rs+dna} (Supplementary Table 37). On the other hand, $DNA\%_{mmc}$ = 100 % (g (tot dna) (g (dw cell))$^{-1}$) at $t_{d\_rs+dna} = 10^4$ s whereas N_{rs} = 3.4 molecules (rs) cell^{-1}.

It must be stressed that faster growth (t_{d_rs+dna} < *approximate* t_C) of the proto-cell is possible only if the value of k_{dp} increases with μ to ensure replication rate values vary over a fairly wide range (as explained in Supplementary Discussion 5.11.10.3). A similar assumption was made for SSPCM-SRS-R (Supplementary Discussion 5.11.11.3).

Supplementary Discussion 5.10: Derivations, analysis and calculations based on SSPCM-SRS-R

It can be concluded from the previous results of proto-cell SRS calculations (Supplementary Discussions 5.1-5.2, 5.4-5.5, 5.7-5.8) that the increase of l_{PWi} or adding new cell components to the proto-cell will inevitably increase the value of t_{d_min}. However, if cells are growing on a medium supplemented with all necessary monomers of macromolecules, then biosynthesis pathways along with corresponding enzymes are theoretically not needed. Therefore, less ribosomes and RP complexes are needed to synthesize proteins and RNA. Such an extreme case corresponds to the proto-cell model SSPCM-SRS-R (Supplementary Figure 9, Supplementary Discussion 5.11.11). Calculations showed that the decrease of SRS enabled also to decrease the value of $t_{d\_srs\text{-}r\_min}$ approximately down to 936.4 s which was comparable to the value of t_{d_rs+PW2} and more than 2 times lower than $t_{d\_srs\text{-}m\_min}$ (Table 1). The calculations reproduced the very well-known fact that growth on a rich medium is considered to be faster than on a minimal medium. It must be stressed that faster growth ($t_{d\_srs\text{-}r}$ < *approximate* t_C) of SSPCM-SRS-R is possible only if the value of k_{dp} increases with μ to

ensure replication rate values vary over a fairly wide range (as explained in Supplementary Discussion 5.11.11.3).

Unsurprisingly, the dependence between $t_{d\_srs\text{-}r}$ and N_{rs} has already a familiar shape (weaker dependence in the case of larger proto-cells and sharp changes in the case of smaller cells). Note that there are now less cell components in the proto-cell whose values of N_{cell_comp} are stoichiometrically fixed to N_{rs} values compared to SSPCM-SRS-M. The value of $t_{d\_srs\text{-}r\_min}$ is again determined by the cell membrane surface area (Supplementary Table 38).

There is a considerable variation of experimentally determined μ_{max} values in the literature. For example, the comparison of calculated $t_{d\_srs\text{-}r\_min}$ and experimentally determined μ_{max} values of *E. coli* cells growing on different rich medium[30] showed that calculated $t_{d\_srs\text{-}r\_min}$ values were considerably smaller. Experimentally determined values of μ_{max} = 1.26 – 1.62 h^{-1} or t_d = 1540 – 1980 s depending on the medium were approximately 2 times slower than proto-cells described by SSPCM-SRS-R. Certainly, this comparison indicates that there are more factors affecting μ of living cells in addition to those discussed above and starting with the growth environment. Environmental parameters (pH, T, concentrations of chemical substances etc) have a long list of effects on k_{cell_comp} values, macromolecule conformations, switching between different metabolic pathways and regulation networks etc. It must be also stressed that experimentally determined μ_{max} values were mostly measured in batch cultivations that have several disadvantages (nonstationary states, inhibition effects) compared to more advanced continuous cultures[32]. Another problem with the comparison is that growth conditions (all monomers available in growth medium) described in SSPCM-SRS-R probably do not correspond entirely to experimental cultivation conditions[30]. The analysis of the consumption of different constituents of tryptone and yeast extract in a lysogeny broth medium is tremendously difficult but certainly, the cells consume also various peptides, nucleosides, cofactors etc besides amino acids. In conclusion, detailed comparison can be done only on the level of organism specific model.

On the other hand, there are also reports[31] that cells of *E. coli* are able to grow much faster reaching t_{d_min} = 1200 s (calculated from μ_2 = 3 doublings h^{-1} according to Supplementary Eq. (67)) which is only approximately 25 % higher than calculated $t_{d\_srs\text{-}r\_min}$ (comparable to the values of $t_{d\_rs+PW2+rna}$ and minimal $t_{d\_rs+PW2+rna+lip}$ (Table 1)).

Experimentally determined very low generation times (t_{d_min} < 10 minutes) of food pathogens[33] are at variance with the calculation results at first view. The comparison gives the impression that cells of *C. perfringens* consist of only ribosomes (RPC) (Table 1). Dismissing all doubts about experimental errors, it is more likely that molecular and cellular parameters of pathogens are tuned to higher μ. For example, the value of n_{dna} is approximately 25 % smaller than that in *E. coli* (SSPCM-SRS-R) and probably the growth is not so easily limited by cell membrane area because energy is not synthesized on the membrane in cells of this anaerobic organism[34]. However, the most critical aspects are the values of k_{cell_comp}. They must be considerably higher than those of *E. coli* in order to get much higher μ values.

Supplementary Discussion 5.11: Descriptions of used models

Used SSPCMs (Figure 3, Supplementary Figures 1-9) involve various cellular interactions (cellular processes and reactions, stoichiometric dependencies etc.) (Supplementary Table 3) between different model components (Supplementary Table 2) that are shortly reviewed in Supplementary Discussion 5.11.2 for SSPCM-SRS-M. Cellular model components and interactions comprise the system of linear and nonlinear algebraic equations (Supplementary Eqs. (73)-(125) for SSPCM-SRS-M) based on a list of simplifications (Supplementary Discussion 5.11.2) and assumptions (steady-state, exact cell doubling, linear growth law of

the cell etc) presented in Supplementary Discussion 5.11.1 providing stoichiometric models. The equations define various balances (cell components, time, mass/volume, surface area) in the models and are more explicitly described in the following subsections. Parameters of equations (and of model components) are defined and input parameter values necessary to carry out calculations are provided in Supplementary Discussions 5.11.2.2, 5.11.3.2, 5.11.4.2, 5.11.5.2, 5.11.6.2, 5.11.7.2, 5.11.8.2, 5.11.9.2, 5.11.10.2 and 5.11.11.2. Input parameters correspond mostly to such cellular parameters (like dimensions and compositions of molecules) that are determined only by genotype (Supplementary Tables 4-24) whereas output parameters depend also on cell growth (like cell size, flux patterns, numbers of biopolymers) (Supplementary Tables 25-26). The majority of the values of input parameters are *specific, precise, average* or *approximate* corresponding to values of *E. coli* K12 MG1655 if possible. The remaining smaller part of parameter values is *generic* or variables. To simplify the solving of models, the number of different model components and the number of relations between them are equal which enables to find unique solutions without optimization (Supplementary Discussions 5.11.2.3, 5.11.3.3, 5.11.4.3, 5.11.5.3, 5.11.6.3, 5.11.7.3, 5.11.8.3, 5.11.9.3, 5.11.10.3 and 5.11.11.3). Also, the selection of input and output parameters enables to simplify model solving by carrying it out in different steps and part of the equations (for example equations describing DNA replication, flux patterns, cellular compositions or geometry properties, presented in Supplementary Tables 25-26)) can be solved and corresponding parameters calculated separately after solving the main system of equations (Supplementary Discussion 5.11.2.3). Some values of calculated output parameters are provided in Supplementary Tables 27-30, 33-38.

SUPPLEMENTARY DISCUSSION 5.11.1: BASE ASSUMPTIONS OF USED MODELS

Used SSPCMs are based on the following main general assumptions which enable to describe the growth of cells by simple algebraic equations and reduce the complexity of calculations:

1. M_{tot} and all cell components (N_{cell_comp}) are exactly doubled t_{d_srs}. It means that values of cell parameters before the start of the synthesis processes are integrals (over doubling time t_{d_srs}) of respective cell parameters' values of cell components synthesized during t_{d_srs}.
2. M_{tot} and N_{cell_comp} are growing according to the linear growth law[3,35,36]. It is assumed that the numbers of active cell components (ribosomes, polymerases, enzymes etc.) are the same throughout t_{d_srs} – these numbers are calculated from the main equation system of the models (Supplementary Discussion 5.11.2.3). Additional N_{cell_comp} synthesized during t_{d_srs} are not considered active. The responsible mechanism is not specified in the current work and it is not specified which cell components (old or new) are active. It is also assumed that all processes follow the linear function. Compared to other alternative growth laws[37-42], this assumption allows mathematical simplifications as the active N_{cell_comp} and F_{cell_comp} values remain constant during the synthesis if catalysing cell components (polymerases, enzymes) of the cell have constant k_{cell_comp} that do not depend on t_{d_srs}.
3. Cell metabolism is in stationary state[32,43]. Metabolite concentrations are not changing during growth which means that synthesis and degradation fluxes of metabolites are equal. This enables to simplify the model considerably as changes in metabolite pools are not taken into account in balance equations. Stationary state assumption is also very common in most of the metabolic models[32] and

experimentally it is realized presumably in continuous cultures (chemostat family cultures).

4. It is assumed that cell metabolism is optimized and organized so that the values of all cell parameters (numbers of cell components, fluxes of reactions/pathways/processes etc.) are precisely balanced and all cell components are necessary for self-replication (with certain exceptions explained below). Before the start of the synthesis processes, all cell components are active, they are participating in synthesis processes and there are no free cell components. It means for example that the values of F_{cell_comp} correspond exactly to the values of N_{cell_comp} considering their k_{cell_comp} values. This assumption enables to keep the number of independent cell parameters minimal and reduces the number of unknowns (solution space) but, on the other hand, it produces a more complex system of equations that can not be further simplified. However, the models used in the current work were small enough and model calculations were carried out with reasonable time and resources. Alternative theories of suboptimal growth[44], mechanisms behind maintenance costs[45], stochastic nature of polymerisation processes and role of posttranslational regulation in the coordination of enzyme expression and fluxes[46] are not included in the models. As said before, there are a few exceptions (periodical cell processes). Parameter values of periodical processes are averaged over the t_{d_srs} (according to the linear growth law as mentioned above). It means for example that the value of N_{rc} is usually higher than the value of N_{rce}.
5. It is assumed that the pool sizes of intracellular metabolites (including monomers of biopolymers) are very small and they do not affect anyhow cell processes. Also, there are no limitations by the spatial distribution of cell components in the cell.
6. It is expected that corresponding proto-cells include only a single genome and a pair of RC before the start of the synthesis processes although it is widely known that the values of N_{dna} and N_{rc} change with t_d in living cells. Such dependencies are omitted in the current work to simplify the model. It is assumed that the gene copy number is not the growth limiting factor.
7. It is assumed that all cell processes (synthesis, transport etc.) are taking place in parallel and continuously (including periodical cell processes). In other words, developed SSPCMs do not specify a timely line up for cell processes (regulational aspects are not included) and the models are essentially stoichiometric, clearly not kinetic.

SUPPLEMENTARY DISCUSSION 5.11.2: DESCRIPTION OF SSPCM-SRS-M

A condensed view of the description of SSPCM-SRS-M is included in this chapter as an understanding of simplifications introduced in the model is important for the comparison of the SSPCMs and living cells, especially taking into account that not all simplifications can be understood easily from the equations (Supplementary Eqs. (73)-(125)) provided in following subchapters. The results of the calculations obtained with SSPCMs are in most cases similar and transferrable to those of living cells. Therefore, a more concise description of the simplifications is warranted.

SSPCM-SRS-M is structured explicitly into cytoplasmic space and bilayer cell membrane as known from the standard textbooks (Supplementary Table 1, Figure 3). Most of the cell components are localized in the cytoplasmic space except membrane lipids, transport proteins and ETC complexes that are integrated into the cell membrane, as also known very well. The

information on the localization of cell components is necessary to include cell geometry and size calculations in the model. The list of cell components of SSPCM-SRS-M is also traditional, and it is presented in (Supplementary Tables 1-3):

Supplementary Table 1. Cellular structures and locations of cell components in SSPCM-SRS-M.

Structure	Cell components (Supplementary Table 2)	Processes (Supplementary Table 3)
Cytoplasm	DNA.	DNA replication.
	All RNA.	Transcription.
	All proteins except transport protein and electron transport chain (ETC) complex.	Translation. Membrane lipid synthesis.
	Monomers and building blocks.	Biosynthesis of monomers.
		Central metabolic pathway PW_1.
Cell membrane	Transport protein.	Substrate transport.
	ETC complex.	ATP synthesis.
	Membrane lipids.	Cell membrane formation.

A more detailed description of biopolymers and other cellular components along with equations are presented in Supplementary Discussions 5.11.2.1.1-5.11.2.1.8. The main classes of simplifications are:

1. All biopolymers are composed of *average* monomers (in terms of m_{mon} (Supplementary Tables 16-20)).
2. Biopolymer complexes (assembled rRNA complex, RC, RP, RPC, ETC complex, transport protein) are single molecules without subunits (monomeric sequences) in the models for simplification purposes but their n_{cell_comp} values correspond to *specific*, *precise* or *approximate* lengths of actual subunits with appropriate stoichiometries in *E. coli* K12 MG1655 (Supplementary Tables 10-15).
3. Part of the cell components (mRNA, LPE, enzyme of metabolic pathways PW_1-PW_5) have *generic* parameter values (Supplementary Tables 4-6, 9-10, 16, 21-22). Substrate and intermediates of the metabolic network are unspecified molecules without specified structure and molecular characteristics (parameters).

A circular bihelical chromosome is composed of *average* (in terms of m_{dnt}) deoxyribonucleotides linked together by phosphodiester bonds – this assumption allows to calculate more easily the need for energy (ATP) for the polymerization reactions using the stoichiometries known from biochemistry.

RNA fraction includes all the main types of cellular RNA – assembled rRNA complex for ribosomes, *average* (in terms of n_{trna}) tRNA and different mRNAs for all proteins (Figure 3). All RNA types consist of *average* (in terms of m_{nt} (Supplementary Table 16)) ribonucleotides linked together by phosphodiester bonds. Only one universal *average* (in terms of n_{trna} (Supplementary Tables 10, 13)) tRNA is introduced as different amino acids are not specified. The parameters of rRNA are based on 3 subunits (5S, 16S, 23S – *E. coli*) assembled with 1:1:1 stoichiometry (one copy of each subunit) (Supplementary Tables 10, 12, 16). There are specific mRNAs coding different proteins. It is assumed that mRNAs contain only coding regions.

Enzymes with *generic* n_{enz} = 300 molecules (aa) enz^{-1} (Supplementary Table 10) catalyse all reactions in central metabolic and biosynthesis pathways PW_1-PW_5.

Membrane lipid synthesis is also carried out by LPE with *generic* parameters (Supplementary Tables 4, 10, 16). Ribosomes, RP, RC, LPE carry out polymerisation processes (Figure 3).

Values of parameters of RC are based on different enzymes and proteins with fixed stoichiometries (*E. coli*) (Supplementary Tables 4, 10, 15-16). The *approximate* values of the parameters of RP are based on different subunits as in *E. coli* (Supplementary Tables 4, 10, 14, 16). Energy is produced on the membrane by ETC with *approximate* parameter values (Supplementary Tables 4, 10, 16) and the substrate is transported to the cell through transport protein with *approximate* parameter values (based on data of *E. coli*) (Figure 3, Supplementary Tables 4, 10, 16).

A simplified proto-cell contains only one type of membrane lipid that forms a bilayer cell membrane (Figure 3).

SSPCM-SRS-M includes all main cell processes and interactions between cell components needed for proto-cell reproduction (Supplementary Tables 2-3). There are all kinds of simplifications in all details of cell interactions but the main classes of simplifications are:

1. Polymerisation processes (DNA replication, transcription, translation) have different steps (maturation, folding, processing etc) in living cells but these steps are not explicitly described in the model.
2. Metabolic network is composed of 5 linear chains ($PW_{1\text{-}5}$) of isomerization reactions with identical unspecific stoichiometries. It means that $N_{enz\_PWi\_r}$ for each reaction of the same pathway PW_i are exactly equal assuming that k_{enz} is constant. Also, $F_{enz\_PWi\_r}$ for each reaction of the same pathway PW_i are exactly equal. All reactions are strictly unidirectional which means that there are only net fluxes as it is possible to exclude exchange fluxes of enzymes operating close to the thermodynamic equilibrium.
3. Cell has an ideal cylindrical shape with spherical caps which resembles the idealized shape of an *E. coli* cell[47]. It is assumed that S_{tot} is equal for both lipid layers. M_{tot} and V_{tot} have equal values assuming that *generic* ρ_{tot} = 1 g (cell) (cm^3 (cell))$^{-1}$.

The proto-cell (SSPCM-SRS-M) is growing on a minimal medium and is utilizing an unspecified substrate for biomass and energy synthesis. The substrate is transported into the proto-cell by transport protein (active transport) with *approximate* parameter values (Supplementary Tables 4, 10, 16). The transported substrate is converted to unspecified metabolic intermediates (building blocks) via the central metabolic pathway PW_1 that consists of a linear chain of reactions (with *generic* pathway length l_{PW1}). It is expected that reactions between intracellular metabolites have strict 1:1 stoichiometries. Building blocks and ATP are utilized to synthesize all 4 necessary monomers (amino acids, deoxyribonucleotides, ribonucleotides and lipids) in parallel biosynthesis pathways (PW_2-PW_5) consisting of linear reaction chains with *generic* pathway lengths l_{PW2}-l_{PW5} (Supplementary Table 5) respectively. Reactions on pathways PW_1-PW_5 are catalysed by identical enzymes with *generic* n_{enz} = 300 molecules (aa) enz^{-1} (Supplementary Table 10).

Translation processes of all proteins are carried out by ribosomes. Specific mRNA molecules are serving as matrices for translation processes, $N_{mrna\_cell\_comp}$ values are calculated based on P_{cell_comp} values (Supplementary Tables 22-23). Amino acids are delivered to ribosomes by *average* (in terms of n_{trna}) tRNA molecules (Supplementary Tables 10, 13).

Transcription is carried out by the RP complex. All 7 different mRNA molecules, universal tRNA and assembled rRNA complex are polymerized during the doubling time using *average* ribonucleotides (in terms of m_{nt}) (Supplementary Tables 16-17, 19) and ATP. The numbers of different RNA types are determined by the necessary translation rate.

Membrane lipids with *generic* parameter values (Supplementary Tables 16, 21) are polymerized by LPE with *generic* parameter values (Supplementary Tables 4, 10, 16) using

lipids and ATP. The amount of membrane lipids in the cell is determined by the available cell surface.
DNA replication starts from the oriC region of the circular chromosome by forming two parallel RC. DNA replication is taking place during *approximate* t_C compared to other polymerisation processes and therefore *approximate* N_{rc} does not correspond exactly to replication rate requirements reflected by N_{rce} if t_{d_srs-m} > *approximate* t_C.
ATP is produced by the ETC complex on the membrane and consumed by active transport, monomer synthesis and polymerization processes. It is assumed that energy production is not connected to metabolic pathways and there are no waste products.
It is assumed that the cell shape is an ideal cylinder with spherical caps. The inner surface area of the cell membrane equals the outer surface area to simplify the model. S_{tot} depends on V_{cyt} and S_{tot} is the sum of surface areas of different membrane components (S_{lip}, S_{stp}, S_{etc}). The volume of a cellular structure is derived as a sum of masses of all molecules of cell components (including water) localized in that cellular structure assuming that *generic* ρ_{tot} is similar to water density. M_{tot} can be divided roughly into two parts – M_{cyt} and M_{mem}.
The simplifications introduced to simplify the system of equations of the SSPCM-SRS-M (Supplementary Eqs. (73)-(125)) and also make the results intuitively more transparent. It is important to notice here also that despite of the simplifications the steady physiological states of SSPCM-SRS-M are quantitatively quite similar to the experimental results obtained studying *E. coli* cells.

Supplementary Table 2. Short overview of different types of cell components in a simplified single-cell model of the self-reproduction system of the abstract proto-cell growing on minimal medium (SSPCM-SRS-M). The table includes references to different types of cell processes and interactions corresponding to cell components. The table includes references to the main parameters of cell components used in SSPCM-SRS-M. Symbols of model input parameters are designated in bold, symbols of model output parameters are designated in regular.

Cell component type	Description	Reactions, interactions (Supplementary Table 3)	Parameters
DNA	Circular bihelix chromosome (*approximate* genome copy number in the cell (N_{dna}) and total dry weight content of DNA fraction in the cell ($DNA\%_{mmc}$)) which *specific, precise* length of deoxyribonucleotide sequence (n_{dna}) corresponds to the chromosome of *E. coli* K12 MG1655. Consists of *average* (in terms of the mass of polymerized molecule (m_{dnt})) deoxyribonucleotides linked together by phosphodiester bonds which determine the *approximate* mass of the genome (m_{dna}).	DNA replication.<br>Size of cytoplasm.	$\boldsymbol{n_{dna}}$ (Supplementary Table 10), $\boldsymbol{m_{dna}}$ (Supplementary Table 16), $\boldsymbol{m_{dnt}}$ (Supplementary Tables 16-17, 20), $\boldsymbol{N_{dna}}$ (Supplementary Table 24), $DNA\%_{mmc}$ (Supplementary Table 25)
RNA	The RNA fraction is composed of three main parts – ribosomal, transfer and messenger RNA. All RNA types consist of *average* (in terms of the mass of polymerized molecule (m_{nt})) ribonucleotides linked together by phosphodiester bonds.		$\boldsymbol{m_{nt}}$ (Supplementary Tables 16-17, 19)
tRNA	One universal (*average* ribonucleotide sequence length (n_{trna}) and *approximate* mass (m_{trna})) tRNA (the number of molecules in the cell (N_{trna})) is introduced as different amino acids (*generic* combined (various processes) effective working rate of tRNA (k_{trna})) are	Transcription.<br>Translation.<br>Size of cytoplasm.	$\boldsymbol{k_{trna}}$ (Supplementary Table 4), $\boldsymbol{n_{trna}}$ (Supplementary Tables 10, 13), $\boldsymbol{m_{trna}}$ (Supplementary Table 16), N_{trna} (Supplementary Table 25)

	not specified.		
rRNA	Ribosomal RNA complex (numbers in the cell (N_{rrna})) is assumed to be a monomeric sequence but the *specific, precise* sequence length (n_{rrna}) corresponded to three usual subunits (5S, 16S, 23S) of *E. coli* K12 MG1655 (*approximate* mass of rRNA complex (m_{rrna})) assembled in ribosomes with 1:1:1 stoichiometry (one copy of each subunit).	Transcription.<br>Translation.<br>Size of cytoplasm.	$\boldsymbol{n_{rrna}}$ (Supplementary Tables 10, 12), $\boldsymbol{m_{rrna}}$ (Supplementary Table 16), N_{rrna} (Supplementary Table 25)
mRNA	There are specific mRNAs (number of molecules in the cell ($N_{mrna\_cell\_comp}$)) coding each of the different proteins in the model. It is assumed that mRNAs contain only coding regions of which *approximate* or *generic* sequence lengths ($n_{mrna\_cell\_comp}$) and *approximate* or *generic* masses ($m_{mrna\_cell\_comp}$) correspond to amino acid sequences of corresponding proteins.	Transcription.<br>Translation.<br>Size of cytoplasm.	$\boldsymbol{n_{mrna\_cell\_comp}}$ (Supplementary Table 10), $\boldsymbol{m_{mrna\_cell\_comp}}$ (Supplementary Table 16), $N_{mrna\_cell\_comp}$ (Supplementary Table 25)
Proteins	Protein fraction is comprised of the following different proteins – enzymes in metabolic pathways PW_1-PW_5, ribosomal protein complexes (RPC), replisome complexes (RC), RNA polymerase (RP) complexes, transport proteins, electron transport chain (ETC) complexes, lipid synthesis enzymes (LPE). All proteins consist of *average* (in terms of the mass of polymerized molecule (m_{aa})) amino acids linked together by peptide bonds. All proteins are monomeric sequences for simplification purposes.		$\boldsymbol{m_{aa}}$ (Supplementary Tables 16-18)
Enzymes	All reactions in central and biosynthesis pathways	Synthesis of intracellular	$\boldsymbol{k_{enz}}$ (Supplementary Table 4),

	PW_1-PW_5 are catalysed by identical (*generic* amino acid sequence length (n_{enz}) = 300 molecules (aa) enz^{-1}, *generic* mass (m_{enz}), *generic* apparent working rate (k_{enz}) = 100 molecules (metabolite) s^{-1} enz^{-1}) enzymes (number of molecules per reaction in the cell ($N_{enz\_PWi\_r}$)).	metabolites and monomers (flux balances).<br>Energy balance (ATP costs for monomer synthesis).<br>Translation.<br>Size of cytoplasm.	$\boldsymbol{n_{enz}}$ (Supplementary Table 10), $\boldsymbol{m_{enz}}$ (Supplementary Table 16), $N_{enz\_PWi\_r}$ (Supplementary Table 25)
RPC	Stoichiometry and *approximate* amino acid sequence length (n_{rpc}) of RPC as a single monomeric polypeptide chain (number of complexes in the cell (N_{rs})) are based on data of *E. coli* K12 MG1655. *Approximate* mass (m_{rpc}) and *approximate* apparent working rate (k_{rs}) of the complex.	Amino acid flux balance.<br>Energy balance (ATP cost for protein synthesis).<br>Translation.<br>Size of cytoplasm.	$\boldsymbol{k_{rs}}$ (Supplementary Table 4), $\boldsymbol{n_{rpc}}$ (Supplementary Tables 10-11), $\boldsymbol{m_{rpc}}$ (Supplementary Table 16), N_{rs} (Supplementary Table 25)
RC	DNA replication starts from the oriC region of the circular chromosome by forming parallel replication complexes (*approximate* number of complexes in the cell (N_{rc})) with *approximate* amino acid sequence length (n_{rc}) based on data of different enzymes and proteins with fixed stoichiometries of *E. coli*. The *approximate* mass of RC (m_{rc}) and *approximate* apparent working rate of DNA polymerase III (k_{dp}). Molecular mechanisms of DNA replication initiation are not included in the models.	Deoxyribonucleotide flux balance.<br>Energy balance (ATP cost for DNA synthesis).<br>Translation (RC).<br>Size of cytoplasm.<br>DNA replication.	$\boldsymbol{k_{dp}}$ (Supplementary Table 4), $\boldsymbol{n_{rc}}$ (Supplementary Table 10, 15), $\boldsymbol{m_{rc}}$ (Supplementary Table 16), $\boldsymbol{N_{rc}}$ (Supplementary Table 24)
RP complex	RP complex is a monomeric polypeptide chain (number of complexes in the cell (N_{rp})) with *approximate* amino acid sequence length (n_{rp}) based on data of *E. coli*.	Ribonucleotide flux balance.<br>Energy balance (ATP cost	$\boldsymbol{k_{rp}}$ (Supplementary Table 4), $\boldsymbol{n_{rp}}$ (Supplementary Tables 10, 14), $\boldsymbol{m_{rp}}$ (Supplementary Table 16),

	Approximate mass (m_{rp}) and *approximate* apparent working rate (k_{rp}) of RP complex.	for RNA synthesis).<br>Translation (RP).<br>Size of cytoplasm.<br>Transcription.	N_{rp} (Supplementary Table 25)
Transport protein	The transporter for the unspecified substrate is a protein complex (number of molecules in the cell (N_{stp})) with *approximate* amino acid sequence length (n_{stp}), *approximate* mass (m_{stp}), *generic* apparent working rate (k_{stp}), the *approximate* surface area occupied by single molecule (s_{stp}) based on data of *E. coli*.	Central pathway flux balance.<br>Energy balance (ATP cost for substrate transport).<br>Translation (transport protein).<br>Size of the membrane.<br>Cell surface area.	$\boldsymbol{k_{stp}}$ (Supplementary Table 4), $\boldsymbol{n_{stp}}$ (Supplementary Table 10), $\boldsymbol{m_{stp}}$ (Supplementary Table 16), $\boldsymbol{s_{stp}}$ (Supplementary Table 21), N_{stp} (Supplementary Table 25)
ETC complex	ETC complex (number of complexes in the cell (N_{etc})) with *approximate* amino acid sequence length (n_{etc}), *approximate* mass (m_{etc}), *generic* apparent working rate (k_{etc}), the *approximate* surface area occupied by single molecule (s_{etc}) for the synthesis of ATP based on data of *E. coli*.	Energy balance (ATP synthesis).<br>Translation (ETC).<br>Size of the membrane.<br>Cell surface area.	$\boldsymbol{k_{etc}}$ (Supplementary Table 4), $\boldsymbol{n_{etc}}$ (Supplementary Table 10), $\boldsymbol{m_{etc}}$ (Supplementary Table 16), $\boldsymbol{s_{etc}}$ (Supplementary Table 21), N_{etc} (Supplementary Table 25)
LPE	*Generic* (amino acid sequence length (n_{lpe}), mass (m_{lpe}), apparent working rate (k_{lpe})) enzyme (number of molecules in the cell (N_{lpe})).	Lipid flux balance.<br>Energy balance (ATP cost for membrane lipid synthesis).<br>Translation (LPE).<br>Size of cytoplasm.<br>Membrane lipid synthesis.	$\boldsymbol{k_{lpe}}$ (Supplementary Table 4), $\boldsymbol{n_{lpe}}$ (Supplementary Table 10), $\boldsymbol{m_{lpe}}$ (Supplementary Table 16), N_{lpe} (Supplementary Table 25)
Membrane lipid	The simplified cell contains only one type of *generic*	Membrane lipid	$\boldsymbol{m_{lip}}$ (Supplementary Table 16),

	(mass (m_{lip}), surface area occupied by single molecule (s_{lip})) membrane lipid (number of molecules in the cell (N_{lip}) and total dry weight content of lipid fraction in the cell ($LIP\%_{mmc}$)) that forms bilayer cell membrane.	synthesis.<br>Cell surface area.<br>Size of the membrane.	$\boldsymbol{s_{lip}}$ (Supplementary Table 21), N_{lip} (Supplementary Table 25), $LIP\%_{mmc}$ (Supplementary Table 25)
Amino acid	*Average* (mass of polymerized amino acid molecule (m_{aa})) of 20 amino acids.	Composition of proteins.<br>Amino acid flux balance.<br>Translation.	$\boldsymbol{m_{aa}}$ (Supplementary Tables 16-18)
Deoxyribonucleotide	*Average* (mass of polymerized deoxyribonucleotide molecule (m_{dnt})) of 4 deoxyribonucleotides.	Composition of DNA.<br>Deoxyribonucleotide flux balance.<br>Replication.	$\boldsymbol{m_{dnt}}$ (Supplementary Table 16-17, 20)
Ribonucleotide	*Average* (mass of polymerized ribonucleotide molecule (m_{nt})) of 4 ribonucleotides.	Composition of RNA.<br>Ribonucleotide flux balance.<br>Transcription.	$\boldsymbol{m_{nt}}$ (Supplementary Tables 16-17, 19)
Intracellular metabolites and substrate	Unspecified molecules without specified structure and molecular characteristics.	Synthesis of intracellular metabolites and monomers (flux balances).	

Supplementary Table 3. Different types of cell processes and interactions between cell components of the simplified single-cell model of the self-reproduction system of the abstract proto-cell growing on minimal medium (SSPCM-SRS-M). The table includes references to corresponding equations in SSPCM-SRS-M. The table includes references to the main parameters of cell interactions used in SSPCM-SRS-M. Symbols of model input parameters are designated in bold, symbols of model output parameters are designated in regular.

Cell processes or interactions	Description	Cell component types involved (Supplementary Table 2)	Parameters

Metabolic network	The cell is growing on a minimal medium and is utilizing an unspecified substrate for biomass and energy synthesis. The substrate is transported into the cell by transport protein (active transport, *generic* energy cost of substrate transport (X_{stp})) (Supplementary Eq. (78)).<br>Subsequently, the substrate is converted to unspecified building blocks via a series of reactions and intracellular metabolites on central metabolic pathway PW_1 (*generic* pathway length (l_{PW1})) (flux balance equation of branching of the pathways – Supplementary Eqs. (77)-(78)).<br>Building blocks and ATP are further used by 4 parallel biosynthesis pathways (PW_2-PW_5) consisting of linear chains of reactions (*generic* pathway lengths (l_{PW2}-l_{PW5})) to produce *average* (in terms of m_{aa}) amino acids (corresponding flux of reaction r of amino acid synthesis pathway PW_2 ($F_{enz\_PW2\_r}$), *approximate* energy cost of amino acid synthesis (X_{PW2})), deoxyribonucleotides (*approximate* energy cost of deoxyribonucleotide synthesis (X_{PW3})), ribonucleotides (*approximate* energy cost of ribonucleotide synthesis (X_{PW4})) and lipids (*generic* energy cost of lipid synthesis (X_{PW5})) (Supplementary Eqs. (73)-(77)). Reactions on pathways PW_1-PW_5 are catalysed by identical enzymes with *generic* parameter values.	Transport protein.<br>Enzymes of metabolic pathways.<br>Intracellular metabolites and substrate.<br>Monomers.<br>ATP.	$F_{enz\_PWi\_r}$ (Supplementary Table 25), $\boldsymbol{l_{PWi}}$ (Supplementary Table 5), $\boldsymbol{X_{stp}}$ (Supplementary Table 9), $\boldsymbol{X_{PWi}}$ (Supplementary Table 6)
Protein synthesis	Proteins are synthesized during the doubling time of SSPCM-SRS-M (t_{d_srs-m}) by ribosomes (assemblies of ribosomal proteins and rRNA subunits, Supplementary Eq. (92)) using *average* (in terms of the mass of polymerized molecule (m_{aa})) amino acids	All 7 proteins.<br>All 9 RNAs.<br>Amino acid.<br>ATP.	$\boldsymbol{P_{cell\_comp}}$ (Supplementary Table 22-23), $\boldsymbol{X_{prot}}$ (Supplementary Table 9), $\boldsymbol{t_{d\_srs-m}}$ (Supplementary Table 24)

	and ATP (*approximate* energy cost of translation (X_{prot})) (Supplementary Eqs. (80), (82)-(88), (92)). Amino acids are delivered to ribosomes by *average* (in terms of ribonucleotide sequence length (n_{trna})) tRNA molecules (Supplementary Eq. (93)). Specific mRNA molecules are used for the translation process based on *generic* polysome densities (P_{cell_comp}) (Supplementary Eqs. (94)-(100)).		
RNA synthesis	Transcription is carried out by RNA polymerase (RP) complex. All 7 different mRNA molecules, single tRNA and assembled rRNA complex are polymerized during the doubling time using *average* (in terms of the mass of polymerized molecule (m_{nt})) ribonucleotides and ATP (*approximate* energy cost of transcription (X_{rna})) (Supplementary Eqs. (90)-(91)). The number of molecules/complexes of different RNAs (N_{rrna}, N_{trna}, $N_{mrna\_cell\_comp}$) are determined by the necessary translation rate (Supplementary Eqs. (92)-(100)).	All 9 RNAs.<br>RP complex.<br>Ribonucleotide.<br>ATP.	$\boldsymbol{X_{rna}}$ (Supplementary Table 9), $\boldsymbol{t_{d\_srs\text{-}m}}$ (Supplementary Table 24)
Lipid synthesis	Membrane lipids are polymerized by lipid synthesis enzymes (LPE) with *generic* parameter values using lipids and ATP (*approximate* energy cost of membrane lipid synthesis (X_{lip})) (Supplementary Eq. (101)).	Lipids.<br>LPE.<br>Membrane lipid.<br>ATP.	$\boldsymbol{X_{lip}}$ (Supplementary Table 9), $\boldsymbol{t_{d\_srs\text{-}m}}$ (Supplementary Table 24)
Energy balance	ATP is produced by the ETC complex on the membrane and produced ATP is consumed by active transport, monomer synthesis and polymerization reactions (Supplementary Eq. (102)).	ETC.<br>All polymerases.<br>Transport protein.<br>ATP.	
Cell geometry and mass	It is assumed that the cell shape is an ideal cylinder with spherical caps. The inner surface area of the cell membrane	All cell components except intracellular metabolites	M_{mem}, M_{cyt}, M_{tot}, S_{tot}, V_{cyt}, V_{tot} (Supplementary Table 26), $\boldsymbol{\rho_{tot}}$

	equals the outer surface area to simplify the model. Cell membrane surface area (S_{tot}) depends on the volume of cytoplasm (V_{cyt}) and S_{tot} is the sum of surface areas of cell membrane covered by different cell components (membrane lipids (S_{lip}), transporter proteins (S_{stp}), ETC (S_{etc})) (Supplementary Eq. (103)).<br>The volume of a cell or cytoplasm (V_{tot}, V_{cyt}) is derived as a sum of masses of all corresponding molecules (including water) (Supplementary Eqs. (106)-(107)) assuming that *generic* cell density (ρ_{tot}) is equal to water density (Supplementary Eq. (104)).<br>Total cell mass (M_{tot}) can be divided roughly into the following parts – a mass of cytoplasm (M_{cyt}) and cell membrane mass (M_{mem}) (Supplementary Eq. (105)).	and substrate.	(Supplementary Table 24)
DNA replication	DNA replication starts from the oriC region of the circular chromosome by replisome complexes (RC). DNA replication is carried out during *approximate* genome replication time (t_C) compared with other polymerisation processes and therefore the *approximate* number of RC (N_{rc}) does not correspond exactly to averaged replication rate (number of effective replisomes (N_{rce}), *approximate* energy cost of DNA replication (X_{dna})) if $t_{d\_srs\text{-}m}$ > *approximate* t_C (Supplementary Eq. (108)).	DNA.<br>Deoxyribonucleotide.<br>ATP.<br>RC.	N_{rce} (Supplementary Table 25), $\boldsymbol{t_C}$, $\boldsymbol{X_{dna}}$ (Supplementary Table 9), $\boldsymbol{t_{d\_srs\text{-}m}}$ (Supplementary Table 24)

Supplementary Discussion 5.11.2.1: Model components and interactions

Supplementary Discussion 5.11.2.1.1: Metabolic network

Proto-cells with complete bacterial SRS growing on minimal medium use unspecified growth substrate for the synthesis of metabolic intermediates (building blocks) which are converted to monomers (nucleotides, amino acid, lipid) of macromolecules (Figure 3, Supplementary Tables 2-3). Each monomer is synthesized from the same intermediate via a respective specific chain of subsequent unidirectional reactions (Figure 3). It is assumed that all reactions are similar to monomolecular isomerization reactions which have always strict 1:1 stoichiometry between reaction substrate and product. Each reaction chain forms a linear monomer synthesis pathway PW_i with *generic* length l_{PWi} for each monomer (amino acid synthesis pathway PW_2, deoxyribonucleotide synthesis pathway PW_3, ribonucleotide synthesis pathway PW_4, lipid synthesis pathway PW_5) (Supplementary Table 5).

All reactions are catalysed by identical (*generic* m_{enz}, *generic* n_{enz}) enzymes with the same *generic* k_{enz} (Supplementary Tables 4, 10, 16-18). It means that the $N_{enz\_PWi\_r}$ values are the same for each reaction in the same pathway PW_i because all reactions are carrying fluxes with identical sizes assuming identical enzymes and reaction stoichiometry. Therefore, Supplementary Eqs. (73)-(76) describe all fluxes of all reactions for all metabolic intermediates in respective metabolic pathways. This simplification enables to describe explicitly only one reaction flux balance in each pathway. All the monomers synthesized are subsequently polymerized by polymerases (ribosomes, RC, RP complex, LPE):

$$N_{enz\_PW2\_r} \cdot k_{enz} = N_{rs} \cdot k_{rs} \quad (73)$$

$$N_{enz\_PW3\_r} \cdot k_{enz} = 2 \cdot N_{rce} \cdot k_{dp} \quad (74)$$

$$N_{enz\_PW4\_r} \cdot k_{enz} = N_{rp} \cdot k_{rp} \quad (75)$$

$$N_{enz\_PW5\_r} \cdot k_{enz} = N_{lpe} \cdot k_{lpe} \quad (76)$$

The coefficient 2 in Supplementary Eq. (74) refers to two molecules of DNA polymerase III per RC (Supplementary Table 15).

Biosynthesis pathways PW_2-PW_5 are branching off from the central pathway PW_1 from the same intermediate (Figure 3, Supplementary Tables 2-3). Again, it is assumed that the topology of PW_1 is similar to those of biosynthesis pathways and that reactions of PW_1 are catalysed by identical enzymes (as in PW_2-PW_4). Therefore, the flux balances of the whole pathway are again described by the flux balance of one reaction. Parameter k_{enz} can be excluded from the flux balance equation due to identical enzymes for all pathways (Supplementary Eq. (77)).

$$N_{enz\_PW1\_r} = N_{enz\_PW2\_r} + N_{enz\_PW3\_r} + N_{enz\_PW4\_r} + N_{enz\_PW5\_r} \quad (77)$$

The unspecific growth substrate is imported into the cell by a transporter protein located on the cell membrane (Figure 3, Supplementary Tables 2-3). The total amount of consumed substrate must correspond to the biosynthesis requirements assuming that the simplified cell is utilizing substrate through the central metabolic pathway PW_1). Fluxes of PW_1 and flux of substrate consumption must be equal according to Supplementary Eq. (78):

$$N_{stp} \cdot k_{stp} = N_{enz\_PW1\_r} \cdot k_{enz} \quad (78)$$

Supplementary Discussion 5.11.2.1.2: Protein synthesis

The protein fraction of the proto-cell is comprised of the following different proteins (Supplementary Table 2):

1. RP complex (carries out transcription).
2. RPC (part of ribosomes, carries out translation).
3. Enzymes on biosynthetic pathways PW_1-PW_5 (carry out the synthesis of metabolic intermediates and monomers of macromolecules) (Figure 3).
4. LPE (membrane lipid synthesis).
5. ETC complex (energy production on the membrane).
6. Transport protein (substrate transport on the membrane).
7. RC (carries out DNA replication).

All proteins are assumed to be single molecules (monomeric polypeptide chain) without different subunits in the model for simplification purposes (enables to reduce the number of assembly processes and different mRNAs from the model) although RC, RP, RPC, ETC and transport proteins are actually assemblies of different proteins in living biological cells (see Supplementary Tables 10-11, 14-15). The numbers of all proteins are doubled during the doubling time by ribosomes carrying out translation. Different actual steps of translation and post-translational processes (protein maturation, folding, cofactor binding, complex formation etc.) are not described. Instead, there is a heavily simplified translation process which uses amino acids as substrates and produces proteins (Supplementary Table 3).

The balanced steady-state exponential growth of ribosomal protein self-replication was derived by[16] and is described here by following Supplementary Eq. (79) using designations of Equation 7 of[1]:

$$\bar{K} = \frac{\mu \cdot c}{\alpha} \tag{79}$$

Note that Supplementary Eq. (79) was further improved by introducing ribosome efficiency (Equation 10 of[48]). Although original derivations of Supplementary Eq. (79) and Equation 10 of[48] were based on ratios of translation rates, it is possible to express the relation between growth rate and translation based on fractions of ribosome numbers, proteome mass fractions etc which has led to additional (re)derivations/(re)discoveries with different designations and which have been collectively named as the ribosome allocation growth law[2,6,8,26-27,49-50].

In order to simplify the following analysis, the description of translation in SSPCM-SRS-M is based on assumption that all ribosomes are active which corresponds to the original Supplementary Eq. (79). The essence of Supplementary Eq. (79) is captured in SSPCM-SRS-M by following Supplementary Eq. (80). The number of RPC is doubled during the t_{d_srs} by RPC itself carrying out translation:

$$t_{d\_srs} = \frac{N_{rs} \cdot n_{rpc}}{N_{rs\_rpc} \cdot k_{rs}} \tag{80}$$

Compared to Supplementary Eq. (79), the following small modifications are made in Supplementary Eq. (80). It is assumed that the self-reproduction of ribosomes is carried out according to the linear growth law in a single proto-cell (Supplementary Discussion 5.11.1) instead of an exponentially growing population (doubling time $t_{d\_srs\text{-}m}$ instead of μ, omission of ln2). Molecular fractions are used instead of rate fractions as in[6,26]. Designations were changed for clarification purposes. The essence of α of Supplementary Eq. (79) is captured by the ratio of N_{rs_rpc}/N_{rs} in the current work, c of Supplementary Eq. (79) is identical to the n_{rpc} in the current work, $\bar{K}$ of Supplementary Eq. (79) is identical to k_{rs} in the current work. Note that translation descriptions for other proteins are basically similar to Supplementary Eq. (80).

The balanced steady-state exponential growth of RP synthesis was derived by[2] and is described here by following Supplementary Eq. (81) with original designations:

$$\mu = \frac{k_{ribo} \cdot \Phi_{ribo}^{RNAP} \cdot f_{ribo}^{active} \cdot N_{ribo}}{N_{RNAP} \cdot N_{RNAP}^{a.a.}} \quad (81)$$

In order to simplify the following analysis, the description of translation in SSPCM-SRS-M is based on assumption that all ribosomes are active. The essence of Supplementary Eq. (81) is captured in SSPCM-SRS-M by following Supplementary Eq. (82):

$$t_{d\_srs-m} = \frac{N_{rp} \cdot n_{rp}}{N_{rs\_rp} \cdot k_{rs}} \quad (82)$$

Compared to Supplementary Eq. (81), the following small modifications are made in Supplementary Eq. (82). It is assumed that the translation is carried out according to the linear growth law in a single proto-cell (Supplementary Discussion 5.11.1) instead of an exponentially growing population (t_{d_srs-m} instead of μ, omission of ln2). Designations were changed for clarification purposes. The essence of $\Phi_{ribo}^{RNAP} \cdot N_{ribo}$ of Supplementary Eq. (81) is captured by N_{rs_rp} in the current work, $N_{RNAP}^{a.a.}$ of Supplementary Eq. (81) is identical to the n_{rp} in the current work, k_{ribo} of Supplementary Eq. (81) is identical to k_{rs} in the current work, N_{RNAP} of Supplementary Eq. (81) is identical to N_{rp} in the current work.

$N_{rs\_cell\_comp}$ of remaining proteins are also determined by t_{d_srs-m} and the requirement of amino acids of each protein according to the following Supplementary Eqs. (83)-(87) which forms are very similar to Supplementary Eqs. (80), (82):

$$t_{d\_srs-m} = \frac{n_{enz} \cdot \sum_{i=1}^{5} N_{enz\_PWi\_r} \cdot l_{PWi}}{N_{rs\_enz} \cdot k_{rs}} \quad (83)$$

$$t_{d\_srs-m} = \frac{N_{lpe} \cdot n_{lpe}}{N_{rs\_lpe} \cdot k_{rs}} \quad (84)$$

$$t_{d\_srs-m} = \frac{N_{etc} \cdot n_{etc}}{N_{rs\_etc} \cdot k_{rs}} \quad (85)$$

$$t_{d\_srs-m} = \frac{N_{stp} \cdot n_{stp}}{N_{rs\_stp} \cdot k_{rs}} \quad (86)$$

$$t_{d\_srs-m} = \frac{N_{rc} \cdot n_{rc}}{N_{rs\_rc} \cdot k_{rs}} \quad (87)$$

N_{rs} is expressed by the following sum (Supplementary Eq. (88)):

$$N_{rs} = N_{rs\_rc} + N_{rs\_rp} + N_{rs\_rpc} + N_{rs\_etc} + N_{rs\_stp} + N_{rs\_lpe} + N_{rs\_enz} \quad (88)$$

Supplementary Discussion 5.11.2.1.3: RNA synthesis

All three main types of RNA molecules are synthesized in the described proto-cell model (Figure 3, Supplementary Table 2):

1. The parameters of the ribosomal RNA complex are based on 3 subunits (5S, 16S, 23S) assembled in ribosomes with 1:1:1 stoichiometry (one copy of each subunit) (Supplementary Table 12). Subunits of the complex are not described separately to simplify the model.
2. Transfer RNA. The model was simplified by introducing only one universal tRNA molecule because different amino acids are not specified (Supplementary Table 13). No amino acid specific aminoacyl tRNA synthetases are included to simplify the model.

3. There are specific mRNAs coding different proteins in the model. It is assumed that mRNAs contain only coding regions to simplify the model.

Numbers of all RNA molecules are doubled during the doubling time by RP complexes carrying out transcription. Different actual steps of transcription and post-transcriptional processes (RNA processing, modifications, charging, degradation etc.) are not described. Instead, there is a heavily simplified transcription process which uses ribonucleotides as substrates and produces RNA molecules (Supplementary Tables 2-3).

The balanced steady-state exponential growth of rRNA synthesis was derived by[2] and is described here by following Supplementary Eq. (89) with original designations:

$$\mu = \frac{k_{RNAP} \cdot \Phi_{RNAP}^{rRNA} \cdot f_{RNAP}^{active} \cdot N_{RNAP}}{N_{ribo} \cdot N_{ribo}^{nucl}} \tag{89}$$

In order to simplify the following analysis, the description of translation in SSPCM-SRS-M is based on assumption that all RP complexes are active. The essence of Supplementary Eq. (89) is captured in SSPCM-SRS-M by following Supplementary Eqs. (90)-(91):

$$t_{d\_srs-m} = \frac{N_{rrna} \cdot n_{rrna} + N_{trna} \cdot n_{trna} + \sum_{1}^{7} N_{mrna\_cell\_comp} \cdot n_{mrna\_cell\_comp}}{N_{rp} \cdot k_{rp}} \tag{90}$$

$$\sum_{1}^{7} N_{mrna\_cell\_comp} \cdot n_{mrna\_cell\_comp} = N_{mrna\_rc} \cdot n_{mrna\_rc} + N_{mrna\_rp} \cdot n_{mrna\_rp} + N_{mrna\_rpc} \cdot n_{mrna\_rpc} + \\ + N_{mrna\_etc} \cdot n_{mrna\_etc} + N_{mrna\_stp} \cdot n_{mrna\_stp} + N_{mrna\_lpe} \cdot n_{mrna\_lpe} + N_{mrna\_enz} \cdot n_{mrna\_enz} \tag{91}$$

Compared to Supplementary Eq. (89), the following modifications are made in Supplementary Eqs. (90)-(91). It is assumed that the transcription is carried out according to the linear growth law in SSPCM-SRS-M (Supplementary Discussion 5.11.1) instead of an exponentially growing population ($t_{d\_srs\text{-}m}$ instead of μ, omission of ln2). Also, the transcription of tRNA and all relevant mRNAs are included. Designations were changed for clarification purposes. N_{ribo}^{nucl} of Supplementary Eq. (89) is identical to the n_{rrna} in the current work, k_{RNAP} of Supplementary Eq. (89) is identical to k_{rp} in the current work.

Considering relatively conserved structure[51] and assuming fixed stoichiometry of ribosome subunits (rRNA complex and RPC) instead of possible variations[52], it is possible to compose the following Supplementary Eq. (92). As mentioned before, the assembly processes of ribosomes are not taken into account and described to simplify the model.

$$N_{rrna} = N_{rs} \tag{92}$$

N_{trna} is determined by the amino acid requirement and translational activity of the proto-cell described by following flux balance (Supplementary Eq. (93)). Different reactions and steps of translation involving tRNA in living cells are not described explicitly in the model.

$$N_{trna} \cdot k_{trna} = N_{rs} \cdot k_{rs} \tag{93}$$

$N_{mrna\_cell\_comp}$ values are determined by translational activity and also by P_{cell_comp} (Supplementary Eqs. (94)-(100), Supplementary Table 22).

$$N_{mrna\_rc} = N_{rs\_rc} \cdot P_{rc} \tag{94}$$

$$N_{mrna\_rp} = N_{rs\_rp} \cdot P_{rp} \tag{95}$$

$$N_{mrna\_rpc} = N_{rs\_rpc} \cdot P_{rpc} \tag{96}$$

$$N_{mrna\_etc} = N_{rs\_etc} \cdot P_{etc} \tag{97}$$

$$N_{mrna\_stp} = N_{rs\_stp} \cdot P_{stp} \tag{98}$$

$$N_{mrna\_lpe} = N_{rs\_lpe} \cdot P_{lpe} \tag{99}$$

$$N_{mrna\_enz} = N_{rs\_enz} \cdot P_{enz} \tag{100}$$

Supplementary Discussion 5.11.2.1.4: Lipid synthesis

Proto-cell contains only one type of membrane lipid that forms a bilayer cell membrane (Supplementary Tables 1-2). Different residues of membrane lipid molecule are not specified. The value of N_{lip} is doubled during the doubling time by LPE (Supplementary Table 3) with *generic* parameters according to the following Supplementary Eq. (101):

$$t_{d\_srs-m} = \frac{N_{lip}}{N_{lpe} \cdot k_{lpe}} \tag{101}$$

Supplementary Discussion 5.11.2.1.5: Energy balance

ETC on the cell membrane (Supplementary Table 1) must produce the exact amount of ATP that is consumed by different monomer biosynthesis pathways, polymerization processes and substrate transport (Figure 3, Supplementary Tables 2-3) to accomplish the doubling condition (Supplementary Eq. (102)). The exact mechanism of ATP production (electron transfer from the substrate) is not specified to simplify the model.

$$N_{etc} \cdot k_{etc} = N_{stp} \cdot k_{stp} \cdot X_{stp} + 2 \cdot N_{rce} \cdot k_{kdp} \cdot X_{dna} + N_{rp} \cdot k_{rp} \cdot X_{rna} + N_{lpe} \cdot k_{lpe} \cdot X_{lip} + \\ + N_{rs} \cdot k_{rs} \cdot X_{prot} + k_{enz} \cdot \sum_{2}^{5} N_{enz\_PWi\_r} \cdot X_{PWi} \tag{102}$$

Supplementary Discussion 5.11.2.1.6: Cell geometry

It is assumed that the proto-cell shape is an ideal cylinder with spherical caps (Figure 3, Supplementary Table 3) which resembles the idealized shape of an *E. coli* cell[47]. The inner surface area of the cell membrane equals the outer surface area to simplify the model. S_{tot} depends on V_{cyt} and S_{tot} is the sum of surface areas of different membrane components (S_{lip}, S_{stp}, S_{etc}) according to Supplementary Eq. (103):

$$N_{lip} \cdot s_{lip} + N_{etc} \cdot s_{etc} + N_{stp} \cdot s_{stp} = \left(\frac{V_{cyt}}{\frac{4}{3} + HR} \right)^{\frac{2}{3}} \cdot (4 + 2 \cdot HR) \cdot \pi^{\frac{1}{3}} \tag{103}$$

Note that whereas ETC and transport proteins are needed also for energy production and substrate transport on the membrane, the N_{lip} is solely determined by the reminding surface area. Therefore, the surface area of lipids starts to decrease during growth increase which might determine the growth boundary (t_{d_min}).

V_{cyt} is equal to the M_{cyt} assuming that the density of cytoplasm is identical to *generic* ρ_{tot} and equal to water density Supplementary Eq. (104).

$$V_{cyt} = \frac{M_{cyt}}{\rho_{tot}} \tag{104}$$

Supplementary Discussion 5.11.2.1.7: Mass balance

M_{tot} (Supplementary Table 3) can be divided roughly into two parts by cell structures (Supplementary Table 1) – M_{cyt} and M_{mem} – and it is the sum of masses of all cell components including water (mass of water is described by *approximate DWC*) (Supplementary Eq. (105)):

$$M_{tot} = M_{cyt} + \frac{N_{lip} \cdot m_{lip} + N_{stp} \cdot m_{stp} + N_{etc} \cdot m_{etc}}{DWC} \tag{105}$$

M_{cyt} equals the sum of masses of all cell components (including water) localized in cytoplasmic space:

$$M_{cyt} = \frac{N_{rc} \cdot m_{rc} + N_{rp} \cdot m_{rp} + N_{rs} \cdot m_{rpc} + N_{lpe} \cdot m_{lpe} + m_{enz} \cdot \sum_{i=1}^{5} N_{enz\_PWi\_r} \cdot l_{PWi} + N_{rrna} \cdot m_{rrna}}{DWC} +$$
$$+ \frac{N_{trna} \cdot m_{trna} + \sum_{1}^{7} N_{mrna\_cell\_comp} \cdot m_{mrna\_cell\_comp} + N_{dna} \cdot m_{dna}}{DWC} \tag{106}$$

$$\sum_{1}^{7} N_{mrna\_cell\_comp} \cdot m_{mrna\_cell\_comp} = N_{mrna\_rc} \cdot m_{mrna\_rc} + N_{mrna\_rp} \cdot m_{mrna\_rp} + N_{mrna\_rpc} \cdot m_{mrna\_rpc} +$$
$$+ N_{mrna\_etc} \cdot m_{mrna\_etc} + N_{mrna\_stp} \cdot m_{mrna\_stp} + N_{mrna\_lpe} \cdot m_{mrna\_lpe} + N_{mrna\_enz} \cdot m_{mrna\_enz} \tag{107}$$

Supplementary Discussion 5.11.2.1.8: DNA synthesis

Molecular mechanisms of DNA replication initiation are not included in the current model. DNA replication starts from the oriC region of the circular chromosome by forming two parallel RC (see Supplementary Tables 2-3). Parameter values of RC are based on different enzymes and proteins with fixed stoichiometries (Supplementary Table 15). Individual enzymes of RC are not described explicitly in the model. The replication of a genome requires two RC for both directions of the circular chromosome and each RC contains in turn two active DNA polymerase III molecules (bidirectional replication of leading and lagging strands) that are all working in parallel[53]. DNA replication is a periodical process during the cell cycle (takes place during t_C) compared with other polymerisation processes and therefore *approximate* N_{rc} (Supplementary Table 24) does not correspond exactly to total replication requirements if $t_{d\_srs\text{-}m}$ > *approximate* t_C (Supplementary Table 24). To optimize and simplify the model, N_{rce} was introduced and it corresponds to the number of effective RC that is necessary for the replication process averaged over $t_{d\_srs\text{-}m}$ (takes place continuously) (Supplementary Eq. (108)).

$$t_{d\_srs-m} = \frac{N_{dna} \cdot n_{dna}}{2 \cdot N_{rce} \cdot k_{dp}} \tag{108}$$

Supplementary Discussion 5.11.2.2: Model parameters

Cell parameters used in the equations and the values of input parameters in the model are described and presented in this chapter.

The parameters are divided into input (predetermined) and output (calculated using the system of equations) parameters. The number of independent model cell parameters is approximately 1/3 larger than the number of independent equations of the SSPCM-SRS-M which means that a smaller part of the parameters must be fixed (input model parameters) to get a determined system with a unique solution (Supplementary Discussion 5.11.2.3). Input parameters for solving models were chosen mostly among those cell parameters (dimensions and compositions of molecules (m_{cell_comp} (Supplementary Table 16), n_{cell_comp} (Supplementary Table 10), s_{cell_comp} (Supplementary Table 21)), stoichiometries of reactions and cellular processes (X_{cell_comp}) (Supplementary Tables 6, 9), ρ_{tot} (Supplementary Table 24), apparent working rates of polymerases (k_{pol}) (Supplementary Table 4) etc.) that have constant or relatively unchanging values for a given strain in a given environment. Input parameter values were taken from literature and databases (sequence lengths of nucleic acids and polymerases) if possible in order to provide familiar context for the readers. These values (labels *specific*, *precise*, *average* and *approximate*) correspond to the most thoroughly studied bacterium, *Escherichia coli* (strain K-12 MG1655). In case of lack of information about input parameter values, the input parameters have *generic* (k_{enz} (Supplementary Table 4), P_{cell_comp} (Supplementary Tables 22-23) etc.) values or they are variables (t_{d_srs-m}) (Supplementary Table 24).

Other physiological parameters (dependent cellular, molecular and growth parameters) determining partially the steady physiological state of the growing proto-cells, and calculable from the model (output parameters of the model) were chosen among those cell parameters that lack respective databases and have nonstatic nature (NB! depend on genotype and growth conditions): N_{cell_comp}, F_{cell_comp}, cell compositions ($cell\_comp\%_{mmc}$) (Supplementary Table 25), geometric dimensions and size of a cell (Supplementary Table 26).

It should be stressed that the current selection of input and output parameters is not predetermined and unique, i.e., the only possible. If experimentally determined values of cell parameters (for example N_{rs}, $F_{enz\_PWi\_r}$, $LIP\%_{mmc}$ etc) would be available, then it is possible to use them as input parameters and specify the values of other cell parameters (for example k_{enz}, N_{stp} etc). Current models are simple enough to avoid most difficulties in solving potentially mathematically inefficient (certain selections of input parameters) models.

Supplementary Discussion 5.11.2.2.1: Input parameters

Values of input parameters (k_{cell_comp}, X_{cell_comp}, dimensions (m_{cell_comp}, $s_{\_cell\_comp}$) of molecules, n_{cell_comp}, P_{cell_comp}, cell shape (HR)) are presented in Supplementary Tables 4-24 below.

Supplementary Table 4. Values of apparent working rates of catalysing cell components (k_{cell_comp}).

Catalysing cell component [4.1]	Symbol	Value	Term	Unit
RNA polymerase complex [4.2]	k_{rp}	40	*approximate*	molecules (nt) s^{-1} rp^{-1}
Ribosome [4.3]	k_{rs}	20	*approximate*	molecules (aa) s^{-1} rs^{-1}
Electron transport chain complex	k_{etc}	100	*generic*	molecules (atp) s^{-1} etc^{-1}
Lipid synthesis enzyme	k_{lpe}	100	*generic*	molecules (lip) s^{-1} lpe^{-1}
Replisome complex [4.4]	k_{dp}	10^3	*approximate*	molecules (dnt) s^{-1} $(dp)^{-1}$
Transporter protein	k_{stp}	100	*generic*	molecules (substrate) s^{-1} stp^{-1}
tRNA [4.5]	k_{trna}	4	*approximate*	molecules (aa) s^{-1} $trna^{-1}$

Enzyme of metabolic pathways PW_1-PW_5	k_{enz}	100	*generic*	molecules (metabolite) s^{-1} enz^{-1}

4.1 The apparent working rates of most of the enzymes (except polymerases) can change considerably and depend on many factors like growth conditions or kinetic mechanisms. Another problem is that experimental enzymology studies do not necessarily correspond to *in vivo* conditions and therefore measured values are far from realistic values. It was assumed in the current model that k_{enz}, k_{lpe}, k_{stp} and k_{etc} have *generic* values (100 molecules (metabolite) s^{-1} $prot^{-1}$).
4.2 Various values ranging from 30 to 90 molecules (nt) s^{-1} rp^{-1} have been reported in the literature depending on cultivation conditions, used strains and RNA types[54,55]. It is assumed in the current model that *approximate* k_{rp} corresponds to medium mRNA and rRNA elongation rates.
4.3 Polypeptide elongation rate values are between 10 – 20 molecules (aa) s^{-1} rs^{-1} depending on several factors according to the literature[56].
4.4 DNA replication rate is approximately 600 – 10^3 molecules (bp) s^{-1} rc^{-1} [57] or 600 – 10^3 molecules (dnt) s^{-1} dp^{-1}.
4.5 Values correspond to the usual RNA fraction composition (80 % of rRNA, 15 % of tRNA and 5 % of mRNA)[58].

Supplementary Table 5. Numbers of reactions in metabolic pathways (pathway lengths) of the metabolic network (l_{PWi}).

**Pathway** 5.1	**Symbol**	**Value**	**Term**	**Unit**
Central metabolic pathway PW_1	l_{PW1}	200	*generic*	reactions PW_1^{-1}
Amino acid synthesis pathway PW_2	l_{PW2}	200	*generic*	reactions PW_2^{-1}
Deoxyribonucleotide synthesis pathway PW_3	l_{PW3}	200	*generic*	reactions PW_3^{-1}
Ribonucleotide synthesis pathway PW_4	l_{PW4}	200	*generic*	reactions PW_4^{-1}
Lipid synthesis pathway PW_5	l_{PW5}	200	*generic*	reactions PW_5^{-1}

5.1 The *generic* number of reactions per pathway is the same for all pathways PW_1-PW_5. The sum of selected numbers is comparable to 1050 different kinds of proteins in Table 1 of[58].

Supplementary Table 6. Energy costs of monomer synthesis (X_{cell_comp}).

**Monomer** 6.1	**Symbol**	**Value**	**Term**	**Unit**
Amino acid	X_{PW2}	1.434	*approximate*	molecules (atp) aa^{-1}
Deoxyribonucleotide	X_{PW3}	10.878	*approximate*	molecules (atp) dnt^{-1}
Ribonucleotide	X_{PW4}	10.381	*approximate*	molecules (atp) nt^{-1}
Lipid 6.2	X_{PW5}	10	*generic*	molecules (atp) $lipid^{-1}$

6.1 Energy costs for biosynthesis were calculated based on the data from Table 1 in[59] by finding average energy costs for the synthesis of each monomer type (amino acid, deoxyribonucleotide, ribonucleotide) weighted by monomer composition of each macromolecular fraction (protein, DNA, RNA). The values of stoichiometric energy cost coefficients of specific monomer synthesis pathways (fourth column in Table 1 of[59]) and the values of specific monomer contents in biomass (second column in Table 1 of[59]) were

multiplied to get the biosynthesis energy costs for all twenty amino acids, four deoxyribonucleotides and four ribonucleotides (Supplementary Table 7).

Supplementary Table 7. Biosynthesis energy costs for monomers (amino acids, deoxyribonucleotides, ribonucleotides) based on monomer composition of macromolecular fractions (protein, DNA, RNA) and stoichiometric energy cost coefficients of monomer synthesis pathways in Table 1 of[59].

Monomer	Energy cost (μmol (atp) (g (dw cell))$^{-1}$)
Alanine	0
Arginine	1967
Asparagine	687
Aspartate	0
Cysteine	348
Glutamate	0
Glutamine	250
Glycine	0
Histidine	540
Isoleucine	552
Leucine	0
Lysine	652
Methionine	1022
Phenylalanine	176
Proline	210
Serine	0
Threonine	482
Tryptophan	270
Tyrosine	131
Valine	0
ATP	1815
GTP	2639
CTP	1134
UTP	952
dATP	271.7
dGTP	330.2
dCTP	228.6
dTTP	259.35

Latter values were summed to get the biosynthesis energy costs for macromolecular fractions (protein amino acids, RNA ribonucleotides, DNA deoxyribonucleotides) of biomass (Supplementary Table 8). Specific monomer contents in biomass (second column in Table 1 of[59]) were summed by a macromolecular fraction to get the contents of corresponding fractions (Supplementary Table 8). Finally, calculated biosynthesis energy costs for macromolecular fractions (protein amino acids, RNA ribonucleotides, DNA

deoxyribonucleotides) of biomass were divided by the contents of corresponding macromolecular fractions.

Supplementary Table 8. Contents and biosynthesis energy costs of macromolecular fractions (protein amino acids, RNA ribonucleotides, DNA deoxyribonucleotides) of biomass based on Table 1 of[59].

Macromolecular fraction	Energy cost (μmol (atp) (g (dw cell))$^{-1}$)	Content (μmol (mon) (g (dw cell))$^{-1}$)
Amino acids	7287	5081
Ribonucleotides	6540	630
Deoxyribonucleotides	1089.85	100.2

[6.2] The *generic* value of X_{PW5} is comparable to the synthesis costs of docosanoic acid (C22:0).

Supplementary Table 9. Energy costs of polymerization and transport (X_{cell_comp}).

Cell process [9.1]	Symbol	Value	Term	Unit
DNA replication	X_{dna}	1.372	*approximate*	molecules (atp) dnt^{-1}
Transcription	X_{rna}	0.4	*approximate*	molecules (atp) nt^{-1}
Translation	X_{prot}	4.306	*approximate*	molecules (atp) aa^{-1}
Lipid synthesis	X_{lip}	0.5	*approximate*	molecules (atp) lip^{-1}
Transport [9.2]	X_{stp}	0.5	*generic*	molecules (atp) substrate^{-1}

[9.1] Energy requirements for polymerization processes (replication, transcription, translation, lipid synthesis) were approximated from the data of[60,61].
[9.2] Transport costs correspond to *generic* costs of proton-symport electroneutral transport mechanisms[62] assuming that all protons used in the transport are secreted out of the cells to maintain intracellular pH and to form proton gradient assuming maximal H^+/ATP ratio 2 for prokaryotes[63].

Supplementary Table 10. The number of molecules of monomer in (monomeric sequence lengths of) macromolecular cell component molecules/complexes (n_{cell_comp}).

Cell component [10.1]	Symbol	Value	Term	Unit
DNA [10.2]	n_{dna}	4639675	*specific, precise*	molecules (bp) genome^{-1}
Ribosomal protein complex (RPC) [10.3]	n_{rpc}	7242	*approximate*	molecules (aa) rpc^{-1}
rRNA complex [10.4]	n_{rrna}	4567	*specific, precise*	molecules (nt) rrna^{-1}
tRNA [10.5]	n_{trna}	77	*average*	molecules (nt) trna^{-1}
RNA polymerase (RP) complex [10.6]	n_{rp}	5574	*approximate*	molecules (aa) rp^{-1}
Replisome complex (RC) [10.7]	n_{rc}	48882	*approximate*	molecules (aa) rc^{-1}

Lipid synthesis enzyme (LPE)	n_{lpe}	300	*generic*	molecules (aa) lpe^{-1}
Transport protein [10.8]	n_{stp}	$2 \cdot 10^3$	*approximate*	molecules (aa) stp^{-1}
Electron transport chain (ETC) complex [10.9]	n_{etc}	10^4	*approximate*	molecules (aa) etc^{-1}
Enzyme of metabolic pathways PW_1-PW_5	n_{enz}	300	*generic*	molecules (aa) enz^{-1}
mRNA of RC [10.10]	n_{mrna_rc}	146646	*approximate*	molecules (nt) $(mrna\ rc)^{-1}$
mRNA of RP complex [10.10]	n_{mrna_rp}	16722	*approximate*	molecules (nt) $(mrna\ rp)^{-1}$
mRNA of LPE [10.10]	n_{mrna_lpe}	900	*generic*	molecules (nt) $(mrna\ lpe)^{-1}$
mRNA of RPC [10.10]	n_{mrna_rpc}	21726	*approximate*	molecules (nt) $(mrna\ rpc)^{-1}$
mRNA of transport protein [10.10]	n_{mrna_stp}	$6 \cdot 10^3$	*approximate*	molecules (nt) $(mrna\ stp)^{-1}$
mRNA of ETC complex [10.10]	n_{mrna_etc}	$3 \cdot 10^4$	*approximate*	molecules (nt) $(mrna\ etc)^{-1}$
mRNA of the enzyme of metabolic pathways PW_1-PW_5 [10.10]	n_{mrna_enz}	900	*generic*	molecules (nt) $(mrna\ enz)^{-1}$

[10.1] Values of *specific, precise* n_{rrna}, *average* n_{trna}, *approximate* n_{rpc}, n_{rp}, and n_{rc} (partially) are based on data of *Escherichia coli* K-12 MG1655 from KEGG database[64].

[10.2] Value of *specific, precise* n_{dna} was taken from[65].

[10.3] The value of *approximate* n_{rpc} is comparable to the respective value of *E. coli* K-12 MG1655 based on[51,66] and corresponds to the following RPC composition based on data of *E. coli* K-12 MG1655 from KEGG (Supplementary Table 11).

Supplementary Table 11. Composition of ribosomal protein complex (RPC) of *E. coli* K-12 MG1655 corresponding to *approximate* n_{rpc}.

Ribosomal protein [11.1]	Gene	KEGG identifier	Stoichiometry of the complex	Total number of amino acids in RPC complex (molecules (aa) rpc^{-1})
L1	rplA	b3984	1	234
L2	rplB	b3317	1	273
L3	rplC	b3320	1	209
L4	rplD	b3319	1	201
L5	rplE	b3308	1	179
L6	rplF	b3305	1	177
L9	rplI	b4203	1	149
L10	rplJ	b3985	1	165
L11	rplK	b3983	1	142
L12	rplL	b3986	2 [11.2]	242
L13	rplM	b3231	1	142
L14	rplN	b3310	1	123
L15	rplO	b3301	1	144
L16	rplP	b3313	1	136
L17	rplQ	b3294	1	127
L18	rplR	b3304	1	117
L19	rplS	b2606	1	115
L20	rplT	b1716	1	118
L21	rplU	b3186	1	103
L22	rplV	b3315	1	110
L23	rplW	b3318	1	100
L24	rplX	b3309	1	104
L27	rpmA	b3185	1	85

L28	rpmB	b3637	1	78
L29	rpmC	b3312	1	63
L30	rpmD	b3302	1	59
L31	rpmE	b3936	1	70
L32	rpmF	b1089	1	57
L33	rpmG	b3636	1	55
L34	rpmH	b3703	1	46
L35	rpmI	b1717	1	65
L36	rpmJ	b3299	1	38
S1	rpsA	b0911	1	557
S2	rpsB	b0169	1	241
S3	rpsC	b3314	1	233
S4	rpsD	b3296	1	206
S5	rpsE	b3303	1	167
S6	rpsF	b4200	1	131
S7	rpsG	b3341	1	179
S8	rpsH	b3306	1	130
S9	rpsI	b3230	1	130
S10	rpsJ	b3321	1	103
S11	rpsK	b3297	1	129
S12	rpsL	b3342	1	124
S13	rpsM	b3298	1	118
S14	rpsN	b3307	1	101
S15	rpsO	b3165	1	89
S16	rpsP	b2609	1	82
S17	rpsQ	b3311	1	84
S18	rpsR	b4202	1	75

S19	rpsS	b3316	1	92
S20	rpsT	b0023	2 [11.3]	174
S21	rpsU	b3065	1	71
Sum				7242

[11.1] The value of *approximate* n_{rpc} is smaller than the respective value of *E. coli* K-12 MG1655 assuming stoichiometry in[51,66] and excluding possible stoichiometry variations[52]. The difference includes, for example, amino acid sequence lengths of 2 L12, L25 and nonessential S22[67] proteins.
[11.2] There are 4 copies of L7/L12 proteins organized as 2 homodimers in the single ribosomal stalk of *E. coli* cells[51]. Currently, the value of *approximate* n_{rpc} does not cover the amino acid sequence lengths of all ribosomal proteins.
[11.3] The stoichiometry coefficient 2 refers to L26 protein which is identical to S20[66].

[10.4] Value of *specific, precise* n_{rrna} was calculated based on the following data of *E. coli* K-12 MG1655 from KEGG (Supplementary Table 12).

Supplementary Table 12. Composition of rRNA complex of *E. coli* K-12 MG1655.

rRNA subunits	Gene	KEGG identifier	Stoichiometry of the complex	Total number of nucleotides in rRNA complex (molecules (nt) rrna^{-1})
5S	rrfA [12.1]	b3855	1	120
16S	rrsA [12.2]	b3851	1	1542
23S	rrlA [12.3]	b3854	1	2905
Sum				4567

[12.1] There are altogether 8 different paralogous genes organized in 7 rRNA operons that code 5S rRNA in the genome of *E. coli* K-12 MG1655[65]. Although there are minor differences in corresponding sequences, the total length is identical for all genes.
[12.2] There are altogether 7 different paralogous genes organized in 7 rRNA operons that code 16S rRNA in the genome of *E. coli* K-12 MG1655[65]. Although there are minor differences in corresponding sequences, the total length is identical for all genes.
[12.3] There are altogether 7 different paralogous genes organized in 7 rRNA operons that code 23S rRNA in the genome of *E. coli* K-12 MG1655[65]. Although there are minor differences in corresponding sequences, the total length is almost identical for all genes differing only by 1 nt (the length of the remaining 6 genes is 2904 molecules (dnt) gene^{-1}). It was decided that such difference was negligible for not to use the term *specific, precise*.

[10.5] It is assumed that there is only one type of universal tRNA which *average* n_{trna} is the average length of ribonucleotide sequences of all different tRNAs of *E. coli* K-12 MG1655 from KEGG (Supplementary Table 13).

Supplementary Table 13. tRNA molecules of *E. coli* K-12 MG1655.

tRNA	Gene	KEGG identifier	Number of nucleotides (molecules (nt) trna^{-1})
tRNA of alanine	alaV [13.1]	b0203	76
tRNA of arginine	argU [13.2]	b0536	77
tRNA of asparagine	asnT [13.1]	b1977	76
tRNA of aspartate	aspT [13.1]	b3760	77
tRNA of cysteine	cysT	b1910	74
tRNA of glutamine	glnV [13.1]	b0665	75
tRNA of glutamate	gltU [13.1]	b3757	76
tRNA of glycine	glyT [13.2]	b3978	75
tRNA of histidine	hisR	b3797	77
tRNA of isoleucine	ileV [13.1]	b0202	77

tRNA of leucine	leuW [13.2]	b0672	85
tRNA of lysine	lysY [13.1]	b0747	76
tRNA of methionine	metT [13.2]	b0673	77
tRNA of phenylalanine	pheU [13.1]	b4134	76
tRNA of proline	proL [13.1]	b2189	77
tRNA of serine	serW [13.2]	b0883	88
tRNA of threonine	thrV [13.1]	b3273	76
tRNA of tryptophan	trpT	b3761	76
tRNA of tyrosine	tyrU [13.1]	b3977	85
tRNA of valine	valZ [13.2]	b0746	76
Average			77.6

[13.1] There is a number of different paralogous genes that code several tRNAs of *E. coli* K-12 MG1655[65]. Although there are minor differences between sequences, the lengths of all paralogous genes (belonging to the same amino acid tRNA) are identical for certain tRNAs (2 genes for tRNA of phenylalanine; 3 genes for tRNA of aspartate, isoleucine, proline and tyrosine; 4 genes for tRNA of asparagine, glutamine, glutamate and threonine; 5 genes for tRNA of alanine; 6 genes for tRNA of lysine).
[13.2] In the case of some tRNAs, the lengths of paralogous genes (belonging to the same amino acid tRNA) differ by 1-5 nt (77 molecules (dnt) gene^{-1} for 6 genes and 75 molecules (dnt) gene^{-1} for 1 gene of tRNA of arginine; 76 molecules (dnt) gene^{-1} for 4 genes, 74 and 75 molecules (dnt) gene^{-1} for 1 gene of tRNA of glycine; 87 molecules (dnt) gene^{-1} for 6 genes and 85 molecules (dnt) gene^{-1} for 2 genes of tRNA of leucine; 77 molecules (dnt) gene^{-1} for 6 genes and 76 molecules (dnt) gene^{-1} for 2 genes of tRNA of methionine; 88 molecules (dnt) gene^{-1} for 3 genes, 90 and 93 molecules (dnt) gene^{-1} for 1 gene of tRNA of serine; 76 molecules (dnt) gene^{-1} for 5 genes and 77 molecules (dnt) gene^{-1} for 2 genes of tRNA of valine). It was decided that such differences were negligible for the term *approximate*.

[10.6] The value of *approximate* n_{rp} was calculated from KEGG based on the following stoichiometry of the complex of *E. coli* K-12 MG1655[68] and assuming that the complex involves the most common sigma factor (Supplementary Table 14).

Supplementary Table 14. Composition of RNA polymerase II of *E. coli* K-12 MG1655.

**RNA Pol II subunits**	**Gene**	**KEGG identifier**	**Stoichiometry of the complex**	**Total number of amino acids in the complex (molecules (aa) rp^{-1})**
Core α	rpoA	b3295	2	658
Core β	rpoB	b3987	1	1342
Core β'	rpoC	b3988	1	1407
ω (ppGpp binding)	rpoZ	b3649	1	91
Sigma factor (σ70)	rpoD	b3067	1	613

post-termination factor	rapA	b0059	1	968
termination factor L	nusA	b3169	1	495
Sum				5574

[10.7] The value of *approximate* n_{rc} was calculated from KEGG based on the following stoichiometry of *E. coli* from different literature sources (Supplementary Table 15).

Supplementary Table 15. Composition of replisome complex (RC) of *E. coli*.

RC subunits	Gene	KEGG identifier	Stoichiometry of the complex	Total number of amino acids in the complex (molecules (aa) rc^{-1})	References
DNA Pol III core (α)	dnaE	b0184	3	3480	[53]
DNA Pol III core (ε) (exonuclease)	dnaQ	b0215	3	729	[53]
DNA Pol III core (θ) (stimulatory)	holE	b1842	3	228	[69]
DNA Pol III (β) (sliding clamp)	dnaN	b3701	6	2196	[53]
DNA Pol III (τ) (clamp loader)	dnaX	b0470	3	1929	[53]
dnaZ (γ)	dnaX [15.1]	b0470	3	1434	[69,70]
DNA Pol III preinitiation complex (δ) ("wrench" protein)	holA	b0640	1	343	[53]
DNA Pol III preinitiation complex (δ') (rigid protein)	holB	b1099	1	334	[53,69]
DNA Pol III (χ) (links τ to ssb)	holC	b4259	4	588	[69,53]
DNA Pol III (ψ) (stabilising protein)	holD	b4372	4	548	[69,53]
Helicase	dnaB	b4052	6	2826	[53]
ssDNA binding protein	ssb	b4059	32	5696	[53]
DNA Pol I	polA	b3863	1	928	[71,72]
DNA gyrase A	gyrA	b2231	2	1750	[73]
DNA gyrase B	gyrB	b3699	2	1608	[73]
DNA topoisomerase IV A	parC	b3019	4	3008	[73]

DNA topoisomerase IV B	parE	b3030	4	2520	[73]
Chromosomal replication initiator protein	dnaA	b3702	30	14010	[74]
DNA replication protein	dnaC	b4361	6	1470	[75]
DNA primase	dnaG	b3066	1	581	[53,69]
ssDNA binding factor	priA	b3935	1	732	[76,77]
Primosomal replication protein N	priB	b4201	2	208	[76,78]
Primosomal replication protein N"	priC	b0467	1	175	[76,78]
Primosome factor	dnaT	b4362	3	537	[76]
DNA ligase	ligA	b2411	1	671	[71]
Ribonuclease HI	rnhA	b0214	1	155	
Ribonuclease HII	rnhB	b0183	1	198	
Sum				48882	

[15.1] DNA polymerase III holoenzyme τ and γ subunits are both products of gene dnaX, which contains only one open reading frame for τ. However, γ subunit is generated by -1 frameshift during translation. Frameshift sequence is followed immediately by a termination codon and γ is smaller (total length is 1434 (molecules (dnt) gene^{-1}) than τ[70]. Note that corresponding sequences of γ are not available in KEGG.

[10.8] The value of *approximate* n_{stp} is comparable to the respective value of glucose-specific PTS complex (E I, HPr and transporter (IIA, IIBC) components) of *E. coli* K-12 MG1655 assuming the following subunit composition: $[PtsI]_2[PtsH][PtsG]_2[Crr]$[79,80]. PtsI – PTS enzyme I (b2416), PtsH – phosphocarrier protein HPr (b2415), PtsG – glucose-specific PTS enzyme IIBC component (b1101), Crr – enzyme IIA(glucose) (b2417).
[10.9] The value of *approximate* n_{etc} is comparable to the respective value of the ETC complex of *E. coli* K-12 MG1655 composed of the following components – NADH:quinone oxidoreductase I (EC 7.1.1.2), cytochrome bd-I ubiquinol oxidase (EC 7.1.1.7)[81] and ATP synthase (EC 7.1.2.2) assuming 1:1:1 stoichiometry between different components. The subunit composition of NADH:quinone oxidoreductase I is assumed to be the following: [NuoA][NuoH][NuoJ][NuoK][NuoL][NuoM][NuoN][NuoB][NuoC][NuoE][NuoF][NuoG][NuoI][82]. NuoA – NADH:quinone oxidoreductase I subunit A (b2288), NuoH – NADH:quinone oxidoreductase I subunit H (b2282), NuoJ – NADH:quinone oxidoreductase I subunit J (b2280), NuoK – NADH:quinone oxidoreductase I subunit K (b2279), NuoL – NADH:quinone oxidoreductase I subunit L (b2278), NuoM – NADH:quinone oxidoreductase I subunit M (b2277), NuoN – NADH:quinone oxidoreductase I subunit N (b2276), NuoB – NADH:quinone oxidoreductase I subunit B (b2287), NuoC – NADH:quinone oxidoreductase I subunit C/D (b2286), NuoE – NADH:quinone oxidoreductase I subunit E (b2285), NuoF – NADH:quinone oxidoreductase I subunit F (b2284), NuoG – NADH:quinone oxidoreductase I subunit G (b2283), NuoI – NADH:quinone oxidoreductase I subunit I (b2281). The subunit composition of cytochrome bd-I ubiquinol oxidase is assumed to be the following: [CydA][CydB][CydX][83]. CydA – cytochrome bd-I ubiquinol oxidase subunit I (b0733), CydB – cytochrome bd-I ubiquinol oxidase subunit II (b0734), CydX – cytochrome bd-I ubiquinol oxidase subunit X (b4515). The subunit ATP synthase is assumed to be composed of F0 (subunits a, b and c) and F1 (subunits α, β, γ, δ and ε) complexes with the following overall subunit composition: $[AtpB][AtpF]_2[AtpE]_{10}[AtpA]_3[AtpD]_3[AtpG][AtpH][AtpC]$[84]. AtpB – ATP synthase F0 complex subunit a (b3738), AtpF – ATP synthase F0 complex subunit b (b3736), AtpE – ATP synthase F0 complex subunit c (b3737), AtpA – ATP synthase F1 complex subunit α (b3734), AtpD – ATP synthase F1 complex subunit β (b3732), AtpG – ATP synthase F1 complex subunit γ (b3733), AtpH – ATP synthase F1 complex subunit δ (b3735), AtpH – ATP synthase F1 complex subunit ε (b3731).
[10.10] Values of $n_{mrna\_cell\_comp}$ are calculated from Supplementary Eq. (109):

$$n_{mrna\_cell\_comp} = 3 \cdot n_{cell\_comp} \qquad (109)$$

where coefficient 3 represents the codon. It is assumed that transcripts do not include noncoding regions, stop-codons etc to simplify the model.

Supplementary Table 16. Masses of cell component molecules/complexes (m_{cell_comp}).

Cell component [16.2]	Symbol	Value	Term	Unit
Amino acid [16.1]	m_{aa}	$1.97 \cdot 10^{-22}$	*average*	g aa^{-1}
Deoxyribonucleotide [16.1]	m_{dnt}	$5.12 \cdot 10^{-22}$	*average*	g dnt^{-1}
Ribonucleotide [16.1]	m_{nt}	$5.34 \cdot 10^{-22}$	*average*	g nt^{-1}
DNA	m_{dna}	$4.75 \cdot 10^{-15}$	*approximate*	g $genome^{-1}$
rRNA complex	m_{rrna}	$2.44 \cdot 10^{-18}$	*approximate*	g $rrna^{-1}$
tRNA	m_{trna}	$4.11 \cdot 10^{-20}$	*approximate*	g $trna^{-1}$
mRNA of replisome complex (RC)	m_{mrna_rc}	$7.83 \cdot 10^{-17}$	*approximate*	g $(mrna\ rc)^{-1}$
mRNA of RNA polymerase (RP)	m_{mrna_rp}	$8.93 \cdot 10^{-18}$	*approximate*	g $(mrna\ rp)^{-}$

complex				[1]
mRNA of lipid synthesis enzyme (LPE)	m_{mrna_lpe}	$4.80 \cdot 10^{-19}$	*generic*	g (mrna lpe)$^{-1}$
mRNA of ribosomal protein (RPC) complex	m_{mrna_rpc}	$1.16 \cdot 10^{-17}$	*approximate*	g (mrna rpc)$^{-1}$
mRNA of transport protein	m_{mrna_stp}	$3.20 \cdot 10^{-18}$	*approximate*	g (mrna stp)$^{-1}$
mRNA of electron transport chain (ETC) complex	m_{mrna_etc}	$1.60 \cdot 10^{-17}$	*approximate*	g (mrna etc)$^{-1}$
mRNA of the enzyme of metabolic pathways	m_{mrna_enz}	$4.80 \cdot 10^{-19}$	*generic*	g (mrna enz)$^{-1}$
RC	m_{rc}	$9.65 \cdot 10^{-18}$	*approximate*	g rc^{-1}
RP complex	m_{rp}	$1.10 \cdot 10^{-18}$	*approximate*	g rp^{-1}
LPE	m_{lpe}	$5.92 \cdot 10^{-20}$	*generic*	g lpe^{-1}
RPC	m_{rpc}	$1.43 \cdot 10^{-18}$	*approximate*	g rpc^{-1}
Transport protein	m_{stp}	$3.95 \cdot 10^{-19}$	*approximate*	g stp^{-1}
ETC	m_{etc}	$1.97 \cdot 10^{-18}$	*approximate*	g etc^{-1}
Enzyme of metabolic pathways	m_{enz}	$5.92 \cdot 10^{-20}$	*generic*	g enz^{-1}
Membrane lipid [16.3]	m_{lip}	$6.44 \cdot 10^{-22}$	*generic*	g lip^{-1}

[16.1] The values of *average* m_{mon} are calculated from Supplementary Eq. (110):

$$m_{mon} = \frac{Mw_{mon}}{N_A} \tag{110}$$

where *specific, precise* $N_A = 6.02 \cdot 10^{23}$ molecules mol^{-1}. The values of molar masses of monomers in the model are the *average* values of actual monomers presented in Supplementary Table 17.

Supplementary Table 17. Molar masses of polymerized monomers (*Mw_{mon}*).

Monomer	Symbol	Value	Term	Unit
Amino acid [17.1]	Mw_{aa}	118.89	*average*	g (mol (aa))$^{-1}$
Ribonucleotide [17.2]	Mw_{nt}	321.46	*average*	g (mol (nt))$^{-1}$
Deoxyribonucleotide [17.3]	Mw_{dnt}	308.46	*average*	g (mol (dnt))$^{-1}$

[17.1] *Average* Mw_{aa} is the average molar mass of all 20 amino acids (Supplementary Table 18). The molar mass of H_2O is subtracted from the molar masses of all amino acids to take into account the formation of the peptide bond.

Supplementary Table 18. Molar masses of polymerized amino acids.

Amino acid	Molar mass g (mol (aa))$^{-1}$
Alanine	71.08
Arginine	156.19

Asparagine	114.10
Aspartate	115.09
Cysteine	103.14
Glutamine	128.13
Glutamate	129.12
Glycine	57.05
Histidine	137.14
Isoleucine	113.16
Leucine	113.16
Lysine	128.17
Methionine	131.20
Phenylalanine	147.18
Proline	97.12
Serine	87.08
Threonine	101.11
Tryptophan	186.21
Tyrosine	163.17
Valine	99.13
Average	118.89

[17.2] The *average* Mw_{nt} is the average molar mass of all 4 ribonucleotides (Supplementary Table 19). The molar mass of H_2O is subtracted from the molar masses of all ribonucleotides in order to take into account the formation of the phosphodiester bond.

Supplementary Table 19. Molar masses of polymerized ribonucleotides.

**Ribonucleotide**	**Molar mass g (mol (nt))$^{-1}$**
AMP	329.22
CMP	305.20
GMP	345.22
UMP	306.18
Average	321.46

[17.3] The *average* Mw_{dnt} is the average molar mass of all 4 deoxyribonucleotides (Supplementary Table 20). The molar mass of H_2O is subtracted from the molar masses of all deoxyribonucleotides in order to take into account the formation of the phosphodiester bond.

Supplementary Table 20. Molar masses of polymerized deoxyribonucleotides.

**Deoxyribonucleotide**	**Molar mass g (mol (dnt))$^{-1}$**
dAMP	313.22

dCMP	289.19
dGMP	329.22
dTMP	302.19
Average	308.46

[16.2] Values of m_{cell_comp} except monomers and membrane lipid are calculated from Supplementary Eq. (111):

$$m_{cell\_comp} = n_{cell\_comp} \cdot m_{mon} \tag{111}$$

[16.3] The *generic* m_{lip} (Mw_{lip} = 388 g (mol (lip))$^{-1}$) is comparable to that of monopalmitin phosphoglyceride (C19H39O7P).

Supplementary Table 21. The cell membrane surface areas occupied by single cell components located on the cell membrane (s_{cell_comp}).

Cell component [21.1]	Symbol	Value	Term	Unit
Transport protein	s_{stp}	$2.21 \cdot 10^{-13}$	*approximate*	cm^2 stp^{-1}
Electron transport chain	s_{etc}	$1.04 \cdot 10^{-12}$	*approximate*	cm^2 etc^{-1}
Membrane lipid [21.2]	s_{lip}	$2.75 \cdot 10^{-15}$	*Generic*	cm^2 lip^{-1}

[21.1] The values of s_{cell_comp} are calculated from Supplementary Eq. (112) assuming that different subunits formed a tight complex with an ideal cylindrical shape:

$$s_{cell\_comp} = \frac{m_{cell\_comp}}{h_{mprot} \cdot \rho_{mprot}} \tag{112}$$

Where *generic* h_{stp} = $1.45 \cdot 10^{-6}$ cm stp^{-1} and *generic* h_{etc} = $1.54 \cdot 10^{-6}$ cm stp^{-1} which are approximately 3 times higher than the thickness of lipid bilayer membrane assuming it is similar to the inner membrane of Gram-negative bacterial cell[85] and *generic* ρ_{mprot} = 1.23 g (mprot) (cm^3 (mprot))$^{-1}$ [86].

[21.2] Surface areas of cell membrane occupied by different single phospholipid heads vary between 40 – 70 Å^2 [87,88]. The average value of 55 Å^2 was chosen and was divided by 2 to take into account the bilayer of the cell membrane. It is assumed that there are no differences between surface areas from the inner and outer layers in order to simplify the model.

Supplementary Table 22. Polysome densities of mRNAs of proteins (P_{cell_comp}).

**Protein** [22.1]	**Symbol**	**Value**	**Term**	**Unit**	**Additional explanation**
Replisome complex (RC)	P_{rc}	0.01	*generic*	molecules (nt (covered by rs)) (molecules (nt (covered by tot rs)))$^{-1}$	Reciprocal value of P_{rc} (100 molecules (rs rc) cell^{-1} (molecules (mrna rc) cell^{-1})$^{-1}$) is the number of ribosome molecules per molecule of mRNA of RC
RNA polymerase (RP) complex	P_{rp}	0.01	*generic*	molecules (nt (covered by rs)) (molecules (nt (covered by tot rs)))$^{-1}$	Reciprocal value of P_{rp} (100 molecules (rs rp) cell^{-1} (molecules (mrna rp) cell^{-1})$^{-1}$) is the number of ribosome molecules per molecule of mRNA of RP
Ribosomal protein complex (RPC)	P_{rpc}	0.01	*generic*	molecules (nt (covered by rs)) (molecules (nt (covered by tot rs)))$^{-1}$	Reciprocal value of P_{rpc} (100 molecules (rs rpc) cell^{-1} (molecules (mrna rpc) cell^{-1})$^{-1}$) is the number of ribosome molecules per molecule of mRNA of RPC
Lipid synthesis enzyme (LPE)	P_{lpe}	0.05	*generic*	molecules (nt (covered by rs)) (molecules (nt (covered by tot rs)))$^{-1}$	Reciprocal value of P_{lpe} (20 molecules (rs lpe) cell^{-1} (molecules (mrna lpe) cell^{-1})$^{-1}$) is the number of ribosome molecules per molecule of mRNA of LPE
Transport protein	P_{stp}	0.01	*generic*	molecules (nt (covered by rs)) (molecules (nt (covered by tot rs)))$^{-1}$	Reciprocal value of P_{stp} (100 molecules (rs stp) cell^{-1} (molecules (mrna stp) cell^{-1})$^{-1}$) is the number of ribosome molecules per molecule of mRNA of transport protein
Electron transport chain (ETC) complex	P_{etc}	0.01	*generic*	molecules (nt (covered by rs)) (molecules (nt (covered by tot rs)))$^{-1}$	Reciprocal value of P_{etc} (100 molecules (rs etc) cell^{-1} (molecules (mrna etc) cell^{-1})$^{-1}$) is the number of ribosome molecules per molecule of mRNA of ETC
Enzyme of metabolic pathways	P_{enz}	0.05	*generic*	molecules (nt (covered by rs)) (molecules (nt (covered by tot rs)))$^{-1}$	Reciprocal value of P_{enz} (20 molecules (rs enz) cell^{-1} (molecules (mrna enz) cell^{-1})$^{-1}$) is the number of ribosome molecules per molecule of mRNA of enzyme

[22.1] P_{cell_comp} determines the ratio of $N_{mrna\_cell\_comp}$ to $N_{rs\_cell\_comp}$ (Supplementary Eqs. (94)-(100)).

P_{cell_comp} have different *generic* values for different proteins depending on the size. For example, the aforementioned *generic* value of P_{enz} refers to 20 ribosomes involved in the translation of enzymes on a single mRNA whereas there are 100 ribosomes in the case of a much larger ETC complex assuming that 1 ribosome covers approximately 40 ribonucleotides on mRNA[89]. The values of $P_{cell\_comp\_min}$ (an inverse (reciprocal) ratio of $n_{mrna\_cell\_comp}$ to *approximate* u_{rs}) are calculated from Supplementary Eq. (113) assuming that mRNAs are fully covered by ribosomes (Supplementary Table 23):

$$P_{cell\_comp\_\min} = \left(\frac{n_{mrna\_cell\_comp}}{u_{rs}} \right)^{-1} \tag{113}$$

It appears that *generic* P_{cell_comp} values are near-minimal for shorter mRNAs. In the case of the aforementioned *generic* P_{enz} value, ribosomes cover 800 and they do not cover 100 ribonucleotides on the respective mRNA. In the case of the P_{enz_min} value, the mRNA of the enzyme has space for 22.52 ribosomes.

Note that P_{cell_comp} can be defined based on different parameters of cell components but these different definitions are all equivalent by their values. It is the ratio of *approximate* u_{rs} to the number of ribonucleotide molecules of mRNA covered by all ribosomes (the corresponding unit is molecules (nt (covered by rs)) (molecules (nt (covered by tot rs)))$^{-1}$). For example, 40 ribonucleotide molecules per 800 ribonucleotide molecules in the case of *generic* P_{enz} value. Alternatively, P_{cell_comp} is the ratio of the sum of *approximate* u_{rs} and the number of free ribonucleotide molecules of mRNA per ribosome to the $n_{mrna\_cell\_comp}$ (the corresponding unit is molecules (nt (allocated to rs)) (molecules (nt (mrna prot)$^{-1}$))$^{-1}$). For example, the sum of 40 ribonucleotide molecules (covered by a single ribosome) and 5 ribonucleotide molecules (not covered by the ribosome, 100 ribonucleotide molecules per 20 ribosomes) per 900 ribonucleotide molecules in case of *generic* P_{enz} value. Finally, P_{cell_comp} can be interpreted as the inverse ratio of $N_{rs\_cell\_comp}$ to $N_{mrna\_cell\_comp}$ (the corresponding unit is molecules (mrna prot) cell^{-1} (molecules (rs prot) cell^{-1})$^{-1}$).

Supplementary Table 23. Calculated minimal polysome densities of mRNAs of proteins ($P_{cell\_comp\_min}$).

Protein	Symbol	Value	Unit	Additional explanation
Replisome complex (RC)	P_{rc_min}	$2.73 \cdot 10^{-4}$	molecules (nt (covered by rs)) (molecules (nt (mrna rc)$^{-1}$))$^{-1}$	Reciprocal value of P_{rc_min} (3663 molecules (rs rc) cell^{-1} (molecules (mrna rc) cell^{-1})$^{-1}$) is the maximum number of ribosome molecules per molecule of mRNA of RC
RNA polymerase (RP) complex	P_{rp_min}	$2.39 \cdot 10^{-3}$	molecules (nt (covered by rs)) (molecules (nt (mrna rp)$^{-1}$))$^{-1}$	Reciprocal value of P_{rp_min} (418.41 molecules (rs rp) cell^{-1} (molecules (mrna rp) cell^{-1})$^{-1}$) is the maximum number of ribosome molecules per molecule of mRNA of RP
Lipid synthesis enzyme (LPE)	P_{lpe_min}	$4.44 \cdot 10^{-2}$	molecules (nt (covered by rs)) (molecules (nt (mrna lpe)$^{-1}$))$^{-1}$	Reciprocal value of P_{lpe_min} (22.52 molecules (rs lpe) cell^{-1} (molecules (mrna lpe) cell^{-1})$^{-1}$) is the maximum number of ribosome molecules per molecule of mRNA of LPE
Transport protein	P_{stp_min}	$6.67 \cdot 10^{-3}$	molecules (nt (covered by rs)) (molecules (nt (mrna stp)$^{-1}$))$^{-1}$	Reciprocal value of P_{stp_min} (149.93 molecules (rs stp) cell^{-1} (molecules (mrna stp) cell^{-1})$^{-1}$) is the maximum number of ribosome molecules per molecule of mRNA of transport protein
Electron transport chain (ETC)	P_{etc_min}	$1.33 \cdot 10^{-3}$	molecules (nt (covered by rs)) (molecules (nt (mrna etc)$^{-1}$))$^{-1}$	Reciprocal value of P_{etc_min} (751.88 molecules (rs etc) cell^{-1} (molecules (mrna etc) cell^{-1})$^{-1}$) is the maximum number of ribosome molecules per molecule of mRNA of ETC
Enzyme of metabolic pathways	P_{enz_min}	$4.44 \cdot 10^{-2}$	molecules (nt (covered by rs)) (molecules (nt (mrna enz)$^{-1}$))$^{-1}$	Reciprocal value of P_{enz_min} (22.52 molecules (rs enz) cell^{-1} (molecules (mrna enz) cell^{-1})$^{-1}$) is the maximum number of ribosome molecules per molecule of mRNA of enzyme
Ribosomal protein complex (RPC)	P_{rpc_min}	$1.84 \cdot 10^{-3}$	molecules (nt (covered by rs)) (molecules (nt (mrna rpc)$^{-1}$))$^{-1}$	Reciprocal value of P_{rpc_min} (543.48 molecules (rs rpc) cell^{-1} (molecules (mrna rpc) cell^{-1})$^{-1}$) is the maximum number of ribosome molecules per molecule of mRNA of RPC

Supplementary Table 24. Values of additional input cell parameters.

Input cell parameter	Symbol	Value	Term	Unit
Dry weight content of cell [24.1]	*DWC*	0.3	*approximate*	g (dw cell) (g (cell))$^{-1}$
Ratio of length of the cylindrical part of the cell to cell radius [24.2]	*HR*	2.2	*approximate*	cm (cylinder) (cm (radius))$^{-1}$
Doubling time [24.3]	$t_{d\_srs\text{-}m}$			s
Cell density [24.4]	ρ_{tot}	1	*generic*	g (cell) (cm^3 (cell))$^{-1}$
Genome copy number [24.5]	N_{dna}	1	*approximate*	molecules (genome) cell^{-1}
Number of replisome complexes [24.6]	N_{rc}	2	*approximate*	molecules (rc) cell^{-1}
Genome replication time [24.7]	t_C	2319.84	*approximate*	s or s genome^{-1}

[24.1] The value is comparable to experimentally determined values (31-32 % (g (dw cell) (g (cell))$^{-1}$)) of *E. coli* cells[90].
[24.2] The value was approximated from cell length (H_{cyl} + 2 R_{tot} in SSPCM-SRS-M) and cell width (2 R_{tot} in SSPCM-SRS-M) measurement data of membrane elusion selected new-born *E. coli* B/rA cells[91] assuming that the cell shape did not change with t_d changes[92]. The value corresponds approximately to the mean cell length (H_{cyl} + R_{tot} in SSPCM-SRS-M) and mean cell width (2 R_{tot} in SSPCM-SRS-M) values of *E. coli* K-12 BW25113 cells grown on glucose or glucosamine[30].
[24.3] Independent variable.
[24.4] The value is comparable to experimentally determined values (1.09-1.13 g (cell) (cm^3 (cell))$^{-1}$) of *E. coli* cells[90].
[24.5] The value of N_{dna} corresponds to slowly growing cells that contain a single genome at the beginning of the cell cycle and during the B period of the cell cycle[7].
[24.6] The value of N_{rc} corresponds to the replication requirements of a single genome assuming that a pair of RCs are necessary for the bidirectional replication of a circular chromosome starting from the origin region and meeting again at the opposite side of the chromosome (terminus region)[42].
[24.7] The value t_C is determined by the synthesis rate of the new DNA chain or more precisely by k_{dp} (Supplementary Eq. (114)):

$$t_C = \frac{n_{dna}}{4 \cdot k_{dp}} \tag{114}$$

Coefficient 4 shows that the replication of a genome requires two RC for both directions of a circular chromosome and each RC contains in turn two active DNA polymerase III molecules (bidirectional replication of leading and lagging strands) that are all working in parallel[53].

Supplementary Discussion 5.11.2.2.2: Output parameters

The selection of output parameters of the model (N_{cell_comp}, F_{cell_comp}, cell composition, geometric dimensions and sizes) is presented in (Supplementary Tables 25-26).

Supplementary Table 25. Selection of output parameters of SSPCM-SRS-M.

Cell component	Symbol	Unit
Specific growth rate [25.1]	μ	h^{-1}
Numbers of molecules/complexes of cell components in the cell		
Ribosome	N_{rs}	molecules (rs) $cell^{-1}$
rRNA complex	N_{rrna}	molecules (rrna) $cell^{-1}$
tRNA	N_{trna}	molecules (trna) $cell^{-1}$
RNA polymerase (RP) compex	N_{rp}	molecules (rp) $cell^{-1}$
Lipid synthesis enzyme (LPE)	N_{lpe}	molecules (lpe) $cell^{-1}$
Transport protein	N_{stp}	molecules (stp) $cell^{-1}$
Electron transport chain (ETC) complex	N_{etc}	molecules (etc) $cell^{-1}$
Membrane lipid	N_{lip}	molecules (lip) $cell^{-1}$
Enzyme of central metabolic pathway PW_1 (per reaction)	$N_{enz\_PW1\_r}$	molecules (enz PW_1) $cell^{-1}$ $reaction^{-1}$
Enzyme of amino acid biosynthesis pathway PW_2 (per reaction)	$N_{enz\_PW2\_r}$	molecules (enz PW_2) $cell^{-1}$ $reaction^{-1}$
Enzyme of deoxyribonucleotide biosynthesis pathway PW_3 (per reaction)	$N_{enz\_PW3\_r}$	molecules (enz PW_3) $cell^{-1}$ $reaction^{-1}$
Enzyme of ribonucleotide biosynthesis pathway PW_4 (per reaction)	$N_{enz\_PW4\_r}$	molecules (enz PW_4) $cell^{-1}$ $reaction^{-1}$
Enzyme of lipid biosynthesis pathway PW_5 (per reaction)	$N_{enz\_PW5\_r}$	molecules (enz PW_5) $cell^{-1}$ $reaction^{-1}$
Enzyme of amino acid biosynthesis pathway PW_2 (per pathway) [25.2]	N_{enz_PW2}	molecules (enz PW_2) $cell^{-1}$
mRNA of replisome complex	N_{mrna_rc}	molecules (mrna rc) $cell^{-1}$
mRNA of RP complex	N_{mrna_rp}	molecules (mrna rp) $cell^{-1}$
mRNA of LPE	N_{mrna_lpe}	molecules (mrna lpe) $cell^{-1}$
mRNA of ribosomal protein complex	N_{mrna_rpc}	molecules (mrna rpc) $cell^{-1}$
mRNA of transport protein	N_{mrna_stp}	molecules (mrna stp) $cell^{-1}$
mRNA of ETC	N_{mrna_etc}	molecules (mrna etc) $cell^{-1}$
mRNA of the enzyme of metabolic pathways	N_{mrna_enz}	molecules (mrna enz) $cell^{-1}$
Metabolic fluxes of reactions/pathways/processes [25.3]		
Reaction r of pathway PW_i	$F_{enz\_PWi\_r}$	molecules (metabolite) s^{-1} $cell^{-1}$ $reaction^{-1}$
Reaction r of amino acid synthesis pathway PW_2	$F_{enz\_PW2\_r}$	molecules (metabolite) s^{-1} $cell^{-1}$ $reaction^{-1}$
Translation flux	F_{rs}	molecules (aa) s^{-1} $cell^{-1}$
Cell composition		
DNA content [25.4]	$DNA\%_{mmc}$	[% (g (tot dna) (g (dw

		cell))$^{-1}$)
Membrane lipid content [25.4]	$LIP\%_{mmc}$	[% (g (tot lip) (g (dw cell))$^{-1}$)
Ribosomal content [25.5]	$RS\%_{mc}$	% (g (tot rs) (g (dw cell))$^{-1}$)

[25.1] μ is calculated from Supplementary Eq. (115):

$$\mu = \frac{\ln 2 \cdot 3600}{t_d} \tag{115}$$

[25.2] N_{enz_PW2} is calculated from Supplementary Eq. (116):

$$N_{enz\_PW_2} = N_{enz\_PW_2\_r} \cdot l_{PW_2} \tag{116}$$

[25.3] F_{cell_comp} is calculated from Supplementary Eq. (117):

$$F_{cell\_comp} = N_{cell\_comp\_cat} \cdot k_{cell\_comp} \tag{117}$$

[25.4] Value of $cell\_comp\%_{mmc}$ is calculated from Supplementary Eq. (118):

$$cell\_comp\%_{mmc} = \frac{\sum_{cell\_comp \in mmc\_fraction} \left(N_{cell\_comp} \cdot m_{cell\_comp}\right)}{M_{tot} \cdot DWC} \cdot 100 \tag{118}$$

[25.5] Value of $RS\%_{mc}$ is calculated from Supplementary Eq. (119):

$$RS\%_{mc} = \frac{N_{rs} \cdot m_{rpc} + N_{rrna} \cdot m_{rrna}}{M_{tot} \cdot DWC} \cdot 100 \tag{119}$$

Supplementary Table 26. Cell geometry parameters.

Geometry parameter	Symbol	Unit
Mass of cell	M_{tot}	g cell^{-1}
Mass of cytoplasm of the cell	M_{cyt}	g cyt^{-1}
Mass of cell membrane [26.1]	M_{mem}	g mem^{-1}
Cell volume [26.2]	V_{tot}	cm^3 cell^{-1}
Volume of cytoplasmic space	V_{cyt}	cm^3 cyt^{-1}
Surface area of cell [26.3]	S_{tot}	cm^2 cell^{-1}
Cell radius [26.4]	R_{tot}	cm cell^{-1}
Length of the cylindrical part of the cell [26.5]	H_{cyl}	cm cell^{-1}
Total surface area of cell membrane covered by cell component molecules [26.6]		
Electron transport chain	S_{etc}	cm^2 (tot etc)$^{-1}$
Membrane lipid	S_{lip}	cm^2 (tot lip)$^{-1}$
Substrate transport protein	S_{stp}	cm^2 (tot stp)$^{-1}$

[26.1] M_{mem} is calculated from Supplementary Eq. (120):

$$M_{mem} = \frac{N_{lip} \cdot m_{lip} + N_{stp} \cdot m_{stp} + N_{etc} \cdot m_{etc}}{DWC} \quad (120)$$

26.2 V_{tot} is calculated from Supplementary Eq. (121):

$$V_{tot} = \frac{M_{tot}}{\rho_{tot}} \quad (121)$$

26.3 S_{tot} is calculated from Supplementary Eq. (122):

$$S_{tot} = 2 \cdot \pi \cdot \left(HR + 2\right) \cdot \left(\frac{V_{tot}}{\left(HR + \frac{4}{3}\right) \cdot \pi} \right)^{\frac{2}{3}} \quad (122)$$

Derivation of Supplementary Eq. (122) and other cell geometry formulas has been reported in[3].

26.4 R_{tot} is calculated from Supplementary Eq. (123):

$$R_{tot} = \left(\frac{S_{tot}}{2\pi \cdot \left(HR + 2\right)} \right)^{\frac{1}{2}} \quad (123)$$

26.5 H_{cyl} is calculated from Supplementary Eq. (124):

$$H_{cyl} = HR \cdot R_{tot} \quad (124)$$

26.6 S_{cell_comp} is calculated from Supplementary Eq. (125):

$$S_{cell\_comp} = N_{cell\_comp} \cdot s_{cell\_comp} \quad (125)$$

Supplementary Discussion 5.11.2.3: Calculation scheme

A short overview of SSPCM-SRS-M calculations, solving and mathematical properties of systems of equations are presented here.

The models are based on systems of algebraic equations that are characterized by a larger number of independent parameters (approximately 1/3) than the number of independent equations – see Supplementary Discussion 5.11.2.2. As the aim of the present work was to explain and illustrate the basic principles of cells by using very simplified models, the number of degrees of freedom (unknown parameters) was reduced in order to simplify also solving of systems of equations. The number of unknown parameters (approximately 2/3 of the parameters) is equal to the number of equations. Therefore, unique solutions can be found for determined systems of equations. Note that in the case of living bacterial cells the models would be usually heavily underdetermined and solving them requires further simplifications or applications of certain optimization algorithms by formulating meaningful objective functions.

The current division of input (known) and output (unknown) parameters of equations, input parameter values which are necessary to carry out calculations of output parameters are provided in the previous section (Supplementary Discussions 5.11.2.2, 5.11.2.2.1-5.11.2.2.2). As mentioned, the division is mainly based on the availability of data and physiological considerations (parameters determined only by the genotype) although *generic* input parameter values had to be also included. Also, there are some considerations about model solving efficiency. Most of the equations cannot be solved and most of the unknown parameters cannot be calculated separately due to the interrelations between equations and unknown parameters. However, careful selection of input and output parameters enabled to solve the model during different subsequent steps which is certainly easier than solving the full system of equations.

Firstly, certain equations/parameters describing DNA replication and associated polymerization (in addition to masses of cell components (Supplementary Tables 16-20), lengths of mRNA sequences (Supplementary Table 10), t_C (Supplementary Table 24) etc) can be calculated separately (Supplementary Eqs. (74), (87), (94), (108) and corresponding output parameters: $N_{enz\_PW3\_r}$, N_{rs_rc}, N_{mrna_rc}, N_{rce}). The second step is mathematically most complicated and involves solving of the main system of equations (29 equations in case of SSPCM-SRS-M (Supplementary Eqs. (73), (75)-(78), (80), (82)-(86), (88), (90)-(93), (95)-(107)) based on values of output parameters found from the first step. Finally, more equations describing for example flux patterns (F_{cell_comp}), cellular compositions ($cell\_comp\%_{mmc}$, $RS\%_{mc}$) or geometry properties (presented in Supplementary Tables 25-26) can be solved separately (Supplementary Eqs. (115)-(125) in case of SSPCM-SRS-M) and corresponding parameters calculated by utilizing output parameter values from the solution of the main system of equations.

It should be stressed that different set of input/output parameters would also change the solving process. For example, if $t_{d\_srs\text{-}m}$ is an unknown parameter, then the model solving becomes more complicated compared to the case if $t_{d\_srs\text{-}m}$ is a known parameter. This is because equations/parameters describing DNA replication and associated polymerization (Supplementary Eqs. (74), (87), (94), (108)) could not be solved separately anymore and the main system of equations would be larger (33 equations in case of SSPCM-SRS-M) due to those equations. Also, $t_{d\_srs\text{-}m}$ appears in nonlinear balance equations that correspond to the doubling condition (Supplementary Eqs. (80), (82)-(87), (90), (101), (108) in case of SSPCM-SRS-M) and solving of the system of nonlinear equations with additional unknown parameter is usually computationally much more demanding. Another example is related to equations describing fluxes that are defined as N_{cell_comp} multiplied with k_{cell_comp} as in Supplementary Eq. (117). Currently, N_{cell_comp} are selected as unknown parameters and k_{cell_comp} as known parameters. From the physiological viewpoint, the latter parameter depends also on cell growth and therefore it is principally justified to treat k_{cell_comp} as an unknown parameter. However, model solving would be then more complicated again as F_{cell_comp} cannot be calculated anymore after solving the main system of equations. Instead, those equations must be also included in the main system of equations due to two unknowns per equation.

Supplementary Table 27. Calculated rounded values of selected output parameters of proto-cell growing in minimal medium (SSPCM-SRS-M) at different doubling time values.

Parameter	Symbol	Unit				
Doubling time	$t_{d\_srs\text{-}m}$	s	2474.27	2490	3520	40000
Number of cell component molecules/complexes in the cell						
Ribosome	N_{rs}	molecules (rs) cell^{-1}	36992	19776	337	0.99
Ribosomes for the synthesis of ribosomal protein complex (RPC)	N_{rs_rpc}	molecules (rs rpc) cell^{-1}	5414	2876	35	$9\cdot10^{-3}$
Ribosomes for the synthesis of enzymes in central and biosynthesis pathways PW_1-PW_5	N_{rs_enz}	molecules (rs enz) cell^{-1}	19874	10619	175	0.42
Ribosomes for the synthesis of RNA polymerase (RP) complex	N_{rs_rp}	molecules (rs rp) cell^{-1}	214	113	0.97	$2.3\cdot10^{-5}$
Ribosomes for the synthesis of lipid synthesis enzyme (LPE)	N_{rs_lpe}	molecules (rs lpe) cell^{-1}	$4.7\cdot10^{-5}$	0.07	0.02	10^{-4}
Ribosomes for the synthesis of electron transport chain (ETC)	N_{rs_etc}	molecules (rs etc) cell^{-1}	11158	5989	122	0.43
Ribosomes for the synthesis of substrate transport protein	N_{rs_stp}	molecules (rs stp) cell^{-1}	331	177	3	$7\cdot10^{-3}$
Ribosomes for the synthesis of replisome complex (RC)	N_{rs_rc}	molecules (rs rc) cell^{-1}	2	2	1	0.12
RP	N_{rp}	molecules (rp) cell^{-1}	1899	1009	12	$3\cdot10^{-3}$
LPE	N_{lpe}	molecules (lpe) cell^{-1}	$8\cdot10^{-3}$	11	4	0.30
ETC	N_{etc}	molecules (etc) cell^{-1}	55214	29827	858	34
Substrate transport protein	N_{stp}	molecules (stp) cell^{-1}	8196	4407	103	3
Effective RC	N_{rce}	molecules (rce) cell^{-1}	2	2	1	0.12
Enzyme catalysing a single reaction r of central metabolic pathway PW_1	$N_{enz\_PW1\_r}$	molecules (enz PW_1) cell^{-1} reaction^{-1}	8196	4407	103	3
Enzyme catalysing a single reaction r of amino acid biosynthesis pathway PW_2	$N_{enz\_PW2\_r}$	molecules (enz PW_2) cell^{-1} reaction^{-1}	7399	3955	67	0.20

Enzyme catalysing a single reaction r of deoxyribonucleotide biosynthesis pathway PW_3	$N_{enz\_PW3\_r}$	molecules (enz PW_3) $cell^{-1}$ $reaction^{-1}$	38	37	26	2
Enzyme catalysing a single reaction r of ribonucleotide biosynthesis pathway PW_4	$N_{enz\_PW4\_r}$	molecules (enz PW_4) $cell^{-1}$ $reaction^{-1}$	760	404	5	10^{-3}
Enzyme catalysing a single reaction r of lipid biosynthesis pathway PW_5	$N_{enz\_PW5\_r}$	molecules (enz PW_5) $cell^{-1}$ $reaction^{-1}$	$8\cdot10^{-3}$	11	4	0.30
Assembled rRNA complex	N_{rrna}	molecules (rrna) $cell^{-1}$	36992	19776	337	0.99
tRNA	N_{trna}	molecules (trna) $cell^{-1}$	184962	98879	1687	5
mRNA of RPC	N_{mrna_rpc}	molecules (mrna rpc) $cell^{-1}$	54	29	0.35	$9\cdot10^{-5}$
mRNA of RP	N_{mrna_rp}	molecules (mrna rp) $cell^{-1}$	2	1	0.01	$2\cdot10^{-7}$
mRNA of the enzyme in central and biosynthesis pathways PW_1-PW_5	N_{mrna_enz}	molecules (mrna enz) $cell^{-1}$	199	106	2	$4\cdot10^{-3}$
mRNA of LPE	N_{mrna_lpe}	molecules (mrna lpe) $cell^{-1}$	$2\cdot10^{-6}$	$3\cdot10^{-3}$	$9\cdot10^{-4}$	$6\cdot10^{-6}$
mRNA of ETC	N_{mrna_etc}	molecules (mrna etc) $cell^{-1}$	112	60	1	$4\cdot10^{-3}$
mRNA of substrate transport protein	N_{mrna_stp}	molecules (mrna stp) $cell^{-1}$	3	2	0.03	$7\cdot10^{-5}$
mRNA of RC	N_{mrna_rc}	molecules (mrna rc) $cell^{-1}$	0.02	0.02	0.01	0.01
Membrane lipid	N_{lip}	molecules (lip) $cell^{-1}$	1913	$2.70\cdot10^{6}$	$1.48\cdot10^{6}$	$1.22\cdot10^{6}$
Size parameters						
Mass of cytoplasm of the cell	M_{cyt}	g cyt^{-1}	$1.18\cdot10^{-12}$	$6.40\cdot10^{-13}$	$2.87\cdot10^{-14}$	$1.61\cdot10^{-14}$
Cell mass	M_{tot}	g $cell^{-1}$	$1.55\cdot10^{-}$	$8.48\cdot10^{-}$	$3.77\cdot10^{-}$	$1.90\cdot10^{-}$

	12	13	14	14

SUPPLEMENTARY DISCUSSION 5.11.3: DESCRIPTION OF SSPCM-RS

The description of the model (SSPCM-RS) is based on the full description of SSPCM-SRS-M (Supplementary Discussion 5.11.2), only the differences compared with SSPCM-SRS-M are subsequently presented.

Supplementary Discussion 5.11.3.1: Model components and interactions

It is assumed that the proto-cell has no structures and it is only comprised of RPC as the sole macromolecular cell component (Supplementary Figure 1). It is not specified how RPC is secluded from the surrounding environment.

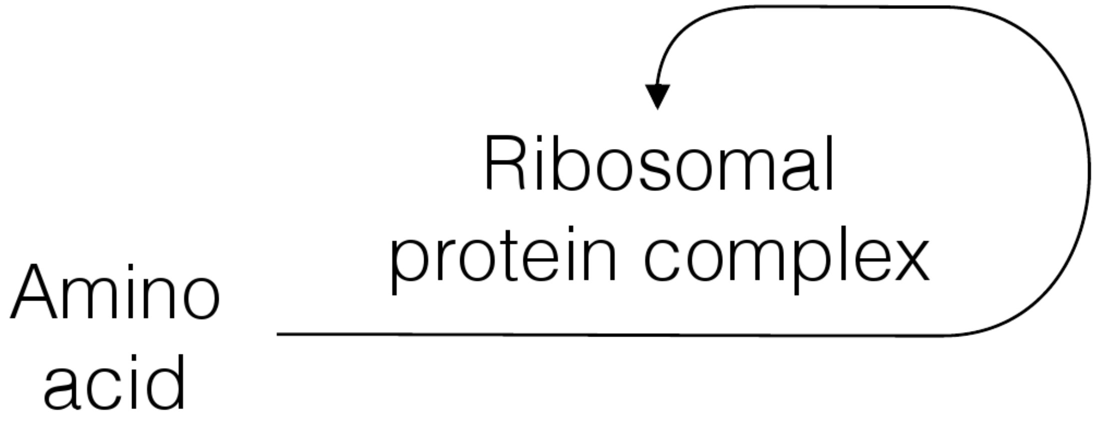

Supplementary Figure 1. Scheme of the bacterial proto-cell SSPCM-RS. The SSPCM-RS model developed includes only ribosomal protein complexes (RPC) and their monomers (amino acids) which are located in pseudo cytoplasm (proto-cell is not separated from the growth environment) (Supplementary Tables 1-2). RPC carries out the synthesis of itself from amino acids (provided by the growth environment) without energy and RNA (Supplementary Table 3).

Supplementary Discussion 5.11.3.1.1: Protein synthesis

The number of RPC is doubled during the t_{d_srs} by RPC itself carrying out translation. It is assumed that rRNA, mRNA, tRNA and energy are not needed for protein synthesis and an unlimited number of amino acids are available in the surrounding environment. Translation (self-reproduction of RPC) is described by Supplementary Eq. (80). N_{rs} is expressed by the following Supplementary Eq. (126):

$$N_{rs} = N_{rs\_rpc} \tag{126}$$

It means that all ribosomes are reproducing themselves.

Supplementary Discussion 5.11.3.1.2: Mass balance

M_{tot} is equal to M_{cyt} and it is the sum of masses of all cell components including water:

$$M_{tot} = M_{cyt} = \frac{N_{rs} \cdot m_{rpc}}{DWC} \quad (127)$$

Supplementary Discussion 5.11.3.2: Model parameters

Corresponding input parameters, their values and output parameters of SSPCM-SRS-M (Supplementary Discussions 5.11.2.2, 5.11.2.2.1-5.11.2.2.2) are also used in the current model except output parameter t_{d_rs} instead of $t_{d\_srs\text{-}m}$.

Supplementary Discussion 5.11.3.3: Calculation scheme

The main equation system of the mathematically determined model consists of only three equations (Supplementary Eqs. (80), (126)-(127)) and the solution for t_{d_rs} is described by Supplementary Eq. (3) (by combining Supplementary Eq. (80) and Supplementary Eq. (126)). As already explained in Supplementary Discussion 5.1, t_{d_rs} is completely independent of N_{rs} and N_{rs_rpc}. Also, the latter parameters cannot be calculated as they are cancelled out from Supplementary Eq. (3). Therefore, t_{d_rs} must be the output parameter whereas N_{rs} or N_{rs_rpc} must be the formal input parameter (they can affect only each other and M_{tot}).

Supplementary Table 28. Calculated rounded values of selected output parameters of SSPCM-RS at different values of a number of ribosomes in the cell.

Parameter	Symbol	Unit		
Number of ribosomes in the cell	N_{rs}	molecules (rs) $cell^{-1}$	1	10^4
Doubling time	t_{d_rs}	s	362.1	362.1
Number of ribosomes for the synthesis of ribosomal protein complex	N_{rs_rpc}	molecules (rs rpc) $cell^{-1}$	1	10^4
Cell mass	M_{tot}	g $cell^{-1}$	$4.77 \cdot 10^{-18}$	$4.77 \cdot 10^{-14}$

SUPPLEMENTARY DISCUSSION 5.11.4: DESCRIPTION OF SSPCM-RS+AA

The description of the model (SSPCM-RS+AA) is based on the full description of SSPCM-SRS-M (Supplementary Discussion 5.11.2), only the differences compared with SSPCM-SRS-M are subsequently presented.

Supplementary Discussion 5.11.4.1: Model components and interactions

It is assumed that the proto-cell has no structures and it is only comprised of RPC and amino acid synthesis enzymes as the only macromolecular cell components (Supplementary Figure 2). It is not specified how cell components are secluded from the surrounding environment.

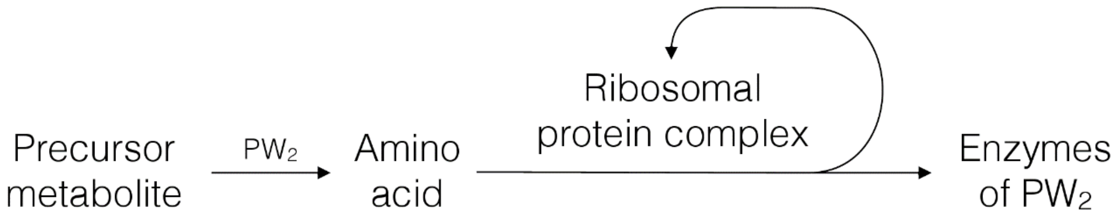

Supplementary Figure 2. Scheme of the bacterial proto-cell SSPCM-RS+AA. The SSPCM-RS+AA model developed includes ribosomal protein complexes (RPC), enzymes of the metabolic network, monomers of proteins (amino acids), precursor and intermediate metabolites which are all located in pseudo cytoplasm (proto-cell is not separated from the growth environment) (Supplementary Tables 1-2). RPC carries out the synthesis of all proteins from amino acids without energy and RNA. Amino acids are synthesized by enzymes in the amino acid biosynthesis pathway PW_2 consisting of a linear reaction chain from precursor metabolite (provided by the growth environment) via metabolic intermediates (Supplementary Table 3).

Distantly, SSPCM-RS+AA is by the cell components similar to the dynamical PTR model[23] which is describing the self-replication of an exponentially growing proto-cell population. These cells are composed of ribosomes and enzymes for amino acid synthesis and substrate transport. Although it has been stated that the enzymes were localized on the cell membrane[23], cell geometry has not been described and it was assumed that the cell membrane was composed of only proteins. Therefore, the T-protein actually resembles very much the enzyme of the amino acid synthesis pathway PW_2 in the SSPCM-RS+AA model. The main differences between models are the absence of an amino acid synthesis pathway in PTR and the absence of protein degradation and the effect of amino acid pool size in the SSPCM-RS+AA model (Supplementary Figure 2). However, the mathematical descriptions of PTR and SSPCM-RS+AA models are different as the base assumptions of models are different.
Similar assumptions and mathematical framework (as in PTR) have been used also for another proto-cell model that resembles PTR with some exceptions[24]. The nonribosomal protein or enzyme fraction carries out also energy synthesis beside substrate conversion to biomass. The model also describes energy balance (ATP consumption by translation) instead of amino acid balance.

Supplementary Discussion 5.11.4.1.1: Metabolic network

It is assumed that the proto-cell converted unspecified metabolic intermediate (available in the surrounding environment) to amino acids of proteins via synthesis pathway PW_2 (Supplementary Figure 2) which reactions are catalysed by identical (*generic* m_{enz}, *generic* n_{enz}) enzymes with the same *generic* k_{enz} as in SSPCM-SRS-M (Supplementary Discussion 5.11.2.1.1). Therefore, Supplementary Eq. (73) describes all fluxes of all reactions for all metabolic intermediates in PW_2.

Supplementary Discussion 5.11.4.1.2: Protein synthesis

The protein fraction of the proto-cell is comprised of the following different proteins (Supplementary Figure 2, Supplementary Table 2):

1. RPC (part of ribosomes, carries out translation).
2. Enzymes on amino acid synthesis pathway PW_2 (carry out the synthesis of metabolic intermediates and amino acids of proteins).

The numbers of all proteins are doubled by ribosomes carrying out translation. It is assumed that rRNA, mRNAs, tRNA and energy are not needed for protein synthesis. $N_{rs\_cell\_comp}$ is determined by t_{d_rs+PW2} and the requirement of amino acids of each protein according to Supplementary Eq. (80) and following Supplementary Eq. (128) that is remarkably similar to Supplementary Eq. (83):

$$t_{d\_rs+PW2} = \frac{N_{enz\_PW2\_r} \cdot n_{enz} \cdot l_{PW2}}{N_{rs\_enz} \cdot k_{rs}} \tag{128}$$

N_{rs} is expressed by the following Supplementary Eq. (129):

$$N_{rs} = N_{rs\_rpc} + N_{rs\_enz} \tag{129}$$

Supplementary Discussion 5.11.4.1.3: Mass balance

M_{tot} is equal to M_{cyt} and it is the sum of masses of all cell components including water:

$$M_{tot} = M_{cyt} = \frac{N_{rs} \cdot m_{rpc} + N_{enz\_PW2\_r} \cdot m_{enz} \cdot l_{PW2}}{DWC} \tag{130}$$

Supplementary Discussion 5.11.4.2: Model parameters

Corresponding input parameters, their values and output parameters of SSPCM-SRS-M (Supplementary Discussions 5.11.2.2, 5.11.2.2.1-5.11.2.2.2) are also used in the current model except:

1. Output parameters $N_{aa\_rpc+enz\_PW2}$, $N_{aa\_enz\_PW2}$ and t_{d_rs+PW2} (the latter instead of t_{d_srs-m}).
2. Input parameter t_{PW2} which is calculated by Supplementary Eq. (6): t_{PW2} = 600 s or s molecules (aa) (molecules (metabolite) $PW_2)^{-1}$.
3. Parameter t_{d_rs} from SSPCM-RS.

Supplementary Discussion 5.11.4.3: Calculation scheme

The main equation system of the mathematically determined model consists of only four equations (Supplementary Eqs. (80), (128)-(130)) and the solution for t_{d_rs+PW2} is described by Supplementary Eq. (5). As already explained in Supplementary Discussion 5.2, t_{d_rs+PW2} is completely independent of N_{cell_comp}. Analogically to the previous model, N_{cell_comp} cannot be calculated as they are cancelled out from Supplementary Eq. (5). Therefore, t_{d_rs+PW2} must be the output parameter whereas one N_{cell_comp} must be the input parameter (it affects other N_{cell_comp} and M_{tot}).

Supplementary Table 29: Calculated rounded values of selected output parameters of proto-cell SSPCM-RS+AA at different values of a number of ribosomes in the cell.

Parameter	Symbol	Unit		
Number of ribosomes in the cell	N_{rs}	molecules (rs) cell^{-1}	1	10^4
Doubling time	t_{d_rs+PW2}	s	962.1	962.1
Number of ribosomes for the synthesis of ribosomal protein complex	N_{rs_rpc}	molecules (rs rpc) cell^{-1}	0.38	3764
Number of ribosomes for the synthesis of enzymes in central and biosynthesis pathways PW_1-PW_5	N_{rs_enz}	molecules (rs enz) cell^{-1}	0.62	6236
Number of molecules of the enzyme catalysing a single reaction r of amino acid biosynthesis pathway PW_2	$N_{enz\_PW2\_r}$	molecules (enz PW_2) cell^{-1} reaction^{-1}	0.2	2000
Number of molecules of the enzyme of amino acid biosynthesis pathway PW_2 in the cell	N_{enz_PW2}	molecules (enz PW_2) cell^{-1}	40	$4\cdot10^5$
Cell mass	M_{tot}	g cell^{-1}	$1.27\cdot10^{-17}$	$1.27\cdot10^{-13}$

SUPPLEMENTARY DISCUSSION 5.11.5: DESCRIPTION OF SSPCM-RS+AA+PROT

The description of the model (SSPCM-RS+AA+PROT) is based on the full description of SSPCM-SRS-M (Supplementary Discussion 5.11.2), only the differences compared with SSPCM-SRS-M are subsequently presented.

Supplementary Discussion 5.11.5.1: Model components and interactions

It is assumed that the proto-cell has no structures and it is only comprised of RPC, amino acid synthesis enzymes, unspecified proteins and amino acids as the only cell components (Supplementary Figure 3). It is not specified how cell components are secluded from the surrounding environment.

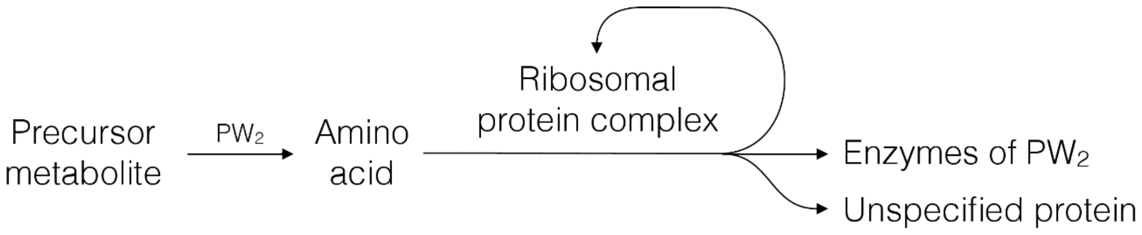

Supplementary Figure 3. Scheme of the bacterial proto-cell SSPCM-RS+AA+PROT. The SSPCM-RS+AA+PROT model developed includes ribosomal protein complexes (RPC), enzymes of the metabolic network, unspecified protein, monomers of proteins (amino acids), precursor and intermediate metabolites which are all located in pseudo cytoplasm (proto-cell is not separated from the growth environment) (Supplementary Tables 1-2). RPC carries out the synthesis of all proteins from amino acids without energy and RNA. Amino acids are synthesized by enzymes in the amino acid biosynthesis pathway PW_2 consisting of a linear reaction chain from precursor metabolite (provided by the growth environment) via metabolic intermediates (Supplementary Table 3). Unspecified protein has no catalytic role in the proto-cell.

A similar model has been published describing the translation process of the ribosome and nonribosomal protein but without amino acid synthesis[25]. An additional model where the nonribosomal protein has been replaced by an inactive ribosome pool has been used to explain the importance of protein degradation and maintenance during slow growth[8].

Supplementary Discussion 5.11.5.1.1: Metabolic network

Amino acid synthesis is described as in Supplementary Discussion 5.11.4.1.1.

Supplementary Discussion 5.11.5.1.2: Protein synthesis

The protein fraction of the proto-cell is comprised of the following different proteins (Supplementary Figure 3):

1. RPC (part of ribosomes, carries out translation) (Supplementary Table 2).
2. Enzymes on amino acid synthesis pathway PW_2 (carry out the synthesis of metabolic intermediates and amino acids of proteins) (Supplementary Table 2).
3. Unspecified intracellular protein.

The numbers of all proteins are doubled by ribosomes carrying out translation. It is assumed that rRNA, mRNAs, tRNA and energy are not needed for protein synthesis. $N_{rs\_cell\_comp}$ is

determined by $t_{d\_rs+PW2+prot}$ and the requirement of amino acids of each protein according to Supplementary Eq. (14).

Supplementary Discussion 5.11.5.1.3: Mass balance

M_{tot} is equal to M_{cyt} and it is the sum of masses of all cell components including water:

$$M_{tot} = M_{cyt} = \frac{N_{rs} \cdot m_{rpc} + N_{enz\_PW2\_r} \cdot l_{PW2} \cdot m_{enz} + N_{aa\_prot} \cdot m_{aa}}{DWC} \quad (131)$$

Supplementary Discussion 5.11.5.2: Model parameters

Corresponding input parameters, their values and output parameters of SSPCM-SRS-M (Supplementary Discussions 5.11.2.2, 5.11.2.2.1-5.11.2.2.2) are also used in the current model except t_{d_rs+PW2} and $N_{aa\_rpc+enz\_PW2}$ from SSPCM-RS+AA, independent variable $t_{d\_rs+PW2+prot}$ instead of $t_{d\_srs\text{-}m}$ and following output parameters:

1. N_{aa_prot} and $N_{aa\_rpc+enz\_PW2+prot}$ which are unique only for SSPCM-RS+AA+PROT.
2. M_{rpc} which is calculated from Supplementary Eq. (132):

$$M_{rpc} = N_{rs} \cdot m_{rpc} \quad (132)$$

Supplementary Discussion 5.11.5.3: Calculation scheme

The main equation system of the model consists of only three equations (Supplementary Eq. (14), Supplementary Eqs. (80), (131)) and the model is mathematically under-determined (one degree of freedom) if $N_{enz\_PW2\_r}$, N_{rs}, N_{aa_prot} and M_{tot} are all unknown. Model solving requires the constraining of one of the previously mentioned parameters. If, for example, N_{rs} is an independent variable, then the solution of the model is partly described by Supplementary Eq. (17).

On the contrary to previous proto-cell models, $t_{d\_rs+PW2+prot}$ is dependent on N_{cell_comp} as already explained in Supplementary Discussion 5.3. Therefore, $t_{d\_rs+PW2+prot}$ can be the input parameter whereas N_{cell_comp} can be the output parameter.

Supplementary Table 30. Calculated rounded values of selected output parameters of proto-cell SSPCM-RS+AA+PROT at different values of a number of ribosomes in the cell and at different doubling time values.

Parameter	Symbol	Unit						
Number of ribosomes in the cell	N_{rs}	molecules (rs) cell^{-1}	1	10^4	1	10^4	1	10^4
Doubling time	$t_{d\_rs+PW2+prot}$	s	962.1	962.1	3520	3520	10000	10000
Number of molecules of the enzyme catalysing a single reaction r of amino acid biosynthesis pathway PW_2	$N_{enz\_PW2\_r}$	molecules (enz PW_2) cell^{-1} reaction^{-1}	0.2	2000	0.2	2000	0.2	2000
Number of molecules of polymerized amino acid of unspecified proteins in the cell	N_{aa_prot}	molecules (aa prot) cell^{-1}	0	0	51158	$5.12 \cdot 10^8$	180758	$1.81 \cdot 10^9$
Combined number of molecules of polymerized amino acid of RPC and enzyme of amino acid biosynthesis pathway PW_2 in the cell	$N_{aa\_rpc+enz\_PW2}$	molecules (aa enz PW_2 rpc) cell^{-1}	19242	$1.92 \cdot 10^8$	19242	$1.92 \cdot 10^8$	19242	$1.92 \cdot 10^8$
Total number of molecules of polymerized amino acid in the proto-cell	$N_{aa\_rpc+enz\_PW2+prot}$	molecules (tot aa) cell^{-1}	19242	$1.92 \cdot 10^8$	70400	$7.04 \cdot 10^8$	$2 \cdot 10^5$	$2 \cdot 10^9$
Cell mass	M_{tot}	g cell^{-1}	$1.27 \cdot 10^{-17}$	$1.27 \cdot 10^{-13}$	$4.63 \cdot 10^{-17}$	$4.63 \cdot 10^{-13}$	$1.31 \cdot 10^{-16}$	$1.31 \cdot 10^{-12}$

SUPPLEMENTARY DISCUSSION 5.11.6: DESCRIPTION OF SSPCM-RS+AA+RNA

The description of the model (SSPCM-RS+AA+RNA) is based on the full description of SSPCM-SRS-M (Supplementary Discussion 5.11.2), only the differences compared with SSPCM-SRS-M are subsequently presented.

Supplementary Discussion 5.11.6.1: Model components and interactions

It is assumed that the proto-cell has no structures and it is only comprised of proteins and RNA (Supplementary Figure 4). It is not specified how cell components are secluded from the surrounding environment.

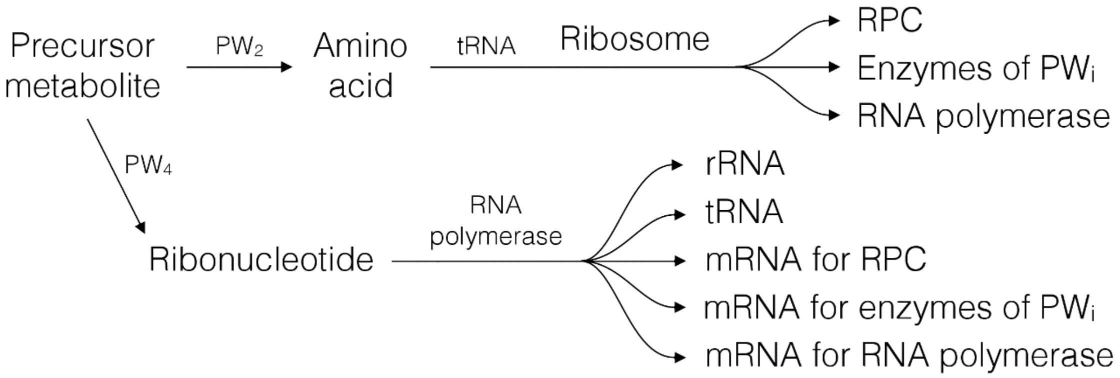

Supplementary Figure 4. Scheme of the bacterial proto-cell SSPCM-RS+AA+RNA. The SSPCM-RS+AA+RNA model developed includes ribosomes, RNA polymerase (RP) complexes, enzymes of the metabolic network, all RNA types (assembled rRNA complex, tRNA, mRNAs of individual proteins), monomers of proteins (amino acids) and RNA (ribonucleotides), precursor and intermediate metabolites which are all located in pseudo cytoplasm (proto-cell is not separated from the growth environment) (Supplementary Tables 1-2). Ribosome (consisting of rRNA and ribosomal protein complex (RPC)) carries out the synthesis of all proteins from amino acids without energy. RP carries out the synthesis of all RNA molecules from ribonucleotides without energy and DNA. Monomers (amino acids, ribonucleotides) are synthesized by enzymes in amino acid and ribonucleotide biosynthesis pathways (PW_2 and PW_4) consisting of linear reaction chains from precursor metabolite (provided by the growth environment) via metabolic intermediates (Supplementary Table 3).

It must be stressed that compared to ribosomes and translation, RNA and transcription have been only rarely analysed. Recently, a coarse-grained model has been presented involving also rRNA synthesis by RP beside ribosome self-reproduction[2]. Compared to SSPCM-RS+AA+RNA (Supplementary Figure 4), model[2] does not involve the synthesis of amino acids and ribonucleotides by biosynthesis pathways. Another simplified model of translation has been constructed that describes the relations between ribosomes, tRNA and rRNA but without modelling the transcription process[28]. Compared to SSPCM-RS+AA+RNA (Supplementary Figure 4), model[28] also does not involve the synthesis of amino acids and ribonucleotides by biosynthesis pathways.

Supplementary Discussion 5.11.6.1.1: Metabolic network

It is assumed that the proto-cell converts the same unspecified metabolic intermediate (available in the surrounding environment) to monomers (ribonucleotide, amino acid) of macromolecules via monomer synthesis pathways PW_i for both monomers (Supplementary Figure 4). All reactions are catalysed by identical (*generic* m_{enz}, *generic* n_{enz}) enzymes with the same *generic* k_{enz} as in SSPCM-SRS-M (Supplementary Discussion 5.11.2.1.1). Therefore, Supplementary Eqs. (73), (75) describe all fluxes of all reactions for all metabolic intermediates in PW_i.

Supplementary Discussion 5.11.6.1.2: Protein synthesis

The protein fraction of the proto-cell is comprised of the following different proteins (Supplementary Figure 4, Supplementary Table 2):

1. RP complex (carries out transcription).
2. RPC (part of ribosomes, carries out translation).
3. Enzymes on biosynthetic pathways PW_2 and PW_4 (carry out the synthesis of metabolic intermediates and monomers of macromolecules).

The numbers of all proteins are doubled by ribosomes carrying out translation. It is assumed that energy is not needed for protein synthesis. $N_{rs\_cell\_comp}$ of all proteins are determined by $t_{d\_rs+PW2+rna}$ and the requirement of amino acids of each protein according to Supplementary Eq. (80) and following Supplementary Eqs. (133)-(134):

$$t_{d\_rs+PW2+rna} = \frac{n_{enz} \cdot \sum_{i=2,4} N_{enz\_PWi\_r} \cdot l_{PWi}}{N_{rs\_enz} \cdot k_{rs}} \quad (133)$$

$$t_{d\_rs+PW2+rna} = \frac{N_{rp} \cdot n_{rp}}{N_{rs\_rp} \cdot k_{rs}} \quad (134)$$

N_{rs} is expressed by the following Supplementary Eq. (135):

$$N_{rs} = N_{rs\_rp} + N_{rs\_rpc} + N_{rs\_enz} \quad (135)$$

Supplementary Discussion 5.11.6.1.3: RNA synthesis

RNA fraction of the proto-cell is comprised of the following different RNAs (Supplementary Figure 4, Supplementary Table 2):

1. Ribosomal RNA complex is based on 3 subunits (5S, 16S, 23S) assembled in ribosomes with 1:1:1 stoichiometry.
2. Transfer RNA. The model was simplified by introducing only one universal tRNA molecule because different amino acids are not specified.
3. There are specific mRNAs coding different proteins in the model. It is assumed that mRNAs contain only coding regions to simplify the model.

Numbers of all RNA molecules are doubled by RP complexes carrying out transcription. It is assumed that energy and DNA are not needed for RNA synthesis. Transcription is described by following Supplementary Eqs. (136)-(137) that are remarkably similar to Supplementary Eqs. (90)-(91):

$$t_{d\_rs+PW2+rna} = \frac{N_{rrna} \cdot n_{rrna} + N_{trna} \cdot n_{trna} + \sum_{1}^{3} N_{mrna\_cell\_comp} \cdot n_{mrna\_cell\_comp}}{N_{rp} \cdot k_{rp}} \quad (136)$$

$$\sum_{1}^{3} N_{mrna\_cell\_comp} \cdot n_{mrna\_cell\_comp} = N_{mrna\_rp} \cdot n_{mrna\_rp} + N_{mrna\_rpc} \cdot n_{mrna\_rpc} + N_{mrna\_enz} \cdot n_{mrna\_enz} \quad (137)$$

The stoichiometry of the ribosome is described by Supplementary Eq. (92) and tRNA balances by Supplementary Eq. (93). $N_{mrna\_cell\_comp}$ values are determined by translational activity and also by P_{cell_comp} (Supplementary Eqs. (94)-(100)).

Supplementary Discussion 5.11.6.1.4: Mass balance

M_{tot} is equal to M_{cyt} and it is the sum of masses of all cell components including water:

$$M_{cyt} = M_{tot} = \frac{N_{rp} \cdot m_{rp} + N_{rs} \cdot m_{rpc} + N_{trna} \cdot m_{trna} + N_{rrna} \cdot m_{rrna} + m_{enz} \cdot \sum_{i=2,4} N_{enz\_PW_i\_r} \cdot l_{PW_i}}{DWC} + \\ + \frac{\sum_{1}^{3} N_{mrna\_cell\_comp} \cdot m_{mrna\_cell\_comp}}{DWC} \quad (138)$$

$$\sum_{1}^{3} N_{mrna\_cell\_comp} \cdot m_{mrna\_cell\_comp} = N_{mrna\_rp} \cdot m_{mrna\_rp} + N_{mrna\_rpc} \cdot m_{mrna\_rpc} + N_{mrna\_enz} \cdot m_{mrna\_enz} \quad (139)$$

Supplementary Discussion 5.11.6.2: Model parameters

Corresponding input parameters, their values and output parameters of SSPCM-SRS-M (Supplementary Discussions 5.11.2.2, 5.11.2.2.1-5.11.2.2.2) are also used in the current model except t_{d_rs} from SSPCM-RS, t_{d_rs+PW2} and t_{PW2} from SSPCM-RS+AA, following input parameters (Supplementary Tables 31-32) and output parameters:

1. $t_{d\_rs+PW2+rna}$ which is unique only for SSPCM-RS+AA+RNA instead of t_{d_srs-m}.
2. M_{trna} which is calculated from Supplementary Eq. (140):
$$M_{trna} = N_{trna} \cdot m_{trna} \quad (140)$$
3. M_{rrna} which is calculated from Supplementary Eq. (141):
$$M_{rrna} = N_{rrna} \cdot m_{rrna} \quad (141)$$

Supplementary Table 31. Values of doubling times of cell component molecule/complex ($t_{d\_cell\_comp}$).

Cell component	Symbol	Value	Unit
mRNA of the enzyme of metabolic pathways PW_1-PW_5 [31.1]	$t_{d\_mrna\_enz}$	1.13	s
mRNA of RNA polymerase (RP) complex [31.2]	$t_{d\_mrna\_rp}$	4.18	s
mRNA of ribosomal protein complex [31.3]	$t_{d\_mrna\_rpc}$	5.43	s
Ribonucleotide biosynthesis pathway PW_4 [31.4]	t_{d_PW4}	3000	s
RP complex [31.5]	t_{d_rp}	278.7	s or s rp^{-1}
Assembled rRNA complex [31.6]	t_{d_rrna}	114.18	s or s $rrna^{-1}$
tRNA [31.7]	t_{d_trna}	1.93	s or s $trna^{-1}$

[31.1] The value of relative doubling time (doubling time multiplied by the value of P_{enz}) $t_{d\_mrna\_enz}$ is calculated from Supplementary Eq. (40).
[31.2] The value of relative doubling time (doubling time multiplied by the value of P_{rp}) $t_{d\_mrna\_rp}$ is calculated from Supplementary Eq. (38).
[31.3] The value of relative doubling time (doubling time multiplied by the value of P_{rpc}) $t_{d\_mrna\_rpc}$ is calculated from Supplementary Eq. (39).
[31.4] The value of relative doubling time (doubling time of a single enzyme multiplied by the value of l_{PW4}) t_{d_PW4} is calculated from Supplementary Eq. (28).
[31.5] The value of t_{d_rp} is calculated from Supplementary Eq. (27).
[31.6] The value of t_{d_rrna} is calculated from Supplementary Eq. (29).
[31.7] The value of t_{d_trna} is calculated from Supplementary Eq. (30).

Supplementary Table 32. Values of time coefficients of cell component (t_{cell_comp}).

Cell component	Symbol	Value	Unit
Ribonucleotide biosynthesis pathway PW_4 [32.1]	t_{PW4}	600	s or s molecules (aa) (molecules (metabolite) PW_4)$^{-1}$
RP complex [32.2]	t_{rp}	139.35	s or s molecules (aa) (molecules (nt))$^{-1}$
tRNA [32.3]	t_{trna}	19.25	s or s molecules (nt) (molecules (aa))$^{-1}$

[32.1] The value of t_{PW4} is calculated from Supplementary Eq. (34).
[32.2] The value of t_{rp} is calculated from Supplementary Eq. (33).
[32.3] The value of t_{trna} is calculated from Supplementary Eq. (35).

Supplementary Discussion 5.11.6.3: Calculation scheme

The solution of the main equation system of the mathematically determined model is described by Supplementary Eq. (41). As already explained in Supplementary Discussion 5.4, $t_{d\_rs+PW2+rna}$ is completely independent of N_{cell_comp}. Analogically to the previous models (SSPCM-RS, SSPCM-RS+AA), N_{cell_comp} cannot be calculated as it is cancelled out from Supplementary Eq. (41). Therefore, $t_{d\_rs+PW2+rna}$ must be output parameter whereas one N_{cell_comp} must be input parameter (it affects other N_{cell_comp} and M_{tot}).

Supplementary Table 33: Calculated rounded values of selected output parameters of proto-cell SSPCM-RS+AA+RNA at different values of a number of ribosomes in the cell.

Parameter	Symbol	Unit		
Number of ribosomes in the cell	N_{rs}	molecules (rs) cell^{-1}	1	10^4
Doubling time	$t_{d\_rs+PW2+rna}$	s	1127.15	1127.15
Cell mass	M_{tot}	g cell^{-1}	$2.38\cdot10^{-17}$	$2.38\cdot10^{-13}$
Number of cell component molecules/complexes in the cell				
Ribosomes for the synthesis of ribosomal protein complex (RPC)	N_{rs_rpc}	molecules (rs rpc) cell^{-1}	0.32	3213
Ribosomes for the synthesis of enzymes in central and biosynthesis pathways PW_1-PW_5	N_{rs_enz}	molecules (rs enz) cell^{-1}	0.65	6512
Ribosomes for the synthesis of RNA polymerase (RP) complex	N_{rs_rp}	molecules (rs rp) cell^{-1}	0.03	276
RP	N_{rp}	molecules (rp) cell^{-1}	0.11	1116
Enzyme catalysing a single reaction r of amino acid biosynthesis pathway PW_2	$N_{enz\_PW2\_r}$	molecules (enz PW_2) cell^{-1} reaction^{-1}	0.2	2000
Enzyme catalysing a single reaction r of amino acid biosynthesis pathway PW_4	$N_{enz\_PW4\_r}$	molecules (enz PW_4) cell^{-1} reaction^{-1}	0.04	446
Assembled rRNA complex	N_{rrna}	molecules (rrna) cell^{-1}	1	10^4
tRNA	N_{trna}	molecules (trna) cell^{-1}	5	$5\cdot10^4$
mRNA of RPC	N_{mrna_rpc}	molecules (mrna rpc) cell^{-1}	$3\cdot10^{-3}$	32
mRNA of RP	N_{mrna_rp}	molecules (mrna rp) cell^{-1}	$3\cdot10^{-4}$	3
mRNA of the enzyme in central and biosynthesis pathways	N_{mrna_enz}	molecules (mrna enz) cell^{-1}	$7\cdot10^{-3}$	65

SUPPLEMENTARY DISCUSSION 5.11.7: DESCRIPTION OF SSPCM-RS+AA+RNA+LIP

The description of the model (SSPCM-RS+AA+RNA+LIP) is based on the full description of SSPCM-SRS-M (Supplementary Discussion 5.11.2), only the differences compared with SSPCM-SRS-M are subsequently presented.

Supplementary Discussion 5.11.7.1: Model components and interactions

Compared to the previous cases, the proto-cell is surrounded by the lipid membrane which separates the growth environment and cytoplasm (Supplementary Figure 5).

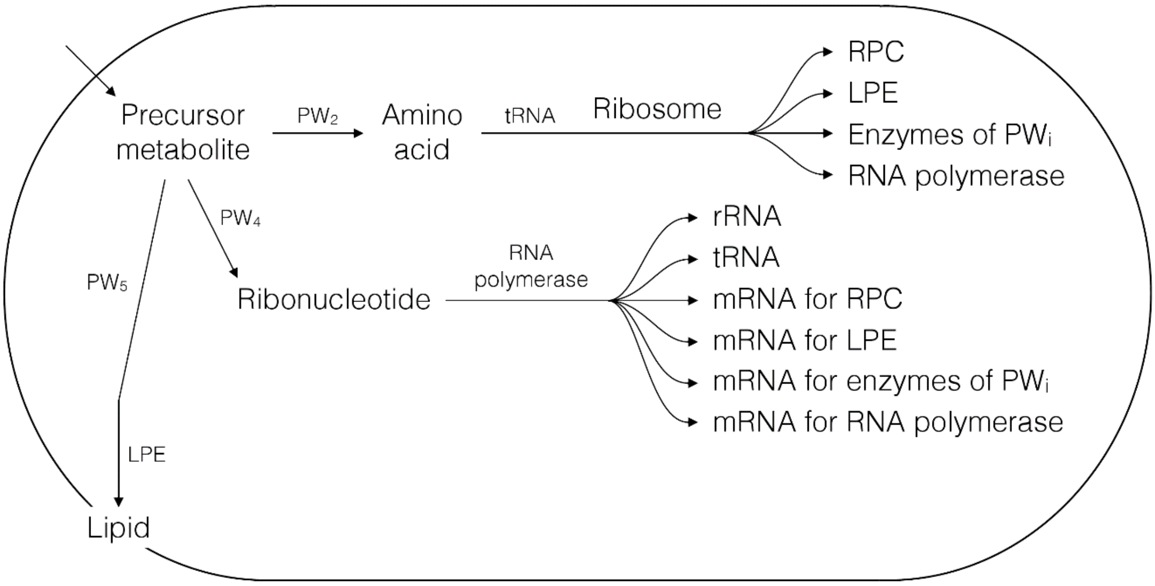

Supplementary Figure 5. Scheme of the bacterial proto-cell SSPCM-RS+AA+RNA+LIP. The SSPCM-RS+AA+RNA+LIP model developed includes ribosomes, RNA polymerase (RP) complexes, lipid synthesis enzyme (LPE), enzymes of the metabolic network, all RNA types (assembled rRNA complex, tRNA, mRNAs of individual proteins), membrane lipids, monomers of proteins (amino acids) and RNA (ribonucleotides), precursor and intermediate metabolites (Supplementary Table 2). Most of the cell components are located in the cytoplasm except membrane lipids that form the bilayer cell membrane (Supplementary Table 1). Ribosome (consisting of rRNA and ribosomal protein complex (RPC)) carries out synthesis of all proteins from amino acids without energy. RP carries out synthesis of all RNA molecules from ribonucleotides without energy and DNA. LPE carries out the synthesis of membrane lipids from metabolic precursors without energy. Monomers (amino acids, ribonucleotides) and metabolic precursor for membrane lipids are synthesized by enzymes in relevant biosynthesis pathways (PW_2, PW_4, PW_5) consisting of linear reaction chains from precursor metabolite (provided by the growth environment) via metabolic intermediates (Supplementary Table 3).

A distantly similar but even more complicated model based on ordinary differential equations depicting coupled translation and transcription auto-catalytic cycles has been proposed[27]. Compared to SSPCM-RS+AA+RNA+LIP (Supplementary Figure 5), the model[27] has an elaborate and dynamic description of polymerization including assembly and degradation of proteins, tRNA synthesis and charging, resting pools of macromolecules, membrane synthesis etc whereas the synthesis of monomers and geometry of the proto-cell have not been included.

Supplementary Discussion 5.11.7.1.1: Metabolic network

It is assumed that the proto-cell converts the same unspecified metabolic intermediate (available in the surrounding environment) to monomers (ribonucleotide, amino acid, lipid) of macromolecules via monomer synthesis pathways PW_i for each monomer (Supplementary Figure 5). All reactions are catalysed by identical (*generic* m_{enz}, *generic* n_{enz}) enzymes with the same *generic* k_{enz} as in SSPCM-SRS-M (Supplementary Discussion 5.11.2.1.1). Therefore, Supplementary Eqs. (73), (75)-(76) describe all fluxes of all reactions for all metabolic intermediates in PW_i.

Supplementary Discussion 5.11.7.1.2: Protein synthesis

The protein fraction of the proto-cell is comprised of the following different proteins (Supplementary Figure 5, Supplementary Table 2):

1. RP complex (carries out transcription).
2. RPC (part of ribosomes, carries out translation).
3. Enzymes on biosynthetic pathways PW_2, PW_4 and PW_5 (carry out the synthesis of metabolic intermediates and monomers of macromolecules).
4. LPE (membrane lipid synthesis).

The numbers of all proteins are doubled by ribosomes carrying out translation. It is assumed that energy is not needed for protein synthesis. $N_{rs\_cell\_comp}$ is determined by $t_{d\_rs+PW2+rna+lip}$ and the requirement of amino acids of each protein according to Supplementary Eq. (80) and following Supplementary Eqs. (142)-(144) that are remarkably similar to Supplementary Eqs. (82)-(84):

$$t_{d\_rs+PW2+rna+lip} = \frac{n_{enz} \cdot \sum_{i=2,4,5} N_{enz\_PWi\_r} \cdot l_{PWi}}{N_{rs\_enz} \cdot k_{rs}} \quad (142)$$

$$t_{d\_rs+PW2+rna+lip} = \frac{N_{rp} \cdot n_{rp}}{N_{rs\_rp} \cdot k_{rs}} \quad (143)$$

$$t_{d\_rs+PW2+rna+lip} = \frac{N_{lpe} \cdot n_{lpe}}{N_{rs\_lpe} \cdot k_{rs}} \quad (144)$$

N_{rs} is expressed by the following Supplementary Eq. (145):

$$N_{rs} = N_{rs\_rp} + N_{rs\_rpc} + N_{rs\_enz} + N_{rs\_lpe} \quad (145)$$

Supplementary Discussion 5.11.7.1.3: RNA synthesis

RNA fraction of the proto-cell is comprised of the following different RNAs (Supplementary Figure 5, Supplementary Table 2):

1. Ribosomal RNA complex is based on 3 subunits (5S, 16S, 23S) assembled in ribosomes with 1:1:1 stoichiometry.
2. Transfer RNA. The model was simplified by introducing only one universal tRNA molecule because different amino acids are not specified.
3. There are specific mRNAs coding different proteins in the model. It is assumed that mRNAs contain only coding regions to simplify the model.

Numbers of all RNA molecules are doubled by RP complexes carrying out transcription. It is assumed that energy and DNA are not needed for RNA synthesis. Transcription is described by following Supplementary Eqs. (146)-(147) that are remarkably similar to Supplementary Eqs. (90)-(91):

$$t_{d\_rs+PW2+rna+lip} = \frac{N_{rrna} \cdot n_{rrna} + N_{trna} \cdot n_{trna} + \sum_{1}^{4} N_{mrna\_cell\_comp} \cdot n_{mrna\_cell\_comp}}{N_{rp} \cdot k_{rp}} \quad (146)$$

$$\sum_{1}^{4} N_{mrna\_cell\_comp} \cdot n_{mrna\_cell\_comp} = N_{mrna\_rp} \cdot n_{mrna\_rp} + N_{mrna\_rpc} \cdot n_{mrna\_rpc} + N_{mrna\_enz} \cdot n_{mrna\_enz} + \\ + N_{mrna\_lpe} \cdot n_{mrna\_lpe} \quad (147)$$

The stoichiometry of the ribosome is described by Supplementary Eq. (92) and tRNA balances by Supplementary Eq. (93). $N_{mrna\_cell\_comp}$ values are determined by translational activity and also by P_{cell_comp} (Supplementary Eqs. (94)-(100)).

Supplementary Discussion 5.11.7.1.4: Lipid synthesis

Proto-cell contains only one type of membrane lipid that forms a bilayer cell membrane (Supplementary Figure 5, Supplementary Tables 1-2). The value of N_{lip} is doubled by LPE according to the following Supplementary Eq. (148) which is remarkably similar to Supplementary Eq. (101):

$$t_{d\_rs+PW2+rna+lip} = \frac{N_{lip}}{N_{lpe} \cdot k_{lpe}} \quad (148)$$

Supplementary Discussion 5.11.7.1.5: Cell geometry

It is assumed that the cell shape is an ideal cylinder with spherical caps (Supplementary Figure 5, Supplementary Table 3) as in SSPCM-SRS-M (Supplementary Discussion 5.11.2.1.6). The inner surface area of the cell membrane equals the outer surface area to simplify the model. S_{tot} depends on V_{cyt} and S_{tot} is the sum of surface areas of membrane lipids (S_{lip}) according to the following Supplementary Eq. (149):

$$N_{lip} \cdot s_{lip} = \left(\frac{V_{cyt}}{\frac{4}{3} + HR} \right)^{\frac{2}{3}} \cdot \left(4 + 2 \cdot HR\right) \cdot \pi^{\frac{1}{3}} \quad (149)$$

V_{cyt} is equal to the M_{cyt} assuming that the density of cytoplasm is identical to *generic* ρ_{tot} and equal to water density (Supplementary Eq. (104)).

Supplementary Discussion 5.11.7.1.6: Mass balance

M_{tot} can be divided roughly into two parts by cell structures – M_{cyt} and M_{mem} – and it is the sum of masses of all cell components including water (Supplementary Tables 1-2):

$$M_{tot} = M_{cyt} + \frac{N_{lip} \cdot m_{lip}}{DWC} \quad (150)$$

The M_{cyt} equals the sum of masses of all cell components (including water) localized in cytoplasmic space:

$$M_{cyt} = \frac{N_{rp} \cdot m_{rp} + N_{rs} \cdot m_{rpc} + N_{trna} \cdot m_{trna} + N_{rrna} \cdot m_{rrna} + m_{enz} \cdot \sum_{i=2,4,5} N_{enz\_PW_i\_r} \cdot l_{PW_i}}{DWC} + \\ + \frac{\sum_{1}^{4} N_{mrna\_cell\_comp} \cdot m_{mrna\_cell\_comp} + N_{lpe} \cdot m_{lpe}}{DWC} \quad (151)$$

$$\sum_{1}^{4} N_{mrna\_cell\_comp} \cdot m_{mrna\_cell\_comp} = N_{mrna\_rp} \cdot m_{mrna\_rp} + N_{mrna\_rpc} \cdot m_{mrna\_rpc} + N_{mrna\_lpe} \cdot m_{mrna\_lpe} + \\ + N_{mrna\_enz} \cdot m_{mrna\_enz} \quad (152)$$

Supplementary Discussion 5.11.7.2: Model parameters

Corresponding input parameters, their values and output parameters of SSPCM-SRS-M (Supplementary Discussions 5.11.2.2, 5.11.2.2.1-5.11.2.2.2) are also used in the current model except input parameter $t_{d\_rs+PW2+rna+lip}$ (independent variable) which is unique only for SSPCM-RS+AA+RNA+LIP instead of $t_{d\_srs\text{-}m}$.

Supplementary Discussion 5.11.7.3: Calculation scheme

The calculation scheme is similar but not identical to SSPCM-SRS-M (Supplementary Discussion 5.11.2.3). The first step (solving of independent equations) involves only the calculation of masses of cell components (Supplementary Tables 16-19), lengths of mRNA sequences (Supplementary Table 10) etc. All above (Supplementary Discussions 5.11.7.1.1-5.11.7.1.6) referred and presented equations comprise the main system of equations solved during the second step.

Supplementary Table 34: Calculated rounded values of selected output parameters of proto-cell SSPCM-RS+AA+RNA+LIP at different doubling time values.

Parameter	Symbol	Unit				
Doubling time	$t_{d\_rs+PW2+rna+lip}$	s	1127.15	1140	1400	3520
Number of cell component molecules/complexes in the cell						
Ribosome	N_{rs}	molecules (rs) cell^{-1}	$6.19 \cdot 10^{14}$	23753	1.88	$9 \cdot 10^{-4}$
Ribosomes for the synthesis of ribosomal protein complex (RPC)	N_{rs_rpc}	molecules (rs rpc) cell^{-1}	$1.99 \cdot 10^{14}$	7545	0.49	$9 \cdot 10^{-5}$
Ribosomes for the synthesis of enzymes in central and biosynthesis pathways PW_1-PW_5	N_{rs_enz}	molecules (rs enz) cell^{-1}	$4.03 \cdot 10^{14}$	15566	1.36	$8 \cdot 10^{-4}$
Ribosomes for the synthesis of RNA polymerase (RP) complex	N_{rs_rp}	molecules (rs rp) cell^{-1}	$1.71 \cdot 10^{13}$	641	0.03	$2 \cdot 10^{-6}$
Ribosomes for the synthesis of lipid synthesis enzyme (LPE)	N_{rs_lpe}	molecules (rs lpe) cell^{-1}	$1.37 \cdot 10^{7}$	2	$2 \cdot 10^{-3}$	$3 \cdot 10^{-6}$
RP	N_{rp}	molecules (rp) cell^{-1}	$6.91 \cdot 10^{13}$	2621	0.17	$3 \cdot 10^{-5}$
LPE	N_{lpe}	molecules (lpe) cell^{-1}	$1.03 \cdot 10^{9}$	116	0.19	$7 \cdot 10^{-4}$
Enzyme catalysing a single reaction r of deoxyribonucleotide biosynthesis pathway PW_2	$N_{enz\_PW2\_r}$	molecules (enz PW_2) cell^{-1} reaction^{-1}	$1.24 \cdot 10^{14}$	4751	0.38	$2 \cdot 10^{-4}$
Enzyme catalysing a single reaction r of ribonucleotide biosynthesis pathway PW_4	$N_{enz\_PW4\_r}$	molecules (enz PW_4) cell^{-1} reaction^{-1}	$2.76 \cdot 10^{13}$	1048	0.07	10^{-5}
Enzyme catalysing a single reaction r of lipid biosynthesis pathway PW_5	$N_{enz\_PW5\_r}$	molecules (enz PW_5) cell^{-1} reaction^{-1}	$1.03 \cdot 10^{9}$	116	0.19	$7 \cdot 10^{-4}$
Assembled rRNA complex	N_{rrna}	molecules (rrna) cell^{-1}	$6.19 \cdot 10^{14}$	23753	1.88	$9 \cdot 10^{-4}$
tRNA	N_{trna}	molecules (trna) cell^{-1}	$3.1 \cdot 10^{15}$	118767	9.40	$4 \cdot 10^{-3}$
mRNA of RPC	N_{mrna_rpc}	molecules (mrna rpc) cell^{-1}	$1.99 \cdot 10^{12}$	75	$5 \cdot 10^{-3}$	$9 \cdot 10^{-7}$

mRNA of RP	N_{mrna_rp}	molecules (mrna rp) cell^{-1}	$1.71\cdot10^{11}$	6	$3\cdot10^{-4}$	$2\cdot10^{-8}$
mRNA of the enzyme in central and biosynthesis pathways PW_1-PW_5	N_{mrna_enz}	molecules (mrna enz) cell^{-1}	$4.03\cdot10^{12}$	156	0.01	$8\cdot10^{-6}$
mRNA of LPE	N_{mrna_lpe}	molecules (mrna lpe) cell^{-1}	683881	0.08	10^{-4}	$2\cdot10^{-7}$
Membrane lipid	N_{lip}	molecules (lip) cell^{-1}	$1.16\cdot10^{14}$	$1.32\cdot10^{7}$	26674	255
Size parameters						
Mass of cytoplasm of the cell	M_{cyt}	g cyt^{-1}	0.01472	$5.69\cdot10^{-13}$	$5.14\cdot10^{-17}$	$4.82\cdot10^{-20}$
Cell mass	M_{tot}	g cell^{-1}	0.01472	$5.97\cdot10^{-13}$	$1.09\cdot10^{-16}$	$5.96\cdot10^{-19}$

SUPPLEMENTARY DISCUSSION 5.11.8: DESCRIPTION OF SSPCM-RS+LIP

The description of the model (SSPCM-RS+LIP) is based on the full description of SSPCM-SRS-M (Supplementary Discussion 5.11.2), only the differences compared with SSPCM-SRS-M are subsequently presented.

Supplementary Discussion 5.11.8.1: Model components and interactions

The proto-cell is surrounded by the lipid membrane which separates the growth environment and cytoplasm consisting of only RPC and LPE (Supplementary Figure 6).

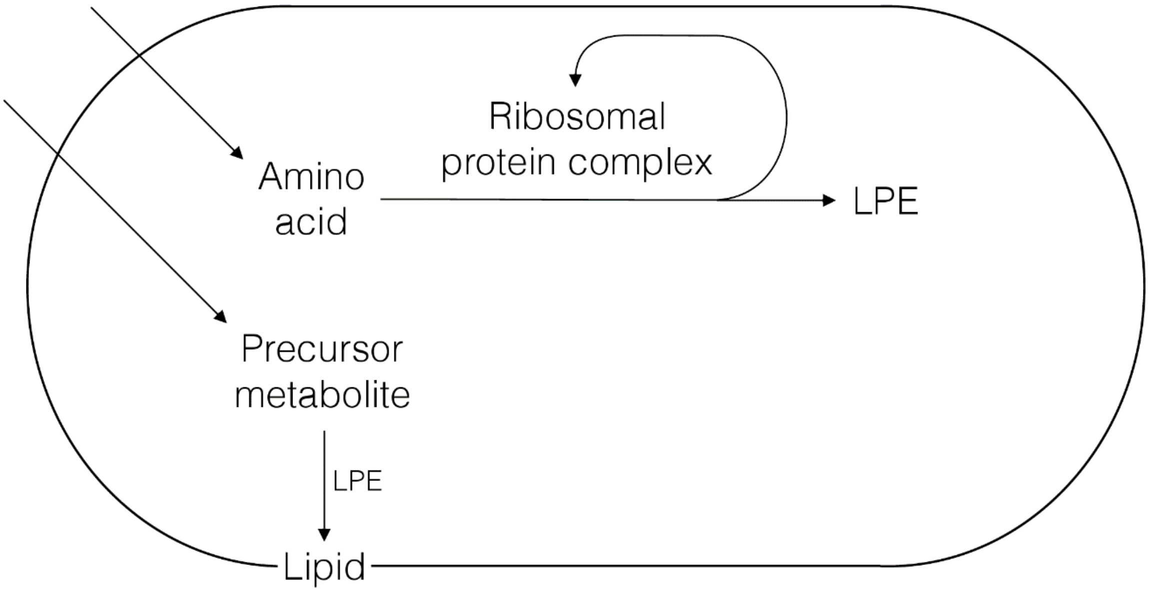

Supplementary Figure 6. Scheme of the bacterial proto-cell SSPCM-RS+LIP. The SSPCM-RS+LIP model developed includes ribosomal protein complex (RPC), lipid synthesis enzyme (LPE), membrane lipids, monomers of proteins (amino acids) and precursor metabolite (Supplementary Table 2). RPC and LPE are located in the cytoplasm, membrane lipids form the bilayer cell membrane (Supplementary Table 1). RPC carries out synthesis of all proteins from amino acids (provided by the growth environment) without energy and RNA. LPE carries out the synthesis of membrane lipids from metabolic precursors (provided by the growth environment) without energy (Supplementary Table 3).

Supplementary Discussion 5.11.8.1.1: Protein synthesis

The protein fraction of the proto-cell is comprised of the following different proteins (Supplementary Figure 6, Supplementary Table 2):

1. RPC (part of ribosomes, carries out translation).
2. LPE (membrane lipid synthesis).

The numbers of all proteins are doubled by ribosomes carrying out translation. It is assumed that rRNA, mRNAs, tRNA and energy are not needed for protein synthesis and an unlimited number of amino acids are available in the surrounding environment. $N_{rs\_cell\_comp}$ is determined by t_{d_rs+lip} and the requirement of amino acids of each protein according to Supplementary Eq. (80) and following Supplementary Eq. (153) that is remarkably similar to Supplementary Eq. (84):

$$t_{d\_rs+lip} = \frac{N_{lpe} \cdot n_{lpe}}{N_{rs\_lpe} \cdot k_{rs}} \tag{153}$$

N_{rs} is expressed by the following Supplementary Eq. (154):

$$N_{rs} = N_{rs\_rpc} + N_{rs\_lpe} \tag{154}$$

Supplementary Discussion 5.11.8.1.2: Lipid synthesis

Proto-cell contains only one type of membrane lipid that forms a bilayer cell membrane (Supplementary Figure 6, Supplementary Tables 1-2). The value of N_{lip} is doubled by LPE assuming that an unlimited number of lipids are available in the surrounding environment. The membrane lipid synthesis is described according to the following Supplementary Eq. (155) which is remarkably similar to Supplementary Eq. (101):

$$t_{d\_rs+lip} = \frac{N_{lip}}{N_{lpe} \cdot k_{lpe}} \tag{155}$$

Supplementary Discussion 5.11.8.1.3: Cell geometry

The geometry of the proto-cell is the same as in Supplementary Discussion 5.11.7.1.5.

Supplementary Discussion 5.11.8.1.4: Mass balance

M_{tot} is described by Supplementary Eq. (150). M_{cyt} equals the sum of masses of all cell components (including water) localized in cytoplasmic space:

$$M_{cyt} = \frac{N_{rs} \cdot m_{rpc} + N_{lpe} \cdot m_{lpe}}{DWC} \tag{156}$$

Supplementary Discussion 5.11.8.2: Model parameters

Corresponding input parameters, their values and output parameters of SSPCM-SRS-M (Supplementary Discussions 5.11.2.2, 5.11.2.2.1-5.11.2.2.2) are also used in the current model except:

1. Input parameter t_{d_rs+lip} (independent variable) which is unique only for SSPCM-RS+LIP instead of $t_{d\_srs\text{-}m}$.
2. Input parameter t_{lpe} = 3 s or s molecules (aa) (molecules (lip))$^{-1}$ calculated from Supplementary Eq. (64).

Supplementary Discussion 5.11.8.3: Calculation scheme

The calculation scheme is similar but not identical to SSPCM-SRS-M (Supplementary Discussion 5.11.2.3). The first step (solving of independent equations) involves only the calculation of masses of cell components (Supplementary Tables 16-18) etc. All above (Supplementary Discussions 5.11.8.1.1-5.11.8.1.4) referred and presented equations comprise the main system of equations solved during the second step.

Supplementary Table 35: Calculated rounded values of selected output parameters of proto-cell SSPCM-RS+LIP at different doubling time values.

Parameter	Symbol	Unit				
Doubling time	t_{d_rs+lip}	s	362.1	362.18	364	3520
Number of cell component molecules/complexes in the cell						
Ribosome	N_{rs}	molecules (rs) cell^{-1}	$1.16 \cdot 10^{28}$	22604	1.68	$4 \cdot 10^{-11}$
Ribosomes for the synthesis of ribosomal protein complex	N_{rs_rpc}	molecules (rs rpc) cell^{-1}	$1.16 \cdot 10^{28}$	22599	1.67	$4 \cdot 10^{-12}$
Ribosomes for the synthesis of lipid synthesis enzyme (LPE)	N_{rs_lpe}	molecules (rs lpe) cell^{-1}	$3.20 \cdot 10^{16}$	5	$9 \cdot 10^{-3}$	$4 \cdot 10^{-11}$
LPE	N_{lpe}	molecules (lpe) cell^{-1}	$7.72 \cdot 10^{17}$	121	0.21	$8 \cdot 10^{-9}$
Membrane lipid	N_{lip}	molecules (lip) cell^{-1}	$2.79 \cdot 10^{22}$	$4.37 \cdot 10^{6}$	7741	$3 \cdot 10^{-3}$
Size parameters						
Mass of cytoplasm of the cell	M_{cyt}	g cyt^{-1}	$5.52 \cdot 10^{10}$	$1.08 \cdot 10^{-13}$	$8.04 \cdot 10^{-18}$	$1.75 \cdot 10^{-27}$
Cell mass	M_{tot}	g cell^{-1}	$5.52 \cdot 10^{10}$	$1.17 \cdot 10^{-13}$	$2.47 \cdot 10^{-17}$	$6.02 \cdot 10^{-24}$

SUPPLEMENTARY DISCUSSION 5.11.9: DESCRIPTION OF SSPCM-RS+RNA+LIP+MPROT

The description of the model (SSPCM-RS+AA+RNA+LIP+MPROT) is based on the full description of SSPCM-SRS-M (Supplementary Discussion 5.11.2), only the differences compared with SSPCM-SRS-M are subsequently presented.

Supplementary Discussion 5.11.9.1: Model components and interactions

The proto-cell is surrounded by the lipid membrane which separates the growth environment and cytoplasm (Supplementary Figure 7).

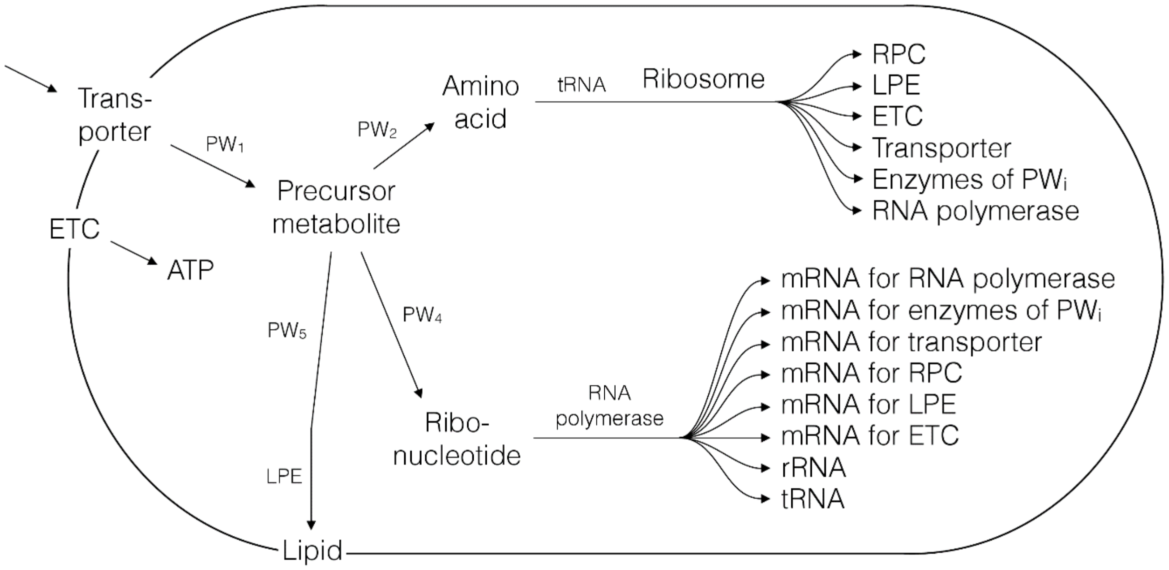

Supplementary Figure 7. Scheme of the bacterial proto-cell SSPCM-RS+AA+RNA+LIP+MPROT. The SSPCM-RS+AA+RNA+LIP+MPROT model developed includes ribosomes, RNA polymerase (RP) complexes, lipid synthesis enzyme (LPE), substrate transporter protein, electron transport chain (ETC) complex, enzymes of the metabolic network, all RNA types (assembled rRNA complex, tRNA, mRNAs of individual proteins), membrane lipids, monomers of proteins (amino acids) and RNA (ribonucleotides), precursor and intermediate metabolites, ATP (Supplementary Table 2). Most of the cell components are located in the cytoplasm except transport protein, ETC and membrane lipids that form the bilayer cell membrane (Supplementary Table 1). Ribosome (consisting of rRNA and ribosomal protein complex (RPC)) carries out synthesis of all proteins from amino acids. RP carries out synthesis of all RNA molecules from ribonucleotides without DNA. LPE carries out the synthesis of membrane lipids from metabolic precursors. Monomers (amino acids, ribonucleotides) and metabolic precursor for membrane lipids are synthesized by enzymes in relevant biosynthesis pathways (PW_2, PW_4, PW_5) consisting of linear reaction chains from precursor metabolite (provided by the growth environment) via metabolic intermediates. An unspecified substrate is transported through the membrane by a membrane transporter and is converted to unspecified metabolic intermediates via a series of reactions of the central catabolic pathway PW_1. Energy is synthesized by the ETC complex on the membrane (Supplementary Table 3).

Supplementary Discussion 5.11.9.1.1: Metabolic network

It is assumed that the proto-cell converts the same unspecified metabolic intermediate to monomers (ribonucleotide, amino acid, lipid) of macromolecules via monomer synthesis pathways PW_i for each monomer (Supplementary Figure 7, Supplementary Tables 2-3). All reactions are catalysed by identical (*generic* m_{enz}, *generic* n_{enz}) enzymes with the same *generic* k_{enz} as in SSPCM-SRS-M (Supplementary Discussion 5.11.2.1.1). Therefore, Supplementary Eq. (73), (75)-(76) describe all fluxes of all reactions for all metabolic intermediates in PW_i.
Biosynthesis pathways PW_2, PW_4 and PW_5 are branching off from the central pathway PW_1 from the same intermediate (Supplementary Figure 7, Supplementary Tables 2-3). Again, it is assumed that the topology of PW_1 is similar to those of biosynthesis pathways and that reactions of PW_1 are catalysed by identical enzymes (as in PW_2, PW_4 and PW_5). Therefore, the flux balances of the whole pathway are again described by the flux balance of one reaction. Parameter k_{enz} can be excluded from the flux balance equation due to identical enzymes for all pathways:

$$N_{enz\_PW1\_r} = N_{enz\_PW2\_r} + N_{enz\_PW4\_r} + N_{enz\_PW5\_r} \tag{157}$$

Substrate transport and consumption by central metabolic pathway PW_1 is described by Supplementary Eq. (78).

Supplementary Discussion 5.11.9.1.2: Protein synthesis

The protein fraction of the proto-cell is comprised of the following different proteins (Supplementary Figure 7, Supplementary Table 2):

1. RP complex (carries out transcription).
2. RPC (part of ribosomes, carries out translation).
3. Enzymes on biosynthetic pathways PW_1, PW_2, PW_4 and PW_5 (carry out the synthesis of metabolic intermediates and monomers of macromolecules).
4. LPE (membrane lipid synthesis).
5. ETC complex (energy production on the membrane).
6. Transport protein (substrate transport on the membrane).

The numbers of all proteins are doubled by ribosomes carrying out translation. $N_{rs\_cell\_comp}$ is determined by $t_{d\_rs+PW2+rna+lip+mprot}$ and the requirement of amino acids of each protein according to Supplementary Eq. (80) and following Supplementary Eqs. (158)-(162) that are remarkably similar to Supplementary Eqs. (82)-(86):

$$t_{d\_rs+PW2+rna+lip+mprot} = \frac{n_{enz} \cdot \sum_{i=1,2,4,5} N_{enz\_PWi\_r} \cdot l_{PWi}}{N_{rs\_enz} \cdot k_{rs}} \tag{158}$$

$$t_{d\_rs+PW2+rna+lip+mprot} = \frac{N_{rp} \cdot n_{rp}}{N_{rs\_rp} \cdot k_{rs}} \tag{159}$$

$$t_{d\_rs+PW2+rna+lip+mprot} = \frac{N_{lpe} \cdot n_{lpe}}{N_{rs\_lpe} \cdot k_{rs}} \tag{160}$$

$$t_{d\_rs+PW2+rna+lip+mprot} = \frac{N_{etc} \cdot n_{etc}}{N_{rs\_etc} \cdot k_{rs}} \tag{161}$$

$$t_{d\_rs+PW2+rna+lip+mprot} = \frac{N_{stp} \cdot n_{stp}}{N_{rs\_stp} \cdot k_{rs}} \tag{162}$$

N_{rs} is expressed by the following Supplementary Eq. (163):

$$N_{rs} = N_{rs\_rp} + N_{rs\_rpc} + N_{rs\_etc} + N_{rs\_stp} + N_{rs\_lpe} + N_{rs\_enz} \tag{163}$$

Supplementary Discussion 5.11.9.1.3: RNA synthesis

RNA fraction of the proto-cell is comprised of the following different RNAs (Supplementary Figure 7, Supplementary Table 2):

1. Ribosomal RNA complex is based on 3 subunits (5S, 16S, 23S) assembled in ribosomes with 1:1:1 stoichiometry.
2. Transfer RNA. The model was simplified by introducing only one universal tRNA molecule because different amino acids are not specified.
3. There are specific mRNAs coding different proteins in the model. It is assumed that mRNAs contain only coding regions to simplify the model.

Numbers of all RNA molecules are doubled by RP complexes carrying out transcription. It is assumed that DNA is not needed for RNA synthesis. Transcription is described by following Supplementary Eqs. (164)-(165) that are remarkably similar to Supplementary Eqs. (90)-(91):

$$t_{d\_rs+PW2+rna+lip+mprot} = \frac{N_{rrna} \cdot n_{rrna} + N_{trna} \cdot n_{trna} + \sum_{1}^{6} N_{mrna\_cell\_comp} \cdot n_{mrna\_cell\_comp}}{N_{rp} \cdot k_{rp}} \quad (164)$$

$$\sum_{1}^{6} N_{mrna\_cell\_comp} \cdot n_{mrna\_cell\_comp} = N_{mrna\_rp} \cdot n_{mrna\_rp} + N_{mrna\_rpc} \cdot n_{mrna\_rpc} + N_{mrna\_etc} \cdot n_{mrna\_etc} + \\ + N_{mrna\_stp} \cdot n_{mrna\_stp} + N_{mrna\_lpe} \cdot n_{mrna\_lpe} + N_{mrna\_enz} \cdot n_{mrna\_enz} \quad (165)$$

The stoichiometry of the ribosome is described by Supplementary Eq. (92) and tRNA balances by Supplementary Eq. (93). $N_{mrna\_cell\_comp}$ values are determined by translational activity and also by P_{cell_comp} (Supplementary Eqs. (94)-(100)).

Supplementary Discussion 5.11.9.1.4: Lipid synthesis

Proto-cell contains only one type of membrane lipid that forms a bilayer cell membrane (Supplementary Figure 7, Supplementary Tables 1-2). The value of N_{lip} is doubled by LPE according to the following Supplementary Eq. (166) which is remarkably similar to Supplementary Eq. (101):

$$t_{d\_rs+PW2+rna+lip+mprot} = \frac{N_{lip}}{N_{lpe} \cdot k_{lpe}} \quad (166)$$

Supplementary Discussion 5.11.9.1.5: Energy balance

ETC on the cell membrane (Supplementary Table 1) must produce the exact amount of ATP that is consumed by different monomer biosynthesis pathways, polymerization processes and substrate transport (Supplementary Tables 2-3) to accomplish the doubling condition described by following Supplementary Eq. (167):

$$N_{etc} \cdot k_{etc} = N_{stp} \cdot k_{stp} \cdot X_{stp} + N_{rp} \cdot k_{rp} \cdot X_{rna} + N_{lpe} \cdot k_{lpe} \cdot X_{lip} + N_{rs} \cdot k_{rs} \cdot X_{prot} + k_{enz} \sum_{i=2,4,5} N_{enz\_PW_i\_r} \cdot X_{PW_i} \quad (167)$$

Supplementary Discussion 5.11.9.1.6: Cell geometry

The geometry of the proto-cell is the same as in SSPCM-SRS-M (Supplementary Discussion 5.11.2.1.6).

Supplementary Discussion 5.11.9.1.7: Mass balance

M_{tot} is described by Supplementary Eq. (105). The M_{cyt} equals the sum of masses of all cell components (including water) localized in cytoplasmic space:

$$M_{cyt} = \frac{N_{rp} \cdot m_{rp} + N_{rs} \cdot m_{rpc} + N_{trna} \cdot m_{trna} + N_{rrna} \cdot m_{rrna} + m_{enz} \cdot \sum_{i=1,2,4,5} N_{enz\_PW_i\_r} \cdot l_{PW_i}}{DWC} +$$

$$+ \frac{\sum_{1}^{6} N_{mrna\_cell\_comp} \cdot m_{mrna\_cell\_comp} + N_{lpe} \cdot m_{lpe}}{DWC}$$

(168)

$$\sum_{1}^{6} N_{mrna\_cell\_comp} \cdot m_{mrna\_cell\_comp} = N_{mrna\_rp} \cdot m_{mrna\_rp} + N_{mrna\_rpc} \cdot m_{mrna\_rpc} + N_{mrna\_etc} \cdot m_{mrna\_etc} +$$

$$+ N_{mrna\_stp} \cdot m_{mrna\_stp} + N_{mrna\_lpe} \cdot m_{mrna\_lpe} + N_{mrna\_enz} \cdot m_{mrna\_enz}$$

(169)

Supplementary Discussion 5.11.9.2: Model parameters

Corresponding input parameters, their values and output parameters of SSPCM-SRS-M (Supplementary Discussions 5.11.2.2, 5.11.2.2.1-5.11.2.2.2) are also used in the current model except input parameter $t_{d\_rs+PW2+rna+lip+mprot}$ (independent variable) which is unique only for SSPCM-RS+AA+RNA+LIP+MPROT instead of $t_{d\_srs\text{-}m}$.

Supplementary Discussion 5.11.9.3: Calculation scheme

The calculation scheme is similar but not identical to SSPCM-SRS-M (Supplementary Discussion 5.11.2.3). The first step (solving of independent equations) involves only the calculation of masses of cell components (Supplementary Tables 16-19), lengths of mRNA sequences (Supplementary Table 10) etc. All above (Supplementary Discussions 5.11.9.1.1-5.11.9.1.7) referred and presented equations comprise the main system of equations solved during the second step.

Supplementary Table 36: Calculated rounded values of selected output parameters of proto-cell SSPCM-RS+AA+RNA+LIP+MPROT at different doubling time values.

Parameter	Symbol	Unit				
Doubling time	$t_{d\_rs+PW2+rna+lip+mprot}$	s	2462.68	2469	3100	3520
Number of cell component molecules/complexes in the cell						
Ribosome	N_{rs}	molecules (rs) cell^{-1}	36699	18369	1.29	0.24
Ribosomes for the synthesis of ribosomal protein complex (RPC)	N_{rs_rpc}	molecules (rs rpc) cell^{-1}	5396	2694	0.15	0.03
Ribosomes for the synthesis of enzymes in central and biosynthesis pathways PW_1-PW_5	N_{rs_enz}	molecules (rs enz) cell^{-1}	19727	9873	0.69	0.13
Ribosomes for the synthesis of RNA polymerase (RP) complex	N_{rs_rp}	molecules (rs rp) cell^{-1}	214	107	$5 \cdot 10^{-3}$	$7 \cdot 10^{-4}$
Ribosomes for the synthesis of lipid synthesis enzyme (LPE)	N_{rs_lpe}	molecules (rs lpe) cell^{-1}	10^{-5}	0.07	$4 \cdot 10^{-4}$	10^{-4}
Ribosomes for the synthesis of electron transport chain (ETC)	N_{rs_etc}	molecules (rs etc) cell^{-1}	11033	5531	0.43	0.09
Ribosomes for the synthesis of substrate transport protein	N_{rs_stp}	molecules (rs stp) cell^{-1}	329	165	0.01	$2 \cdot 10^{-3}$
RP	N_{rp}	molecules (rp) cell^{-1}	1893	945	0.05	$9 \cdot 10^{-3}$
LPE	N_{lpe}	molecules (lpe) cell^{-1}	$2 \cdot 10^{-3}$	11	0.08	0.02
ETC	N_{etc}	molecules (etc) cell^{-1}	54342	27310	2.69	0.60
Substrate transport protein	N_{stp}	molecules (stp) cell^{-1}	8097	4063	0.36	0.08
Enzyme catalysing a single reaction r of amino acid biosynthesis pathway PW_1	$N_{enz\_PW1\_r}$	molecules (enz PW_1) cell^{-1} reaction^{-1}	8097	4063	0.36	0.08
Enzyme catalysing a single reaction r of deoxyribonucleotide biosynthesis pathway PW_2	$N_{enz\_PW2\_r}$	molecules (enz PW_2) cell^{-1} reaction^{-1}	7340	3674	0.26	0.05

Enzyme catalysing a single reaction r of ribonucleotide biosynthesis pathway PW_4	$N_{enz\_PW4\_r}$	molecules (enz PW_4) cell^{-1} reaction^{-1}	757	378	0.02	$4 \cdot 10^{-3}$
Enzyme catalysing a single reaction r of lipid biosynthesis pathway PW_5	$N_{enz\_PW5\_r}$	molecules (enz PW_5) cell^{-1} reaction^{-1}	$2 \cdot 10^{-3}$	11	0.08	0.02
Assembled rRNA complex	N_{rrna}	molecules (rrna) cell^{-1}	36699	18369	1.29	0.24
tRNA	N_{trna}	molecules (trna) cell^{-1}	183496	91845	6	1
mRNA of RPC	N_{mrna_rpc}	molecules (mrna rpc) cell^{-1}	54	27	$2 \cdot 10^{-3}$	$3 \cdot 10^{-4}$
mRNA of RP	N_{mrna_rp}	molecules (mrna rp) cell^{-1}	2	1	$5 \cdot 10^{-5}$	$7 \cdot 10^{-6}$
mRNA of the enzyme in central and biosynthesis pathways	N_{mrna_enz}	molecules (mrna enz) cell^{-1}	197	99	$7 \cdot 10^{-3}$	10^{-3}
mRNA of LPE	N_{mrna_lpe}	molecules (mrna lpe) cell^{-1}	$7 \cdot 10^{-3}$	$3 \cdot 10^{-3}$	$2 \cdot 10^{-5}$	$5 \cdot 10^{-6}$
mRNA of ETC	N_{mrna_etc}	molecules (mrna etc) cell^{-1}	110	55	$4 \cdot 10^{-3}$	$9 \cdot 10^{-3}$
mRNA of substrate transport protein	N_{mrna_stp}	molecules (mrna stp) cell^{-1}	3	2	10^{-4}	$2 \cdot 10^{-5}$
Membrane lipid	N_{lip}	molecules (lip) cell^{-1}	528	$2.72 \cdot 10^{6}$	23751	8327
Size parameters						
Mass of cytoplasm of the cell	M_{cyt}	g cyt^{-1}	$1.15 \cdot 10^{-12}$	$5.78 \cdot 10^{-13}$	$4.61 \cdot 10^{-17}$	$9.35 \cdot 10^{-18}$
Cell mass	M_{tot}	g cell^{-1}	$1.52 \cdot 10^{-12}$	$7.69 \cdot 10^{-13}$	$1.15 \cdot 10^{-16}$	$3.13 \cdot 10^{-17}$

SUPPLEMENTARY DISCUSSION 5.11.10: DESCRIPTION OF SSPCM-RS+DNA

The description of the model (SSPCM-RS+DNA) is based on the full description of SSPCM-SRS-M (Supplementary Discussion 5.11.2), only the differences compared with SSPCM-SRS-M are subsequently presented.

Supplementary Discussion 5.11.10.1: Model components and interactions

It is assumed that the proto-cell has no structures and it is only comprised of DNA, RC, RPC and deoxyribonucleotide synthesis enzymes as the only macromolecular cell components (Supplementary Figure 8). It is not specified how cell components are secluded from the surrounding environment.

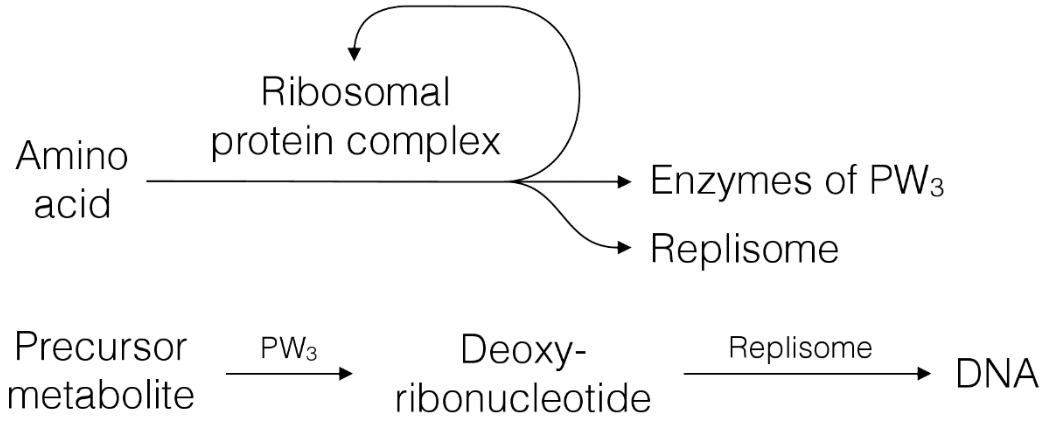

Supplementary Figure 8. Scheme of the bacterial proto-cell SSPCM-RS+DNA. The SSPCM-RS+DNA model developed includes ribosomal protein complexes (RPC), replisome complexes (RC), enzymes of the metabolic network, DNA, monomers of proteins (amino acids) and DNA (deoxyribonucleotides), precursor and intermediate metabolites which are all located in pseudo cytoplasm (proto-cell is not separated from the growth environment) (Supplementary Tables 1-2). RPC carries out synthesis of all proteins from amino acids (provided by the growth environment) without energy and RNA. RC carries out synthesis of DNA from deoxyribonucleotides without energy. Deoxyribonucleotides are synthesized by enzymes in the respective biosynthesis pathway PW_3 consisting of linear reaction chain from precursor metabolite (provided by the growth environment) via metabolic intermediates (Supplementary Table 3).

Supplementary Discussion 5.11.10.1.1: Metabolic network

It is assumed that the proto-cell converts unspecified metabolic intermediate (available in the surrounding environment) to deoxyribonucleotides of DNA via synthesis pathway PW_3 (Supplementary Figure 8, Supplementary Tables 2-3) which reactions are catalysed by identical (*generic* m_{enz}, *generic* n_{enz}) enzymes with the same *generic* k_{enz} as in SSPCM-SRS-M (Supplementary Discussion 5.11.2.1.1). Therefore, Supplementary Eq. (74) describes all fluxes of all reactions for all metabolic intermediates in PW_3.

Supplementary Discussion 5.11.10.1.2: Protein synthesis

The protein fraction of the proto-cell is comprised of the following different proteins (Supplementary Figure 8, Supplementary Table 2):

1. RPC (part of ribosomes, carries out translation).
2. Enzymes on biosynthetic pathways PW_3 (carry out the synthesis of metabolic intermediates and deoxyribonucleotides of DNA).
3. RC (carries out DNA replication).

The numbers of all proteins are doubled by ribosomes carrying out translation. It is assumed that rRNA, mRNAs, tRNA and energy are not needed for protein synthesis and an unlimited number of amino acids are available in the surrounding environment. $N_{rs\_cell\_comp}$ is determined by t_{d_rs+dna} and the requirement of amino acids of each protein according to Supplementary Eq. (80) and following Supplementary Eqs. (170)-(171) that are remarkably similar to Supplementary Eqs. (83), (87):

$$t_{d\_rs+dna} = \frac{N_{enz\_PW3\_r} \cdot l_{PW3} \cdot n_{enz}}{N_{rs\_enz} \cdot k_{rs}} \tag{170}$$

$$t_{d\_rs+dna} = \frac{N_{rc} \cdot n_{rc}}{N_{rs\_rc} \cdot k_{rs}} \tag{171}$$

N_{rs} is expressed by the following Supplementary Eq. (172):

$$N_{rs} = N_{rs\_rpc} + N_{rs\_enz} + N_{rs\_rc} \tag{172}$$

Supplementary Discussion 5.11.10.1.3: Mass balance

M_{tot} is equal to M_{cyt} and it is the sum of masses of all cell components including water:

$$M_{tot} = M_{cyt} = \frac{N_{rs} \cdot m_{rpc} + N_{enz\_PW_3\_r} \cdot l_{PW_3} \cdot m_{enz} + N_{rc} \cdot m_{rc} + N_{dna} \cdot m_{dna}}{DWC} \tag{173}$$

Supplementary Discussion 5.11.10.1.4: DNA synthesis

The genome is replicated by RCs according to Supplementary Eq. (174) which is remarkably similar to Supplementary Eq. (108):

$$t_{d\_rs+dna} = \frac{N_{dna} \cdot n_{dna}}{2 \cdot N_{rce} \cdot k_{dp}} \tag{174}$$

Supplementary Discussion 5.11.10.2: Model parameters

Corresponding input parameters, their values and output parameters of SSPCM-SRS-M (Supplementary Discussions 5.11.2.2, 5.11.2.2.1-5.11.2.2.2) are also used in the current model except input parameter t_{d_rs+dna} (independent variable) which is unique only for SSPCM-RS+DNA instead of $t_{d\_srs\text{-}m}$.

Supplementary Discussion 5.11.10.3: Calculation scheme

The calculation scheme is similar but not identical to SSPCM-SRS-M (Supplementary Discussion 5.11.2.2). The first step (solving of independent equations) involves besides the calculation of masses of cell components (Supplementary Tables 16-18, 20) etc certain equations/parameters describing DNA replication and associated polymerization that can be solved also separately (Supplementary Eqs. (74), (171), (174) and corresponding output parameters: $N_{enz\_PW3\_r}$, N_{rs_rc}, N_{rce}). All other above (Supplementary Discussions 5.11.10.1.1-5.11.10.1.4) referred and presented equations comprise the main system of equations solved during the second step.

Note that additional assumption must be formulated for DNA replication during faster growth. RCs have extra unused synthesis capabilities (*approximate* N_{rc} (Supplementary Table 24) > N_{rce}) during slower growth (t_{d_rs+dna} > *approximate* t_C (Supplementary Table 24)). However,

these capabilities are exhausted at t_{d_rs+dna} = *approximate* t_C because *approximate* $N_{rc} = N_{rce}$. Further faster growth would be impossible with the same set of input parameters because *approximate* $N_{rc} < N_{rce}$. Therefore, it must be assumed that the value of k_{dp} is increasing with μ at faster growth (t_{d_rs+dna} < *approximate* t_C) and *approximate* $N_{rc} = N_{rce}$.

Supplementary Table 37: Calculated rounded values of selected output parameters of proto-cell SSPCM-RS+DNA at different doubling time values.

Parameter	Symbol	Unit				
Doubling time	t_{d_rs+dna}	s	361.1	400	3520	$2 \cdot 10^4$
Number of cell component molecules/complexes in the cell						
Ribosome	N_{rs}	molecules (rs) cell^{-1}	$7.82 \cdot 10^{16}$	18492	27	0.96
Ribosomes for the synthesis of ribosomal protein complex	N_{rs_rpc}	molecules (rs rpc) cell^{-1}	$7.82 \cdot 10^{16}$	16740	3	0.02
Ribosomes for the synthesis of enzymes in central and biosynthesis pathways PW_1-PW_5	N_{rs_enz}	molecules (rs enz) cell^{-1}	2123	1740	22	0.70
Ribosomes for the synthesis of replisome complex (RC)	N_{rs_rc}	molecules (rs rc) cell^{-1}	14	12	1	0.24
Effective RC	N_{rce}	molecules (rce) cell^{-1}	2	2	1	0.23
Enzyme catalysing a single reaction r of ribonucleotide biosynthesis pathway PW_4	$N_{enz\_PW3\_r}$	molecules (enz PW_3) cell^{-1} reaction^{-1}	256	232	26	5
Other parameters						
Apparent working rate of DNA polymerase	k_{dp}	molecules (dnt) s^{-1} dp^{-1}	6407	5800	1000	1000
Cell mass	M_{tot}	g cell^{-1}	3.73	$1.13 \cdot 10^{-13}$	$1.71 \cdot 10^{-14}$	$1.61 \cdot 10^{-14}$

SUPPLEMENTARY DISCUSSION 5.11.11: DESCRIPTION OF SSPCM-SRS+R

The description of the model (SSPCM-SRS-R) is based on the full description of SSPCM-SRS-M (Supplementary Discussion 5.11.2), only the differences compared with SSPCM-SRS-M are subsequently presented.

Supplementary Discussion 5.11.11.1: Model components and interactions

The proto-cell is surrounded by the lipid membrane which separates the growth environment and cytoplasm (Supplementary Figure 9).

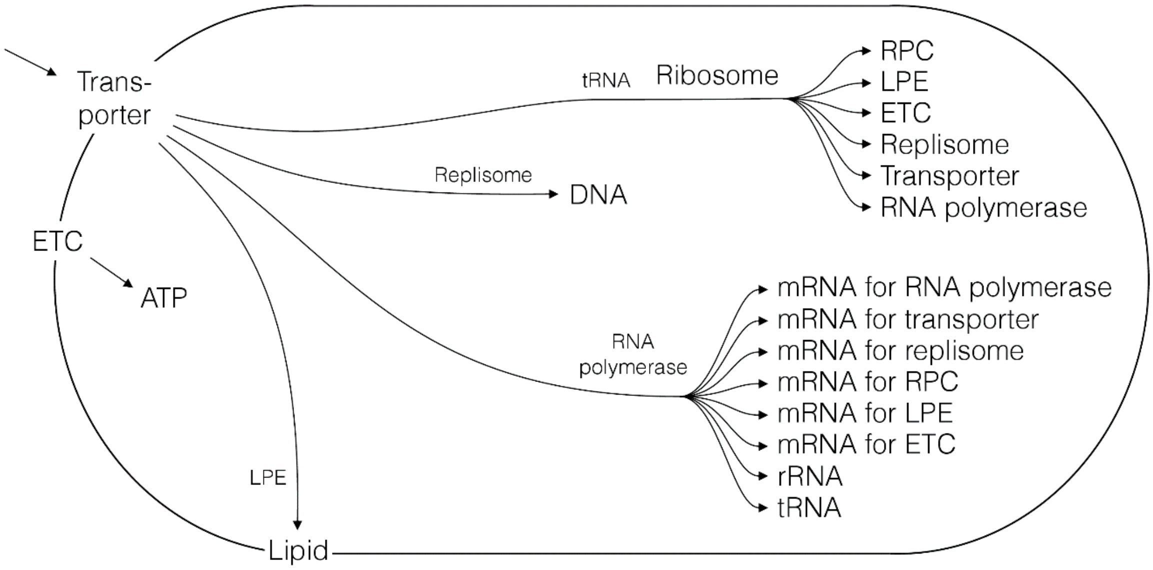

Supplementary Figure 9. Simplified bacterial proto-cell growing on rich medium (SSPCM-SRS-R). The developed model includes all the main cell components (DNA, RNA, proteins, membrane lipids) and their monomers (nucleotides, amino acids) (Supplementary Table 2). Most of the cell components are localized in the cytoplasmic space except membrane lipids, transporter proteins and electron transport chain (ETC) complexes that belong to the bilayer cell membrane (Supplementary Table 1). The genome of the cell is a bihelix circular chromosome. RNA fraction consists of different types (assembled rRNA complex, universal tRNA (not shown) and mRNAs for all proteins). Different proteins are taken into account based on their functions (polymerases, membrane proteins). The growth medium contains all the monomers for polymerisation processes (nucleotides, amino acids, lipid precursors) that are transported through the membrane by multifunctional membrane transporter. Energy is synthesized by ETC on the membrane. Monomers and energy are further utilized by polymerization processes (replication catalysed by replisome complex, transcription catalysed by RNA polymerase complex, translation catalysed by ribosome (consists of rRNA and ribosomal protein complex (RPC)), membrane lipid synthesis catalysed by lipid synthesis enzyme (LPE)) (Supplementary Table 3).

Supplementary Discussion 5.11.11.1.1: Metabolic network

It is supposed that the transport protein on the cell membrane is multifunctional and therefore able to import all different monomers into the cell (Supplementary Figure 9). The total monomer requirement is determined by the sum of all polymerization fluxes (Supplementary Eq. (175)).

$$N_{stp} \cdot k_{stp} = 2 \cdot N_{rce} \cdot k_{dp} + N_{rp} \cdot k_{rp} + N_{rs} \cdot k_{rs} + N_{lpe} \cdot k_{lpe} \quad (175)$$

Coefficient 2 takes into account the number of DNA polymerase III molecules per RC (Supplementary Table 15). Compared to SSPCM-SRS-M, Supplementary Eq. (175) of SSPCM-SRS-R replaces all equations of SSPCM-SRS-M from Supplementary Discussion 5.11.2.1.1 due to the lack of central and biosynthesis pathways PW_1-PW_5. Therefore, Supplementary Eqs. (73)-(78) are not taken into account due to the same reason.

Supplementary Discussion 5.11.11.1.2: Protein synthesis

The protein fraction of the proto-cell is comprised of the following different proteins (Supplementary Figure 9, Supplementary Table 2):

1. RP complex (carries out transcription).
2. RPC (part of ribosomes, carries out translation).
3. LPE (membrane lipid synthesis).
4. ETC complex (energy production on the membrane).
5. Transport protein (substrate transport on the membrane).
6. RC (carries out DNA replication).

The numbers of all proteins are doubled by ribosomes carrying out translation. $N_{rs\_cell\_comp}$ is determined by $t_{d\_srs\text{-}r}$ and the requirement of amino acids of each protein according to Supplementary Eq. (80) and following Supplementary Eqs. (176)-(180) that are remarkably similar to Supplementary Eq. (82), (84)-(87):

$$t_{d\_srs-r} = \frac{N_{rp} \cdot n_{rp}}{N_{rs\_rp} \cdot k_{rs}} \quad (176)$$

$$t_{d\_srs-r} = \frac{N_{lpe} \cdot n_{lpe}}{N_{rs\_lpe} \cdot k_{rs}} \quad (177)$$

$$t_{d\_srs-r} = \frac{N_{etc} \cdot n_{etc}}{N_{rs\_etc} \cdot k_{rs}} \quad (178)$$

$$t_{d\_srs-r} = \frac{N_{stp} \cdot n_{stp}}{N_{rs\_stp} \cdot k_{rs}} \quad (179)$$

$$t_{d\_srs-r} = \frac{N_{rc} \cdot n_{rc}}{N_{rs\_rc} \cdot k_{rs}} \quad (180)$$

N_{rs} is expressed by the following Supplementary Eq. (181):

$$N_{rs} = N_{rs\_rc} + N_{rs\_rp} + N_{rs\_rpc} + N_{rs\_etc} + N_{rs\_stp} + N_{rs\_lpe} \quad (181)$$

Supplementary Discussion 5.11.11.1.3: RNA synthesis

RNA fraction of the proto-cell is comprised of the following different RNAs (Supplementary Figure 9, Supplementary Table 2):

1. Ribosomal RNA complex is based on 3 subunits (5S, 16S, 23S) assembled in ribosomes with 1:1:1 stoichiometry.
2. Transfer RNA. The model was simplified by introducing only one universal tRNA molecule because different amino acids are not specified.
3. There are specific mRNAs coding different proteins in the model. It is assumed that mRNAs contain only coding regions to simplify the model.

Numbers of all RNA molecules are doubled by RP complexes carrying out transcription. Transcription is described by following Supplementary Eqs. (182)-(183) that are remarkably similar to Supplementary Eqs. (90)-(91):

$$t_{d\_srs-r} = \frac{N_{rrna} \cdot n_{rrna} + N_{trna} \cdot n_{trna} + \sum_{1}^{6} N_{mrna\_cell\_comp} \cdot n_{mrna\_cell\_comp}}{N_{rp} \cdot k_{rp}} \tag{182}$$

$$\sum_{1}^{6} N_{mrna\_cell\_comp} \cdot n_{mrna\_cell\_comp} = N_{mrna\_rc} \cdot n_{mrna\_rc} + N_{mrna\_rp} \cdot n_{mrna\_rp} + N_{mrna\_rpc} \cdot n_{mrna\_rpc} + \\ + N_{mrna\_etc} \cdot n_{mrna\_etc} + N_{mrna\_stp} \cdot n_{mrna\_stp} + N_{mrna\_lpe} \cdot n_{mrna\_lpe} \tag{183}$$

The stoichiometry of the ribosome is described by Supplementary Eq. (92) and tRNA balances by Supplementary Eq. (93). $N_{mrna\_cell\_comp}$ values are determined by translational activity and also by P_{cell_comp} (Supplementary Eqs. (94)-(100)).

Supplementary Discussion 5.11.11.1.4: Lipid synthesis

Proto-cell contains only one type of membrane lipid that forms a bilayer cell membrane (Supplementary Figure 9, Supplementary Tables 1-2). The value of N_{lip} is doubled by LPE according to the following Supplementary Eq. (184) which is remarkably similar to Supplementary Eq. (101):

$$t_{d\_srs-r} = \frac{N_{lip}}{N_{lpe} \cdot k_{lpe}} \tag{184}$$

Supplementary Discussion 5.11.11.1.5: Energy balance

It is assumed that energy costs do not include translation expenditures for monomer synthesis. Therefore, energy balance is described by Supplementary Eq. (185) instead of Supplementary Eq. (102):

$$N_{etc} \cdot k_{etc} = N_{stp} \cdot k_{stp} \cdot X_{stp} + 2 \cdot N_{rce} \cdot k_{kdp} \cdot X_{dna} + N_{rp} \cdot k_{rp} \cdot X_{rna} + N_{lpe} \cdot k_{lpe} \cdot X_{lip} + N_{rs} \cdot k_{rs} \cdot X_{prot} \tag{185}$$

Supplementary Discussion 5.11.11.1.6: Cell geometry

The geometry of the proto-cell is the same as in SSPCM-SRS-M (Supplementary Discussion 5.11.2.1.6).

Supplementary Discussion 5.11.11.1.7: Mass balance

M_{tot} is described by Supplementary Eq. (105). M_{cyt} equals the sum of masses of all cell components (including water) localized in cytoplasmic space:

$$M_{cyt} = \frac{N_{rc} \cdot m_{rc} + N_{rp} \cdot m_{rp} + N_{rs} \cdot m_{rpc} + N_{lpe} \cdot m_{lpe} + N_{rrna} \cdot m_{rrna} + N_{trna} \cdot m_{trna}}{DWC} + \\ + \frac{\sum_{1}^{6} N_{mrna\_cell\_comp} \cdot m_{mrna\_cell\_comp} + N_{dna} \cdot m_{dna}}{DWC} \tag{186}$$

$$\sum_{1}^{6} N_{mrna\_cell\_comp} \cdot m_{mrna\_cell\_comp} = N_{mrna\_rc} \cdot m_{mrna\_rc} + N_{mrna\_rp} \cdot m_{mrna\_rp} + N_{mrna\_rpc} \cdot m_{mrna\_rpc} + \\ + N_{mrna\_etc} \cdot m_{mrna\_etc} + N_{mrna\_stp} \cdot m_{mrna\_stp} + N_{mrna\_lpe} \cdot m_{mrna\_lpe} \tag{187}$$

Supplementary Discussion 5.11.11.1.8: DNA synthesis

The genome is replicated by RCs according to Supplementary Eq. (188) which is remarkably similar to Supplementary Eq. (108):

$$t_{d\_srs-r} = \frac{N_{dna} \cdot n_{dna}}{2 \cdot N_{rce} \cdot k_{dp}} \tag{188}$$

Supplementary Discussion 5.11.11.2: Model parameters

Corresponding input parameters, their values and output parameters of SSPCM-SRS-M (Supplementary Discussions 5.11.2.2, 5.11.2.2.1-5.11.2.2.2) are also used in the current model except those of metabolic network (k_{enz}, l_{PWi}, n_{enz}, m_{enz}, X_{PWi}, $F_{enz\_PWi\_r}$) and corresponding macromolecules (subset of ribosomes, mRNA – P_{enz}, n_{mrna_enz}, m_{mrna_enz}, N_{rs_enz}, N_{mrna_enz}) that are excluded. Also, SSPCM-SRS-R includes input parameter t_{d_srs-r} (independent variable) missing in SSPCM-SRS-M.

Supplementary Discussion 5.11.11.3: Calculation scheme

The calculation scheme is similar but not identical to SSPCM-SRS-M (Supplementary Discussion 5.11.2.3). The first step (solving of independent equations) involves besides the calculation of masses of cell components (Supplementary Tables 16-20), lengths of mRNA sequences (Supplementary Table 10) etc certain equations/parameters describing DNA replication and associated polymerization that can be solved also separately (Supplementary Eqs. (94), (180), (188) and corresponding output parameters: N_{mrna_rc}, N_{rs_rc}, N_{rce}). All other above (Supplementary Discussions 5.11.11.1.1-5.11.11.1.8) referred and presented equations comprise the main system of equations solved during the second step.

Note that additional assumption must be formulated for DNA replication during faster growth. RCs have extra unused synthesis capabilities (*approximate* N_{rc} (Supplementary Table 24) > N_{rce}) during slower growth (t_{d_srs-r} > *approximate* t_C (Supplementary Table 24)). However, these capabilities are exhausted at t_{d_srs-r} = *approximate* t_C because *approximate* $N_{rc} = N_{rce}$. Further faster growth would be impossible with the same set of input parameters because *approximate* $N_{rc} < N_{rce}$. Therefore, it must be assumed that the value of k_{dp} is increasing with μ at faster growth (t_{d_srs-r} < *approximate* t_C) and *approximate* $N_{rc} = N_{rce}$.

Supplementary Table 38: Calculated rounded values of selected output parameters of proto-cell SSPCM-SRS-R at different doubling time values.

**Parameter**	**Symbol**	**Unit**			
Doubling time	$t_{d\_srs\text{-}r}$	s	936.40	3520	$1.3 \cdot 10^4$
Number of cell component molecules/complexes in the cell					
Ribosome	N_{rs}	molecules (rs) cell^{-1}	24681	13	1
Ribosomes for the synthesis of ribosomal protein complex (RPC)	N_{rs_rpc}	molecules (rs rpc) cell^{-1}	9544	1	0.03
Ribosomes for the synthesis of RNA polymerase (RP) complex	N_{rs_rp}	molecules (rs rp) cell^{-1}	1021	0.04	$3 \cdot 10^{-3}$
Ribosomes for the synthesis of lipid synthesis enzyme (LPE)	N_{rs_lpe}	molecules (rs lpe) cell^{-1}	$4 \cdot 10^{-5}$	0.02	10^{-3}
Ribosomes for the synthesis of electron transport chain (ETC)	N_{rs_etc}	molecules (rs etc) cell^{-1}	13426	9	0.59
Ribosomes for the synthesis of substrate transport protein	N_{rs_stp}	molecules (rs stp) cell^{-1}	684	0.93	0.06
Ribosomes for the synthesis of replisome complex (RC)	N_{rs_rc}	molecules (rs rc) cell^{-1}	5	1	0.38
RP	N_{rp}	molecules (rp) cell^{-1}	3432	0.49	0.01
LPE	N_{lpe}	molecules (lpe) cell^{-1}	$3 \cdot 10^{-3}$	3	0.93
ETC	N_{etc}	molecules (etc) cell^{-1}	25144	65	15
Substrate transport protein	N_{stp}	molecules (stp) cell^{-1}	6408	33	8
Effective RC	N_{rce}	molecules (rce) cell^{-1}	2	1	0.36
Assembled rRNA complex	N_{rrna}	molecules (rrna) cell^{-1}	24681	13	1
tRNA	N_{trna}	molecules (trna) cell^{-1}	123403	65	5
mRNA of RPC	N_{mrna_rpc}	molecules (mrna rpc) cell^{-1}	95	0.01	$3 \cdot 10^{-3}$
mRNA of RP	N_{mrna_rp}	molecules (mrna rp) cell^{-1}	10	$4 \cdot 10^{-4}$	$3 \cdot 10^{-6}$
mRNA of LPE	N_{mrna_lpe}	molecules (mrna lpe) cell^{-1}	$2 \cdot 10^{-6}$	$7 \cdot 10^{-4}$	$5 \cdot 10^{-5}$
mRNA of ETC	N_{mrna_etc}	molecules (mrna etc) cell^{-1}	134	0.09	$6 \cdot 10^{-3}$
mRNA of substrate transport protein	N_{mrna_stp}	molecules (mrna stp) cell^{-1}	7	$9 \cdot 10^{-3}$	$6 \cdot 10^{-4}$
mRNA of RC	N_{mrna_rc}	molecules (mrna rc) cell^{-1}	0.05	0.01	$4 \cdot 10^{-3}$
Membrane lipid	N_{lip}	molecules (lip) cell^{-1}	242	$1.20 \cdot 10^6$	$1.21 \cdot 10^6$
Other parameters					

Apparent working rate of DNA polymerase	k_{dp}	molecules (dnt) s^{-1} dp^{-1}	2477.4	1000	1000
Mass of cytoplasm of the cell	M_{cyt}	g cyt^{-1}	$3.75 \cdot 10^{-13}$	$1.61 \cdot 10^{-14}$	$1.59 \cdot 10^{-14}$
Cell mass	M_{tot}	g $cell^{-1}$	$5.49 \cdot 10^{-13}$	$1.91 \cdot 10^{-14}$	$1.86 \cdot 10^{-14}$

Supplementary References

1. Schleif, R. Control of production of ribosomal protein. *J. Mol. Biol.* **27**, 41-55 (1967).
2. Kostinski, S. & Reuveni, S. Ribosome composition maximizes cellular growth rates in *E. coli*. *Phys. Rev. Lett.* **125**, 028103 (2020).
3. Abner, K., Aaviksaar, T., Adamberg, K. & Vilu, R. Single-cell model of prokaryotic cell cycle. *J. Theor. Biol.* **341**, 78-87 (2014).
4. Goelzer, A. & Fromion, V. Bacterial growth rate reflects a bottleneck in resource allocation. *Biochim. Biophys. Acta* **1810**, 978-988 (2011).
5. Bleecken, S. Duplication of the bacterial cell and its initiation. *J. Theor. Biol.* **25**, 137-158 (1969).
6. Kafri, M., Metzl-Raz, E., Jonas, F. & Barkai, N. Rethinking cell growth models. *FEMS Yeast Res.* **16**, fow081 (2016).
7. Cooper, S. & Helmstetter, C. E. Chromosome replication and the division cycle of *Escherichia coli* B/r. *J. Mol. Biol.* **31**, 519-540 (1968).
8. Calabrese, L., Grilli, J., Osella, M., Kempes, C. P., Lagomarsino, M. C. & Ciandrini, L. Protein degradation sets the fraction of active ribosomes at vanishing growth. *PLoS Comput. Biol.* **18**, e1010059 (2022).
9. Ehrenberg, M., Bremer, H. & Dennis, P. P. Medium-dependent control of the bacterial growth rate. *Biochimie* **95**, 643-658 (2013).
10. Schrum, J. P, Zhu, T. F. & Szostak J. W. The origins of cellular life. *Cold Spring Harb. Perspect. Biol.* **2**, 002212 (2010).
11. Kuzenkov, O. & Morozov, A. Towards the construction of a mathematically rigorous framework for the modelling of evolutionary fitness. *Bull. Math. Biol.* **81**, 4675-4700 (2019).
12. Sakuma, Y. & Imai, M. From vesicles to protocells: the roles of amphiphilic molecules. *Life* **5**, 651-675 (2015).
13. Ueno, T. Functionalization of viral protein assemblies by self-assembly reactions. *J. Mater. Chem.* **18**, 3741-3745 (2008).
14. Morçöl, G. Chapter 4: Self-Organization. in *A Complexity Theory for Public Policy* 93-121 (Routledge, New York, 2012).
15. Kempes, C.P., Wang, L., Amend, J. P., Doyle, J. & Hoehler, T. Evolutionary tradeoffs in cellular composition across diverse bacteria. *ISME J.* **10**, 2145-2157 (2016).
16. Maaløe, O. & Kjeldgaard, N. O. *Control of Macromolecular Synthesis: a Study of DNA, RNA, and Protein Synthesis in Bacteria* (W. A. Benjamin, New York, 1966).
17. Belliveau, N. M., Chure, G., Hueschen, C. L., Garcia, H. G., Kondev, J., Fisher, D. S., Theriot, J. A. & Phillips, R. Fundamental limits on the rate of bacterial growth and their influence on proteomic composition. *Cell Syst.* **12**, 924-944 (2021).
18. Dill, K. A., Ghosh, K. & Schmit, J. D. Physical limits of cells and proteomes. *Proc. Natl. Acad. Sci. U. S. A.* **108**, 17876-17882 (2011).
19. Milo, R., Jorgensen, P., Moran, U., Weber, G. & Springer, M. BioNumbers--the database of key numbers in molecular and cell biology. *Nucleic Acids Res.* **38**, D750-753 (2010).
20. Zaslaver, A., Kaplan, S., Bren, A., Jinich, A., Mayo, A., Dekel, E., Alon, U. & Itzkovitz, S. Invariant distribution of promoter activities in *Escherichia coli*. *PLoS Comput. Biol.* **5**, e1000545 (2009).

21. Paalme, T., Elken, R., Kahru, A., Vanatalu, K. & Vilu, R. The growth rate control in *Escherichia coli* at near to maximum growth rates: the A-stat approach. *Antonie Van Leeuwenhoek* **71**, 217-230 (1997).
22. Bremer, H. & Dennis, P. P. Modulation of chemical composition and other parameters of the cell by growth rate. in *Escherichia coli and Salmonella typhimurium: Cellular and Molecular Biology, Volume 2* (eds Neidhardt, F. C., Ingraham, J. L., Brooks Low, K., Magasanik, B., Schaechter, M. & Umbarger, H. E.) 1527–1542 (American Society for Microbiology, Washington, D.C., 1987).
23. Pandey, P. P. & Jain, S. Analytic derivation of bacterial growth laws from a simple model of intracellular chemical dynamics. *Theory Biosci*. **135**, 121-130 (2016).
24. Maitra, A. & Dill, K. A. Bacterial growth laws reflect the evolutionary importance of energy efficiency. *Proc*. *Natl*. *Acad*. *Sci*. *U*. *S*. *A*. **112**, 406-411 (2015).
25. Kempes, C. P., Wolpert, D., Cohen, Z. & Pérez-Mercader, J. The thermodynamic efficiency of computations made in cells across the range of life. *Philos*. *Trans*. *A Math*. *Phys*. *Eng*. *Sci*. **375**, 20160343 (2017).
26. Metzl-Raz, E., Kafri, M., Yaakov, G., Soifer, I., Gurvich, Y. & Barkai, N. Principles of cellular resource allocation revealed by condition-dependent proteome profiling. *Elife* **6**, e28034 (2017).
27. Roy, A., Goberman, D. & Pugatch, R. A unifying autocatalytic network-based framework for bacterial growth laws. *Proc*. *Natl*. *Acad*. *Sci*. *U*. *S*. *A*. **118**, e2107829118 (2021).
28. Hu, X. P. & Lercher, M. J. An optimal growth law for RNA composition and its partial implementation through ribosomal and tRNA gene locations in bacterial genomes. *PLoS Genet*. **17**, e1009939 (2021).
29. Bremer, H. & Dennis P. P. Modulation of chemical composition and other parameters of the cell at different exponential growth rates. *EcoSal Plus* **3** (2008).
30. Volkmer, B. & Heinemann, M. Condition-dependent cell volume and concentration of *Escherichia coli* to facilitate data conversion for systems biology modeling. *PLoS One* **6**, e23126 (2011).
31. Bipatnath, M., Dennis, P. P. & Bremer, H. Initiation and velocity of chromosome replication in *Escherichia coli* B/r and K-12. *J*. *Bacteriol*. **180**, 265-273 (1998).
32. Adamberg, K., Valgepea, K. & Vilu, R. Advanced continuous cultivation methods for systems microbiology. *Microbiology* **161**, 1707-1719 (2015).
33. Labbe, R. G. & Huang, T. H. Generation times and modeling of enterotoxin-positive and enterotoxin-negative strains of *Clostridium perfringens* in laboratory media and ground beef. *J*. *Food Prot*. **58**, 1303-1306 (1995).
34. Shimizu, T., Ohshima, S., Ohtani, K., Shimizu, T. & Hayashi, H. Genomic map of *Clostridium perfringens* strain 13. *Microbiol*. *Immunol*. **45**, 179-189 (2001).
35. Kubitschek, H. E. Cell volume increase in *Escherichia coli* after shifts to richer media. *J*. *Bacteriol*. **172**, 94-101 (1990).
36. Kubitschek, H. E. Increase in cell mass during the division cycle of *Escherichia coli* B/rA. *J*. *Bacteriol*. **168**, 613-618 (1986).
37. Koppes, L. J., Woldringh, C. L. & Grover, N. B. Predicted steady-state cell size distributions for various growth models. *J*. *Theor*. *Biol*. **129**, 325-335 (1987).
38. Grover, N. B., Woldringh, C. L. & Koppes, L. J. Elongation and surface extension of individual cells of *Escherichia coli* B/r: comparison of theoretical and experimental size distributions. *J*. *Theor*. *Biol*. **129**, 337-348 (1987).
39. Cooper, S. What is the bacterial growth law during the division cycle? *J*. *Bacteriol*. **170**, 5001-5005 (1988).

40. Grover, N. B., Eidelstein, E. & Koppes, L. J. Bacterial shape maintenance: an evaluation of various models. *J. Theor. Biol.* **227**, 547-559 (2004).
41. Reshes, G., Vanounou, S., Fishov, I. & Feingold, M. Cell shape dynamics in *Escherichia coli*. *Biophys. J.* **94**, 251-264 (2008).
42. Jun, S., Si, F., Pugatch, R. & Scott, M. Fundamental principles in bacterial physiology-history, recent progress, and the future with focus on cell size control: a review. *Rep. Prog. Phys.* **81**, 056601 (2018).
43. Painter, P. R. & Marr, A. G. Mathematics of microbial populations. *Annu. Rev. Microbiol.* **22**, 519-548 (1968).
44. Schuster, S., Pfeiffer, T. & Fell, D. A. Is maximization of molar yield in metabolic networks favoured by evolution? *J. Theor. Biol.* **252**, 497-504 (2008).
45. Tempest, D. W. & Neijssel, O. M. The status of YATP and maintenance energy as biologically interpretable phenomena. *Annu. Rev. Microbiol.* **38**, 459-486 (1984).
46. Kochanowski, K., Sauer, U. & Noor, E. Posttranslational regulation of microbial metabolism. *Curr. Opin. Microbiol.* **27**, 10-17 (2015).
47. Huang, K. C., Mukhopadhyay, R., Wen, B., Gitai, Z. & Wingreen, N. S. Cell shape and cell-wall organization in Gram-negative bacteria. *Proc. Natl. Acad. Sci. U. S. A.* **105**, 19282-19287 (2008).
48. Dennis, P. P. & Bremer, H. Macromolecular composition during steady-state growth of *Escherichia coli* B-r. *J. Bacteriol.* **119**, 270-281 (1974).
49. Scott, M., Klumpp, S., Mateescu, E. M. & Hwa, T. Emergence of robust growth laws from optimal regulation of ribosome synthesis. *Mol. Syst. Biol.* **10**, 747 (2014).
50. Kempes, C. P., van Bodegom, P. M., Wolpert, D., Libby, E., Amend, J. & Hoehler, T. Drivers of bacterial maintenance and minimal energy requirements. *Front. Microbiol.* **8**, 31 (2017).
51. Davydov, I. I., Wohlgemuth, I., Artamonova, I. I., Urlaub, H., Tonevitsky, A. G. & Rodnina, M. V. Evolution of the protein stoichiometry in the L12 stalk of bacterial and organellar ribosomes. *Nat. Commun.* **4**, 1387 (2013).
52. Slavov, N., Semrau, S., Airoldi, E., Budnik, B. & van Oudenaarden, A. Differential stoichiometry among core ribosomal proteins. *Cell Rep.* **13**, 865-873 (2015).
53. Reyes-Lamothe, R., Sherratt, D. J. & Leake, M. C. Stoichiometry and architecture of active DNA replication machinery in *Escherichia coli*. *Science* **328**, 498-501 (2010).
54. Vogel, U. & Jensen, K. F. The RNA chain elongation rate in *Escherichia coli* depends on the growth rate. *J. Bacteriol.* **176**, 2807-2813 (1994).
55. Gotta, S. L., Miller, O. L. Jr & French, S. L. rRNA transcription rate in *Escherichia coli*. *J. Bacteriol.* **173**, 6647-6649 (1991).
56. Young, R. & Bremer, H. Polypeptide-chain-elongation rate in *Escherichia coli* B/r as a function of growth rate. *Biochem. J.* **160**, 185-194 (1976).
57. Reyes-Lamothe, R., Wang, X. & Sherratt, D. *Escherichia coli* and its chromosome. *Trends Microbiol.* **16**, 238-245 (2008).
58. Neidhardt, F. C., Ingraham, J. L. & Schaechter, M. Chapter 1: Composition and organization of the bacterial cell. in *Physiology of the Bacterial Cell: a Molecular Approach* 1-29 (Sinauer Associates, Sunderland, 1990).
59. Neidhardt, F. C., Ingraham, J. L. & Schaechter, M. Chapter 5: Biosynthesis and fueling. in *Physiology of the Bacterial Cell: a Molecular Approach* 133-173 (Sinauer Associates, Sunderland, 1990).

60. Neidhardt, F. C., Ingraham, J. L. & Schaechter, M. Chapter 3: Assembly and polymerization: the bacterial interior. in *Physiology of the Bacterial Cell: a Molecular Approach* 62-101 (Sinauer Associates, Sunderland, 1990).
61. Neidhardt, F. C., Ingraham, J. L. & Schaechter, M. Chapter 4: Assembly and polymerization: the bacterial envelope. in *Physiology of the Bacterial Cell: a Molecular Approach* 102-132 (Sinauer Associates, Sunderland, 1990).
62. Konings, W. N. The cell membrane and the struggle for life of lactic acid bacteria. *Antonie Van Leeuwenhoek* **82**, 3-27 (2002).
63. Russell, J. B. & Cook, G. M. Energetics of bacterial growth: balance of anabolic and catabolic reactions. *Microbiol. Rev.* **59**, 48-62 (1995).
64. Kanehisa, M. & Goto, S. KEGG: kyoto encyclopedia of genes and genomes. *Nucleic Acids Res.* **28**, 27-30 (2000).
65. Blattner, F. R., Plunkett, G. 3rd, Bloch, C. A., Perna, N. T., Burland, V., Riley, M., Collado-Vides, J., Glasner, J. D., Rode, C. K., Mayhew, G. F., Gregor, J., Davis, N. W., Kirkpatrick, H. A., Goeden, M. A., Rose, D. J., Mau, B. & Shao, Y. The complete genome sequence of *Escherichia coli* K-12. *Science* **277**, 1453-1462 (1997).
66. Hardy, S. J. The stoichiometry of the ribosomal proteins of *Escherichia coli*. *Mol. Gen. Genet.* **140**, 253-274 (1975).
67. Izutsu, K., Wada, C., Komine, Y., Sako, T., Ueguchi, C., Nakura, S. & Wada A. *Escherichia coli* ribosome-associated protein SRA, whose copy number increases during stationary phase. *J. Bacteriol.* **183**, 2765-2773 (2001).
68. Kansara, S. G. & Sukhodolets, M. V. Oligomerization of the *E. coli* core RNA polymerase: formation of (α2ββ'ω)2-DNA complexes and regulation of the oligomerization by auxiliary subunits. *PLoS One* **6**, e18990 (2011).
69. Johnson, A. & O'Donnell, M. Cellular DNA replicases: components and dynamics at the replication fork. *Annu. Rev. Biochem.* **74**, 283-315 (2005).
70. Tsuchihashi, Z. & Kornberg, A. Translational frameshifting generates the gamma subunit of DNA polymerase III holoenzyme. *Proc. Natl. Acad. Sci. U. S. A.* **87**, 2516-2520 (1990).
71. López de Saro, F. J. & O'Donnell, M. Interaction of the beta sliding clamp with MutS, ligase, and DNA polymerase I. *Proc. Natl. Acad. Sci. U. S. A.* **98**, 8376-8380 (2001).
72. Kornberg, A. Enzyme studies of replication of the *Escherichia coli* chromosome. *Adv. Exp. Med. Biol.* **179**, 3-16 (1984).
73. Sissi, C. & Palumbo, M. In front of and behind the replication fork: bacterial type IIA topoisomerases. *Cell Mol. Life Sci.* **67**, 2001-2024 (2010).
74. Leonard, A. C. & Grimwade, J. E. Regulating DnaA complex assembly: it is time to fill the gaps. *Curr. Opin. Microbiol.* **13**, 766-772 (2010).
75. Wahle, E., Lasken, R. S. & Kornberg, A. The dnaB-dnaC replication protein complex of *Escherichia coli*. I. Formation and properties. *J. Biol. Chem.* **264**, 2463-2468 (1989).
76. Allen, G. C. Jr & Kornberg, A. Assembly of the primosome of DNA replication in *Escherichia coli*. *J. Biol. Chem.* **268**, 19204-19209 (1993).
77. Mizukoshi, T., Tanaka, T., Arai, K., Kohda, D. & Masai, H. A critical role of the 3' terminus of nascent DNA chains in recognition of stalled replication forks. *J. Biol. Chem.* **278**, 42234-42239 (2003).
78. Sandler, S. J. Multiple genetic pathways for restarting DNA replication forks in *Escherichia coli* K-12. *Genetics* **155**, 487-497 (2000).

79. Teplyakov, A., Lim, K., Zhu, P. P., Kapadia, G., Chen, C. C., Schwartz, J., Howard, A., Reddy, P. T., Peterkofsky, A. & Herzberg, O. Structure of phosphorylated enzyme I, the phosphoenolpyruvate:sugar phosphotransferase system sugar translocation signal protein. *Proc. Natl. Acad. Sci. U. S. A.* **103**, 16218-16223 (2006).
80. Meins, M., Zanolari, B., Rosenbusch, J. P. & Erni, B. Glucose permease of *Escherichia coli*. Purification of the IIGlc subunit and functional characterization of its oligomeric forms. *J. Biol. Chem.* **263**, 12986-12993 (1988).
81. Borisov, V. B., Murali, R., Verkhovskaya, M. L., Bloch, D. A., Han, H., Gennis, R. B. & Verkhovsky, M. I. Aerobic respiratory chain of *Escherichia coli* is not allowed to work in fully uncoupled mode. *Proc. Natl. Acad. Sci. U. S. A.* **108**, 17320-17324 (2011).
82. Schulte, M., Mattay, D., Kriegel, S., Hellwig, P. & Friedrich, T. Inhibition of *Escherichia coli* respiratory complex I by Zn(2+). *Biochemistry* **53**, 6332-6339 (2014).
83. Al-Attar, S., Yu, Y., Pinkse, M., Hoeser, J., Friedrich, T., Bald, D. & de Vries, S. Cytochrome bd displays significant quinol peroxidase activity. *Sci. Rep.* **6**, 27631 (2016).
84. Steigmiller, S., Turina, P. & Gräber, P. The thermodynamic H+/ATP ratios of the H+-ATPsynthases from chloroplasts and *Escherichia coli*. *Proc. Natl. Acad. Sci. U. S. A.* **105**, 3745-3750 (2008).
85. Silhavy, T. J., Kahne, D. & Walker, S. The bacterial cell envelope. *Cold Spring Harb. Perspect. Biol.* **2**, a000414 (2010).
86. Andersson, K. M. & Hovmöller, S. The average atomic volume and density of proteins. *Z. Kristallogr.* **213**, 369-373 (1998).
87. Kucerka, N., Nagle, J. F., Sachs, J. N., Feller, S. E., Pencer, J., Jackson, A. & Katsaras, J. Lipid bilayer structure determined by the simultaneous analysis of neutron and X-ray scattering data. *Biophys. J.* **95**, 2356-2367 (2008).
88. Nagle, J. F. & Tristram-Nagle, S. Structure of lipid bilayers. *Biochim. Biophys. Acta* **1469**, 159-195 (2000).
89. Mohammad, F., Green, R. & Buskirk, A. R. A systematically-revised ribosome profiling method for bacteria reveals pauses at single-codon resolution. *Elife.* **8**, e42591 (2019).
90. Bratbak, G. & Dundas I. Bacterial dry matter content and biomass estimations. *Appl. Environ. Microbiol.* **48**, 755-757 (1984).
91. Grover, N. B. & Woldringh, C. L. Dimensional regulation of cell-cycle events in *Escherichia coli* during steady-state growth. *Microbiology* **147**, 171-181 (2001).
92. Cooper, S. The constrained hoop: an explanation of the overshoot in cell length during a shift-up of *Escherichia coli*. *J. Bacteriol.* **171**, 5239-5243 (1989).
